# An integrative assessment of the diversity, phylogeny, distribution, and conservation of the terrestrial reptiles (Sauropsida, Squamata) of the United Arab Emirates

**DOI:** 10.1371/journal.pone.0216273

**Published:** 2019-05-02

**Authors:** Bernat Burriel-Carranza, Pedro Tarroso, Johannes Els, Andrew Gardner, Pritpal Soorae, Ahmed Ali Mohammed, Sai Ravi Krishna Tubati, Mohamed Mustafa Eltayeb, Junid Nazeer Shah, Héctor Tejero-Cicuéndez, Marc Simó-Riudalbas, Juan Manuel Pleguezuelos, Daniel Fernández-Guiberteau, Jiří Šmíd, Salvador Carranza

**Affiliations:** 1 Institute of Evolutionary Biology (CSIC-Universitat Pompeu Fabra), Passeig Marítim de la Barceloneta, Barcelona, Spain; 2 CIBIO/InBIO, Research Centre in Biodiversity and Genetic Resources, Universidade do Porto, Campus Agrário de Vairão, Rua Padre Armando Quintas, Vairão, Vila do Conde, Portugal; 3 Breeding Centre for Endangered Arabian Wildlife, Environment and Protected Areas Authority, Sharjah, United Arab Emirates; 4 School of Molecular Sciences, University of Western Australia, Crawley, Western Australia, Australia; 5 Environment Agency, Abu Dhabi, United Arab Emirates; 6 Natural Resource Conservation Section, Environment Department, Dubai Municipality, Dubai, United Arab Emirates; 7 Departamento de Zoología, Facultad de Ciencias, Universidad de Granada, Granada, Spain; 8 Grup de Recerca de l’Escola de Natura de Parets, Parets del Vallès, Spain; 9 Department of Zoology, National Museum, Prague, Czech Republic; 10 Department of Zoology, Faculty of Science, Charles University, Prague, Czech Republic; State Museum of Natural History, GERMANY

## Abstract

In the present study we use an unprecedented database of 5,535 distributional records to infer the diversity, ecological preferences and spatial distribution of the 60 species of terrestrial reptiles of the United Arab Emirates (UAE), and use the 57 native species to test the effectiveness of the protected areas’ network in conserving this unique vertebrate fauna. We infer a time-calibrated phylogeny with 146 species of squamates and 15 genes including all UAE terrestrial reptile species to determine the phylogenetic diversity (PD) and evolutionary distinctiveness (ED) of the native species and to compare it with the distribution of the hotspots of native species richness. The results of this study indicate that the sampling effort is remarkable, covering 75% of the country’s territory representing nearly the entire climatic space of the UAE defined by the mean annual temperature and the total annual precipitation, as well as the multivariate climatic space defined by a principal component analysis (PCA). Species richness is highest in the northeast of the country, in a transitional area from sandy desert to the mountainous terrain of the Hajar Mountains. The highest PD of a single square cell of 10 arc-minutes grid is of 2,430 million years (my) of accumulated evolutionary history and the strong correlation between PD and species richness suggests that the raw number of species is a good surrogate to quantify the evolutionary history (i.e., PD). The species with the highest values of ED are those in families represented by only one species in the UAE. Finally, the assessment of the UAE protected areas shows that, despite their relevance in protecting the terrestrial reptiles, they do not offer adequate protection for some threatened species. Therefore, a reassessment of some of the protected areas or the creation of species specific conservation action plans are recommended in order to ensure the preservation of the unique diversity of UAE terrestrial reptiles.

## Introduction

Reptiles, a paraphyletic group including all non-avian sauropsids, are considered to be excellent models for evolutionary, biogeographic, ecologic, and conservation studies [1, 2]. With 10,793 species described to date [3], reptiles constitute the most diverse group of tetrapods but the continuous discovery of new species (i.e. 154 new species have been described since October 2017 and more than 1,000 since 2013 [3, 4]) suggests that there are still high levels of hidden diversity in this group. Reptiles have successfully colonized most areas of the world including the oceans [2] and they have played a very important role in the origin and later radiation of other amniote vertebrates [5]. As ectotherms, reptiles are greatly affected by the thermal landscapes of their habitat and have accumulated a great diversity of morphological, behavioral, physiological and ecological strategies to confront and adapt to the different demands of the ecosystems that they inhabit.

Even though reptiles are the most diverse group of tetrapods, the knowledge on their conservation status is lesser than other groups such as birds, mammals or amphibians. Approximately 40% of the world’s reptile species have not had their conservation status assessed by the International Union for Conservation of Nature (IUCN). Furthermore, studies indicate that the assessment of the conservation status of reptiles is biased against those species that are harder to find such as nocturnal species, species with small body or clutch size, species with restricted distributions (usually more susceptible to threats) or species located in southern latitudes [6].

Protecting biological diversity with limited resources requires placing conservation priorities on different taxa. The IUCN Red List of Threatened Species is a project that determines priority for conservation depending on criteria such as population trend, geographic distribution and abundance of each evaluated species. An additional way of determining conservation priorities is by taking into consideration phylogenetic approaches. For example, a system that reflects the value of taxonomic diversity can be achieved by setting priorities such that the subset of taxa that is protected has maximum underlying feature diversity, predicted by the phylogenetic relationships among taxa [7]. This technique is known as phylogenetic diversity (PD) and has already been used [8–11]. Another method that could be implemented in determining conservation priorities is measuring species relative contributions to phylogenetic diversity by calculating their evolutionary distinctiveness (ED) [12]. Some species are more distinct than others in terms of representing a larger amount of unique evolutionary history [11]. As an example, species like the tuatara (*Sphenodon*) have no close living relatives and have millions of years of independent evolution, resulting in a unique phylogenetic branch, and thus have higher ED than any other reptile taxon. However, these two methods do not take into consideration distribution and abundance, being therefore incomplete ways of assessing conservation priorities. Some studies suggest that the future of conservation will include the combination of quantitative measures of taxonomic and phylogenetic patterns with more conventional ecological considerations on threats [10, 12–15].

In recent years, major global populations declines of all kinds of animals and plants have been detected. These declines are manifested in higher species extinction rates, which exceed normal background rates by two or three orders of magnitude [16]. Reptiles are no exception and declines in reptile populations are troubling, not only for their ecological relevance to many ecosystems, but also because they indicate a general decay of environmental health as well as declines of other species [17]. Moreover, being a group with a poor knowledge on its conservation status, it is harder to determine which species should be protected. A double task of evaluating species lacking extinction risk assessment and the proposal of conservation actions for threatened species is still to be achieved. Efficient conservation of reptile species requires an expansion of knowledge on species distribution, life history, ecology and taxonomy, focusing on those under-assessed taxa inhabiting under-sampled regions with high levels of reptile species richness, such as the arid areas of North Africa and Arabia [1, 18].

**Fig 1:**
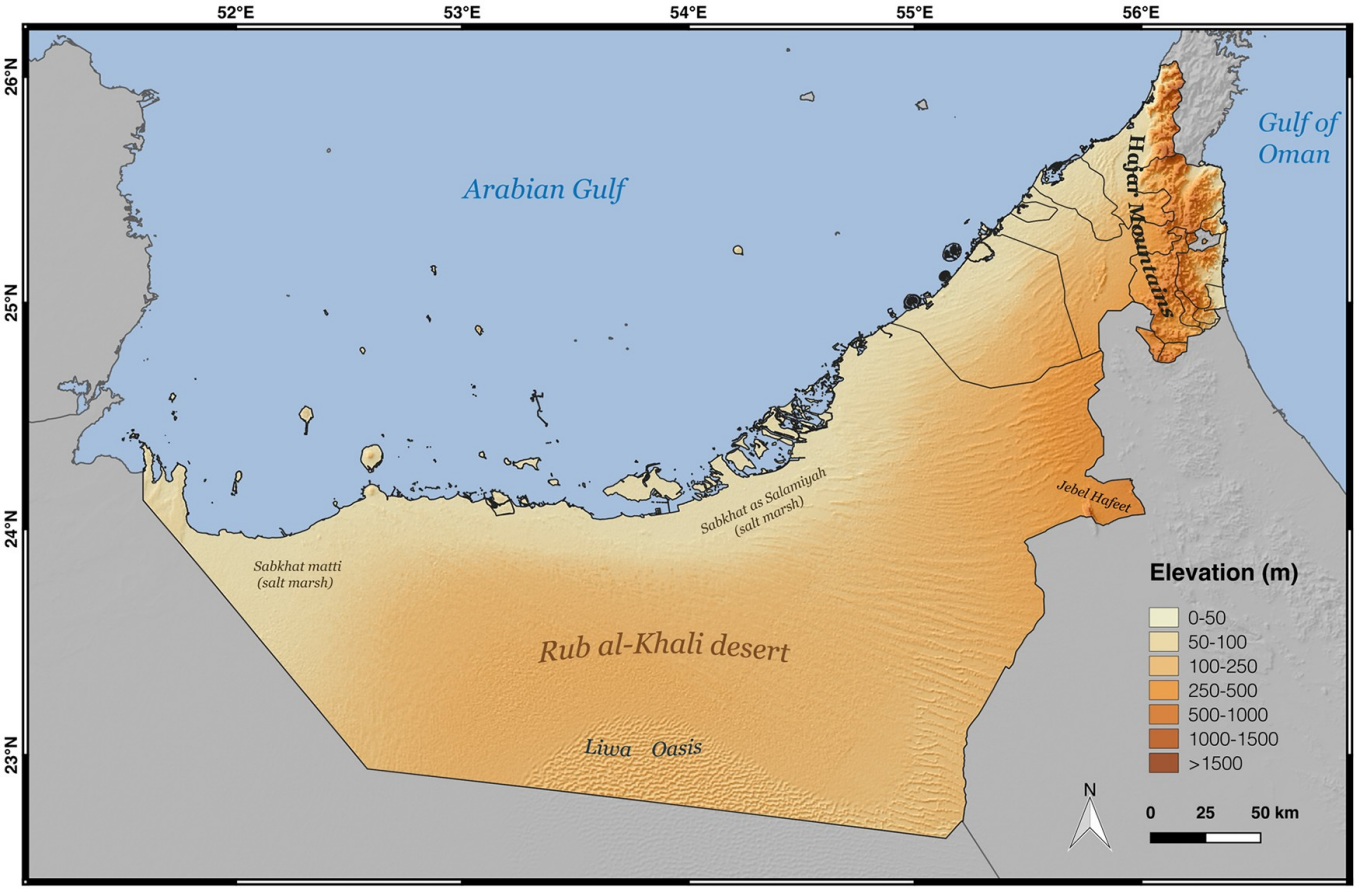
Physical map of the UAE. Map of the UAE showing topographical relief and names of relevant toponyms. Credits: OpenStreetMap contributors, SRTM.

The United Arab Emirates (UAE) is a country situated in the eastern Arabian Peninsula, bordering Oman and Saudi Arabia (Fig 1). Currently, 60 species of terrestrial reptiles are known to occur within the UAE [3, 19]. With an annual mean temperature of 28°C and an annual mean precipitation of 93 mm, the UAE is considered to have a hot desert climate according the Köpper-Geiger climate classification [20]. As over 90% of the country’s surface is sand or rocky desert (S1 Fig), it is an excellent area to study reptiles adapted to arid conditions. Even though deserts cover almost all of the UAE, the country landscape is not homogeneous, as there are a wide variety of desert types. Coastal sands, salt flats, deltas or evaporite deserts extend along the coastal regions. Inland, vast areas are occupied by sand dunes, including the Rub al-Khali (also known as the Empty Quarter), which is the largest contiguous sand desert in the world, and rocky deserts in the northern UAE [21] (Fig 1). Apart from the deserts, the UAE has a variety of spatially restricted habitats supporting distinct species communities with different requirements. Two areas of high reptile diversity are the Hajar Mountains in the northeast and Jebel Hafeet in the east [19]. With an elevation of 1,934 m, Jebel Jais in the Hajar Mountains is the highest point in the UAE. Although the Hajar Mountains form a continuous arc of over 650 km that runs parallel to the coast of the Gulf of Oman, the UAE only contains a very small part of this impressive massif, considered one of the top hotspots of endemicity in Arabia [1]. Jebel Hafeet is located on the UAE’s eastern border with Oman, and is about 26 km long, rising to 1,249 m in its highest peak. It is formed by Cambrian continental shelf sediments and has a system of caves, providing shelter and suitable habitats for a wide range of species [22]. Finally, oases, such as the Liwa Oasis located in the northern edge of the Rub al-Khali desert and stretching for 100 km from east to west, or the seven oases of the city of Al Ain located at the foot of the Jebel Hafeet, are very important places for species less adapted to extremely arid deserts conditions and which usually support richer assemblages of species than the surrounding deserts. Al Ain is one of the fastest changing city of the Arabian Peninsula and has transformed from a desert oasis to a highly developed city in less than 30 years [23], diminishing the accessibility to the oasis for wild species. In recent decades, and due to the oil industry, most major cities in the UAE have gone through an exponential growth, changing the landscape of the coast with massive land reclamation projects [23]. These infrastructure projects change the dynamics of the coastal environments and alter the distribution of reptile species, consequently increasing the importance of updating the knowledge on reptile taxonomy and their current distributions in the UAE.

The UAE is formed by seven emirates (Abu Dhabi, Dubai, Sharjah, Ajman, Umm Al Quwain, Ras Al Khaimah and Fujairah) and covers approximately 83,600 km^2^ of land. The smallest emirate is Ajman with only 0.3% of the country’s area and the largest emirate is Abu Dhabi with 83.5% of the total area, and where the capital city of the country (Abu Dhabi) is located (S2 Fig). The UAE includes 42 protected areas with a total surface of 13,850 km^2^, representing 16.6% of the territory’s terrestrial area.

This study aims to determine the spatial distribution of all 60 species of UAE terrestrial reptiles using an unparalleled database including 5,535 records and a range of GIS-based spatial tools. It also aims to assess the quality of our sampling, the ecological preferences of each species, to infer the spatial patterns of species richness and the effectiveness of the protected areas in protecting the country’s 57 native reptile species (three species are introduced). This work represents an integrative approach using not only ecological and spatial data but also a phylogenetic dataset including 146 species for 15 genes newly assembled this study to determine the phylogenetic diversity (PD) and evolutionary distinctiveness (ED) of the UAE native terrestrial reptiles.

## Materials and methods

### Ethics statement

No in vivo experiments were performed. This research is not Institutional. As a result of the characteristics of this study and the total control and compliance with the laws, regulations and procedures of this kind of biodiversity studies in the United Arab Emirates, it did not need the approval by an Institutional Animal Care and Use Committee or ethics committee.

### Database

The database used in the present study includes a total of 5,535 locality records of UAE terrestrial reptiles (Sauropsida, Squamata) obtained from various sources: 31.6% of the data (1,750 observations) were obtained from observations gathered by the Environmental Agency from Abu Dhabi. Another one-third of the data used, 31.2% (1,727 observations), were published by Gardner (2013) [19], and resulted from his personal fieldwork, bibliographic data, museum vouchers and observations collected from many herpetologists. The highest numbers of records, totaling 33.9% of the data (1,879 observations), were compiled by Johannes Els from the Breeding Centre for Endangered Arabian Wildlife, Sharjah. Finally, the remaining 3.2% of the data (177 records) resulted from fieldwork carried out by several authors of the present study between 2005 and 2018. Prior to analyzing the data, all datasets were compared and duplicate records were removed. The final dataset included observations of all the 60 species of UAE terrestrial reptiles belonging to 14 different families. Marine snakes and turtles were excluded from the study.

### IUCN Red List conservation categories

The IUCN conservation categories at a global level of each UAE species have been obtained from the IUCN Red List of Threatened Species website (http://www.iucnredlist.org). The IUCN conservation categories at the national level of each of the 60 UAE reptile species and their native or introduced status were obtained from the still unpublished conclusions of the 2018 UAE Red List of Reptiles and Amphibians, sponsored by the UAE Ministry of Climate Change and Environment. One of the advantages of using the IUCN categories of this recent national assessment is that it is up-to-date with the recent taxonomic changes that have affected the UAE reptile fauna [24, 25]. These categories substitute those published in the IUCN website or in the regional assessment by Cox et al. (2012) [26].

### Spatial data

The spatial layers with the UAE political borders, its seven emirates and the coastline were extracted from the open data initiative OpenStreetMap (https://www.openstreetmap.org). Climate data were obtained from WorldClim v.2.0 (http://www.worldclim.org) and included 19 bioclimatic variables (BIO1–BIO19) at a spatial resolution of 30 arc-seconds (~1 km). Bioclimatic variables were derived from the different values of monthly temperature and precipitation between 1970 and 2000 [27]. The digital elevation model (DEM), used for the elevation data, was downloaded from the reverb tool from NASA (http://reverb.echo.nasa.gov). The elevation data originated from the Shuttle Radar Topography Mission at a spatial resolution of 1 arc-second (~30 m). A layer of land covers was downloaded from the International Steering Committee for Global Mapping (ISCGM) website (https://www.iscgm.org/gmd) at a resolution of 15 arc-seconds (~500 m). From the 18 land cover types compiled by the ISCGM, only nine have been included since the other nine are not present in the study area. The land covers used were: Tree Open, Cropland, Cropland / Other vegetation mosaic, Shrub, Herbaceous, Sparse Vegetation, Bare Areas Gravel Rock, Bare Areas Sand, and Urban. The layer used to get all the protected areas from the UAE was extracted from the World Database on Protected Areas from the IUCN webpage (https://protectedplanet.net) and it was later refined by data compiled by the Environmental Agency from Abu Dhabi, the Environment and Protected Areas Authority, Sharjah and the Natural Resource Conservation Section, Environment Department, Dubai Municipality. All data were processed with geographic coordinates using the WGS84 coordinate system except when the projection needed to be in meters and not decimal degrees. In those cases, an equidistant projection centered in the UAE was created.

### Evaluation of the sampling effort

The evaluation of the sampling effort was carried out by analyzing the distribution of locality records following three different methodologies: i) assessing the spatial distribution of the samples in a 10 arc-minutes grid (~18 km) covering the whole country; ii) analyzing the distribution of all the samples in the UAE’s climatic space determined by the annual mean temperature (BIO1) and annual precipitation (BIO12) climatic variables, and iii) checking the distribution of all the samples in the PCA of the UAE’s climatic space (see below) and within each of the different climatic clusters.

### Distribution maps

Distribution maps for the 60 species of UAE terrestrial reptiles were inferred using the same resolution as in Gardner (2013) [19] and Carranza et al. (2018) [1]: a grid of 10 arc-minutes of latitude and longitude (~18x18 km, 324 km^2^). This resolution was chosen to facilitate the use of Gardner’s data and, more importantly, to establish a baseline resolution to be able to join the different databases of Oman, the UAE and other Arabian Peninsula countries for future studies (work in progress). Also, the resolution is appropriate for the sampling effort (5,535 observations) and for the total area of the UAE (83,600 km^2^). The 10 arc-minute vector grid was created using the tool “vector grid” in QGIS v. 2.18.15 Las Palmas [28]. The grid comprises the whole of Arabia in order to be able to expand the study to nearby countries using the same grid. To obtain an individual vector grid with the distribution of each species the QGIS plugin “JoinSplit”was used. Finally, a distribution map for each of the 60 UAE species of terrestrial reptiles was created (see S1 Appendix) and distribution maps of species richness, venomous species richness and threatened species richness were also created (see Results).

### Ecological characterization of the species

The ecological preferences of the UAE reptile species were characterized using the frequency of occurrence in elevation and with climate and land cover variables. The distribution of the elevation frequency was obtained by overlaying the elevation layer at a resolution of 30 m (see above) with the species occurrence data and it was represented by a histogram of 100-meter bins. For each species, the climatic data were obtained following the same procedure at a resolution of 30 arc-seconds (~1 km). The species climatic preference was inferred within a two-dimensional climatic space of the UAE defined by the total annual precipitation (BIO12) and the mean annual temperature (BIO1). Both variables are ecologically very relevant for many vertebrates, particularly for ectotherms such as reptiles, and provide general and very useful information of the local climate. The land cover preferences were inferred for each species by calculating the distance of each observation to the nine different land cover types (see above), and the distribution was represented as a boxplot. In order to provide metric distances, the land cover was projected using a World Equidistant Cylindrical projection centered in the UAE. The analyses and graphic output were produced with R programming environment [29, 30].

### Principal component analysis (PCA) of the climatic space of the UAE and delimitation of the study area into climatic clusters

A PCA with the 19 climatic variables was carried out in order to assess the climatic space of the UAE. The PCA was performed using the function “prcomp” from the R package “stats”. The different climatic clusters were calculated so that they grouped 20% of the total explained variance in each component (PC1 and PC2). The clusters were calculated following Carranza et al. (2018) [1]. In order to represent the climatic space of each one of the 60 species and the UAE protected areas, the climatic data for each species and for the protected areas was calculated using the “Point Sampling tool” plugin in QGIS.

### Data for species distribution models

The species distribution models (SDMs) were computed to obtain the most likely area of distribution considering each species’ niche from the 5,535 occurrence records obtained for 60 species (see Materials and methods). Each observation was carefully checked for coordinate errors. Disparate localities outside the species’ range for some species that are easily moved by human activities were removed for the modeling. The records were projected to a custom cylindrical equidistant projection fixed at the centroid of the UAE and processed to remove pixel duplicates for each species at the spatial resolution of the analysis (1 km).

### Ecological variables

As our main focus was the distribution of each species, we considered independent ecological variables that described the available habitat and climate in the study region. Since some species in the dataset had a low number of presences data available, we limited the number of variables to four in order to automate the process of modeling to all species. We gathered a decade of Normalized Differential Vegetation Index (NDVI) maps from January 2005 to December 2015. The NDVI data is related to the green productivity of an area and offer a good description of the available habitat with a continuous value. We used the NASA EarthData service (https://earthdata.nasa.gov/) to download the 16-day period NDVI MOD13Q1 imagery with 250m spatial resolution. The NDVI time series was summarized to two components by means of an harmonic regression performed using the R packages “TSA” v1.01 [31], “zoo” v1.8.2 [32] and “rgdal” v1.3.3 [33]. This regression fits an equation where each coefficient describes a periodic variation in the time series [34]. The resulting components were upscaled to the resolution of the analysis (1 km). Climate data was obtained from Worldclim v.2.0 at 30 arc-second resolution (see above). As we limited the number of variables, we opted to include a single variable of temperature (annual mean temperature) and another one of precipitation (annual precipitation). Both datasets were transformed to the custom coordinate reference system for the analysis and clipped to the UAE area. Only pixels with the four variables were retained for further analyses.

### Species distribution models (SDMs)

The SDMs were inferred with the software Simapse [35], based on artificial neural networks. The models were automated for all species with five or more presence data points available without pixel duplicates (52 out of the 60 species). For the eight species with less than five records SDMs were not inferred and the distribution range was approximated using other methods (see below). The parameters values were estimated heuristically and used for all models. This included a five burn-in plus 2,000 iterations, a 0.01 learning rate and a single hidden layer with four neurons. A set of pseudo-absences (PA) was randomly created for each species. The number of PA was set to five times the number of presences available and equally distributed in two altitude classes (below and above 500 m) in order to obtain a good description of the available area. All models included 100 replicates with random subsampling retaining 25% of data to test and the remaining to model training. Model performance was measured by means of the area under a receiver operating characteristic curve (AUC). This value is between 0.5 (random model) to 1 (perfect discrimination) and values above 0.75 are generally considered good. The objective of the SDMs was to be as conservative as possible in the distribution area but with all presences well predicted. The lowest predicted value of all observations was used as a cutoff threshold to make a binary prediction. To eliminate areas with potential presence but not confirmed by observations, for each species we retained only the predicted areas contiguous to each presence record. This was automated by means of an R script with “rgdal” package.

### Gap analysis

A gap analysis was performed to calculate the proportion of the total distribution of each species that was included within a protected area [36]. The species distribution area was inferred using two different approaches: i) the Area of Occupancy (AOO) taking into account all the observed records at a resolution of 2x2 km (4 km^2^); and ii) the species potential distribution (SPD), calculated by the sum of all 4 km^2^ cells inside the potential distribution of each species inferred with the SDM (see above). We believe that the methodology used to define the potential distribution for each species in this study is more refined than the one suggested by the IUCN, which considers the potential distribution (or extent of occupancy) as the minimum convex polygon defined by all the records of each species [37]. The 4 km^2^ resolution has been chosen as suggested by the IUCN Standards and Petitions Subcommittee 2016 [38]. Eight out of the sixty species did not have the minimum of five records to infer their SPD using the modeling approach. In these cases, the SPD was calculated by the sum of all 4 km^2^ squares inside the minimum convex polygon of each species as suggested by the IUCN [37]. In order to be considered adequately protected we have used the 17%, following Aichi Biodiversity Targets [39], and the less restrictive 12% [36, 40–41] of the species’ total area within a protected area as conservation targets. The 2x2 km grid was created with the “vector grid” tool from QGIS. The following analyses and the representation of the percentage of the total area that falls within a protected area were carried out in R environment.

### Phylogenetic analyses

To infer the phylogenetic relationships, we assembled a dataset including i) all 60 species of terrestrial reptiles that occur in the UAE; ii) at least one representative of each one of the 58 families of Squamata; iii) several key species necessary to establish the 13 calibration points in the phylogenetic tree (S2 Appendix); and iv) one specimen of *Sphenodon punctatus* to root the tree. The final dataset included a total of 146 species: 145 species of Squamata and one outgroup. The gene composition of the dataset differed between samples because all the genes used were not always available but included up to 15 genes: six mitochondrial and nine nuclear loci. The concatenated alignment of all the genes (see below) had a total length of 14,454 base pairs (bp). The dataset included mainly sequences downloaded from GenBank [42] or produced *de novo* for the present study. GenBank accession numbers for all the sequences used in the present study are shown in S1 Table. The sequences downloaded from GenBank were previously checked with BLAST in order to verify that they belonged to the correct species and gene. In some cases, the name of some taxa needed to be changed as a result of the multiple taxonomic changes in Squamata occurred in recent years [24, 25].

A total of 1,105 sequences were aligned using two different protocols for the ribosomal and for the coding genes, respectively. For the mitochondrial *12S* and *16S* ribosomal genes we performed multiple sequence alignments using MUSCLE implemented in Geneious 6.1.8 [43]. Poorly aligned positions of the two non-transcribed *12S* and *16S* genes were eliminated with G-blocks [44] using low stringency options [45]. Coding mtDNA and nDNA sequences were cut in order to make sure that all the sequences started with the first codon position. Afterwards, the program TranslatorX [46] was used to align each gene separately. This program translates the sequences into amino acids, aligns the amino acids and back-translates the amino acids into nucleotides taking into account that the coding DNA evolves as codons, so that insertions/deletions in coding genes are incorporated in sets of three nucleotides [46]. Finally, all the coding genes and the ribosomal genes were concatenated in a single file using Geneious v.R9 [43]. Best-fit partitioning schemes and models of evolution for the dataset were inferred using the software PartitionFinder v.2.1.1 [47], with each gene separated as a partition.

The phylogenetic tree was inferred using Maximum Likelihood (ML) in RAxML v.7.4.2 [48] as implemented in raxmlGUI [49] with 100 tree searches, using the GTR+G model of sequence evolution and independent model parameters for all eight independent partitions obtained with PartitionFinder (see S2 Table). Reliability of the ML tree was assessed by bootstrap analysis [50] including 1,000 replicates. The definitive ML and calibration analyses were carried out with the results of PartitionFinder, which included eight independent partitions.

To date the origin and diversification of the different lineages of UAE terrestrial reptiles, a time calibrated tree was inferred using BEAST 1.8.4 [51]. Three individual runs of 10^8^ generations were carried out, sampling at intervals of 10^4^ generations. The following models and prior specifications were applied, otherwise by default: models of sequence evolution for the different partitions as selected by PartitionFinder (partition scheme by gene; see S2 Table); Speciation Yule tree prior; uncorrelated lognormal clock for mitochondrial genes and strict clock for nuclear ones; random starting tree; base substitution prior uniform (0,100); alpha prior uniform (0,10). Substitution and clock models were unlinked. Posterior trace plots and effective sample sizes (ESS) of the runs were monitored in Tracer v1.5 [52] to ensure convergence. The results of the individual runs were combined in LogCombiner discarding 10% of the samples as burn-in and the ultrametric tree was produced with TreeAnnotator (both provided with the BEAST package). In order to reduce computation time, the topology of the BEAST calibration analysis was fixed with the ML topology obtained with the same partition scheme. A total of 12 published calibration points scattered across the Squamata phylogenetic tree (so that they included both deep and shallow nodes) were used for calibration. The calibration points were based on either fossils or biogeographic evidence. The complete list of calibration points and priors used in the analysis can be found in S2 Appendix.

### Phylogenetic diversity

Phylogenetic diversity (PD) is a measure of biodiversity proposed by Faith (1992) [7], which incorporates the phylogenetic difference between species. It is defined and calculated as “the sum of lengths of all those branches that are members of the corresponding minimum spanning path”, understanding “branch” as a segment of a cladogram between two nodes and “the minimum spanning path” as the minimum distance between two nodes.

In this study, PD was calculated using two different approaches: Firstly, PD was calculated in all cells of the 10 arc-minutes grid for which there was observation data. Secondly, the same analysis was performed using the potential distribution of each species obtained with SDMs. To assess the PD of the terrestrial reptiles of the UAE, the ultrametric tree had to be pruned, so that it included only the 57 native species present in the study area (the three introduced species *Chalcides ocellatus*, *Hemidactylus flaviviridis* and *Indotyphlops braminus* were also pruned). PD was quantified as the minimum total branch length connecting all species present in each grid cell of 10 arc-minutes to the root of the phylogenetic tree [7]. We then assessed whether they had higher or lower PD than the expected by random with the same number of species. This was done by calculating the standardized effect size of PD (PDses) comparing the observed PD to the values expected from 10,000 random draws of an equal number of species. As explained in Abellán et al. (2013) [11], positive values of PDses indicate that PD is higher than expected by chance. PDses, PD and the randomization and the significance of PDses were calculated with the R package “picante” [53, 54]. Finally, we tested the correlation between the PD obtained for each cell of the 10 arc-minutes grid and the species richness.

### Identifying cells of high conservation value

We assessed to what extent each cell of the 10 arc-minutes grid had a high conservation value by calculating their evolutionary distinctiveness (ED). ED is a metric proposed by Isaac et al. (2007) [12] and is used to calculate how much evolutionary “information” would be lost if a species disappeared from a certain location. ED measures the distance along the evolutionary tree from one species to its nearest relative within the designated area of interest calculating the length of its terminal branch plus its weighted share of ancestral branches, giving greater value to those species whose genetic history is not shared with many other species [11, 55]. In the present study, we first identified the ED of each species of terrestrial reptiles in the UAE with the R package “picante” [54] and following the metric proposed by Isaac et al. (2007) [12]. Once the ED for each species was calculated, the value of ED for each grid cell was assigned as the mean value of all the species present in that cell. Like in the analyses of PD, for the ED analyses we only included the 57 native taxa and the ED of each cell was calculated twice: i) using the AOO of each species; and ii) using the SPD. In both cases the resolution was of 10 arc-minutes using the grid created for mapping the species distributions (see above).

## Results

### Evaluation of the sampling effort

**Fig 2:**
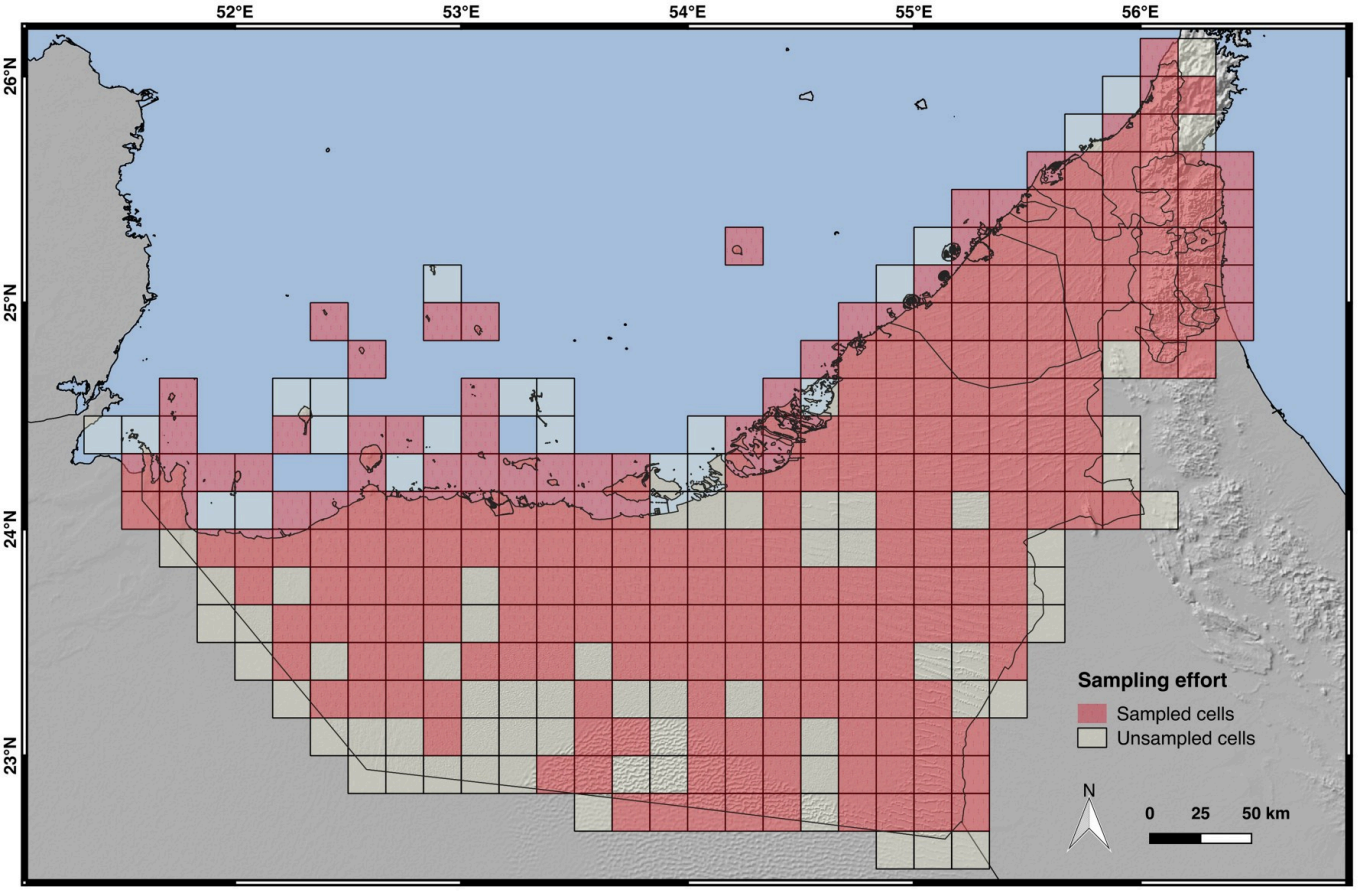
Map of the sampling effort. Grids of 10 arc-minutes (~18x18 km) with observations (red cells). Empty grids can be either no observations or no sampling. Credits: OpenStreetMap contributors, SRTM.

In total, 5,535 records including all 60 species of UAE terrestrial reptiles have been analyzed (See S1 Appendix; S3 Fig; S3–S5 Tables). The spatial data for the UAE terrestrial reptiles is available from S3 Appendix. Our results showed that 240 (75%) of all the 320 grid cells of 10 arc-minutes were sampled for this study (Fig 2). Nearly the entire country was sampled; the unsampled grid cells were mostly situated along the country’s perimeter where they comprised less country area than other grid cells. Other unsampled regions comprised some cells in the desert area of Abu Dhabi and in the Liwa oasis, in the southwest of the country. However, since both unsampled areas have contiguous cells sampled, we believe that the sampling effort for this study has been remarkably good. The sampled areas cover a vast area of the country, and the Hajar Mountains (the climatically most distinct area in the UAE) were completely sampled.

**Fig 3:**
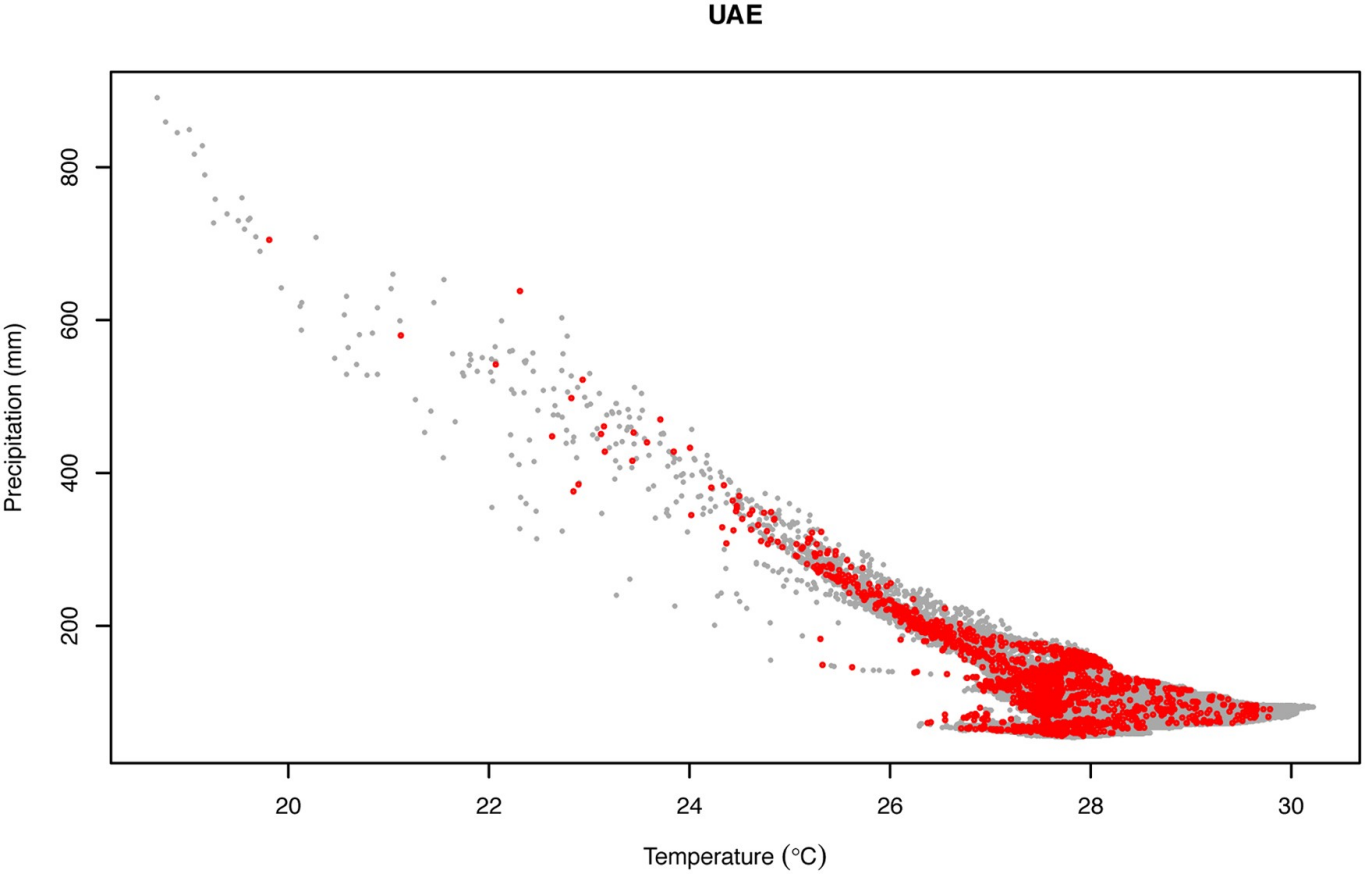
Two-dimensional climatic space of the UAE (grey dots) defined by the annual mean temperature (BIO1) and annual mean precipitation (BIO12). Red dots represent the distribution of the 5,535 occurrence records of all 60 species of terrestrial reptiles of the UAE.

**Fig 4:**
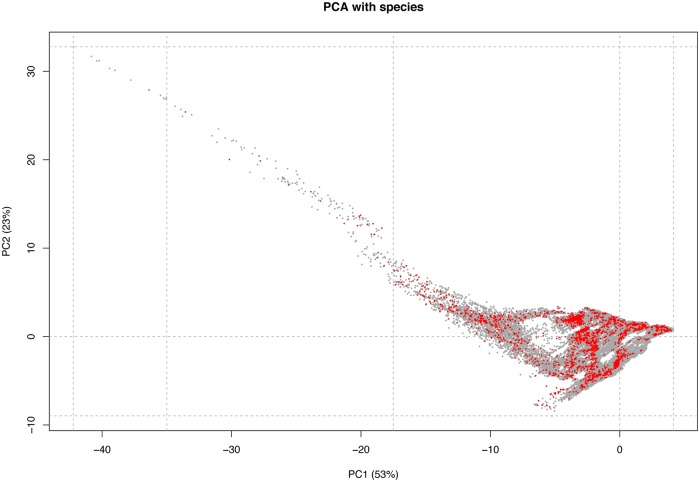
Principal component analysis (PCA) of the climatic space of the UAE (grey dots) using 19 bioclimatic variables from WORLDCLIM. Red dots represent the distribution of the 5,535 occurrence records of all 60 species of terrestrial reptiles of the UAE in this climatic space. Dashed lines delimit climatic clusters that group 20% of the explained variance of PC1 and PC2.

**Fig 5:**
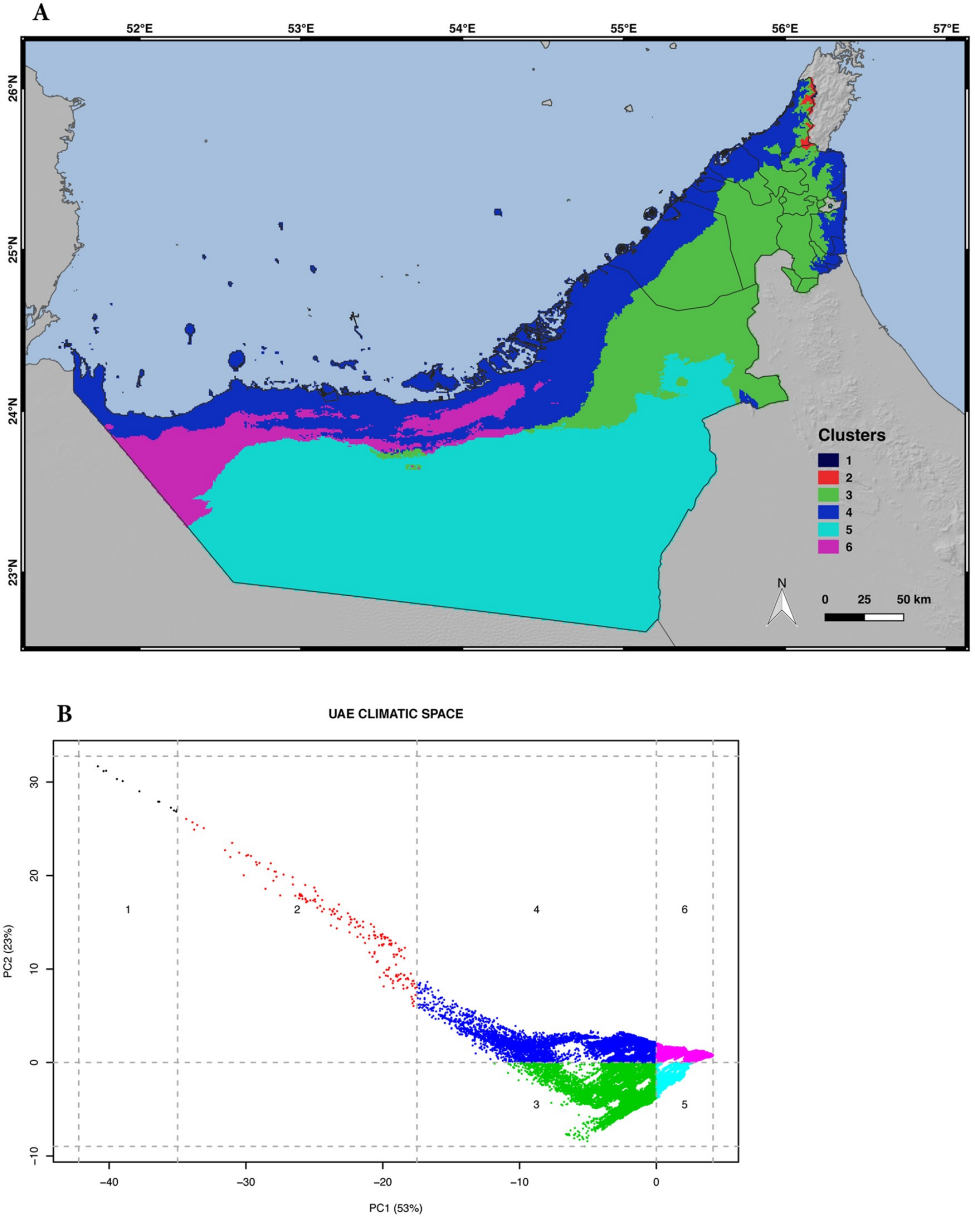
Climatic variability of the UAE. A) Map showing the distribution and extension of the six UAE climatic clusters; B) Principal component analysis (PCA) of the climatic space of the UAE (grey dots) using 19 bioclimatic variables from BIOCLIM with the climatic clusters numbered from one to six and colored with the same colors as in the map. Credits: OpenStreetMap contributors, SRTM.

As shown in Fig 3, the distribution of all the observations in the two-dimensional climatic space of the UAE indicates that samples are distributed along the whole climatic space defined by the annual mean precipitation and annual mean temperature. The maximum number of observations can be found between 26°C to 28°C of annual mean temperature and values below 200 mm of annual precipitation, concordant with the dominant climate in the UAE. The UAE areas with the lowest values of temperature and highest values of annual precipitation are also the ones with fewer observations, being again the regions with less available area (the Hajar Mountains). Finally, the distribution of all the observations across the multidimensional climatic space of the UAE defined by the PCA (S6 Table) shows that observations are well distributed across the climatic space (Fig 4). However, when the area was divided into clusters grouping 20% of the explained climatic variance by PC1 and PC2, not all six resulting clusters included observations and the distribution of the samples across the different clusters was also not proportional to the cluster size (Fig 5A and 5B; Table 1). Clusters 3, 4 and 5 cover large areas across the UAE, including all the coastal regions (covered by cluster 4) and the inland (cluster 3 in the northeast and cluster 5 in the southwest entering into the Rub al-Khali). Other clusters, such as 1 and 2, cover very specific and reduced areas including the highest elevations of the Hajar Mountains. Such small areas can explain 20% of the variance of the PCA because they are in the Hajar Mountains, a region with very different temperature and precipitation regimes from the rest of the country. The high elevation of those mountains compared to the surrounding desert areas offer a wide variety of climates and, since the UAE shares these mountains with Oman, not all the variance and area is taken into account (hence the small areas).

**Table 1:** List of all six climatic clusters that group 20% of the explained variance of PC1 and PC2 of the principal component analysis (PCA). The table shows the cluster denomination; the number of species present in each cluster, the number of observations detected in each cluster, the sampled percentage of each cluster and the total area of each cluster.

Cluster	Number of species by cluster	Number of observations by cluster	% sampled	Cluster area (km^2^)
1	0	0	0.00%	13.00
2	9	38	21.10%	166.70
3	56	2,382	13.50%	16,369.70
4	59	2,397	10.90%	20,329.70
5	29	665	1.40%	43,411.90
6	20	209	3.00%	6,512.30

Clusters can be divided into three categories depending on their area: small (<1,000 km^2^), medium (1,000–20,000 km^2^) and large (>20,000 km^2^). Each category includes two clusters, all of which have been sampled except cluster 1, the most reduced cluster within the smallest category. Although the sampling effort was not equivalent to cluster size, the range of percentage sampled between medium and large categories was similar: medium clusters ranged between 3.0–13.5% of their area sampled while large clusters had a range between 1.4–10.9% of their area sampled.

### Diversity of the taxonomic groups

The terrestrial reptiles that can be found in the UAE are represented by seven taxonomic groups. The most diverse group, with 36% of the species, are the geckos (Gekkota; comprising the families Gekkonidae, Phyllodactylidae and Sphaerodactylidae). It is a highly relevant group as it includes the only endemic vertebrate species of the UAE, *Asaccus caudivolvulus* [24]. Geckos are followed by snakes (Serpentes; comprising the families Boidae, Colubridae, Lamprophiidae, Leptotyphlopidae, Typhlopidae and Viperidae), which comprise 23% of the UAE terrestrial reptile species; lacertid lizards (Lacertidae) 17%; agamids (Agamidae) 10%; skinks (Scincidae) 10%; and finally the least diverse groups are the varanids (Varanidae) and amphisbaenids (Trogonophidae), each representing 2% of the terrestrial reptile diversity of the UAE (S4A Fig). Although the relative proportions among taxonomic groups are very similar to our observations during fieldwork, there are some groups that differed. Snakes and skinks represented 18% and 6% of all the observations, respectively. On the other hand, agamids represented 16% of the observations (S4B Fig).

### Evaluating the species distribution models (SDMs)

The terrestrial reptile fauna of the UAE is widely distributed across the country. Out of the 60 species, we were able to infer SDMs for 52 species, which had five or more presence data available without pixel duplicates. The average AUC for species models was 0.934±0.037SD (minimum AUC = 0.850), indicating good model performance. The potential distribution of the remaining eight species was calculated using the minimum convex polygon between the records at a resolution of 2x2 km (4 km^2^). All the SDMs maps for each species and the potential distribution maps for those species with less than five records can be found in the S1 Appendix.

### Distribution of species richness

**Fig 6:**
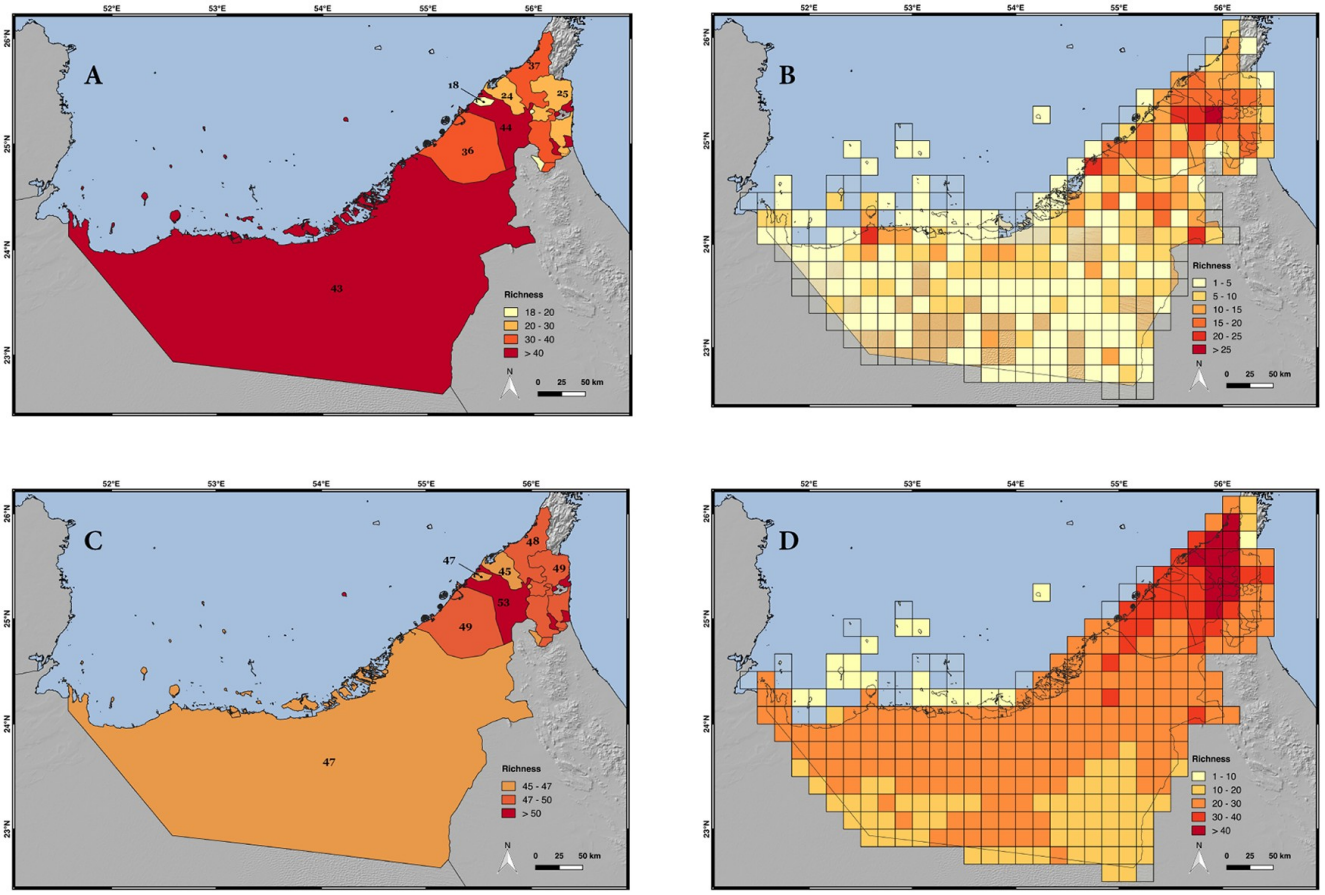
Species richness map of the UAE terrestrial reptiles. A) Species richness by emirate inferred with the occurrence point data; B) Species richness by 10 arc-minutes grid cells inferred with the occurrence point data; C) Species richness by emirate inferred with the species distribution models; D) Species richness by 10 arc-minutes grid cells inferred with the species distribution models. Only the 57 native species have been included in the analyses. Credits: OpenStreetMap contributors, SRTM.

The distribution of species richness of the 57 native reptiles was calculated in four different ways: i) by emirate; ii) by grid cell (at a 10 arc-minute resolution) considering the species occurrence point data; and iii) by grid cell (at a 10 arc-minute resolution) considering the SDMs (Fig 6A–6D). When only the occurrence point data of the species was considered, Sharjah (44 species) and Abu Dhabi (43 species) are the emirates with the highest diversity. They are followed by Ras Al-Khaimah (37 species) and Dubai (36 species), and the remaining emirates all have less than 30 species (Fujairah, 25 species; Umm Al-Quwain, 24 species; Ajman, 18 species) (Fig 6A). When SDMs were considered, species richness increased in all the emirates and the patterns of distribution of species richness changed. Sharjah is still the emirate with the highest number of species (53 species) but it is followed by Dubai and Fujairah (both with 49 species), Ras Al-Khaimah (48 species), Abu Dhabi and Ajman (both with 47 species), and Umm Al-Quwain (45 species) (Fig 6C). With the SDM approach, each emirate contains at least 75% of the terrestrial reptile fauna of the UAE. With the finer resolution of species richness by grid cell, the distribution of richness changed drastically. The highest richness is concentrated in the northeast of the country (27 and 48 species with the occurrence point data and SDM approaches, respectively), in a transitional area from sandy desert to the mountainous terrain of the Hajar Mountains. There is also one grid cell with a high number of species in the southwest coast of the UAE (not recovered with the SDM approach), in the Jebel Hafeet and in the coastal area surrounding the most populated cities of the UAE (Fig 6B and 6D).

The species richness of all the medically important venomous species of the UAE was also taken into consideration, since they are relevant for human health and conservation. As above, the UAE medically important venomous species were also analyzed by emirate and by grid cell, in both cases taking into account the point occurrence data and the SDMs. The four species of medically important venomous reptiles of the UAE (the snakes *Cerastes gasperettii gasperettii*, *Echis carinatus sochureki*, *Echis omanensis* and *Pseudocerastes persicus*) were found in Sharjah, Fujairah and Ras Al-Khaimah but the SDM analysis revealed that these four snake species can potentially be found in almost every emirate except in Umm Al-Quwain, where the habitat is suitable for only three of the four venomous snakes (S5A–S5C Fig). The analysis by grid cell showed that species richness of the four medically important venomous snakes reaches its highest values in the same transitional area where the highest species richness is found. The four species’ distributions converge in the surroundings of the Hajar Mountains. The area with the lowest number of medically important venomous snakes is located in the southern part of the country, in the Rub al-Khali desert (S5B–S5D Fig).

### Evaluation of the IUCN Red List conservation categories

**Fig 7:**
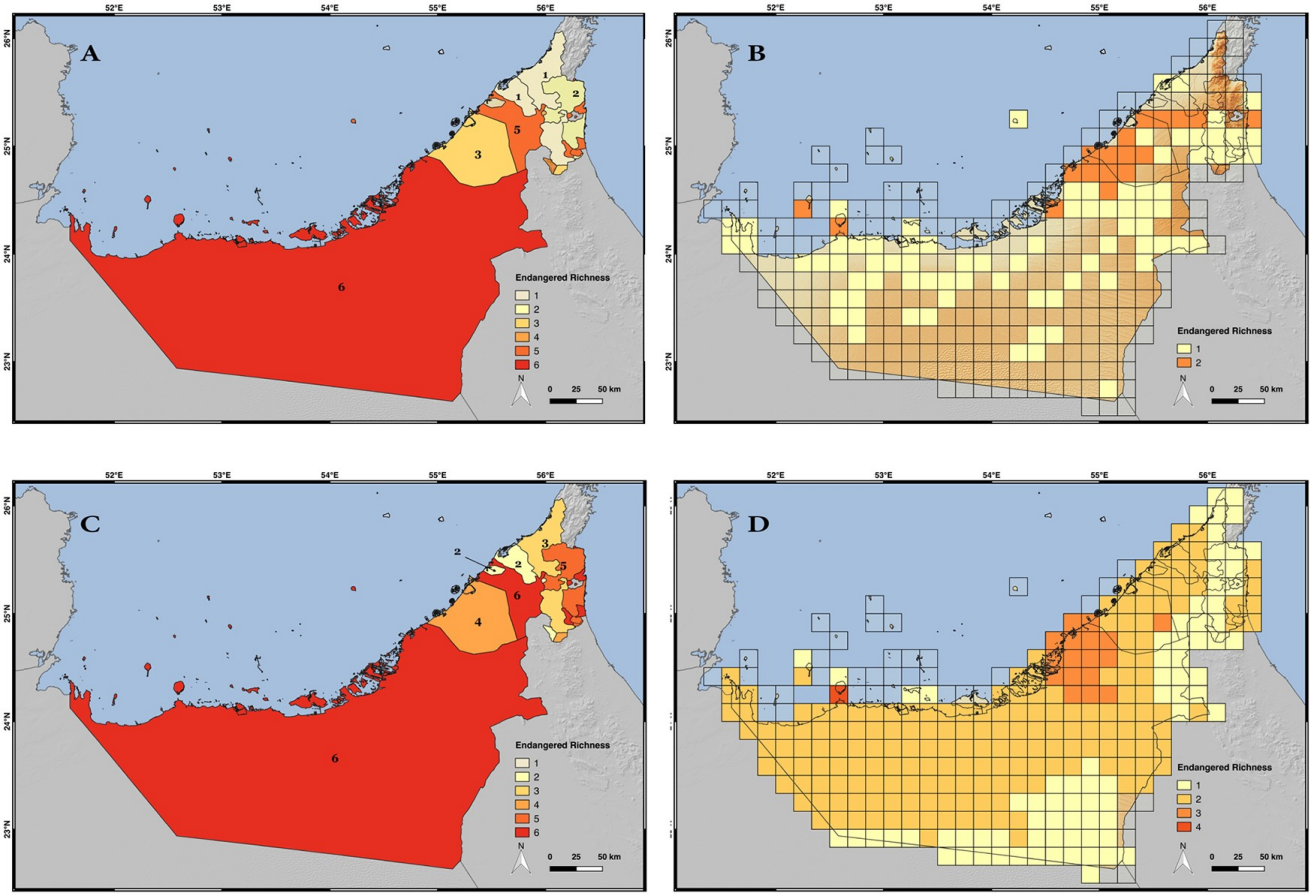
Threatened species richness map of the UAE terrestrial reptiles. A) Threatened species richness by emirate inferred with the occurrence point data; B) Threatened species richness by 10 arc-minutes grid cells inferred with the occurrence point data; C) Threatened species richness by emirate inferred with the species distribution models; D) Threatened species richness by 10 arc-minutes grid cells inferred with the species distribution models. Credits: OpenStreetMap contributors, SRTM.

According to the still unpublished conclusions of the 2018 UAE Red List of Reptiles and Amphibians, there are nine threatened species of terrestrial reptiles in the country (S3 Table; IUCN, unpublished data). Three of them (5% of all UAE species) are listed as Critically Endangered (CR) and include the only endemic vertebrate of the UAE (*Asaccus caudivolvulus*) and two other species of geckos (*Pristurus carteri* and *Teratoscincus keyserlingii*). The other six species (10%) are listed as Vulnerable (VU) and consist of two subspecies of the agamid *Uromastyx aegyptia* (*Uromastyx aegyptia leptieni* and *Uromastyx aegyptia microlepis*), two species of geckos (*Asaccus margaritae* and *Hemidactylus persicus*), one species of lacertid (*Acanthodactylus blanfordii*), and one species of snake (*Platyceps ventromaculatus*). One species (1.6%) is listed as Near Threatened (NT), four species (6.6%) are listed as Data Deficient (DD), three species (5%) are listed as Not Applicable (NA) since they have been introduced in the country, and the remaining taxa, 43 species (71.6%), are listed as Least Concern (LC). When the IUCN categories are analyzed by emirate using the point data it can be seen that with the only exception of Ajman, all the emirates have at least one threatened species within their territory (Fig 7A). The emirate with the highest number of threatened species is Abu Dhabi (six species) followed by Sharjah (five species) and Dubai (three species) (Fig 7A). When the SDMs are applied, the number of threatened species in each emirate tends to increase. Sharjah increases its threatened species from five to six becoming, together with Abu Dhabi, the emirates with the highest number of threatened species (Fig 7C).

When the more refined resolution by grid cell is considered, the area with the highest number of threatened species corresponds to the coast, surrounding and inside the coastal cities of Dubai, Abu Dhabi and Sharjah, the center of the Sharjah emirate and the southwest coast and two islands (Fig 7B–7D). Data show that the threatened species do overlap in their distribution area. Out of the nine threatened species the maximum value of richness in a single cell of the 10 arc-minutes grid is of two species in the occurrence point data (AOO approach) and four species when the SDM approach is considered.

### Evaluation of the protected areas

There are 42 protected areas in the UAE, covering 16.6% of the terrestrial area of the country. The emirates with the highest number of protected areas are Abu Dhabi and Sharjah both with 17, followed by Dubai with eight. Assessing the percentage of area covered, Dubai has the largest extent of territory protected (31.4%) and the rest of emirates range between 9–20% except Umm Al-Quwain, which does not have any protected areas (S7 Table).

**Fig 8:**
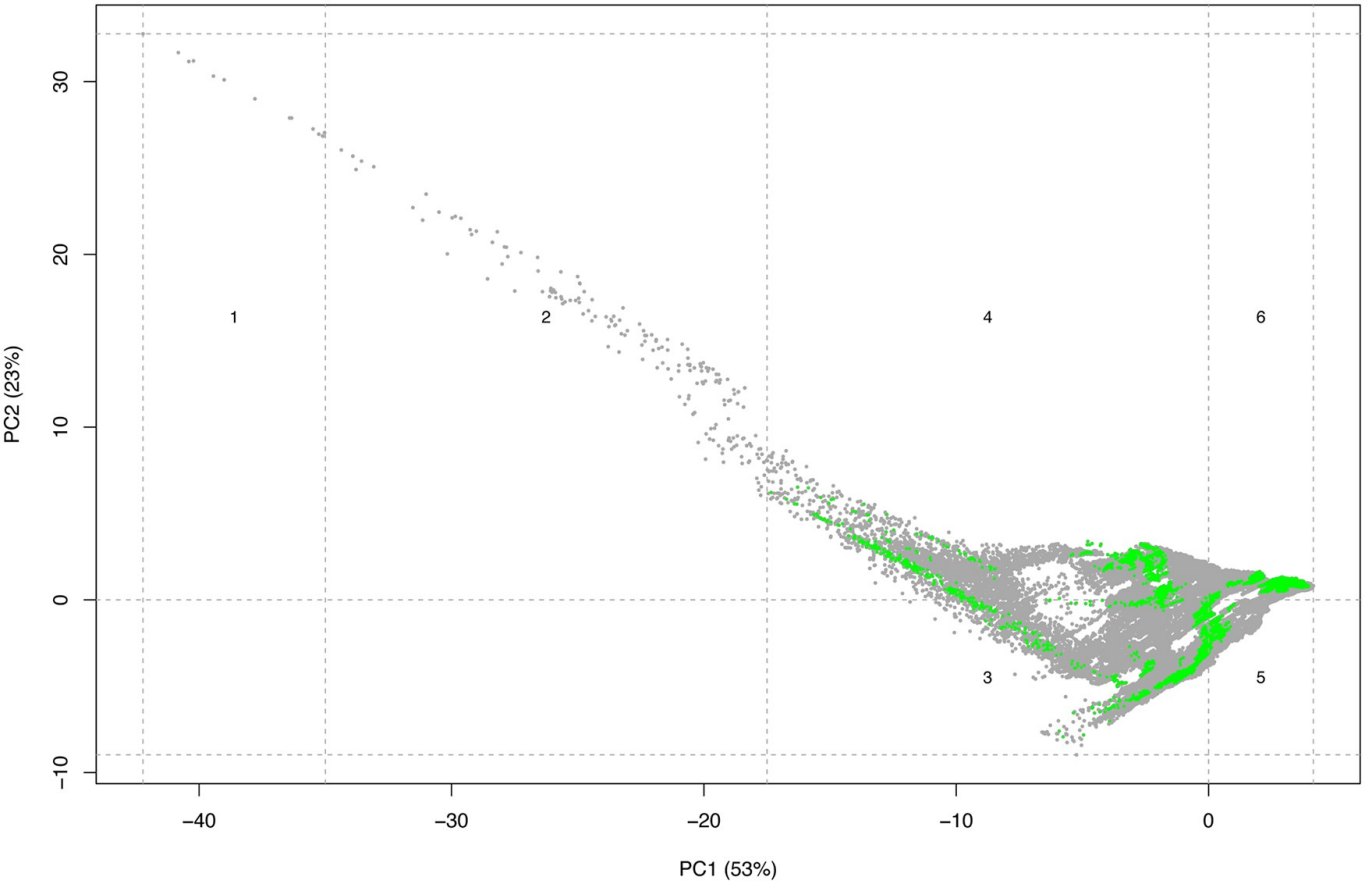
Principal component analysis (PCA) of the climatic space of the UAE (grey dots) using 19 bioclimatic variables from BIOCLIM. Dashed lines delimit climatic clusters that group 20% of the explained variance of PC1 and PC2. Green dots represent the climatic space occupied by the protected areas.

**Fig 9:**
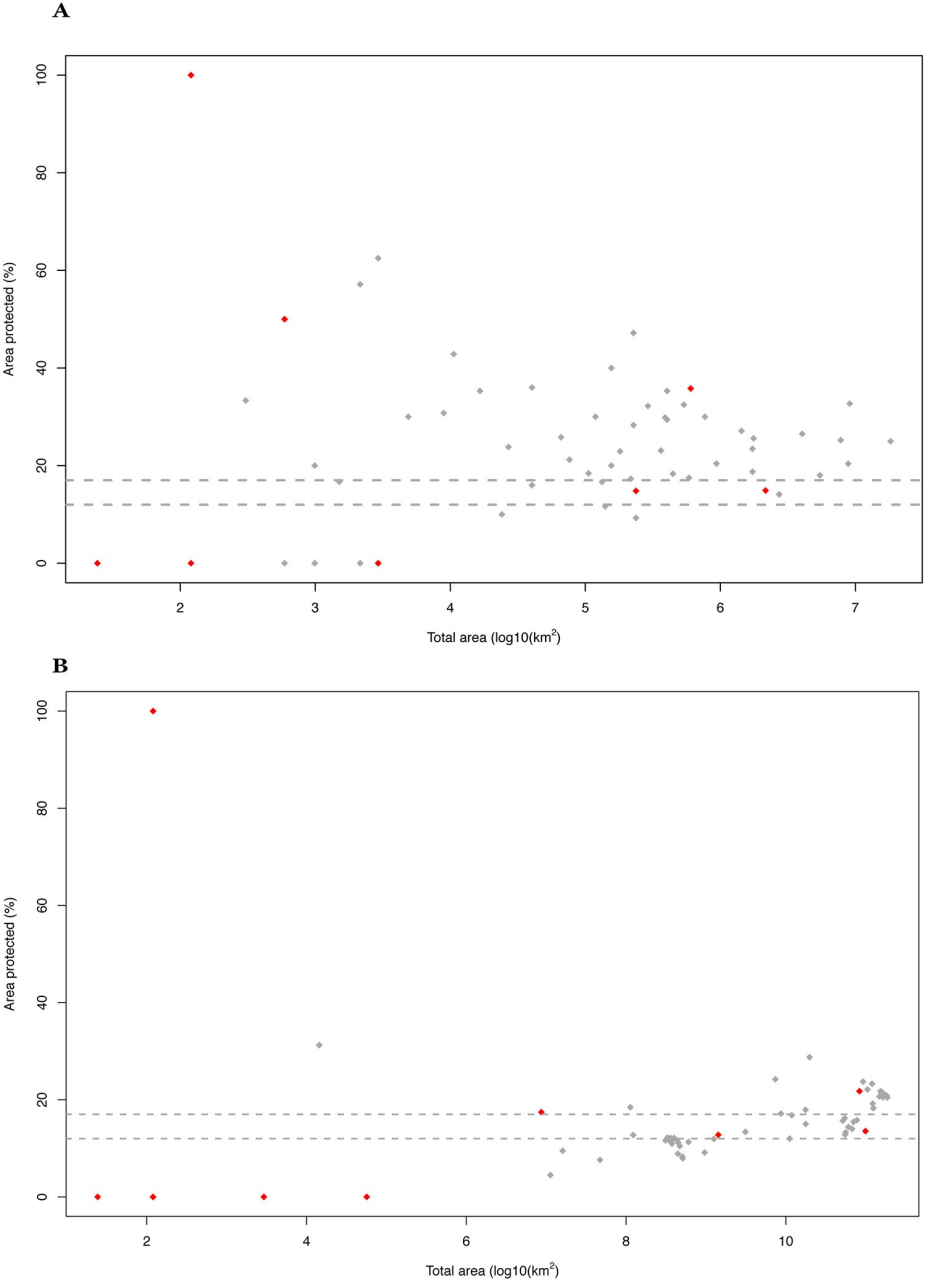
Percentage of the species’ distribution area included within a protected area. Dashed lines indicate the Aichi biodiversity conservation targets of 17% and 12%. A) Results of the analysis using the area of occupancy (AOO) taking into account all the observed records at a resolution of 2x2 km (4 km^2^); B) results of the analysis using the species potential distribution (SPD), calculated by the sum of all 4 km^2^ cells inside the potential distribution of each species inferred with species distribution models (SDMs). For the species for which it was not possible to infer SDMs (see Results), the area used for the analysis was that comprised within the minimum convex polygon of their occurrence point data. Red dots indicate the threatened taxa. Only the 57 native species have been included in the analyses.

As can be seen in Fig 8, the protected areas are widely distributed across the climatic space of the UAE represented by the PCA, and are present in four out of the six climatic clusters that divide the climatic space. The clusters without protected areas (clusters 1 and 2) are the two clusters with the least area (both clusters together occupy 0.2% of the UAE) and are located in the Hajar Mountains. The detailed results of the gap analysis using two different approaches (AOO and SPD) are given in S8 and S9 Tables, respectively. A graphical representation of the results of the gap analysis including the 57 native species is shown in Fig 9. Taking into account the native species only, the results show that 14 species (24,5%; AOO approach) and 35 species (61.4%; SPD approach) have less than 17% of their area protected and therefore are below the more restrictive Aichi Biodiversity conservation target [39]. Regarding the less restrictive conservation target [36, 40], 10 species (17,5%; AOO approach) and 17 species (29,8%; SPD approach) are below the 12% threshold.

As for the threatened species of the UAE (nine species; three CR and six VU), only three (AOO and SPD approach) reach the 17% target and five (AOO and SPD approach) reach the less restrictive 12% target. For the three CR species, not one reaches either of the two conservation targets in the AOO approach and only one (*Teratoscincus keyserlingii)* reaches the 12% target in the SPD approach. It is of much concern that two out of the three CR species (*Asaccus caudivolvulus*, the only endemic vertebrate of the UAE and *Pristurus carteri*) and two VU species (*Asaccus margaritae* and *Platyceps ventromaculatus*), are not included in any protected area (S8 and S9 Tables). The UAE species of native terrestrial reptiles have an average of 25.8% (AOO approach) and 16% (SPD approach) of their distribution inside a protected area. Also, 89,4% (AOO approach) and 94,7% (SPD approach) of the native terrestrial reptiles of the UAE have at least some part of their distribution inside a protected area.

### Ecological analysis of the UAE terrestrial reptiles

The study of the ecological preferences of all 60 species of UAE terrestrial reptiles revealed different degrees of ecological specialization (S1 Appendix). Examples of generalist species are the snake *Psammophis schokari*, which can be found in both the easternmost and westernmost areas of the UAE and from sea level up to 1,000 m and lives near bare areas with sand, bare areas with gravel rocks or even in sparse vegetation; or the snake *Platyceps rhodorachis rhodorachis*, which is the species with the highest altitudinal range (from sea level up to 1,200 m) and has a wide distribution in the climatic space of the UAE (S1 Appendix). Species with preference for low elevations and high temperatures are widely distributed across the country. This can be easily explained by the homogeneous topography of the UAE, with almost 90% being inland or coastal deserts (S1C Fig) below 200 m in elevation (S1D Fig), and by the warm climate of the UAE, with most of the country having an annual mean temperature over 27°C (S1A Fig). Species with ecological preferences that differ from the ones explained above usually live in more restricted areas, limited to coastal zones or mountainous terrains in the area of the Hajar Mountains or in the Jebel Hafeet, in the environs of Al Ain city.

### Ecological analysis of reptiles by taxonomic groups

#### Agamids

Four genera of agamids are found in the UAE: *Phrynocephalus* (two species), *Pseudotrapelus* (one species), *Trapelus* (one species) and *Uromastyx* (two subspecies of one species). The genus *Phrynocephalus* inhabits the most arid areas of the UAE, being widely distributed in both inland and coastal deserts. The two subspecies of the species *Uromastyx aegyptia* [56] have an allopatric distribution and have different ecological preferences. Both subspecies can be found in bare areas with sand but *U*. *a*. *leptieni* is restricted to the northeast of the country, from sea level up to 400 m in elevation in areas where annual precipitation is high, while *U*. *a*. *microlepis* lives in the southwest of the country, can be observed from sea level up to 200 m, tolerates higher temperatures, and lives in more arid areas than *U*. *a*. *leptieni* (S1 Appendix). The only species of *Pseudotrapelus* from the UAE, *P*. *jensvindumi*, is a rock specialist distributed from sea level up to 1,000 m in elevation in the Hajar Mountains.

#### Geckos

Geckos are the most diverse group of terrestrial reptiles of the UAE, including 21 species from the following genera: *Bunopus* (one species), *Cyrtopodion* (one species), *Hemidactylus* (three species, *H*. *flaviviridis* being introduced), *Pseudoceramodactylus* (one species), *Stenodactylus* (three species), *Trachydactylus* (one species), *Asaccus* (four species), *Ptyodactylus* (two species), *Pristurus* (four species) and *Teratoscincus* (one species). The genera described above contain the number of current described species and one species in the process of being described (*Pristurus rupestris*—sp. 3). However, there might still be hidden cryptic diversity in some groups such as *Hemidactylus* and *Bunopus* in neighboring Iran and Oman awaiting to be discovered and described that might affect the taxonomy of the UAE species. As for the ecological preferences of the geckos at the generic level, *Bunopus*, *Hemidactylus*, *Pseudoceramodactylus* and *Stenodactylus* have preferences for arid desert areas and are widespread around the country (except in the mountainous areas). On the other hand, the genera *Trachydactylus*, *Asaccus*, *Ptyodactylus* and *Pristurus* (except *Pristurus minimus*) are found at higher elevations with more precipitation and less temperature and prefer a wide spectrum of habitats, from vegetated to bare areas with gravel rock.

#### Lacertids

The 10 species of Lacertids include three different genera: *Acanthodactylus* (six species), *Mesalina* (two species) and *Omanosaura* (two species). *Acanthodactylus* and *Mesalina* species are mainly limited to elevations below 400 m, in hot and dry deserts or areas with sparse vegetation. On the other hand, *Omanosaura* species are restricted to the vegetated and bare areas with gravel rocks of the Hajar Mountains, ranging between 300 m and 1,200 m in elevation, in colder and wetter areas than the other genera of lacertid lizards.

#### Skinks

There are six species of skinks including five genera: *Ablepharus*, *Chalcides*, *Heremites*, *Scincus* (two species) and *Trachylepis*. *Trachylepis tessellata* and *Ablepharus pannonicus* share their ecological preferences, inhabiting near bare areas with gravel rocks at high elevations (0–1,000 m and 500–800 m, respectively). *Chalcides ocellatus ocellatus* (introduced) and *Heremites septemtaeniatus* both have preferences for low elevations in dry areas with sparse vegetation. Finally, the two species of *Scincus* (*S*. *mitranus* and *S*. *scincus conirostris*) have different ecological preferences. The former species is much more abundant than the latter and it is mainly restricted to hot and dry deserts, at elevations below 400 m, while *S*. *s*. *conirostris* is less abundant but can be seen in a wider range of land covers, such as sparse vegetation, bare areas with gravel rocks or deserts.

#### Varanids and amphisbaenids

*Varanus g*. *griseus* is the largest lizard of the UAE (more than 1.2 m of total length) and inhabits most parts of the country, being more easily found in deserts below 300 m in elevation, in areas that range between 27–29°C of annual mean temperature, with values of total annual precipitation below 200 mm. This habitat is similar to the one exploited by *Diplometopon zarudnyi*, the only amphisbaenid of the UAE.

#### Snakes

The snakes include 13 species from nine genera and constitute the most ecologically heterogeneous group. Two of them are generalist (see above), some of them are adapted to live in sandy deserts, such as *Eryx jayakari*, some are only found in coastal areas, such as *Platyceps ventromaculatus* or *Indotyphlops braminus* (introduced), and others are restricted to the northeast of the country, mainly to the Hajar Mountains, where there have been observations from sea level up to 1,200 m in elevation for the species *Pseudocerastes persicus*, *Echis omanensis*, *Telescopus dhara dhara* or *Platyceps rhodorachis rhodorachis*. These four species live under colder and wetter conditions than most of the reptile species of the country and are associated with rocky environments.

### Phylogenetic analysis and time calibrated tree

The topology obtained with the ML analysis (S6 Fig) was generally consistent with previously published phylogenies [57, 58]. The main Squamata clades were recovered and all the species from each genus grouped forming monophyletic groups. The time-calibrated tree (S7 Fig) suggests that Squamata started diversifying around 215–233 million years ago (mya). The first UAE reptiles to branch off the general Squamata tree were the geckos, which according to our analyses appeared approximately between 169–182 mya. They were followed by the snakes (156–167 mya), skinks (116–133 mya), amphisbaenids and lacertids (107–120 mya), agamids (88–98 mya), and varanids (31–41 mya). The age of varanids seems underestimated, most probably as a result of not having any calibration points within Anguimorpha. Nevertheless, since *V*. *g*. *griseus* is the only UAE representative of Anguimorpha and all the ages that separate Anguimorpha from all the other UAE reptiles belonging to Gekkota, Scincoidea, Lacertoidea, Iguania and Serpentes seem correct according to published studies, the results of the analyses of Phylogenetic Diversity and Evolutionary Distinctiveness are unaffected.

### Evaluation of the phylogenetic diversity (PD) of the UAE terrestrial reptiles

**Fig 10:**
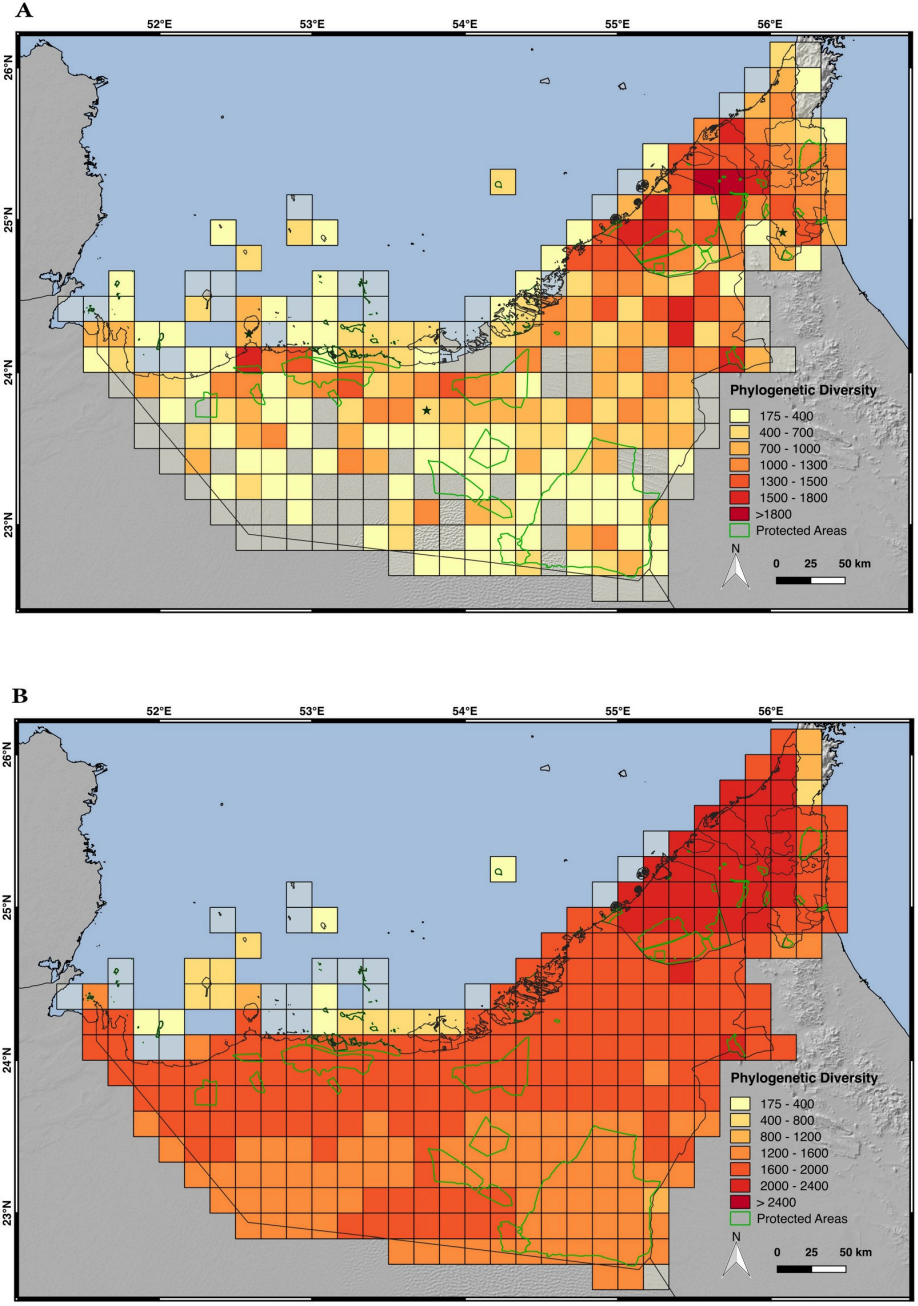
Maps of phylogenetic diversity (PD). A) PD by grid cells of 10 arc-minutes of longitude and latitude, using the area of occupancy of each species calculated with the occurrence point data; B) PD by grid cells of 10 arc-minutes of latitude and longitude inferred with the species distribution models; the terrestrial protected areas of the UAE are shown in light green; black stars represent the cells with values of PD significantly different than the expected by random. Only the 57 native species have been included in the analyses. Credits: OpenStreetMap contributors, SRTM.

The results obtained by analyzing the PD of the 57 native terrestrial reptiles and representing the values at a 10 arc-minute grid resolution for the AOO approach (Fig 10A), show that the area with high values of PD is mostly distributed in the northeast of the country, reaching the highest values in Sharjah (with some grid cells with more than 1,800 million years–my–of PD). The grid cells with the highest values of PD correspond to the grid cells with the highest values of species richness. In fact, both methods show a strong positive correlation (R = 0.955), indicating that the places with high species richness tend to have higher values of PD (S8 Fig). As seen in the species richness map, PD also has a peak in the southwest coast of the UAE (1,500–1,800 my of PD) and in the inland oasis city of Al Ain (1,500–1,800 my of PD). When PD is calculated using the SDM (Fig 10B), it rises in the whole country because more species share the same distribution area but like with the point data, the highest values of PD (>2,400 my) are found in the northeast of the country. On the other hand, the region with lower values of PD is the most southerly region of the UAE, where the Rub al-Khali desert has a major impact on the climate and ecological conditions. Within this region of low PD, the Liwa Oasis is an exception, with slightly higher values of PD than other arid areas. As shown in Fig 10A and 10B, there are some protected areas in regions with high values of PD, although most protected areas are in regions of low PD. In fact, the largest protected area of the country is located where PD is the lowest (under 175 my calculated using point data and under 1,600 my calculated using the SDMs). When PDses were calculated, only three grid cells (point data approach) and one cell (SDM approach) had different values of PD than expected by random. In all four cases, PD values were lower than expected.

### Evaluation of the evolutionary distinctiveness (ED) of the UAE terrestrial reptiles

The values of ED for each one of the 60 UAE terrestrial reptiles are presented in S10 Table. Three categories were created depending on the ED values of each species: low ED (<48); medium ED (48–96); high ED (>96). Six species (10%) fall within the high ED category: three snakes (*Myriopholis macrorhyncha*, the introduced *Indotyphlops braminus* and *Eryx jayakari*), one gecko (*Teratoscincus keyserlingii*), one amphisbaenid (*Diplometopon zarudnyi*) and one varanid (*Varanus g*. *griseus*), the latter being the species with the highest value of ED (159.17), since it is the species with the most independent evolutionary history not shared with other reptile species from the UAE. Thirty-nine species (65%) have low values of ED and 15 species (25%) have medium values of ED.

**Fig 11:**
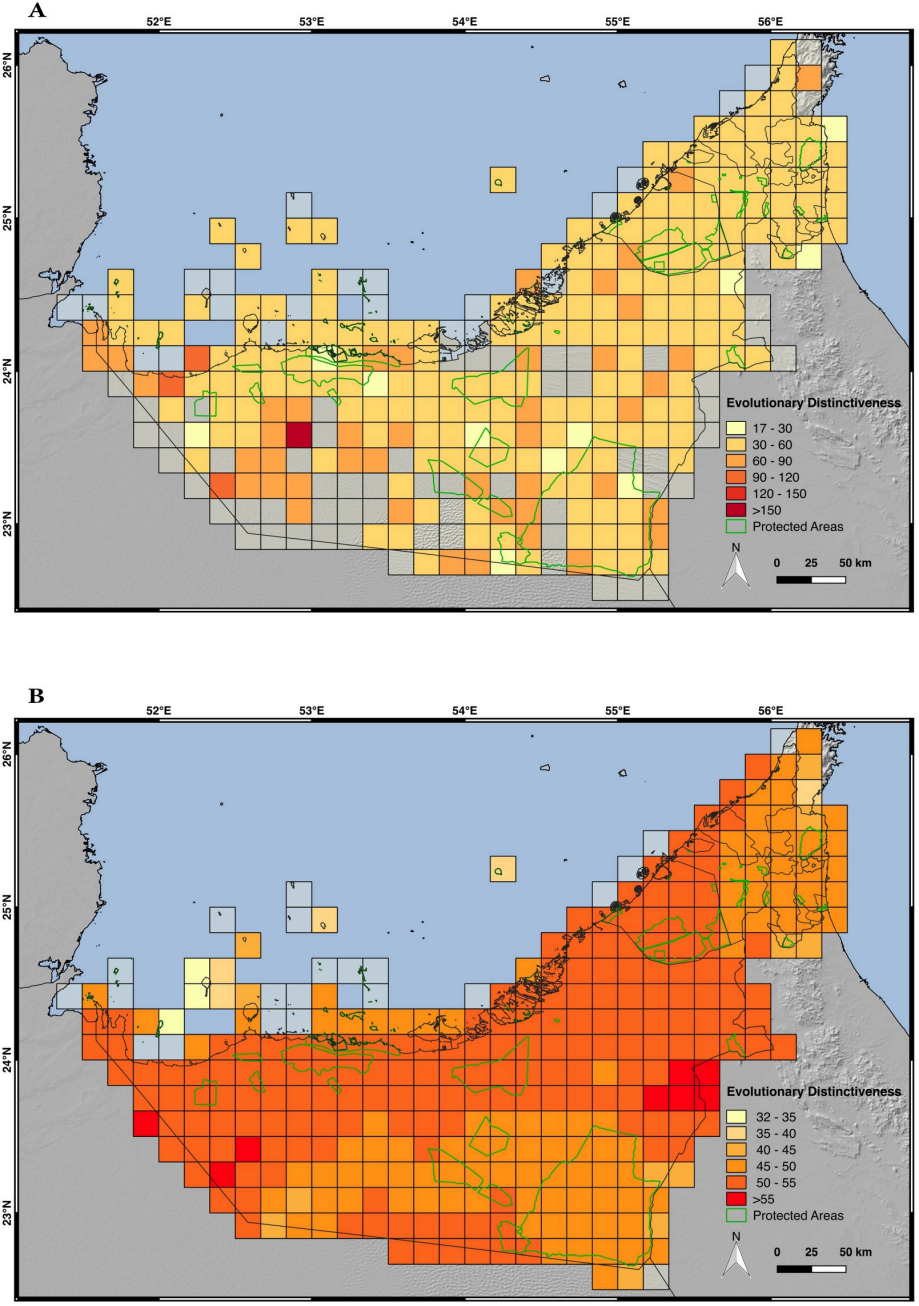
Maps of evolutionary distinctiveness (ED). A) ED by grid cells of 10 arc-minutes of longitude and latitude, using the area of occupancy of each species calculated with the occurrence point data; B) ED by grid cells of 10 arc-minutes of latitude and longitude inferred with the species distribution models; the terrestrial protected areas of the UAE are shown in light green. Only the 57 native species have been included in the analyses. Credits: OpenStreetMap contributors, SRTM.

The average ED of all the 57 native species at a resolution of a 10 arc-minute grid using both point data and SDM approaches (see Fig 11A and 11B, respectively) shows a very different pattern to the species richness and PD (Figs 6 and 10, respectively). The places with the highest values of species richness and PD, such as the region of the Hajar Mountains, show the lowest values of average ED, while the highest values of ED are located in the eastern border of the UAE with Oman, below Jebel Hafeet and in the inland desert of Rub al-Khali, about 36 km east from the western border with Saudi Arabia. Apart from these areas, there are two other cells with high values of ED but they are considered artefacts since they only cover a minimal part of the country and the rest of the cell is either in the sea or in a neighboring country. Most of the cells range between 30–90 (point data approach) and there is a peak of ED in a single cell in the desert located about 90 km east from the western border with Saudi Arabia. This peak of ED is the result of the patchiness of the dataset, as of all 60 species this particular grid cell only contains records for *Varanus g*. *griseus*. The ED value of this grid cell is an artifact because when the SDMs are used instead of the raw point data the peak of ED disappears, and the cell acquires a value comparable to the surrounding cells.

## Discussion

### The terrestrial reptile fauna of the UAE

In the present work, we assembled and analyzed an unparalleled database that included 5,535 records of all 60 known species of terrestrial reptiles of the UAE, gathered over many years of field research and systematic work using morphological and molecular data, analyzed with multivariate, phylogenetic and population genetic methods among others (see [19, 24–26, 59]). The considerable research efforts of previous studies together with the use of DNA techniques, which have revolutionized systematic studies, have provided new information on the reptile fauna of the UAE, discovering high levels of undescribed diversity and increasing species richness over 5% compared to previous systematic research (see [19] and references therein).

As has been shown on multiple occasions, taxonomy has a profound impact on conservation [1, 13–14, 36, 60]. A robust and well resolved taxonomy of the terrestrial reptiles contributes to a better understanding of the real diversity of this relevant vertebrate group in the UAE, hence providing a solid background for conservation in the country. A lack of taxonomic knowledge (the Linnean shortfall [61]) can have a potentially great impact on conservation planning. Species that are considered common and widely distributed may actually hide multiple species, each with a small distribution range and hence being a potential target for high priority conservation. Some examples of the importance of taxonomy in conservation and the reduction of the Linnean shortfall in the UAE are illustrated by the recent split of various taxa, such as the geckos of the genera *Ptyodactylus* [25] and *Asaccus* [24]. Of the latter, *A*. *caudivolvulus* used to have a wide distribution across the northern Hajar Mountains of Oman and the UAE, and was considered as Least Concern by the IUCN. However, it was recently split into three microendemic species: *A*. *gardneri*, *A*. *margaritae* and *A*. *caudivolvulus*, with very restricted distributions and considered Least Concern, Vulnerable and Critically Endangered, respectively by the 2018 UAE Red List of Reptiles and Amphibians (unpublished). Furthermore, *A*. *caudivolvulus* has become the only endemic vertebrate of the UAE and has an extremely restricted distribution in a small coastal area at risk from heavy development [24]. Another example includes *Ptyodactylus ruusaljibalicus* and *P*. *orlovi*, both previously considered conspecific with *P*. *hasselquistii* and now recognized as two different cryptic species with allopatric distributions [25, 59, 62]; the first one restricted to the northern Hajar Mountains, in the Ru’us al Jibal area, and the latter distributed across the rest of the Hajar Mountains [25].

These newly discovered species with restricted distributions, and the revaluation of the national conservation status of the terrestrial reptiles of the UAE, have made it possible to detect new threatened species, which were not taken into consideration in the last assessment [26]. The number of threatened species in the UAE has quadruplicated from two (3.3%) to eight (13.3%). Also, only five taxa of terrestrial reptiles are covered by the 2010 federal legislation of the UAE [63]: two subspecies of the agamid species *Uromastyx aegyptia* (*U*. *a*. *microlepis* and *U*. *a*. *leptieni*), two species of geckos (*Pristurus rupestris* and *Stenodactylus slevini*) and the varanid *Varanus g*. *griseus*. Of these, only two taxa (both subspecies of *Uromastyx aegyptia*) are classified as threatened. The other three taxa are listed as LC by the IUCN. One of them, *Pristurus rupestris*, has been the subject of a recent integrative taxonomic study, which has shown that it is a species complex with several cryptic deep lineages [64]; the one present in the UAE being still undescribed and temporarily named *Pristurus* sp. 3 [1] (*Pristurus rupestris*–sp. 3 in the present manuscript).

Conservation efforts are usually limited, as for political and economic reasons it is impossible to preserve all species and habitats. Therefore, conservation efforts should be targeted at the most threatened species with clear danger of extinction. In the UAE, a reevaluation of the conservation policy for the terrestrial reptile species should be considered, so that the newly assessed CR and VU species are included under conservation legislation.

Out of the total of 60 species of UAE terrestrial reptiles, three are considered introduced: the skink *Chalcides ocellatus ocellatus*, the gecko *Hemidactylus flaviviridis*, and the snake *Indotyphlops braminus* (S1 Appendix; 2018 UAE Red List of Reptiles and Amphibians). Apparently, none of these three introduced species are competing with the native fauna, or are causing any damage to the environment, human economy or human health [1, 19]. In fact, in the UAE all three species are linked to humanized habitats. The originally North African-Mediterranean *Chalcides o*. *ocellatus* is found mainly in residential gardens, oases, orchards and city parks from sea level to 300 m. Fortunately, there is no native congeneric species of *C*. *o*. *ocellatus* and the skink *Trachylepis tessellata* occupies a different niche in the mountains, which reduces the possibilities of impact by competence. The originally Indian *Hemidactylus flaviviridis* is exclusively associated with human habitations including houses and ruins from sea level to 500 m and is of much concern since there are a couple of native, congeneric species, within the country. Finally, the tropical Asian parthenogenetic burrowing snake *Indotyphlops braminus* has only been recorded from urban areas of Dubai and Abu Dhabi from sea level to 100 m (see [19] and S1 Appendix). In the present work, the three introduced species have not been included in the calculations of species richness, PD, ED and in the conclusions of the gap analysis (Figs 6 and 9–11). Despite the fact that apparently the three introduced species are not interfering negatively with the native fauna of the UAE, it has been reported that *Chalcides o*. *ocellatus* and *Hemidactylus flaviviridis* are expanding their range due to human activities, including transport of goods, and the global population is presumed to be increasing as a result [19]. Moreover, in the case of *H*. *flaviviridis*, locally the species can be quite abundant [19]. Therefore, it is important to start monitoring and control the spread of these three reptile species introduced to the UAE.

The present work is not only remarkable from a taxonomic point of view, with multiple observations for most species, but also for the sampling effort and the spatial coverage achieved. This work has covered reptile observations from over a 75% of the total area of the UAE (divided into a 10 arc-minutes grid). Observations are well represented across almost the entire climatic space of the UAE defined by the first two components of the PCA (PC1 and PC2), only lacking observations in cluster 1, which has an area of just 13 km^2^ (0.00015% of the country area). This thorough evaluation of the UAE territory has revealed different patterns of species richness, phylogenetic diversity and evolutionary distinctiveness, and has enabled the reassessment of the effectiveness of the current network of protected areas of the UAE in conserving the reptiles. Detailed discussions of these outcomes are summarized below.

### Patterns of species richness

The highest values of species richness have been detected in the emirate of Sharjah, where up to 27 different species were found in a single grid cell of 10 arc-minutes according to point data and more than 40 species according to the SDM approach (Fig 6). Other regions with higher values of species richness than the average are located in the Jebel Hafeet, near Al Ain city and in the surroundings of the most populated cities of the UAE, such as Abu Dhabi and Dubai. Although the results of the analyses with point data (Fig 6A and 6B) and SDMs (Fig 6C and 6D) are slightly different, it is clear that the eastern part of the UAE harbors the highest levels of species richness. One interesting pattern is that the Hajar Mountains do not seem to have high levels of species richness when point data are analyzed, while they are among the areas with the highest levels of species richness when the SDMs are used. This result can be explained by the difficulty of sampling across the rugged terrain and inaccessible areas of the Hajar Mountains, which has reduced the number of available point data across this area potentially suitable for some species. High values of species richness in the emirate of Sharjah area in the point data analysis are near the city of Al Dhaid (Fig 6B) and are most likely due to the high levels of prospection around the Sharjah Desert Park. The high levels of species richness include several nearby grids both by the coast and in the Hajar Mountains (Fig 6D). Another peak of species richness can be found in the Jebel Hafeet region. Again, this peak is more pronounced in the analysis carried out using point data than SDMs (Fig 6C and 6D), the latter also highlighting as areas of high species richness other grids to the north and especially to the west of Al Ain, up to the Abu Dhabi coast. The high level of species richness on the Jebel Hafeet can be explained by the higher sampling effort in the area and the presence of a wide range of heterogeneous habitats. The Jebel Hafeet is a mountain that reaches up to 1,249 m and provides refuge for several species that are not so well adapted to the other more arid regions. Also, at the foot of the mountain, in Al Ain city, an oasis is found, increasing habitat heterogeneity and making it a hotspot of species richness in the UAE.

Only one species of terrestrial reptile (*Asaccus caudivolvulus*) is endemic to the UAE and it is located in the Hajar Mountains. However, the endemic species’ richness would increases considerably if the Hajar Mountains, a top hotspot in Arabia with over 21 endemic species described so far [1, 65], would be considered as a continuum, regardless of the UAE and Oman borders. Geopolitical borders between these two countries split the Hajar Mountains into two regions (the Hajar Mountains in the UAE and the Hajar Mountains in Oman) but species are not restricted by these political borders, thus being arbitrary as far as biogeography is concerned. However, the present study is an atlas of the UAE terrestrial reptiles because the conservation actions are mostly administratively constrained; in this case to countries. Analyses within the geopolitical limits of a country are useful because most conservation decisions and budgets are planned independently by country [66, 67]. Thus, it is advisable to have information on the diversity, distribution and conservation of the reptile faunas separately for each country.

#### Patterns of medically important venomous species richness

The only medically important venomous terrestrial reptiles from the UAE are the only four snakes of the family Viperidae found in the country (see below). S5A and S5B Fig shows that their greatest richness based on point data is located in the northeastern part of the country, with two grids in the Hajar Mountains range having the highest number of venomous species (three out of four). The analyses using SDMs also support that the highest levels of medically important venomous richness are in the northeast of the UAE, although in this case several grids in the western parts of the Hajar Mountains had all four viperid species (S5D Fig). Two of the species, *Echis omanensis* and *Pseudocerastes persicus*, have a wide altitudinal range of occurrence (from sea level up to 1,300 m) and mostly prefer rocky mountainous environments. On the other hand, the other two species (*Cerastes gasperettii gasperettii* and *Echis carinatus sochureki*) are more common at low elevation areas, under 300 m and prefer sand desert environments. Since most of the UAE is covered by one of these two types of land cover, venomous snake species can theoretically be found in almost every part of the country. Nevertheless, there have not been any records of venomous snakes in any of the many UAE islands, meaning that they have not been able to disperse further enough to arrive there or colonize yet [19]. Finally, it is important to take into account that although in almost every part of the country a person could potentially encounter a venomous snake, snakebites in the UAE are rare. This might be the case because we are dealing with four snakes of very shy behavior, rather scarce within the desert biome that dominate the country, and where the human population is also quite low out of urban areas. The probability of snakebite frequency relies upon human coincidence with poisonous snakes, and from the previous comments, it follows that this probability is very low.

### Patterns of phylogenetic diversity (PD)

This study has included some novel techniques merging spatial and ecological data with phylogenetic analyses in order to evaluate the phylogenetic diversity of the terrestrial reptiles of the UAE. This integrative approach can be crucial for evaluating species in regard of their evolutionary history and for detecting those places where more species with different evolutionary paths (more phylogenetic diversity) co-occur. However, we have to be cautious since sometimes having a higher value of PD does not imply a major necessity for protection. For example, if a certain location contains five species of geckos all closely related to each other (including several local endemics), while another location has distinct, but widespread species such as two geckos, an agamid, a snake and a varanid, the latter will have a much higher PD than the former one but it could be argued that, since the former area contains several endemic species, it should be of conservation priority. This example would become even more complex if we added into consideration the IUCN categories of each species and determine which of them are threatened. What we can see from this example is that conservation prioritisation is a complex task and not single metric can capture all aspects of biodiversity [68]. However, phylogenetic metrics such as PD or ED can be very useful since they provide new angles to the commonly used metrics based on population trends, geographic distributions and abundances, such as the IUCN red list of threatened species.

A strong correlation between patterns of PD and species richness has been observed; those areas with highest values of species richness also being the ones with the highest values of PD. It is logical to have similar patterns in both methods, since if there are more species in a specific area the total sum of their different evolutionary paths will normally be higher than in other places with less species [10]. Places that do not match this correlation are very interesting, since the PD of these cells is not explained by random processes, but by other variables, which might be environmental, anthropic, ecological or phylogenetic among others, diminishing or increasing the PD. In the UAE, we have encountered three grid cells of 10 arc-minutes (point data) which have statistically lower PD than expected by random: the first one in the island Sir Bani Yas and the coastal land in front of it (1,140 my of PD), the second near the town of Madinat Zayed, in the inland desert of Abu Dhabi (570 my of PD) and the third in the northeast of the country, near the village of Al Qawr in the northern border with Oman (767 my of PD) (Fig 10A). The reasons why the species of these cells are more phylogenetically related to each other than expected by random is still unknown. None of the three localities has a very high or very low PD and the composition of species is different in each grid cell: the first grid cell, 16 species (eight geckos, six snakes, one skink and one lacertid); second grid cell, six species (four geckos and two agamids); third grid cell, nine species (four geckos, four snakes and one agamid). Moreover, none of the three grid cells are in a similar ecological, climatic or topographic space; the first being coastal, the second in the desert and the third in the Hajar Mountains. However, the lack of significance when the SDM is taken into account points towards an artifact of differential sampling effort. Future studies should evaluate these three grid cells with further detail to elucidate why the species that live in these three areas share more evolutionary history between them than expected by random. The grid cell that had been detected as having a statistically significant lower value than expected by average in the SDM approach is believed to be an artifact, since the cell with this value occupies a minimal part of the UAE, being mostly located in Oman and showing a similar pattern of low PD as other border cells (Fig 10B). To better discern the patterns of diversity in these border cells and, as previously recommended, for better understanding biodiversity from a biogeographic (and not geopolitical) point of view, an analysis integrating the data from the UAE and Oman should be carried out.

Finally, as for the PD of protected areas, it is important to notice that even though the protected areas do not cover all the grid cells with high PD values these areas are present in most high-PD grid cells. On the other hand, we have found that the largest protected areas are located where PD is the lowest, meaning that there is a mismatch between biodiversity and protected areas in the country, at least with respect to reptiles. However, we do not think that the method used to evaluate the PD of the country at a resolution of 10 arc-minutes cells is the best method to evaluate the level of PD protected by the network of protected areas. It is proposed that to better evaluate the level of protection of PD by the network of protected areas of the country, future studies should focus into determining the exact PD and PDses (standardized effect size of PD) inside each protected area.

### Patterns of evolutionary distinctiveness (ED)

The ED is a crucial analysis to identify the importance of each species regarding the uniqueness of their evolutionary history in the region of study [11] and, together with other analyses or evaluations such as the conservation categories of the IUCN red-listing procedure, can be a very useful tool to detect those species in most urgent need of protection within the study area. However, assessing not only each species’ ED but also the ED of the entire country divided by grid cells of 10 arc-minutes we have been able to detect which areas of the UAE hold a higher evolutionary distinctiveness. The ED analysis of the UAE terrestrial reptile fauna has revealed that the species with highest ED are *Varanus g*. *griseus* and *Diplometopon zarudnyi*. These two species are the only representatives of the families Varanidae and Trogonophidae, respectively, in the UAE, and therefore present a much longer evolutionary path than any other UAE reptile species. All metrics have their pros and cons and each one of them has to be weighted in order to decide which analyses are going to be used. As an example, the gecko *Teratoscincus keyserlingii* is listed as Least Concern (LC) in the global red list of the IUCN but, it is considered Critically Endangered (CR) at a regional scale for the UAE. As stated above, conservation acts and projects are usually administratively constrained and are mostly carried out by countries [66, 67]. Therefore, conservation efforts for not losing biodiversity in a geopolitical area are common, even though the concerned species might not be threatened globally. Species such as *Varanus g*. *griseus* and *Diplometopon zarudnyi* might not be threatened with extinction, are widespread along the country and have other sister species outside of the UAE, but they are key species from a phylogenetic point of view, since they have high value regarding the preservation of evolutionary paths at the regional scale of the UAE.

When the ED of the entire country was calculated (including only the 57 native species), a peak appeared in the eastern border between the UAE and Oman, below the Jebel Hafeet (see Fig 11B) and in the southwest of the country, about 36 km east from the western border with Saudi Arabia in the inland Rub al-Khali desert. These areas with the highest uniqueness of evolutionary history are not located inside any of the UAE protected areas and therefore future studies and conservation plans should target them. However, when the ED of the country divided by grid cells was calculated some artifacts appeared. There was a cell with an extremely high value of ED, which was considered an artifact resulting from the calculation of the ED value as an average of all independent ED values of the species present in the grid cell. We believe that the method used to establish the ED value of each cell might bias the results towards those grid cells with few species that have high ED values. Therefore, other methods should be investigated and applied. A possible approach that could be implemented instead of the ED, is the Evolutionary Distinct Globally Endangered (EDGE) technique [12], where not only the uniqueness of the species is taken into account but also its conservation status.

### Detecting future survey areas

Even though the results presented here are based on the most comprehensive dataset of the UAE reptiles with the best spatial coverage available to date, there are still areas with unique climatic conditions that remain unsampled, such as climatic cluster 1. This cluster is located in the northernmost part of the Hajar Mountains and it is restricted to high elevations. Although this cluster comprises a very small area of the country, future research should target this unsampled climatic cluster. There is also a great dissimilarity in the amount of sampled area among other clusters, ranging between 1.4–21% of area sampled. Future surveys should also focus on clusters 5 and 6, which have the lowest proportion of their area covered. Finally, according to Fig 2, some areas of the southwest of the country entering into the Rub al-Khali desert and of the coastal area and the inland desert below Abu Dhabi remain unsampled (Fig 2). Future surveys should be directed towards those areas too.

### Reassessing the protected areas

The 42 protected areas of the UAE cover 16.6% of the country, are widely distributed, and include at least some distribution area for 51 (AOO approach) and 53 (SPD approach) species of the 57 native terrestrial reptiles of the UAE. Nevertheless, when the more restrictive Aichi biodiversity target is applied, the protected areas can be considered both as providing sufficient protection for the reptile species (75.4% of the species are over the 17% target; AOO) or deficient protection (42.1% of the species are over the 17% target; SPD). This discrepancy between the two methods of analysis may have several explanations: i) although a substantial sampling effort has been made, some species are still underrepresented in the dataset and their distribution records do not cover their whole range within the UAE, thus the area calculated as the number of unique observations (AOO) is underestimated for some species; ii) due to the widespread distribution of desert-like habitats in the UAE, the SDMs tend to predict large distribution areas for species adapted to these habitats; iii) SDMs do not account for factors as competition between species or limited dispersal abilities, which may result in overestimation of species ranges. We, however, optimized the resulting models to account for the above issues and produced rather conservative predictions.

Although the protected areas are distributed across the country, there are some regions with unique conditions which are currently unprotected. Two of the six climatic clusters do not have any protected areas Clusters 1 (13km^2^) and 2 (166.7km^2^), both located in the Hajar Mountains, represent different environmental landscapes that are likely to be worth protecting. The largest protected area is located in the emirate of Abu Dhabi, covering the southern part of the country in the desert of Rub al-Khali. This large protected area is located in a region with one of the lowest species richness, PD and ED of the whole country. Nevertheless, some of the cells with greatest species richness and PD of the UAE are within protected areas, which contributes to the protection of some hotspots of diversity.

It is also of great concern that four out of the nine threatened species, including the only endemic species of the UAE: *Pristurus carteri* (CR), *Asaccus margaritae* (VU), *A*. *caudivolvulus* (CR; endemic), and *Platyceps ventromaculatus* (VU), do not occur inside any protected area, and two of the remaining threatened species are below the 17% conservation target: *Teratoscincus keyserlingii* (CR) and *Uromastyx aegyptia leptieni* (VU). Also, neither of the two regions with the greatest evolutionary distinctiveness are located within protected areas.

## Supporting information

S1 Appendix Atlas of the terrestrial reptiles of the UAE. An appendix showing photographs, distribution and ecological preferences of the 60 species of terrestrial reptiles of the UAE. Species distribution models (SDMs) inferred for each species are also included. Credits for maps: OpenStreetMap contributors, SRTM.
(PDF)

S2 Appendix List of calibration points. List of the 13 calibration points used to calibrate the phylogenetic tree of Squamata and including all the UAE terrestrial species. Each calibration point includes the author and all the parameters used to implement the calibration point in BEAST.
(PDF)

S3 Appendix Spatial data of the terrestrial reptiles of the UAE. Appendix including the spatial data of all the 60 species of terrestrial reptiles of the UAE in shapefile format.
(ZIP)

S1 Fig Topoclimatic characterization of the UAE. A) Map of annual mean temperature in °C (BIO1); B) Map of annual mean precipitation in mm (BIO12); C) Map of land cover types (as of 2008); D) Graph of the frequency of elevations divided into 100-m bins. Credits: OpenStreetMap contributors, SRTM.
(TIF)

S2 Fig Political map of the UAE. Map showing the political borders of the UAE and among the seven emirates. Credits: OpenStreetMap contributors, SRTM.
(TIF)

S3 Fig Observations map. Map with all the 5,535 occurrence points of UAE terrestrial reptiles used in the present study. Credits: OpenStreetMap contributors, SRTM.
(TIF)

S4 Fig Main taxonomic groups observed. Pie charts showing higher taxonomic composition of the UAE terrestrial reptile species (A) and the number of observations for each of the taxonomic groups used in this study (B). Numbers in parenthesis are the number of species (A) and observations (B).
(TIF)

S5 Fig Species richness maps of the medically important venomous species. A) Venomous species richness by emirate inferred with the occurrence point data; B) Venomous species richness by a 10 arc-min grid inferred with the occurrence point data; C) Venomous species richness by emirate inferred with the species distribution models; D) Venomous species richness by a 10 arc-min grid inferred with the species distribution models. The four species of medically important venomous species of UAE terrestrial reptiles are the snakes *Cerastes gasperettii gasperettii*, *Echis carinatus sochureki*, *Echis omanensis* and *Pseudocerastes persicus*. Credits: OpenStreetMap contributors, SRTM.
(TIF)

S6 Fig Maximum likelihood (ML) tree obtained with RaxML. Phylogenetic tree based on the concatenated dataset of 15 genes and 146 species of Squamata including all 60 UAE terrestrial reptiles (highlighted in green) and one outgroup. Asterisks highlight the three introduced species.
(TIF)

S7 Fig Time calibrated tree of Squamata obtained with BEAST. Time calibrated tree based on the concatenated dataset of 15 genes and 146 species of Squamata including all 60 UAE terrestrial reptiles (highlighted in green) and one outgroup inferred with BEAST. The 13 calibration points are indicated as red circles and are labelled as in S2 Appendix which gives further details. The topology of the time calibrated tree was fixed to the ML tree shown in S6 Fig The scale below the tree is in millions of years. Asterisks highlight the three introduced species.
(TIF)

S8 Fig Correlation between species richness and phylogenetic diversity. Points in the graph are cells of the 10 arc-min grid. The regression line was fitted using a linear model.
(TIF)

S1 Table List of all the 146 species of reptiles included in the phylogenetic analyses. For the UAE species the table includes species name, the country and specimen code.
(PDF)

S2 Table Information on the dataset used for the phylogenetic analyses. Gene composition, fragment lengths, partitions, models and run specifications for the different phylogenetic analyses are shown.
(PDF)

S3 Table Checklist of the 60 species of UAE terrestrial reptiles. Table containing all the species recorded in this study grouped by families and higher taxa. The table contains information regarding the regional IUCN conservation category (yet unpublished) for all the species and indicates if they are endemic to the UAE and if it is a medically important venomous species. Asterisks highlight the three introduced species.
(PDF)

S4 Table Information on the 60 species of UAE terrestrial reptiles for the whole country and independently for each emirate. Number and percentage of the total number of species, threatened species and medically important venomous species based on presence data.
(PDF)

S5 Table Area of occupancy (AOO) and species potential distribution (SPD) at 4 km^2^ of all 60 terrestrial reptiles of the UAE. Number of occupied 4 km^2^ cells for each approach, area occupied by each species for each approach. Asterisks highlight the three introduced species.
(PDF)

S6 Table Loadings, eigenvalues, and variance explained by the two first components retained from the principal component analysis (PCA) performed on the 19 bioclimatic variables used in this study. BIO1 = Annual Mean Temperature, BIO4 = Temperature Seasonality, BIO5 = Max Temperature of Warmest Month, BIO6 = Min Temperature of Coldest Month, BIO7 = Temperature Annual Range, BIO10 = Mean Temperature of Warmest Quarter, BIO11 = Mean Temperature of Coldest Quarter, BIO12 = Annual Precipitation, BIO13 = Precipitation of Wettest Month, BIO16 = Precipitation of Wettest Quarter, BIO18 = Precipitation of Warmest Quarter, BIO19 = Precipitation of Coldest Quarter.
(PDF)

S7 Table Information on the protected areas of each emirate. The table contains: the name of each emirate, the area of each emirate in km^2^; the number of protected areas that are inside each emirate; the area in km^2^ that is protected inside each emirate and the percentage of protected areas by emirate.
(PDF)

S8 Table Information from the gap analysis with distribution records with the AOO approach. List of all 60 UAE species, showing their regional IUCN conservation category, their area of occupancy, area of occupancy inside protected areas, and the percentage of area of occupancy inside protected areas. Asterisks highlight the three introduced species. The introduced species have not been included in Fig 9 and in the analysis and discussion of the results of the gap analysis.
(PDF)

S9 Table Information from the gap analysis with distribution records with the species potential distribution (SPD) approach obtained with species distribution modeling (SDM). List of all 60 species of the UAE, showing their regional IUCN conservation category, their SPD area, the SPD area inside protected areas, and the percentage of the SPD area inside protected areas. The eight species that did not have the minimum of five records to infer their SDMs are underlined. In these cases, the SPD was calculated by the sum of all 4 km^2^ squares inside the minimum convex polygon of each species. Asterisks highlight the three introduced species. The introduced species have not been included in Fig 9 and in the analysis and discussion of the results of the gap analysis.
(PDF)

S10 Table Evolutionary distinctiveness (ED) of each of the 60 species of UAE terrestrial reptiles. The numbers show the evolutionary uniqueness of each species with respect to other UAE reptiles in millions of years. Asterisks highlight the three introduced species.
(PDF)
