# Supplementary material for: An integrative assessment of the diversity, phylogeny, distribution, and conservation of the terrestrial reptiles (Sauropsida, Squamata) of the United Arab Emirates
Source: PLoS One. 2019 May 2;14(5):e0216273. doi: 10.1371/journal.pone.0216273 (PMC6497385; doi:10.1371/journal.pone.0216273)

S1 Appendix. Atlas of the terrestrial reptiles of the United Arab Emirates

Appendix showing photographs, distribution and ecology of all the 60 species of terrestrial reptiles of the United Arab Emirates included in the present study and the species' potential distribution inferred with species distribution models (SDMs).

A HIGH-RESOLUTION VERSION OF THIS APPENDIX CAN BE DOWNLOADED FROM THE FOLLOWING WEB ADDRESS

http://purl.org/carranza/data

Supplementary information

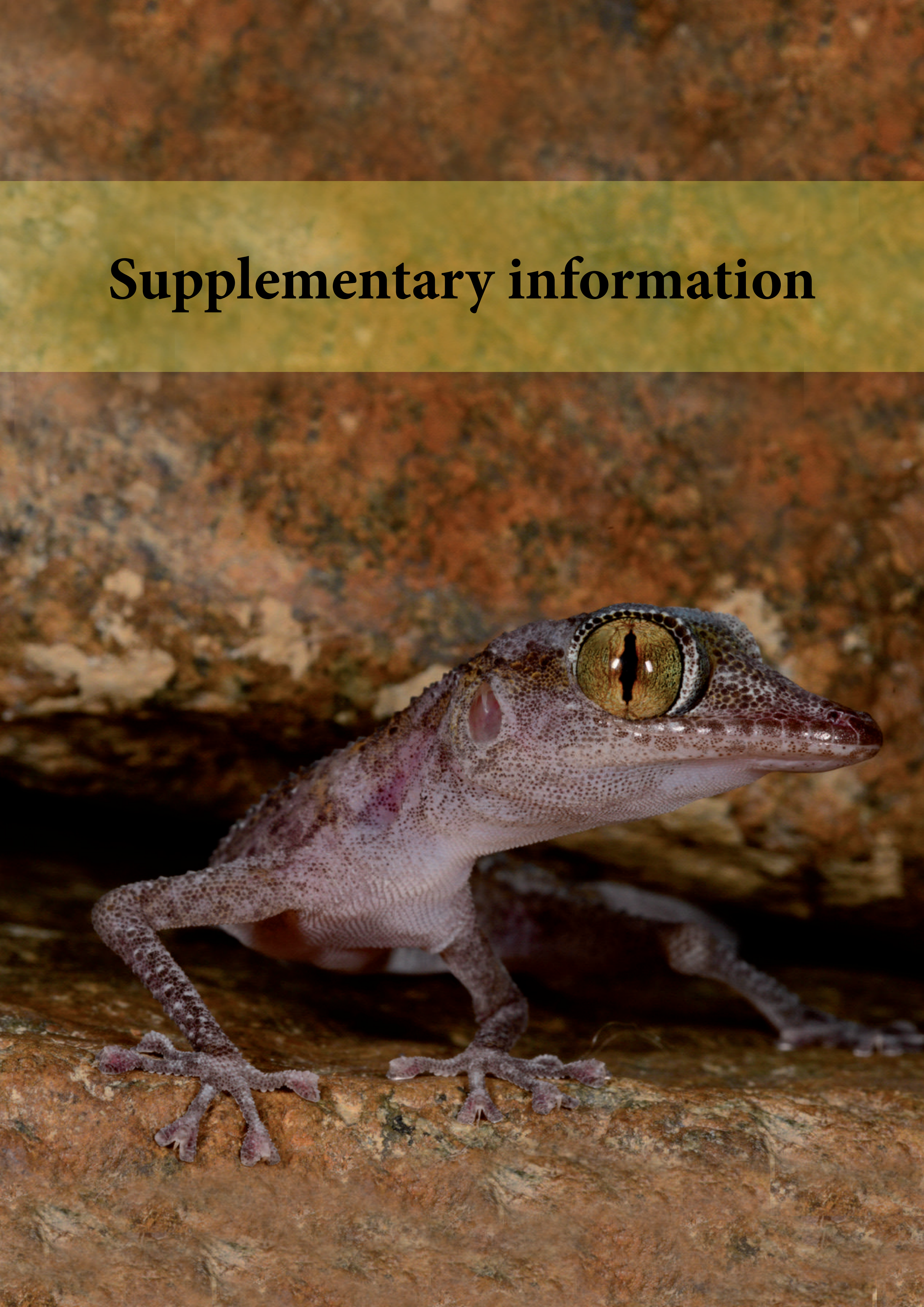

INDEX

S1 APPENDIX: ATLAS OF THE TERRESTRIAL REPTILES OF THE UAE 7
S1-S60: SPECIES INFORMATION 8
LIZARDS 9
AGAMIDS 9
Agamidae 9
Phrynocephalus arabicus 9
Phrynocephalus maculatus 10
Pseudotrapelus jensvindumi 11
Trapelus flavimaculatus 12
Uromastyx aegyptia leptieni 13
Uromastyx aegyptia microlepis 14
GECKOS 15
Gekkonidae 15
Bunopus tuberculatus 15
Cyrtopodion scabrum 16
Hemidactylus flaviviridis 17
Hemidactylus persicus 18
Hemidactylus robustus 19
Pseudoceramodactylus khobarensis 20
Stenodactylus arabicus 21
Stenodactylus doriae 22
Stenodactylus leptocosymbotes 23
Stenodactylus slevini 24
Trachydactylus hajarensis 25
Phyllodactylidae 26
Asaccus caudivolvulus 26
Asaccus gallagheri 27
Asaccus gardneri 28
Asaccus margaritae 29
Ptyodactylus orlovi 30
Ptyodactylus ruusaljibalicus 31
Sphaerodactylidae 32
Pristurus carteri 33
Pristurus celerrimus 33
Pristurus minimus 34
Pristurus rupestris-sp.3 35
Teratoscincus keyserlingii 36
LACERTIDS 37
Lacertidae 37
Acanthodactylus blanfordii 37
Acanthodactylus boskianus asper 38
Acanthodactylus gongrorhynchatus 39
Acanthodactylus haasi 40
Acanthodactylus opheodurus 41
Acanthodactylus schmidti 42
Mesalina adramitana 43
Mesalina brevirostris 44
Omanosaura cyanura 45
Omanosaura jayakari 46

SKINKS 47
Scincidae 47
Ablepharus pannonicus 47
Chalcides ocellatus ocellatus 48
Heremites septemtaeniatus 49
Scincus mitranus 50
Scincus scincus conirostris 51
Trachylepis tessellata 52
AMPHISBAENIDS 53
Trogonophidae 53
Diplometopon zarudnyi 53
VARANIDS 54
Varanidae 54
Varanus griseus griseus 54
SNAKES 55
Boidae 55
Eryx jayakari 55
Colubridae 56
Lytorhynchus diadema diadema 56
Platyceps rhodorachis rhodorachis 57
Platiceps ventromaculatus 58
Spalerosophis diadema cliffordii 59
Telescopus dhara dhara 60
Lamprophiidae 61
Psammophis schokari 61
Rhagerhis moilensis 62
Leptotyphlopidae 63
Myriopholis macrorhyncha 63
Typhlopidae 64
Indotyphlops braminus 64
Viperidae 65
Cerastes gasperettii gasperettii 65
Echis carinatus sochureki 66
Echis omanensis 67
Pseudocerastes persicus 68
S61-S80: SPECIES DISTRIBUTION MODELS 69

AUTHORS OF THE PHOTOGRAPHS

Authors list of photograph with the appendix code for each photo, and specie's photos between contents.

Amat, Fèlix (6): S3, S19, S20 and S23.

Carranza, Salvador (20): S10, S14, S16, S17, S21, S25, S26, S27, S55, S57, S58, S59 and S60.

Donaire, David (1): S39.

Els, Johannes (8): S1, S5, S7, S11, S13, S15, S18, S22, S28, S34, S38, S42, S43, S45, S46, S47, S49, S50, S51, S53 and S54.

Fernández, Daniel (6): S9, S29, S33 and S35.

Gardner, Andrew (3): S8, S24, S32 and S36.

Gebhart, Jürgen (2): S40 and S44.

Mozaffari, Omid (1): S41.

Sindaco, Roberto (6): S2, S12, S37 and S52.

Šmíd, Jiří (3): S4 and S6.

Van Andel, Priscilla (1): S31.

Supplementary information cover page: *Asaccus caudivolvulus* (Els, Johannes)
Pg 7: *Pseudocerastes persicus* (Els, Johannes)

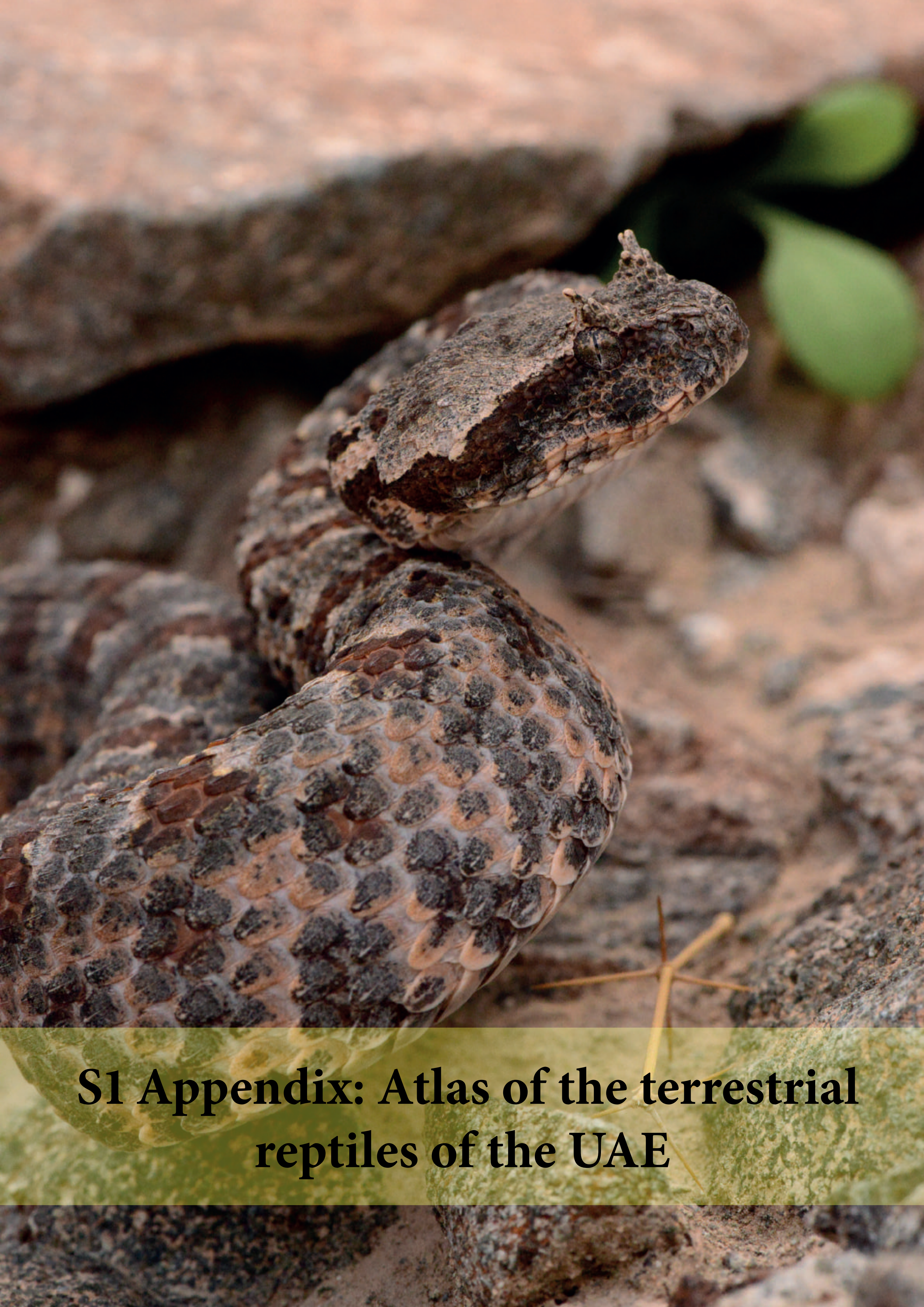

S1 Appendix: Atlas of the terrestrial reptiles of the UAE

S1- S60: Species information

There is a separate plate for each of the 60 species of terrestrial reptiles of the UAE. Each plate contains the species name, the authority (except undescribed species) and the following items:

A) Distribution map with observations (red dots) at a scale of 10 arc-minutes (~18 km).

B) Table containing the IUCN global/regional conservation category and explaining if the species is venomous, endemic or native of the UAE country. A species is considered introduced if the introduction happened after 1,500 AD.

C) Histogram of the elevation frequency of the species with 100 m intervals.

D) Picture of the species

E) Two-dimensional climatic space of UAE (grey dots) caracterized by total annual precipitation (BIO12) and mean annual temperature (BIO1). Red dots represent species distribution.

F) Boxplot of Land Cover distances. TO (Tree Open); C (Cropland); COVM (Cropland/Other Vegetation Mosaic); S (Shrub); H (Herbaceus); SV(Sparse Vegetation); BAGR (Bare Areas Gravel Rock); BAS (Bare Areas Sand); U (Urban). The top of the rectangle indicates the third quartile, a horizontal line near the middle of the rectangle indicates median, and the bottom of the rectangle indicates the first quartile. The vertical line extending from top of the rectangle indicates the maximum value, and another vertical line extending from the bottom of the rectangle indicates the minimum value. Empty circles represent outliers.

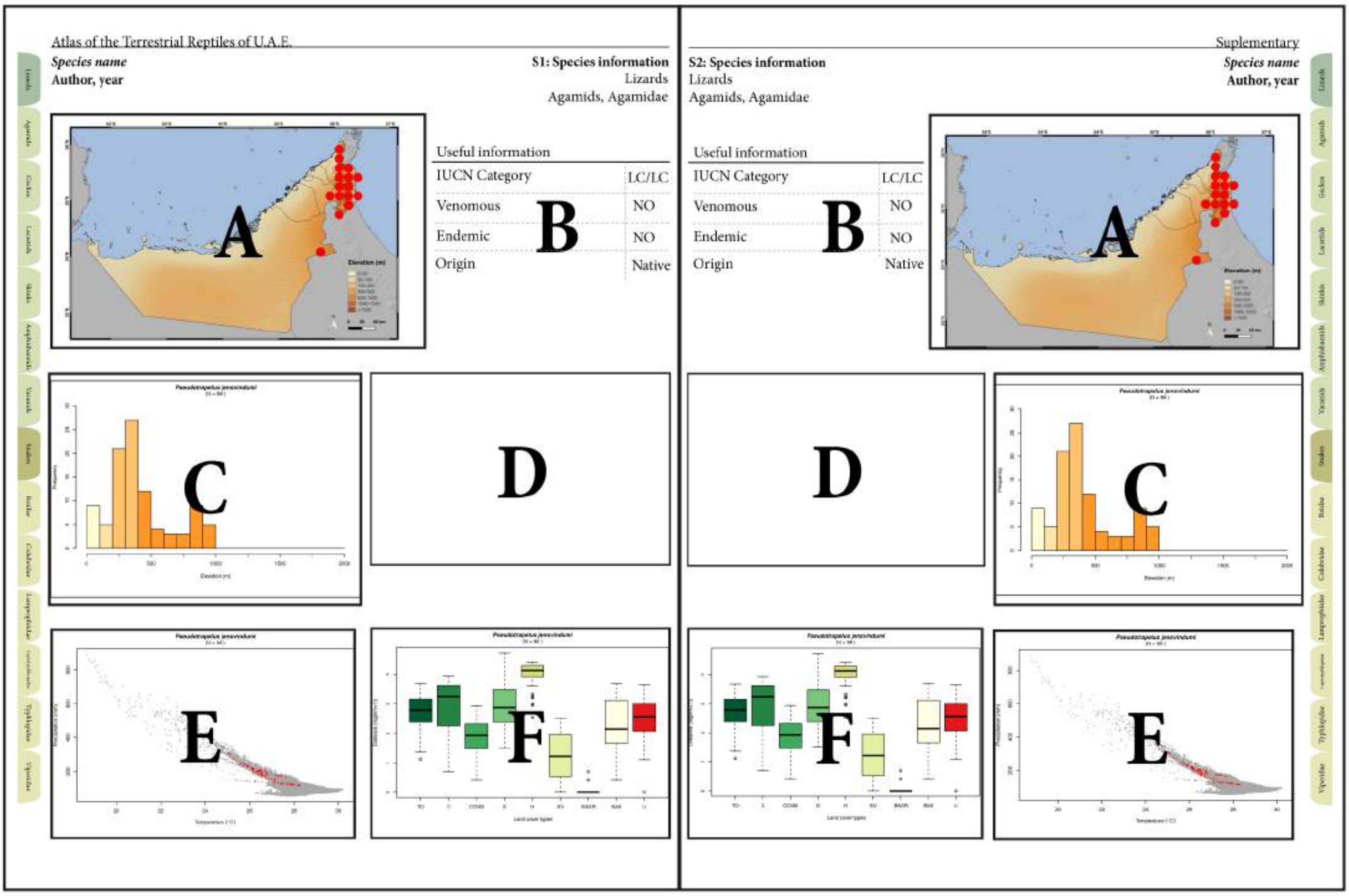

S1: Species information

Lizards

Agamids, Agamidae

Phrynocephalus arabicus

Anderson, 1894

A

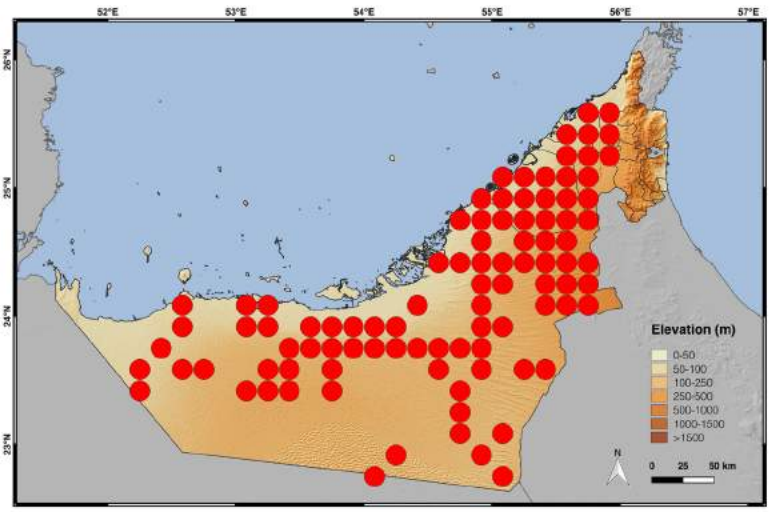

B

Useful information	
IUCN Category	LC/LC
Venomous	NO
Endemic	NO
Origin	Native

D

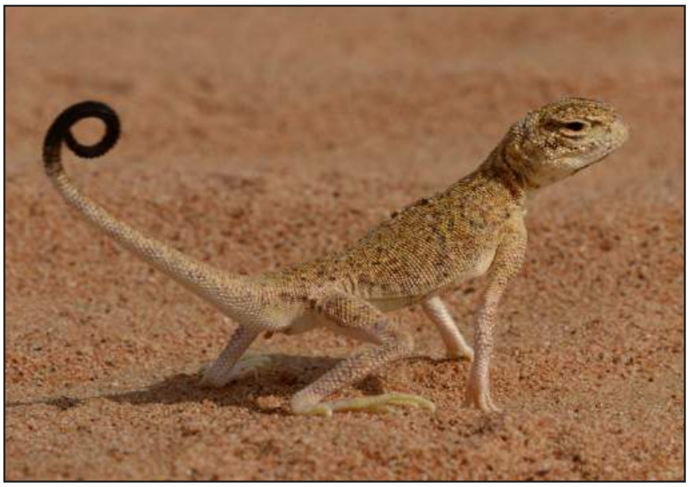

C

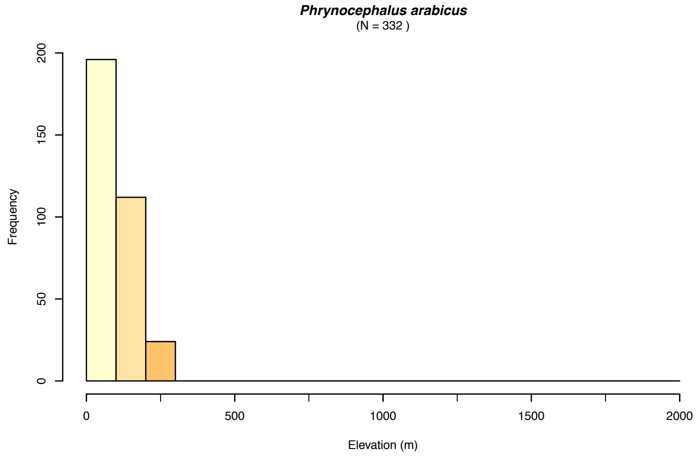

F

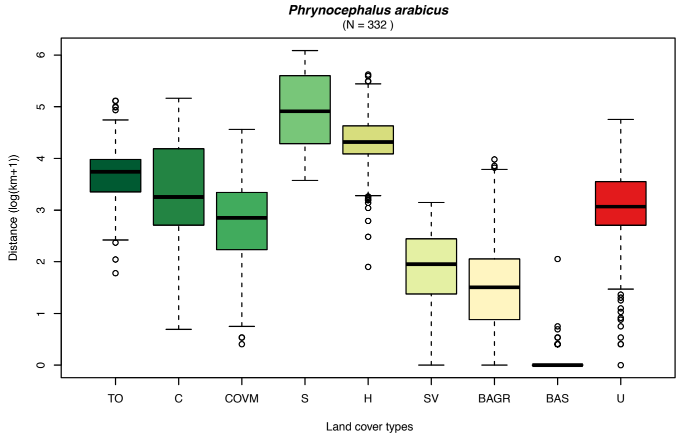

E

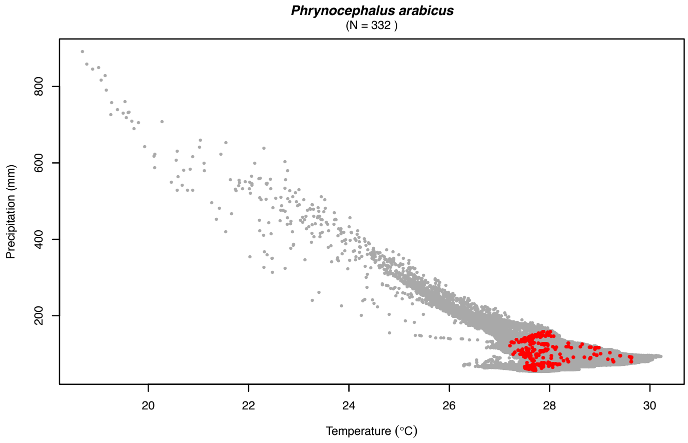

Phrynocephalus maculatus Anderson, 1872

S2: Species information
Lizards
Agamids, Agamidae

A

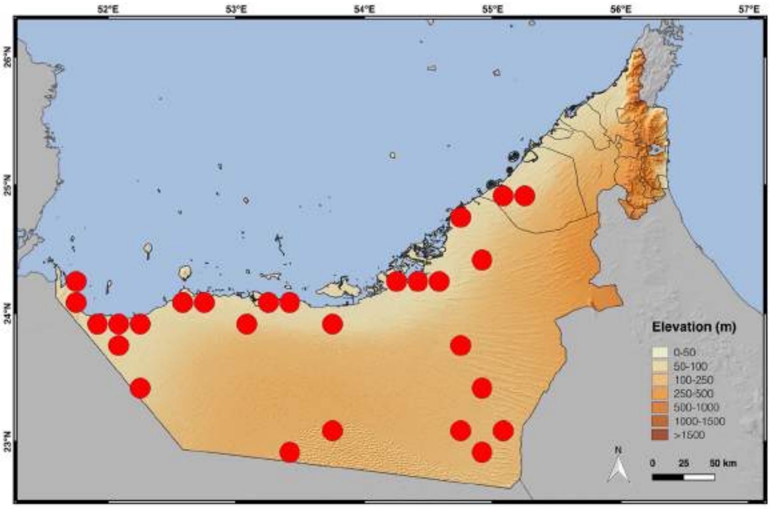

B

Useful information

IUCN Category	LC/LC
Venomous	NO
Endemic	NO
Origin	Native

C

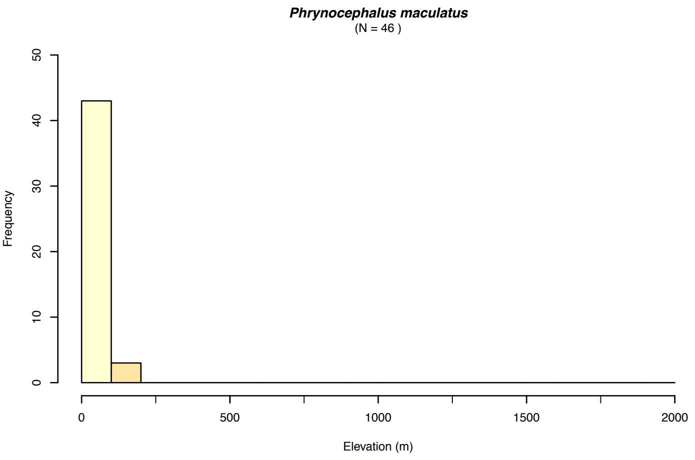

D

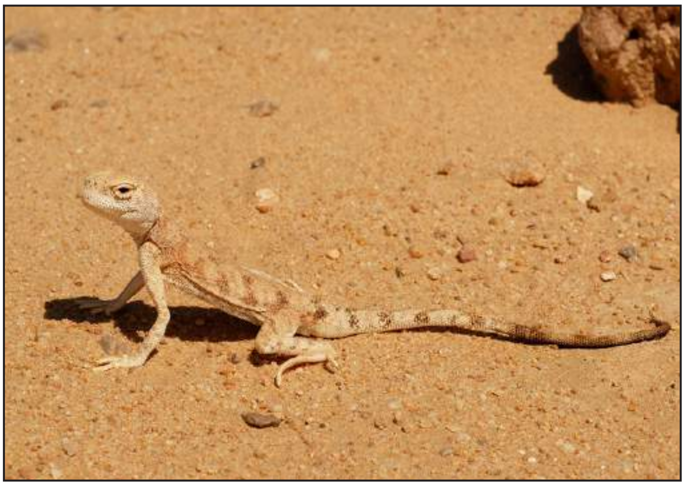

E

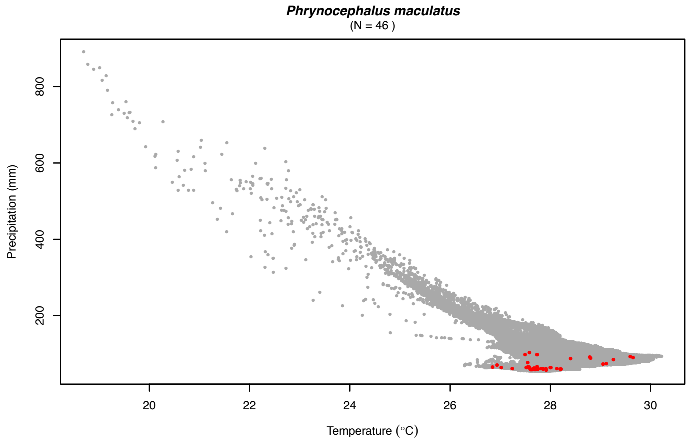

F

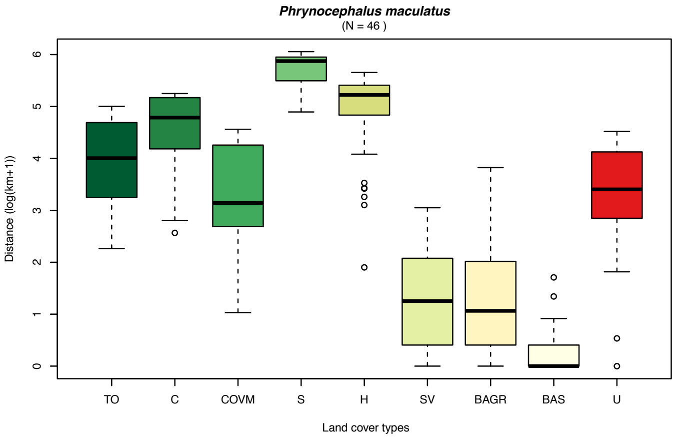

S3: Species information

Lizards

Agamids, Agamidae

Pseudotrapelus jensvindumi

Melnikov, Ananjeva & Papenfuss, 2013

A

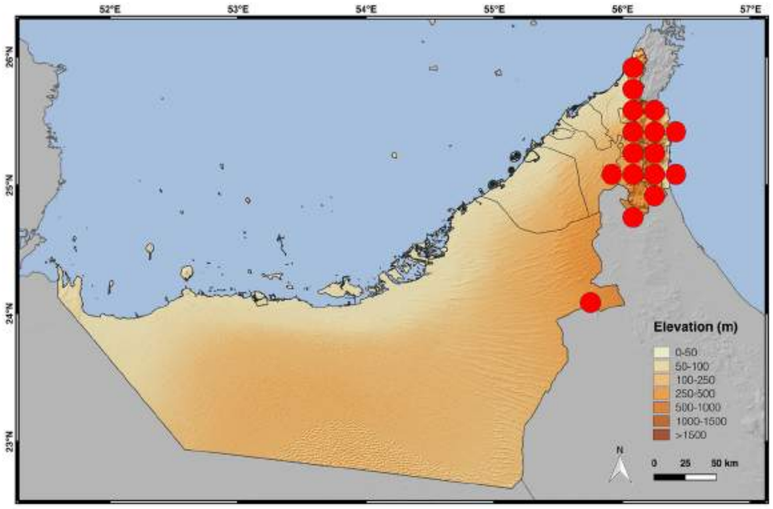

B

Useful information

IUCN Category	LC/LC
Venomous	NO
Endemic	NO
Origin	Native

D

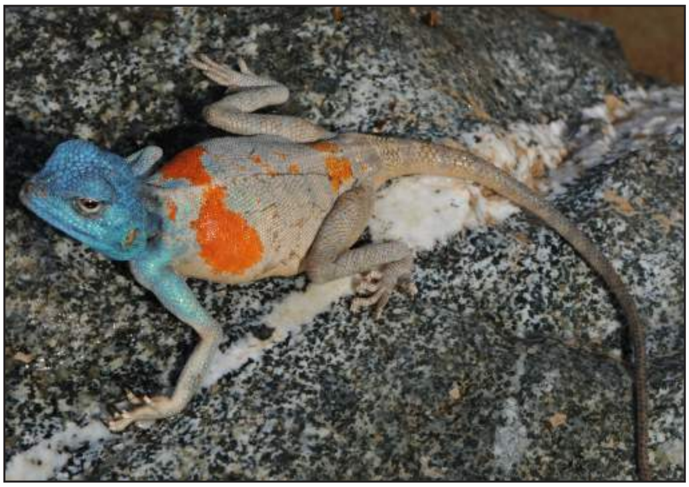

C

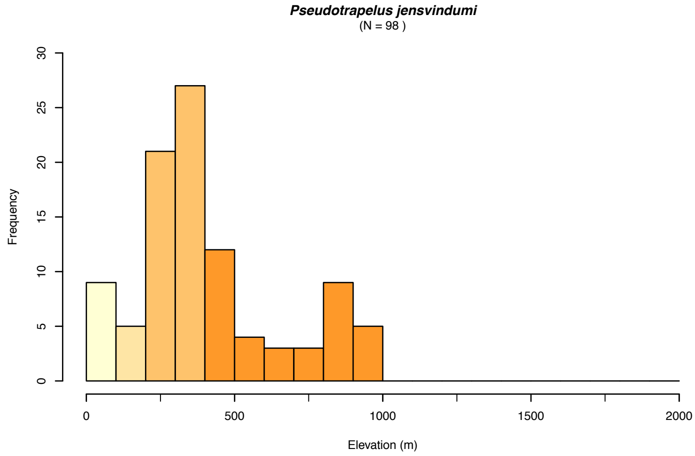

F

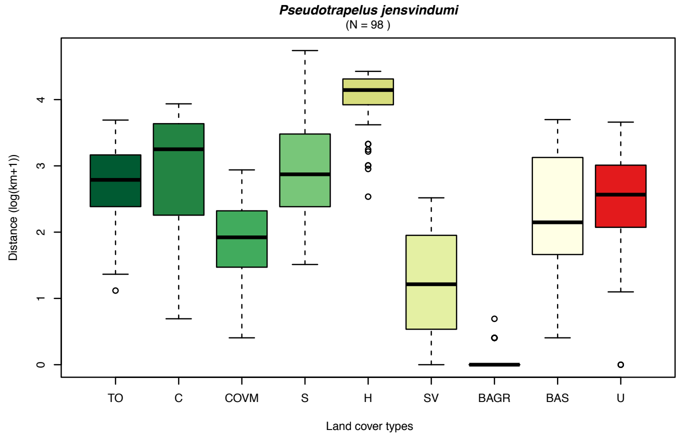

E

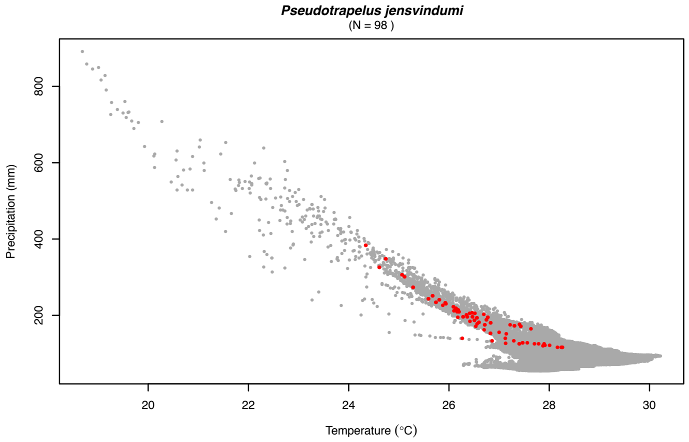

Trapelus flavimaculatus
Rüppell, 1835

S4: Species information
Lizards
Agamids, Agamidae

A

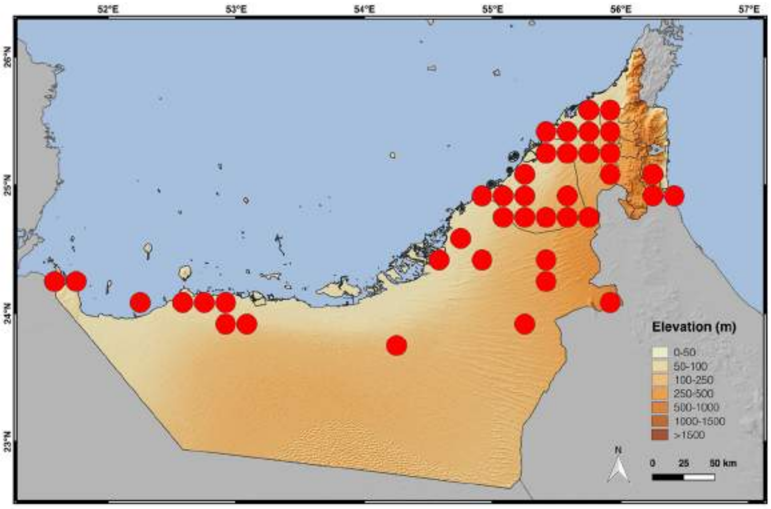

B

Useful information

IUCN Category	LC/LC
Venomous	NO
Endemic	NO
Origin	Native

C

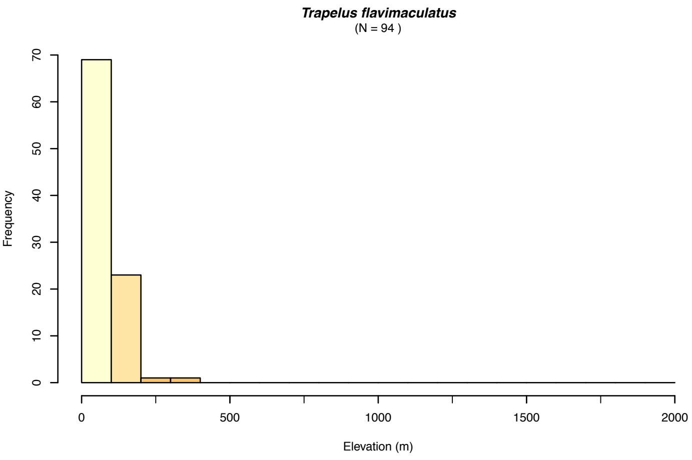

D

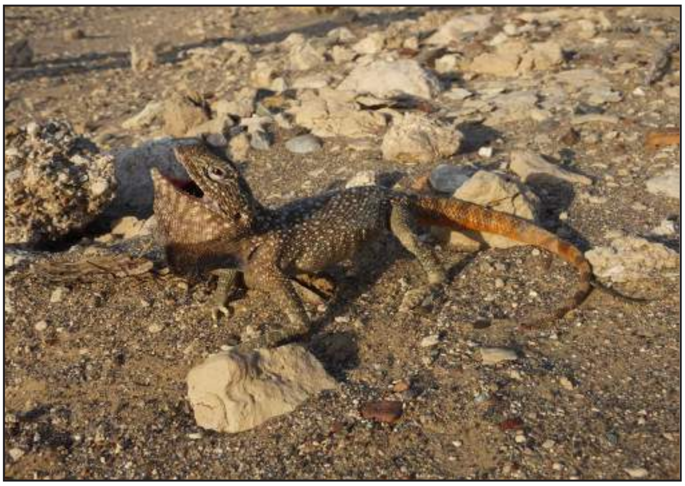

E

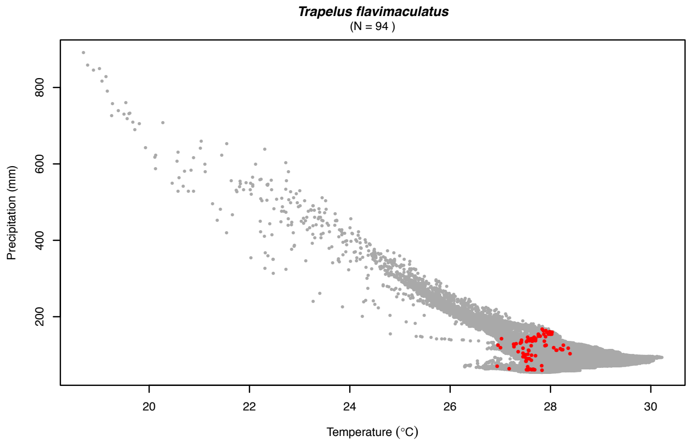

F

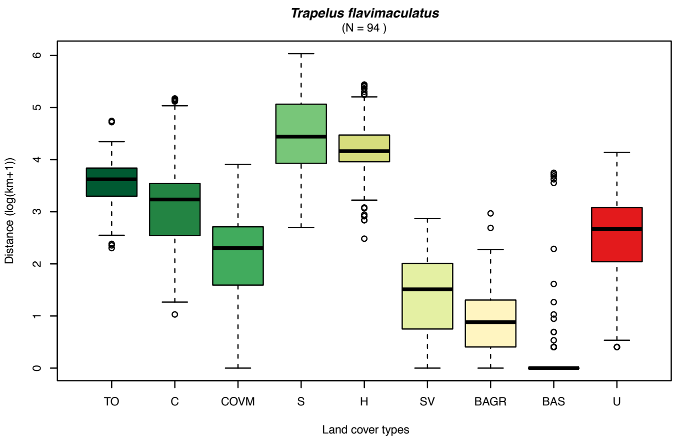

S5: Species information

Lizards

Agamids, Agamidae

Uromastyx aegyptia leptieni

Whilms and Böhme, 2000

B

Useful information

IUCN Category	VU/VU
Venomous	NO
Endemic	NO
Origin	Native

A

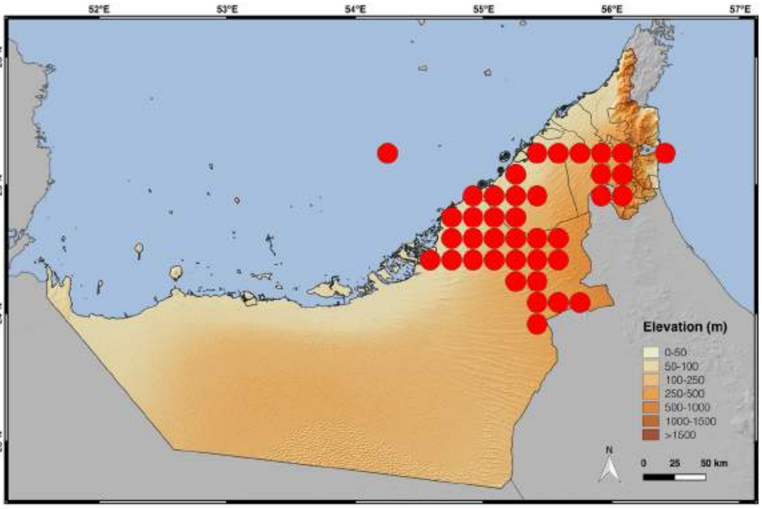

D

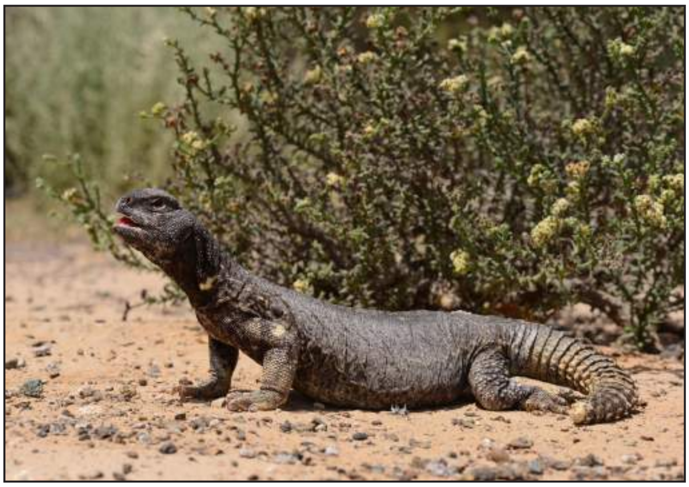

C

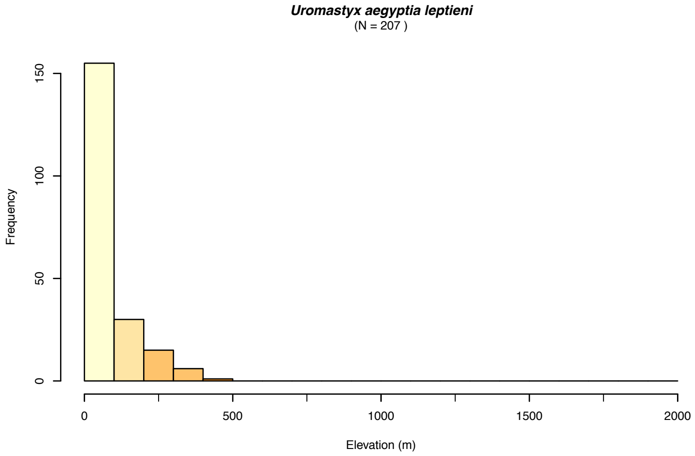

F

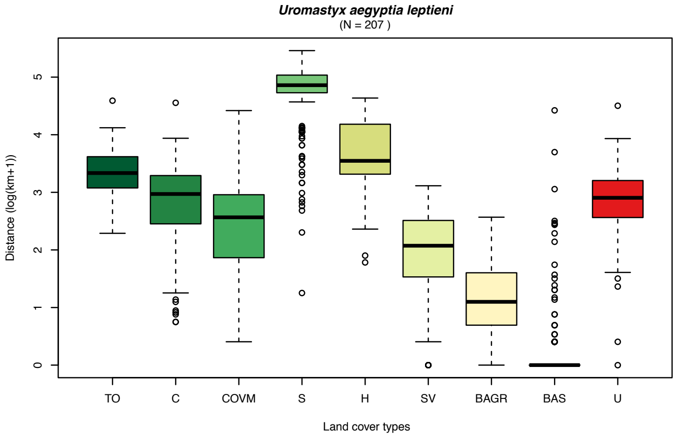

E

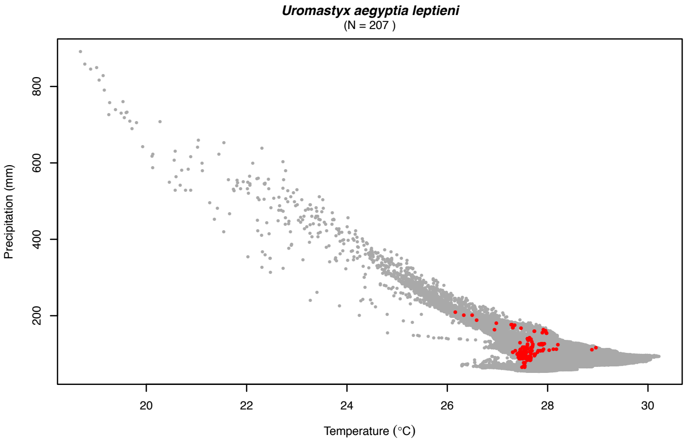

Uromastyx aegyptia microlepis
Arnold, 1980

S6: Species information
Lizards
Agamids, Agamidae

A

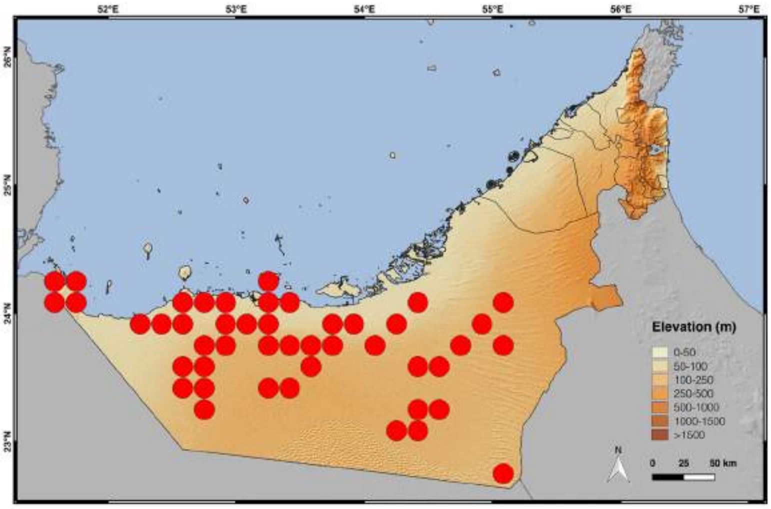

B

Useful information

IUCN Category	VU/VU
Venomous	NO
Endemic	NO
Origin	Native

C

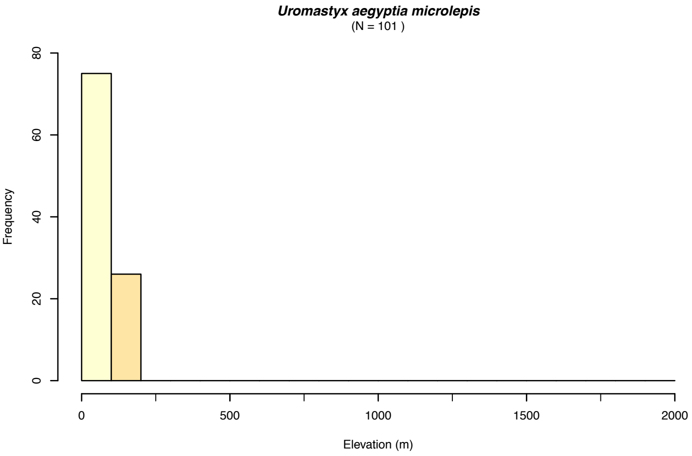

D

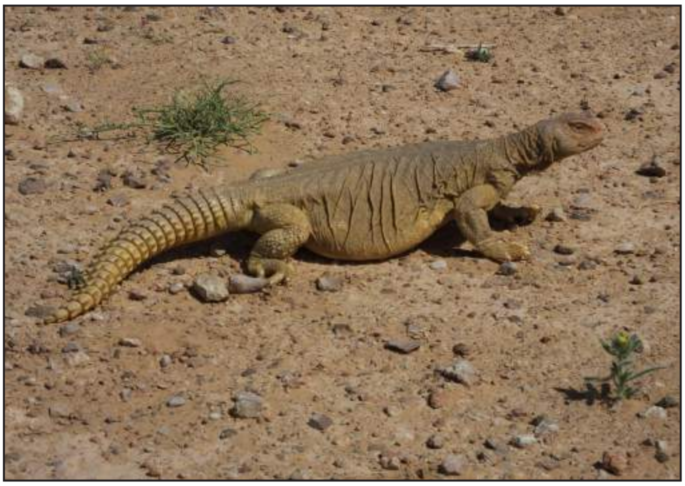

E

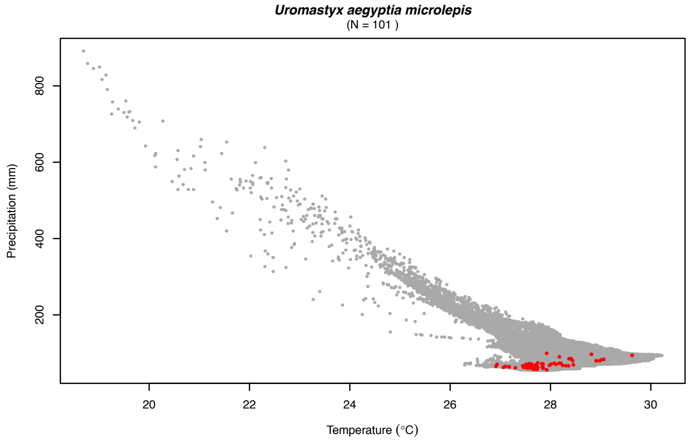

F

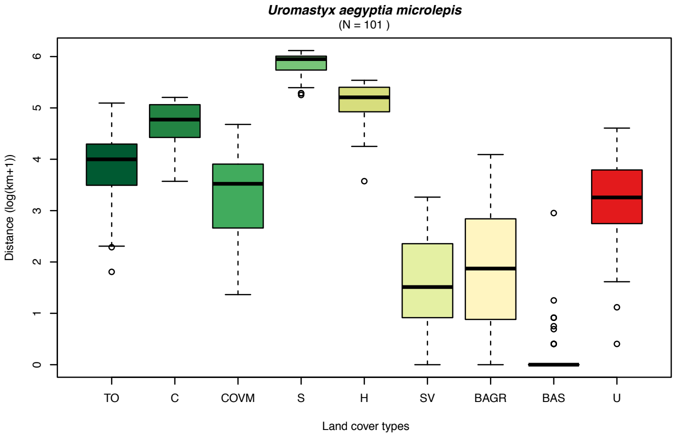

S7: Species information
Lizards
Geckos, Gekkonidae

Bunopus tuberculatus
Blanford, 1874

A

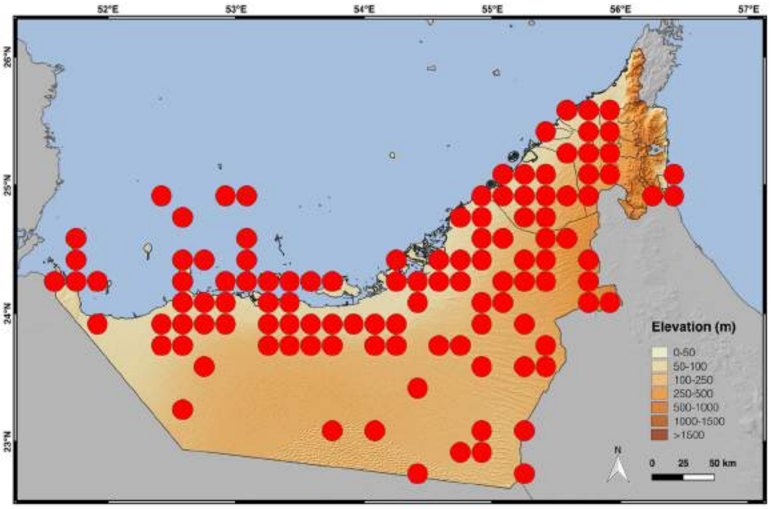

B

Useful information

IUCN Category	LC/LC
Venomous	NO
Endemic	NO
Origin	Native

D

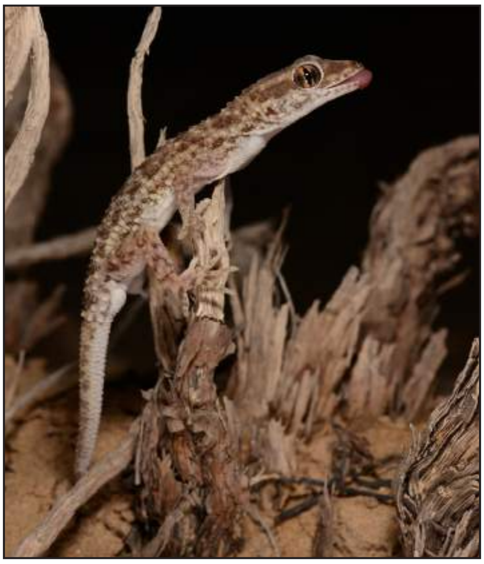

C

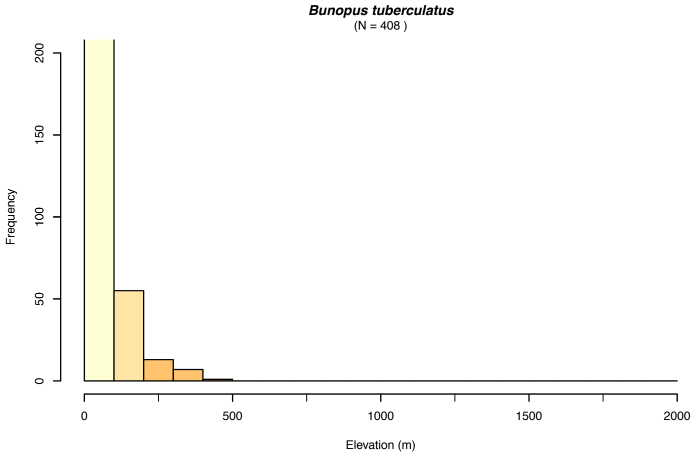

F

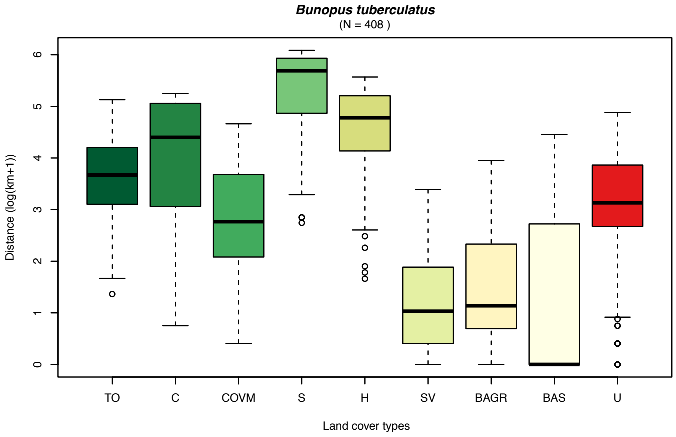

E

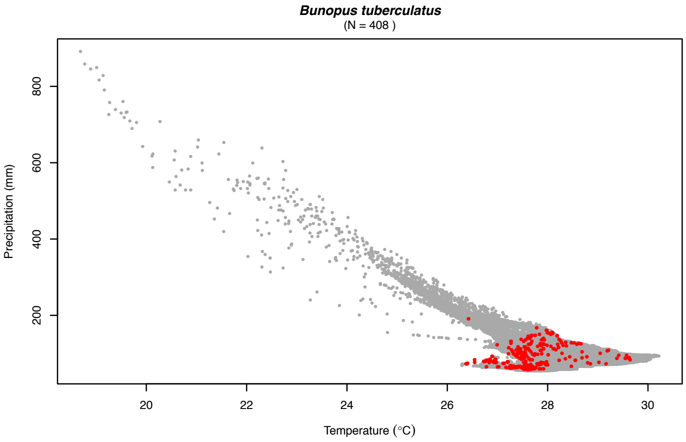

Cyrtopodion scabrum (Heyden, 1827)

S8: Species information

Lizards

Geckos, Gekkonidae

A

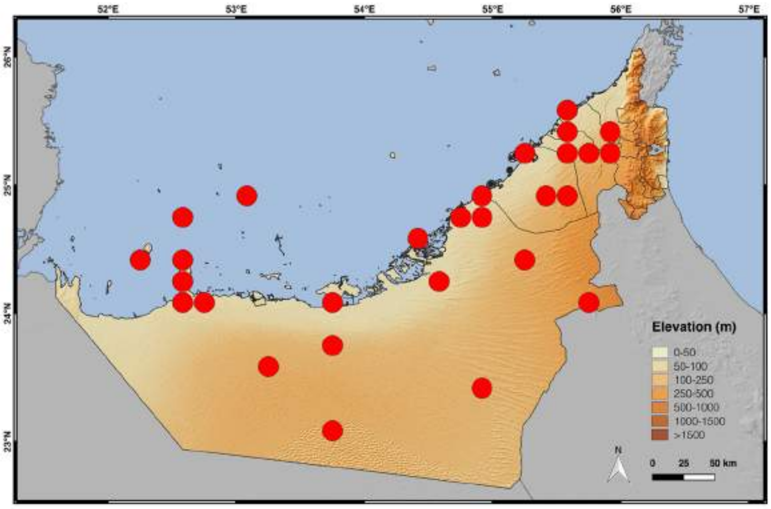

B

Useful information

IUCN Category	LC/LC
Venomous	NO
Endemic	NO
Origin	Native

C

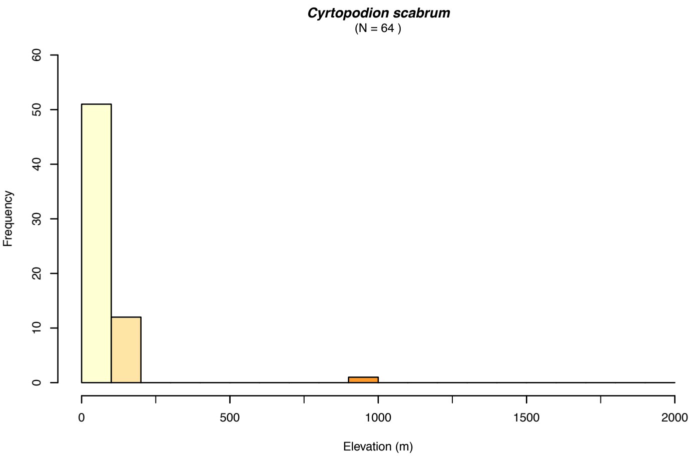

D

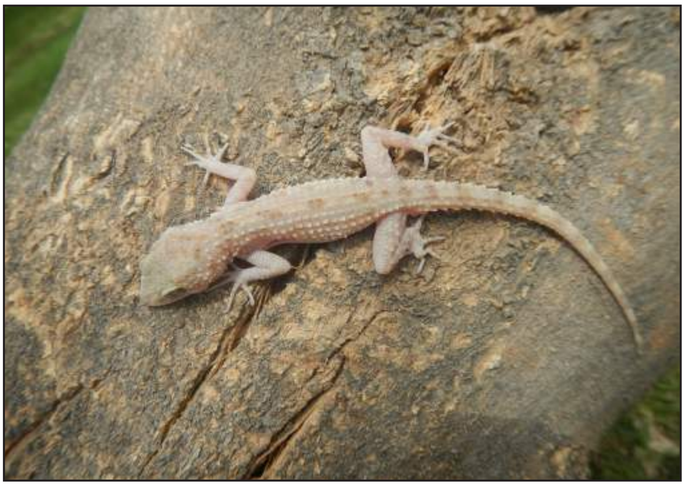

E

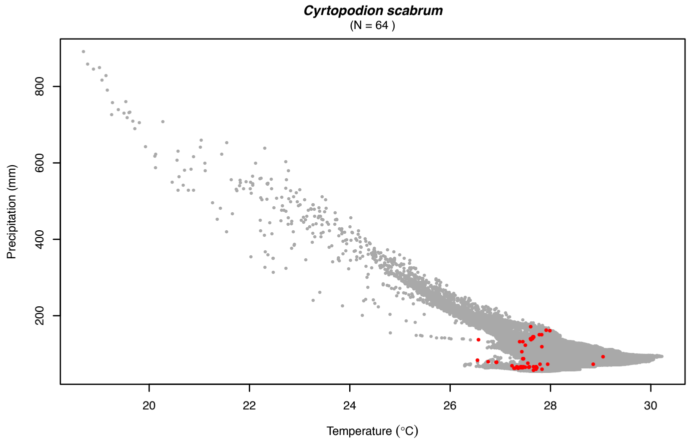

F

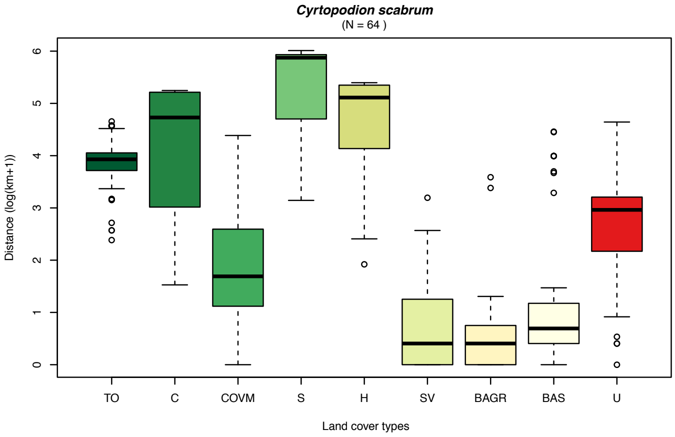

S9: Species information

Lizards

Geckos, Gekkonidae

Hemidactylus flaviviridis

Rüppell, 1835

A

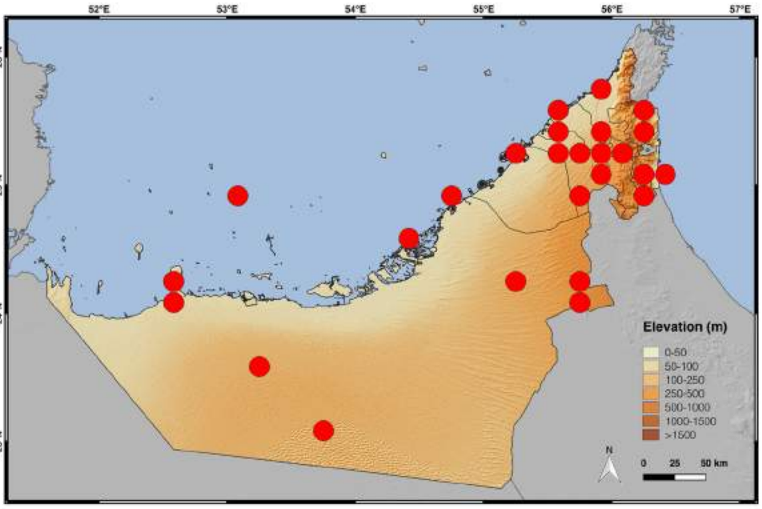

B

Useful information

IUCN Category	LC/NA
Venomous	NO
Endemic	NO
Origin	Introduced

D

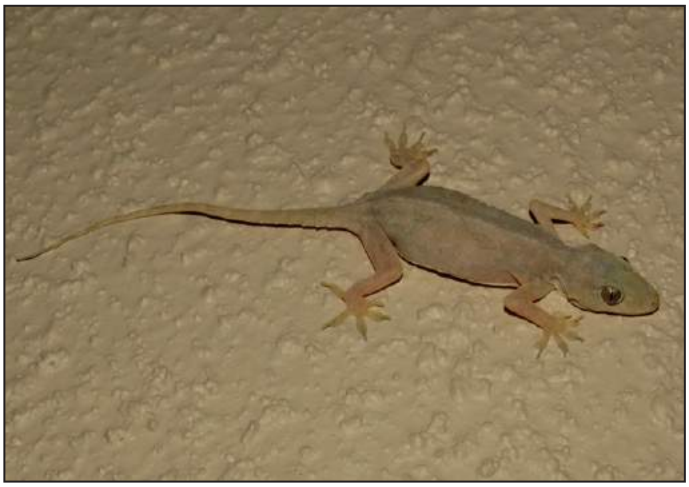

C

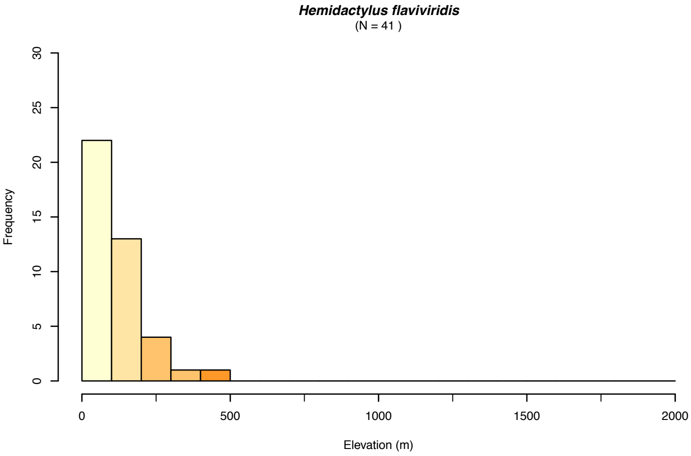

F

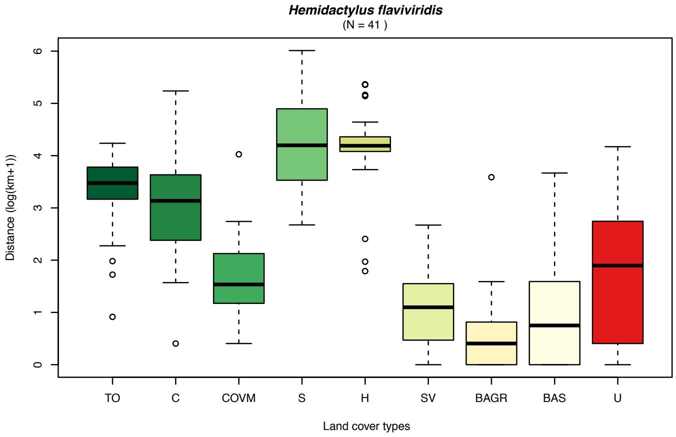

E

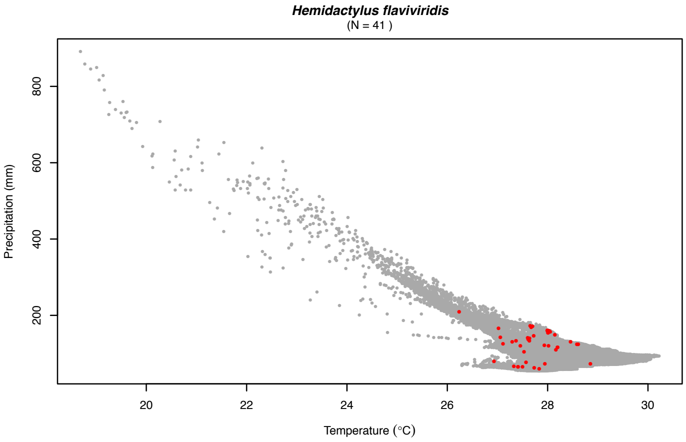

Hemidactylus persicus
Anderson, 1872

S10: Species information
Lizards
Geckos, Gekkonidae

A

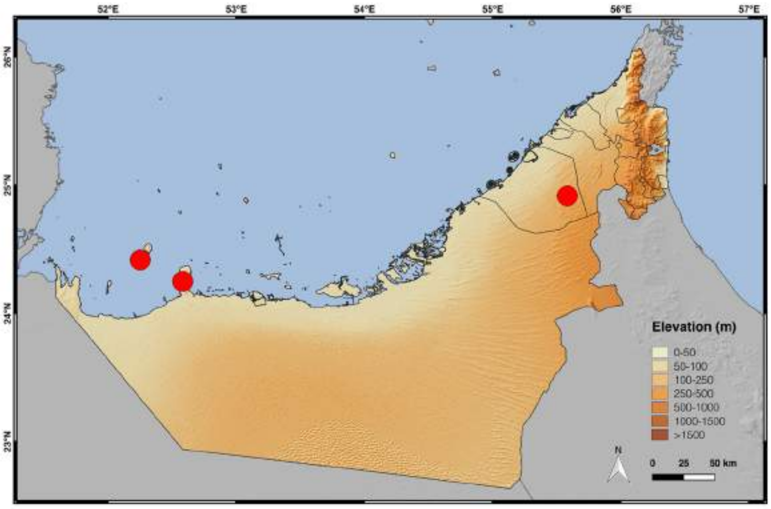

B

Useful information

IUCN Category	LC/VU
Venomous	NO
Endemic	NO
Origin	Native

C

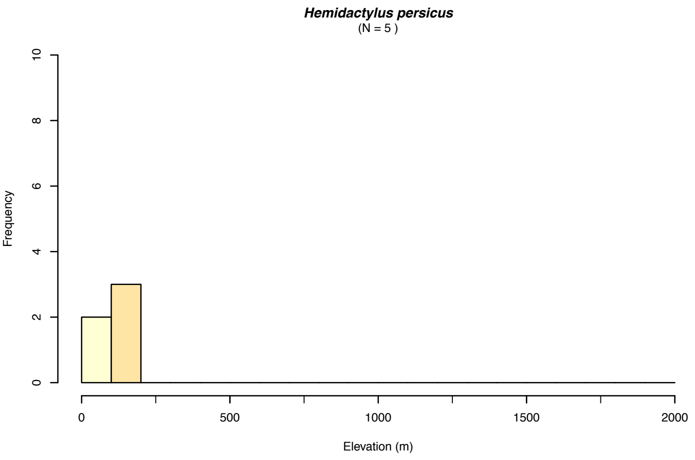

D

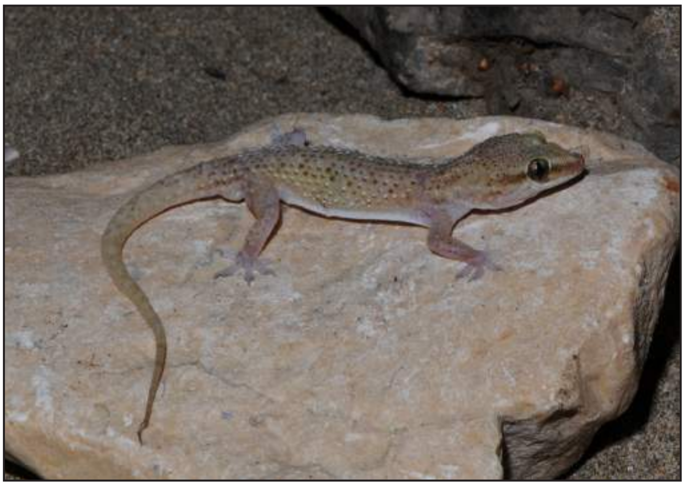

E

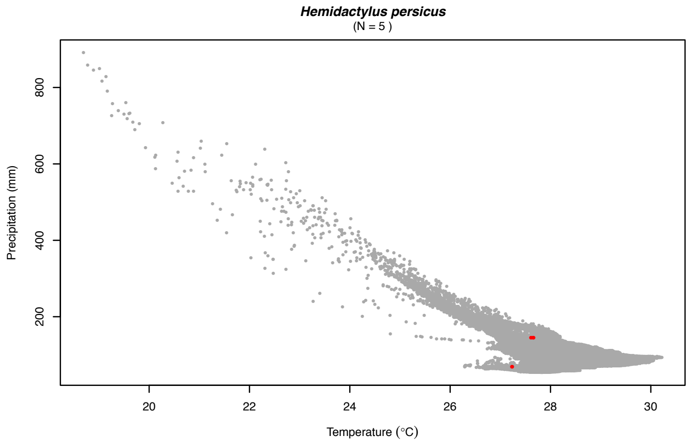

F

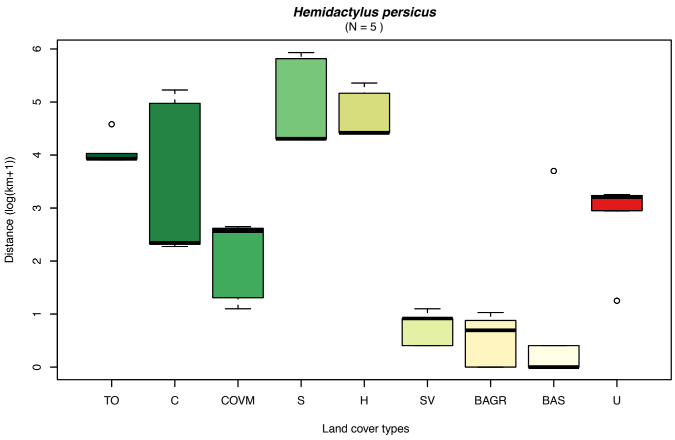

S11: Species information

Lizards

Geckos, Gekkonidae

Hemidactylus robustus

Heyden, 1827

A

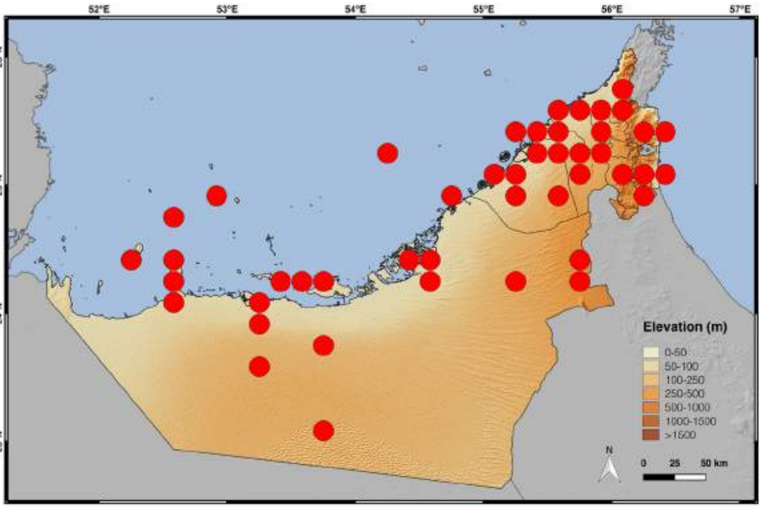

B

Useful information	
IUCN Category	LC/LC
Venomous	NO
Endemic	NO
Origin	Native

D

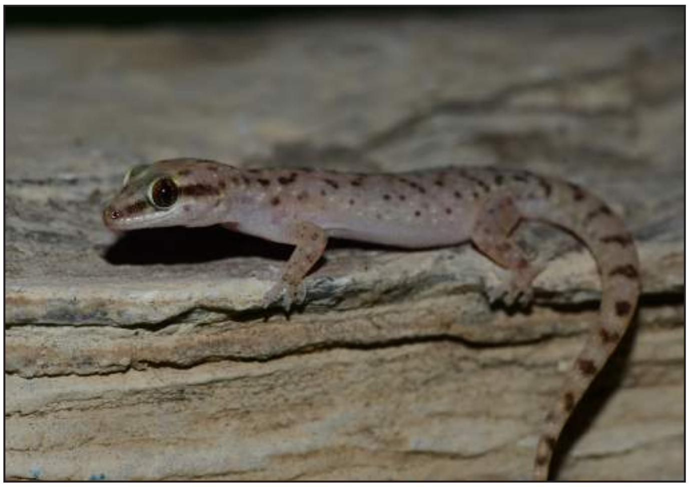

C

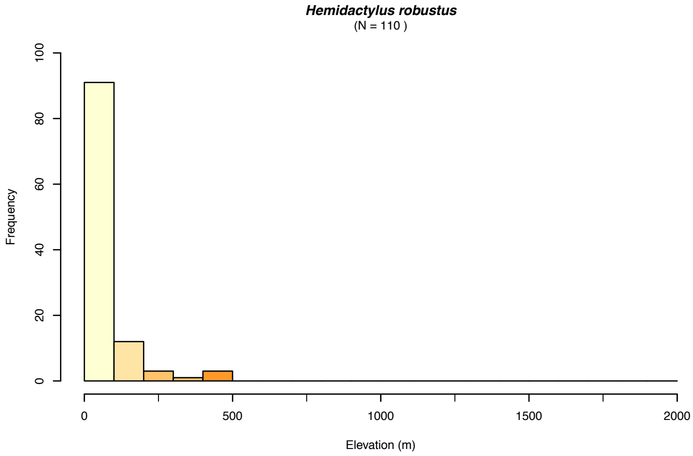

F

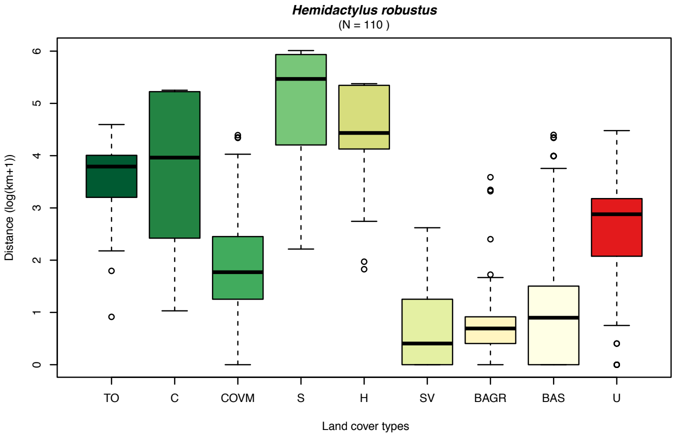

E

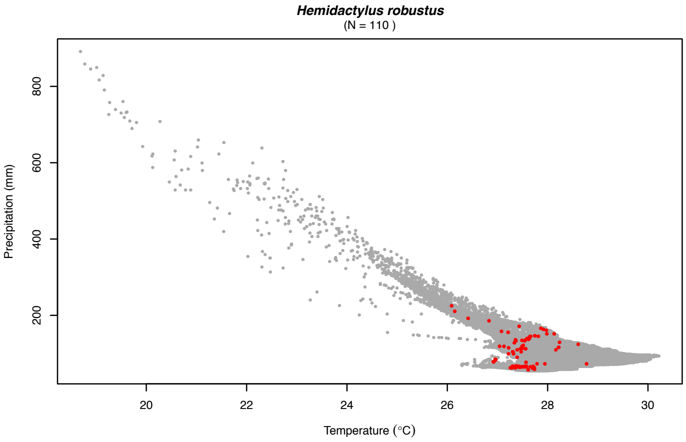

Lizards
Agamids
Geckos
Lacertids
Skinkis
Amphisbaenids
Varanids
Snakes
Boidae
Colubridae
Lamprophiidae
Leptotyphlopidae
Typhlopidae
Viperidae

Pseudoceramodactylus khobarensis Haas, 1957

S12: Species information

Lizards

Geckos, Gekkonidae

A

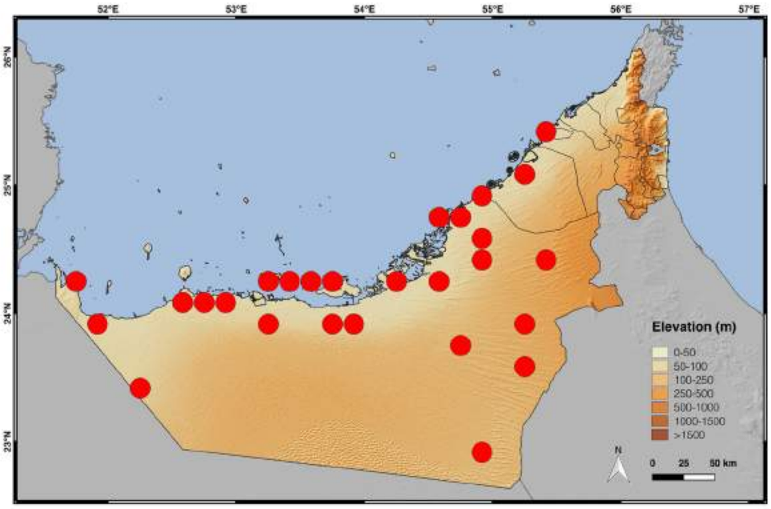

B

Useful information

IUCN Category	LC/LC
Venomous	NO
Endemic	NO
Origin	Native

C

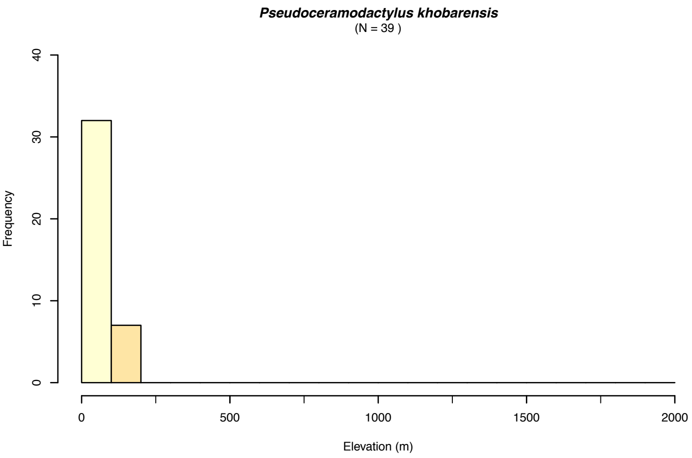

D

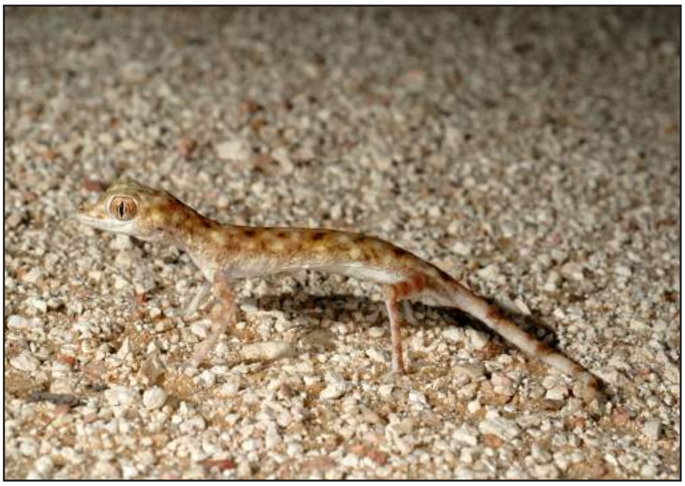

E

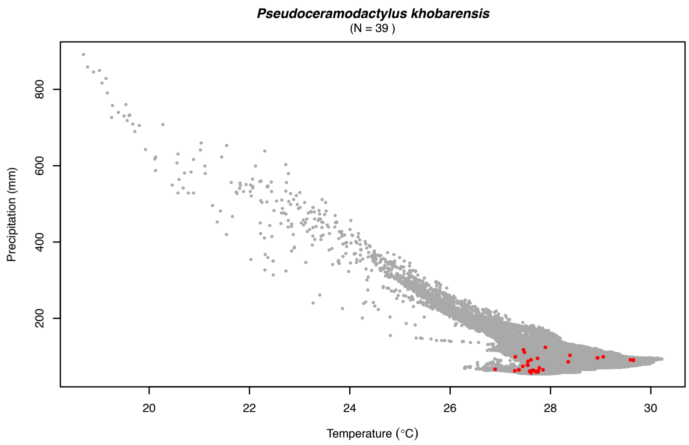

F

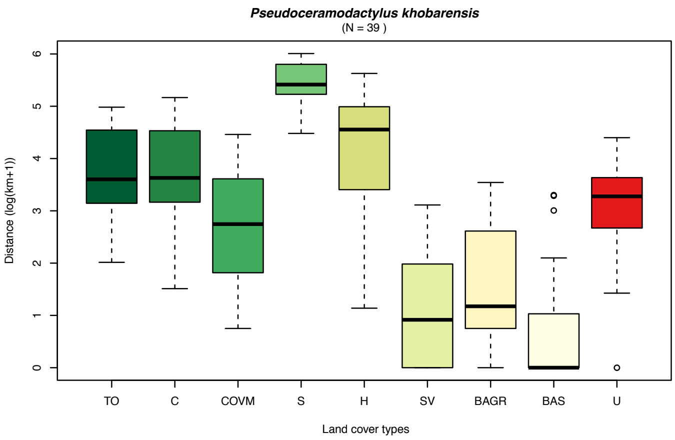

S13: Species information

Lizards

Geckos, Gekkonidae

Stenodactylus arabicus (Haas, 1957)

A

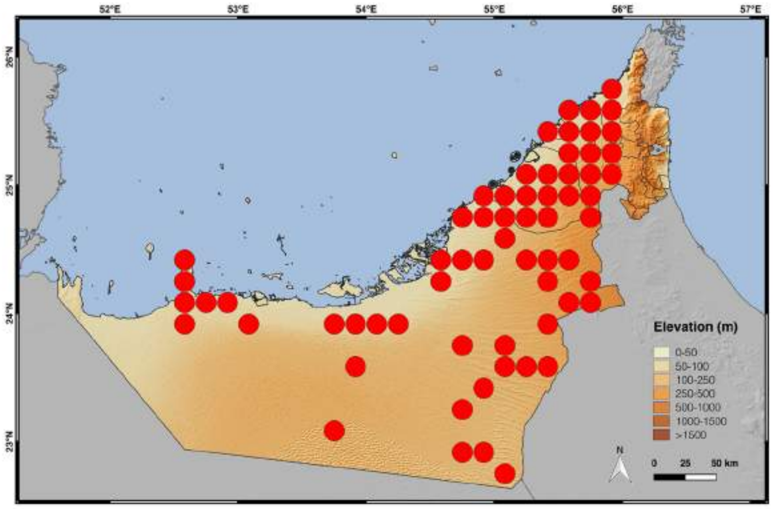

B

Useful information	
IUCN Category	LC/LC
Venomous	NO
Endemic	NO
Origin	Native

D

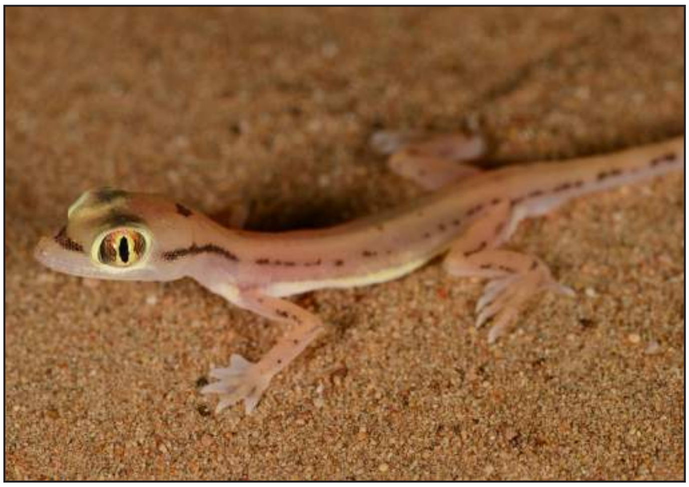

C

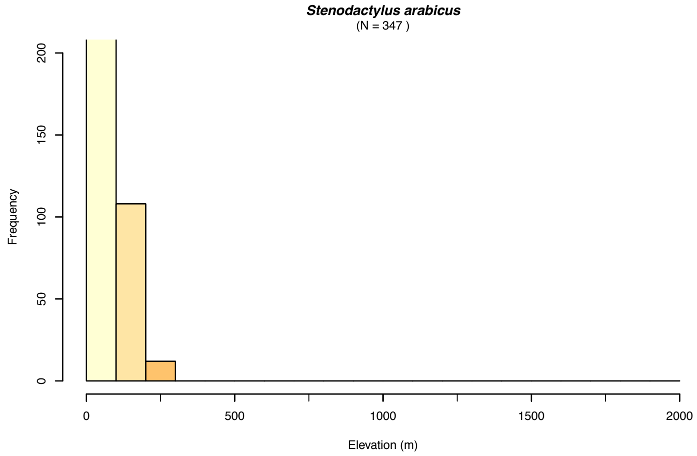

F

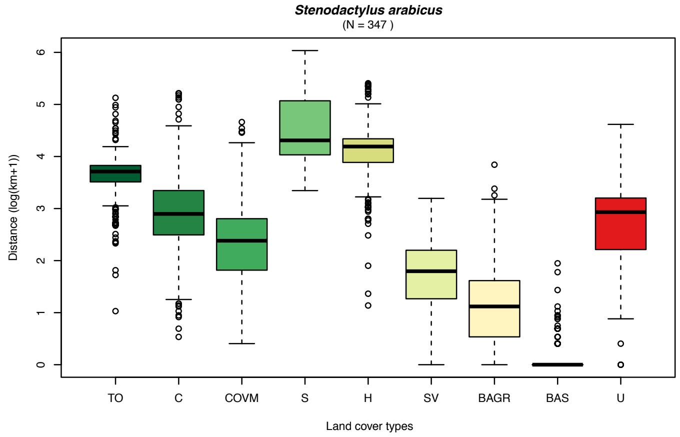

E

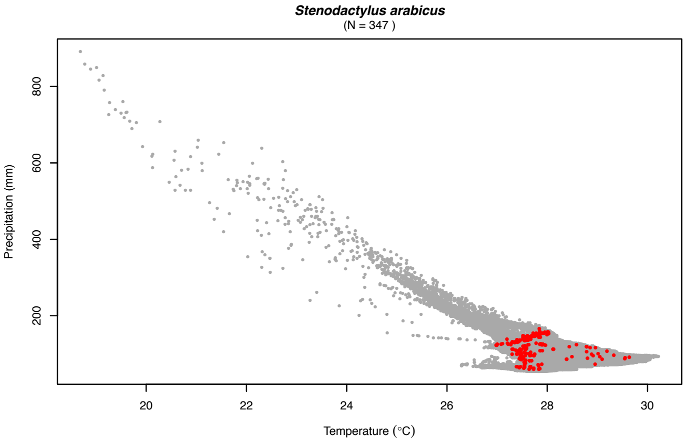

Stenodactylus doriae (Blanford, 1874)

S14: Species information

Lizards

Geckos, Gekkonidae

A

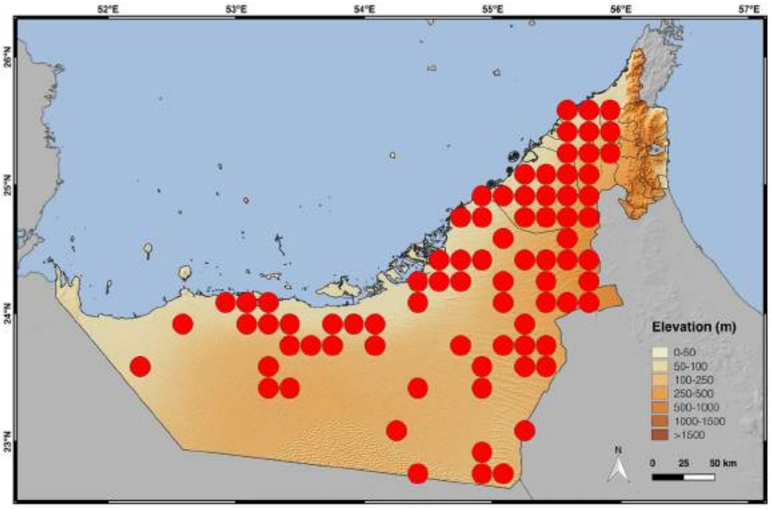

B

Useful information

IUCN Category	LC/LC
Venomous	NO
Endemic	NO
Origin	Native

C

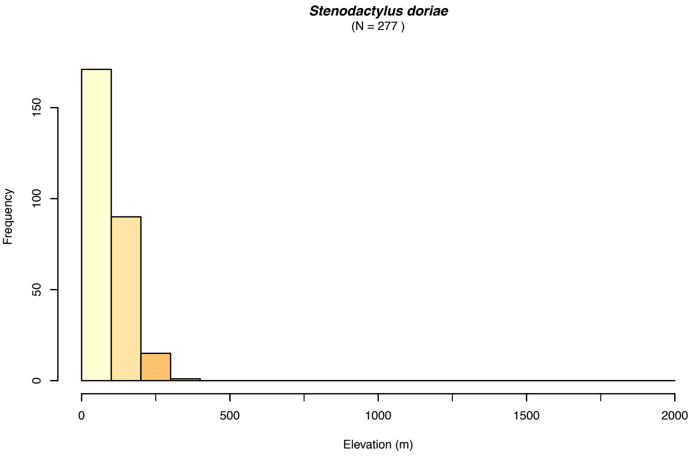

D

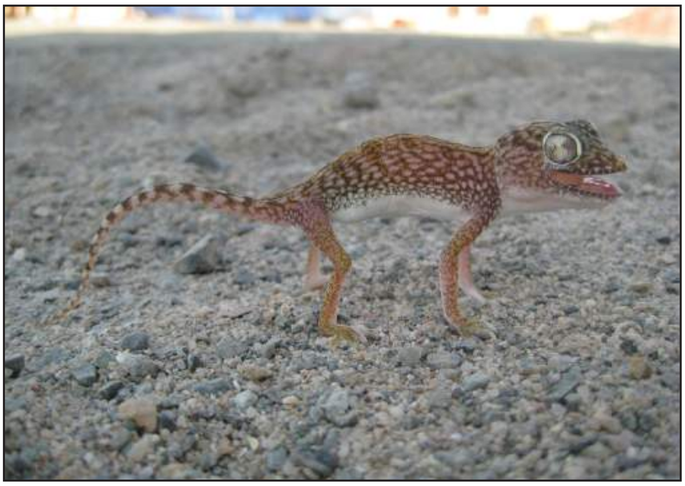

E

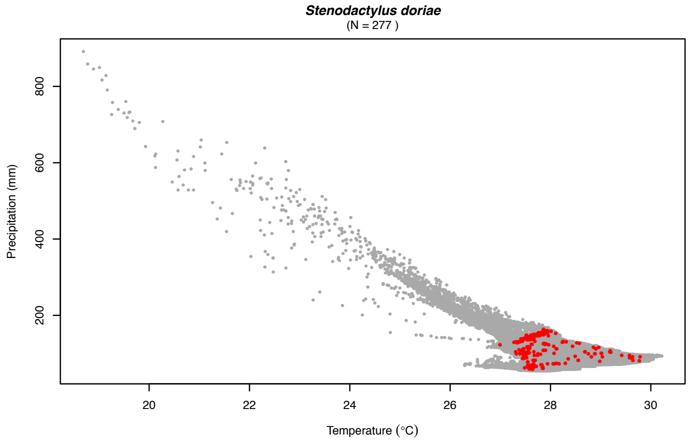

F

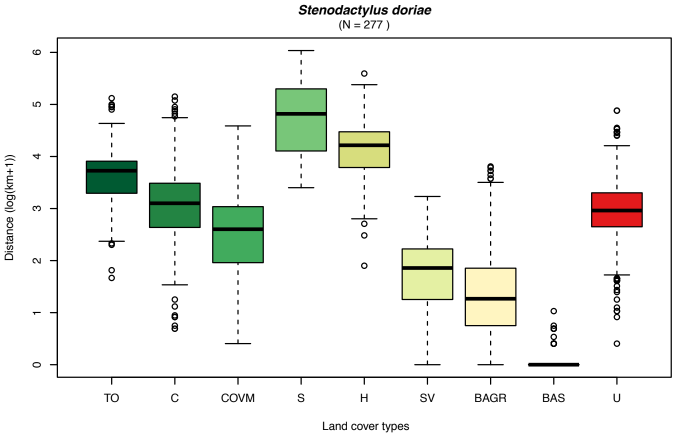

S15: Species information

Lizards

Geckos, Gekkonidae

Stenodactylus leptocosymbotes

Leviton & Anderson, 1967

A

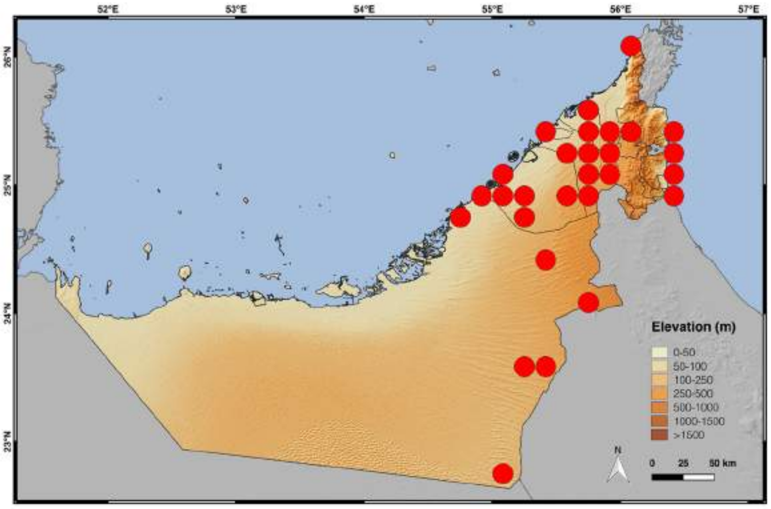

B

Useful information	
IUCN Category	LC/LC
Venomous	NO
Endemic	NO
Origin	Native

D

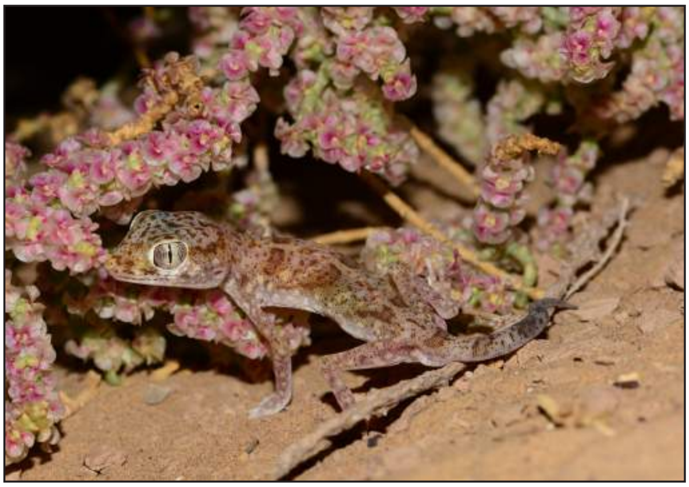

C

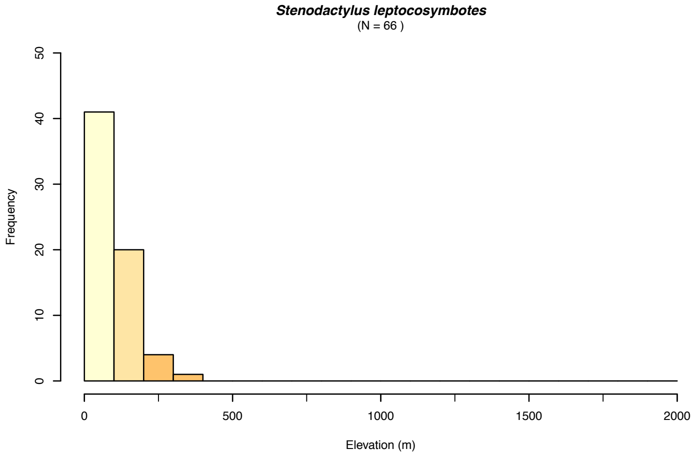

F

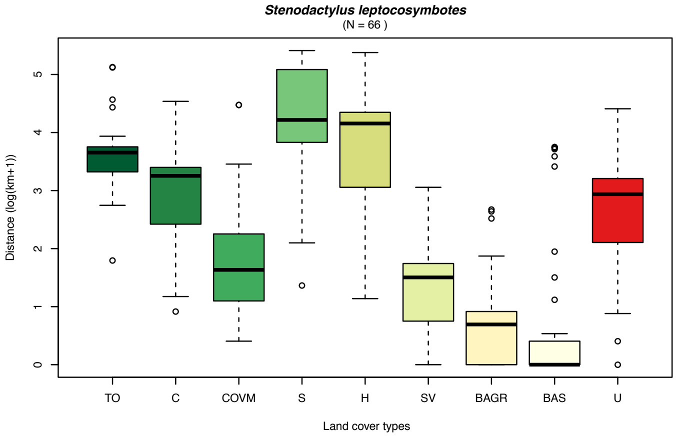

E

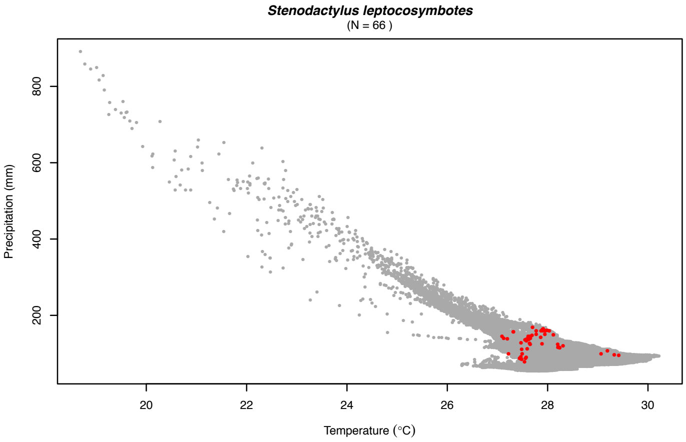

Stenodactylus slevini
Haas, 1957

S16: Species information
Lizards
Geckos, Gekkonidae

A

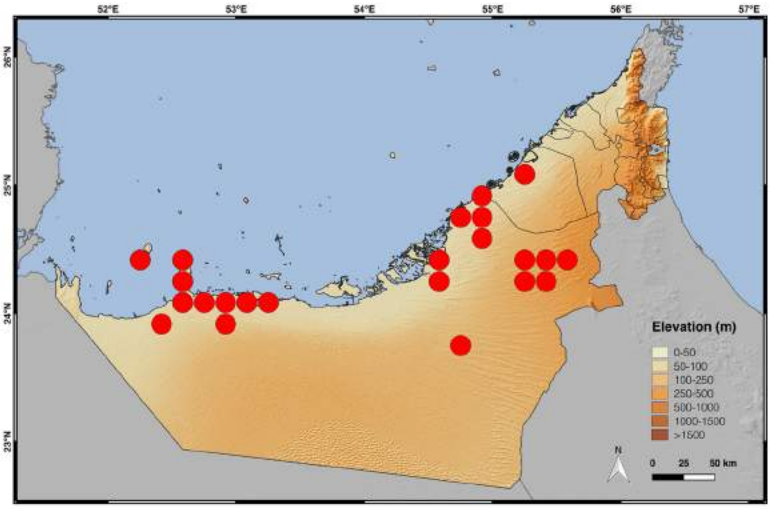

B

Useful information

IUCN Category	LC/LC
Venomous	NO
Endemic	NO
Origin	Native

C

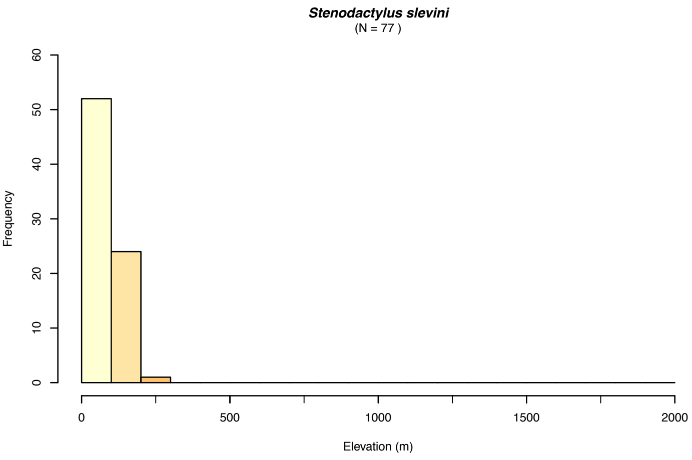

D

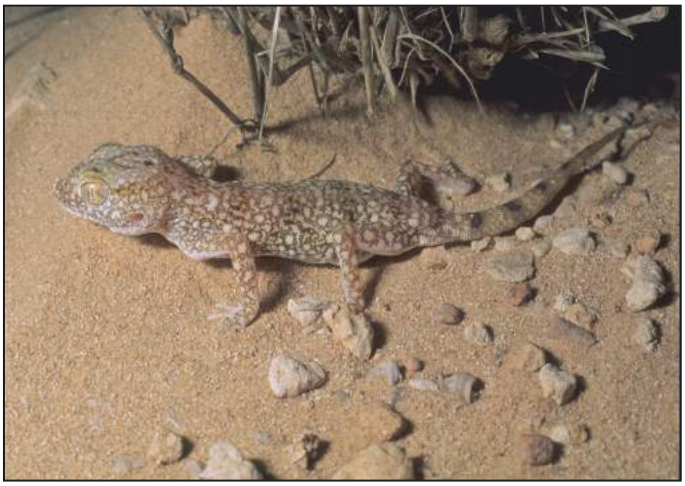

E

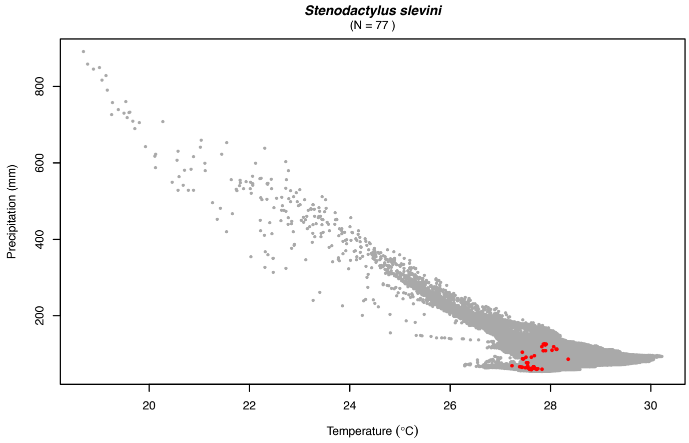

F

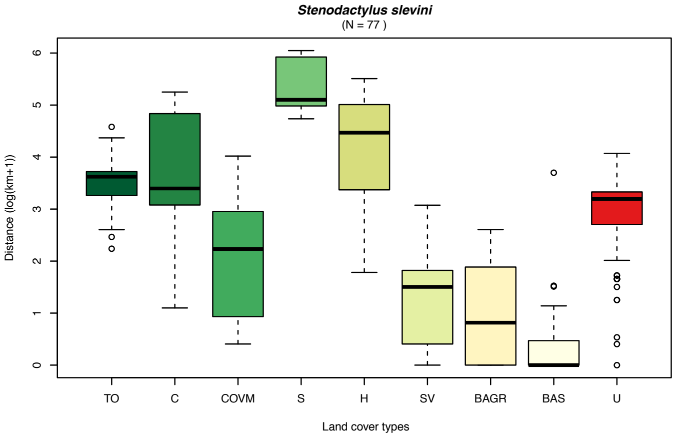

S17: Species information

Lizards

Geckos, Gekkonidae

Trachydactylus hajarensis (Arnold, 1980)

A

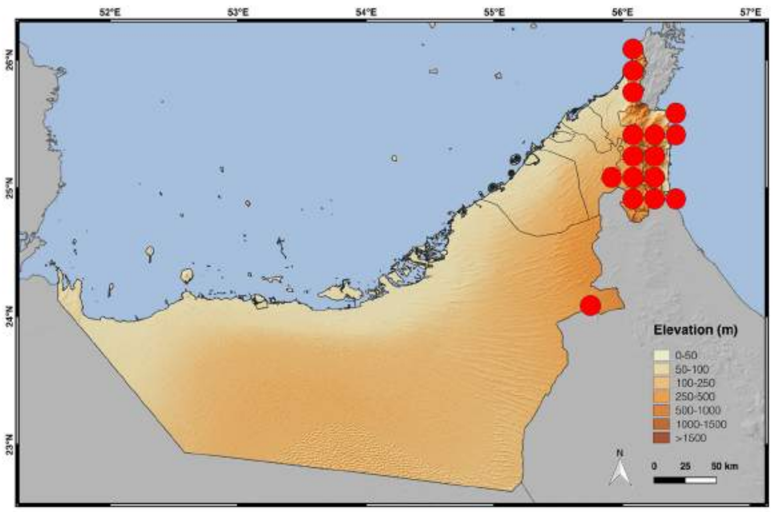

B

Useful information	
IUCN Category	NE/LC
Venomous	NO
Endemic	NO
Origin	Native

D

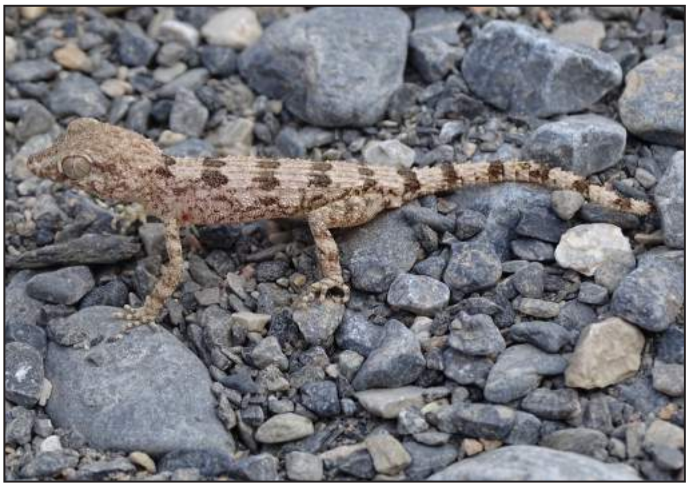

C

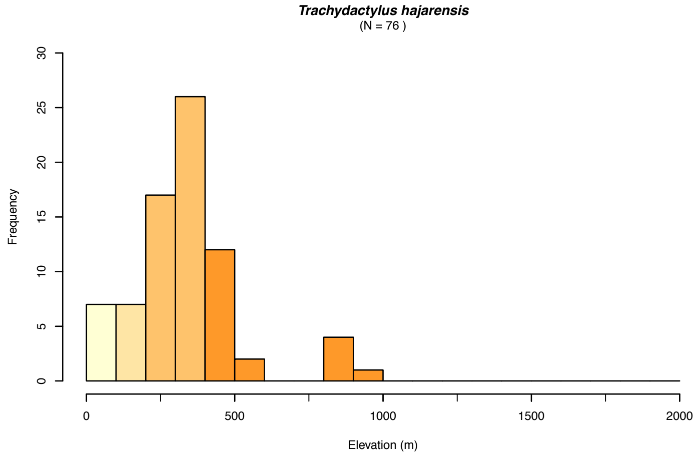

F

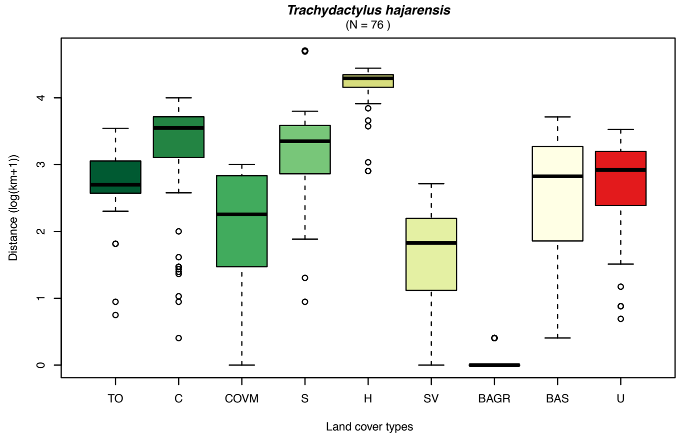

E

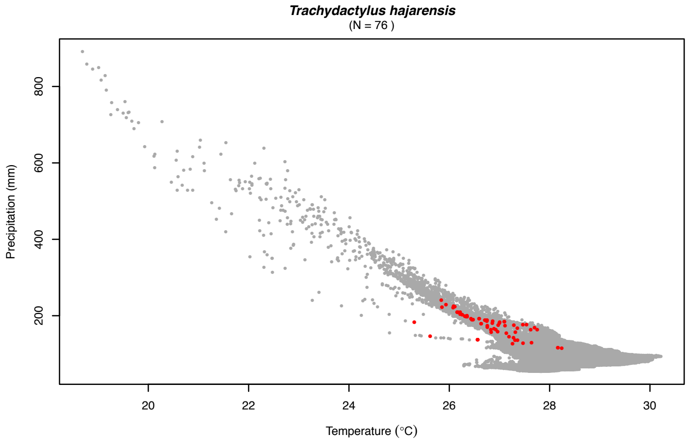

Asaccus caudivolvulus
Arnold & Gardner, 1994

S18: Species information
Lizards
Geckos, Phyllodactylidae

A

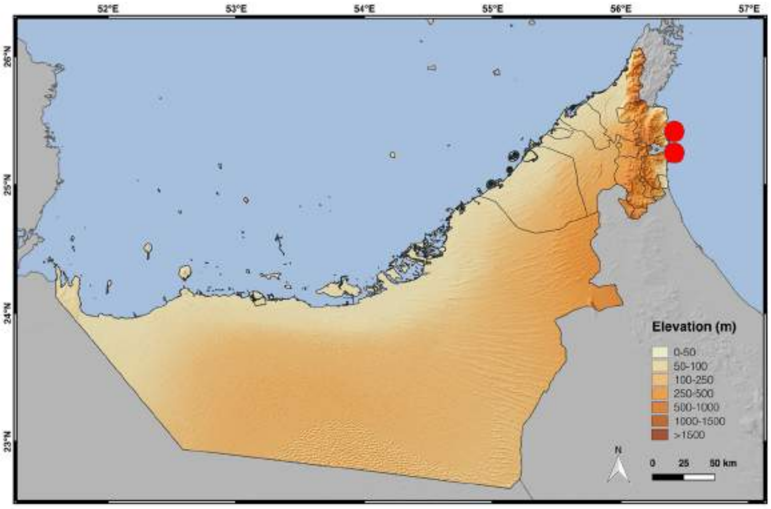

B

Useful information

IUCN Category	LC/CR
Venomous	NO
Endemic	YES
Origin	Native

C

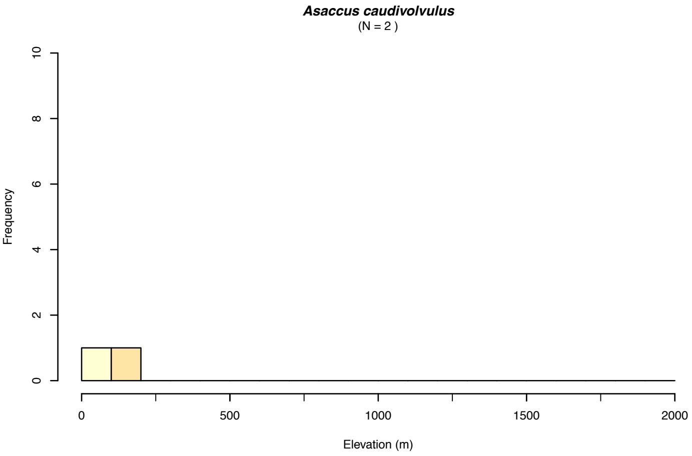

D

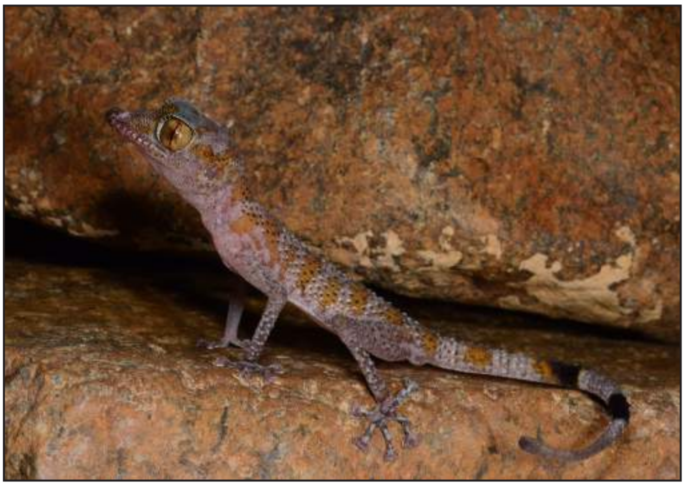

E

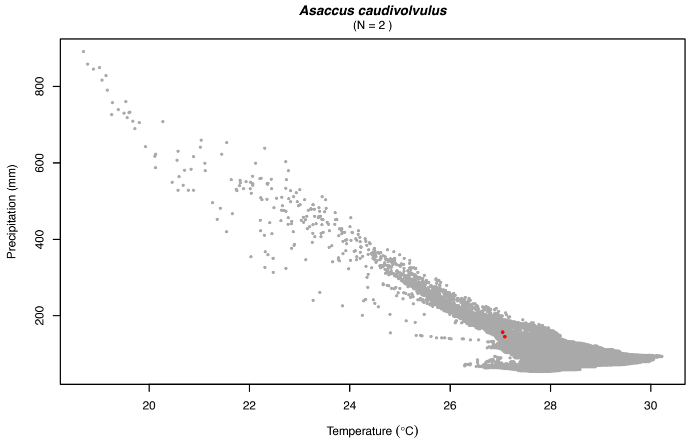

F

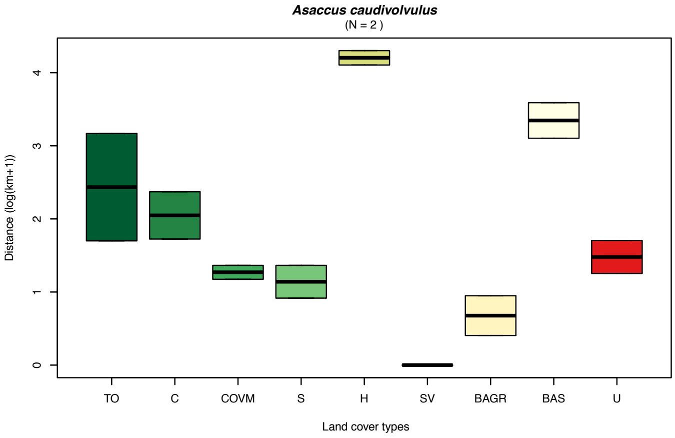

Lizards
Agamids
Geckos
Lacertids
Skinkis
Amphisbaenids
Varanids
Snakes
Boidae
Colubridae
Lamprophiidae
Leptotyphlopidae
Typhlopidae
Viperidae

S19: Species information

Lizards

Geckos, Phyllodactylidae

Asaccus gallagheri (Arnold, 1972)

A

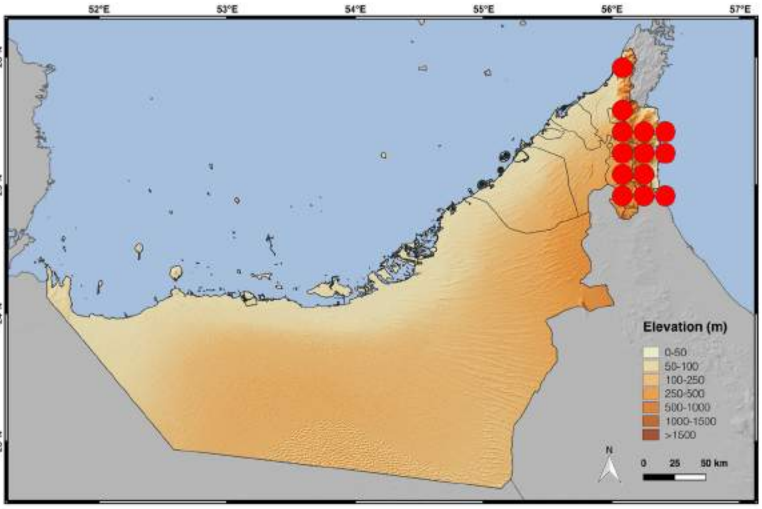

B

Useful information

IUCN Category	LC/LC
Venomous	NO
Endemic	NO
Origin	Native

D

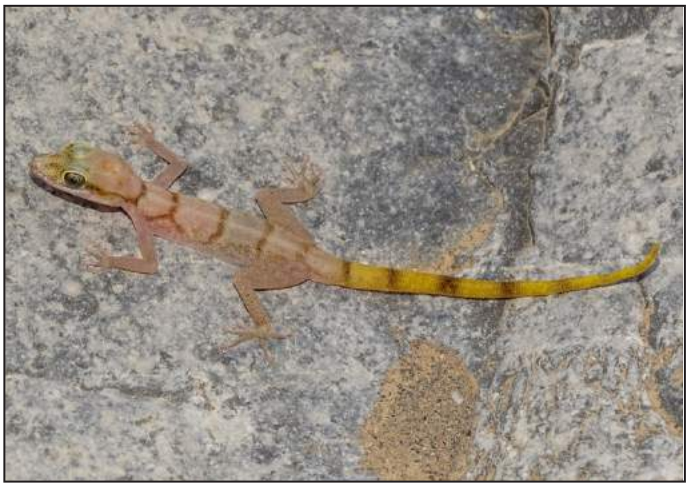

C

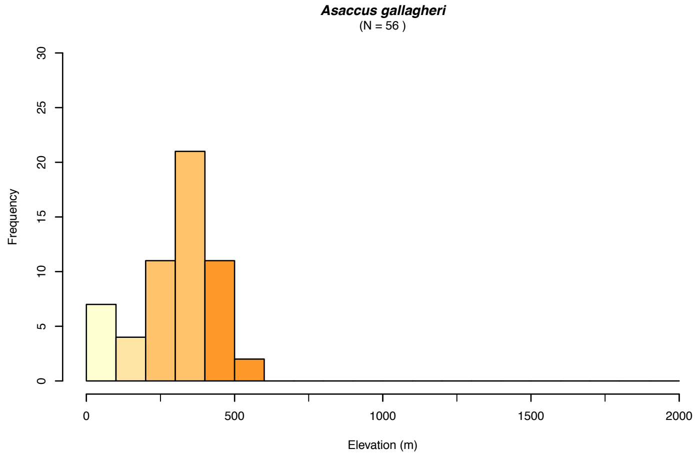

F

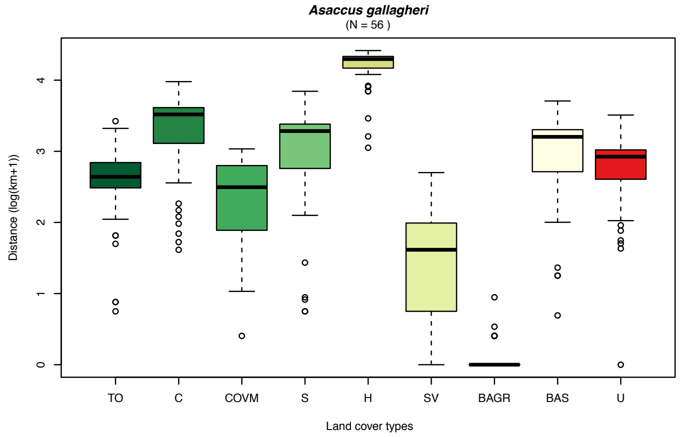

E

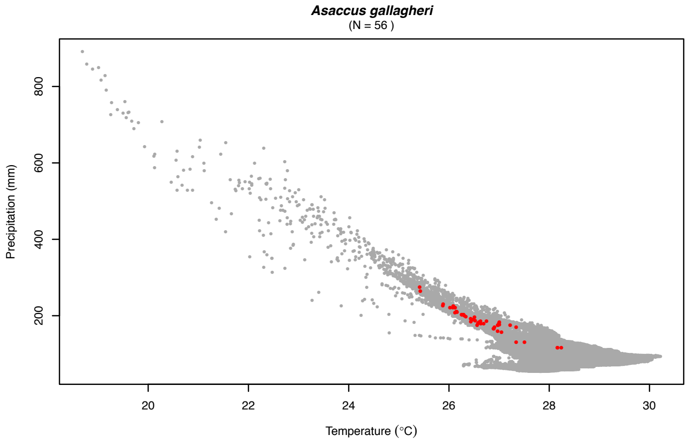

Asaccus gardneri
Carranza, Simó-Riudalbas, Jayasinghe, Wilms & Els, 2016

S20: Species information
Lizards
Geckos, Phyllodactylidae

A

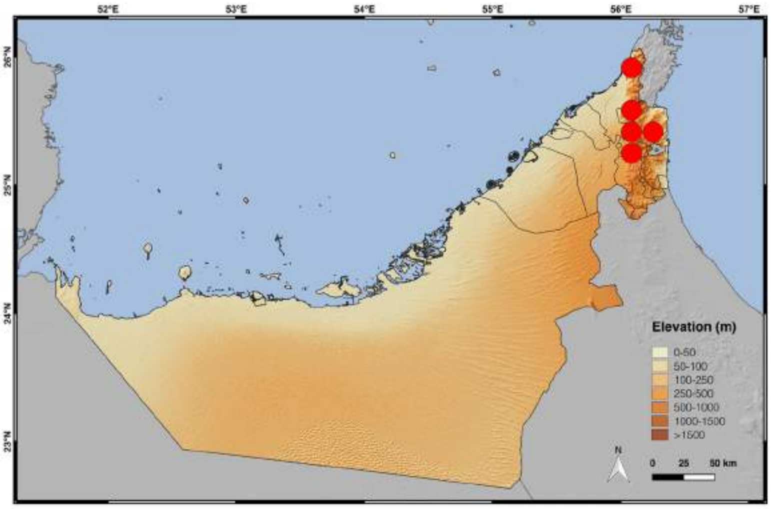

B

Useful information

IUCN Category	NE/LC
Venomous	NO
Endemic	NO
Origin	Native

C

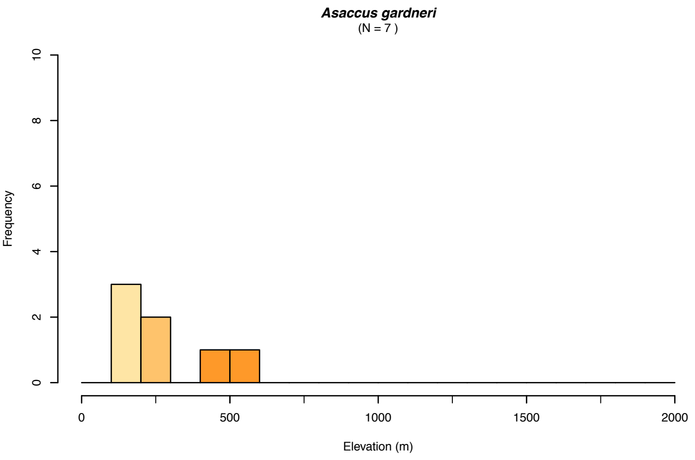

D

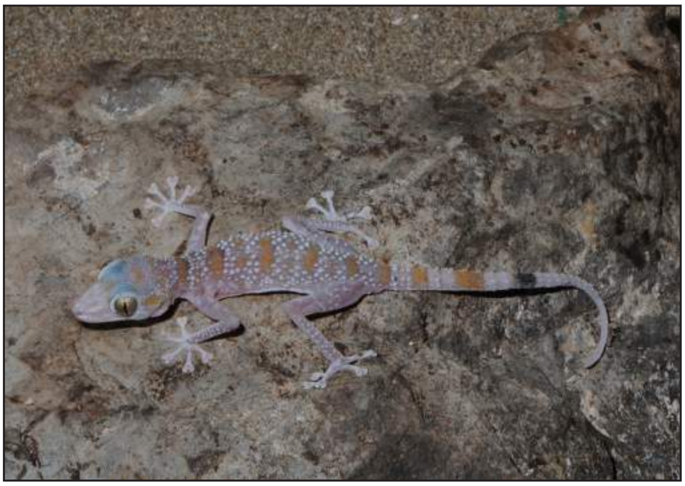

E

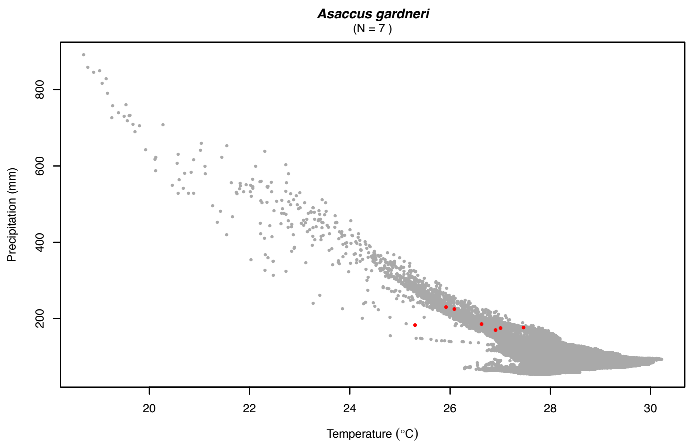

F

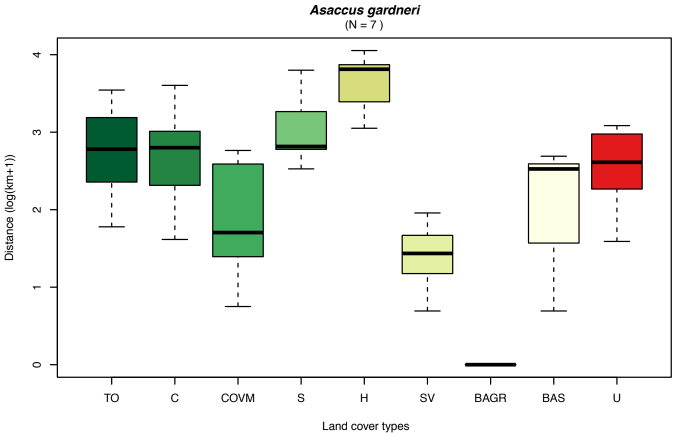

S21: Species information

Lizards

Geckos, Phyllodactylidae

Asaccus margaritae

Carranza, Simó-Riudalbas, Jayasinghe, Wilms & Els, 2016

A

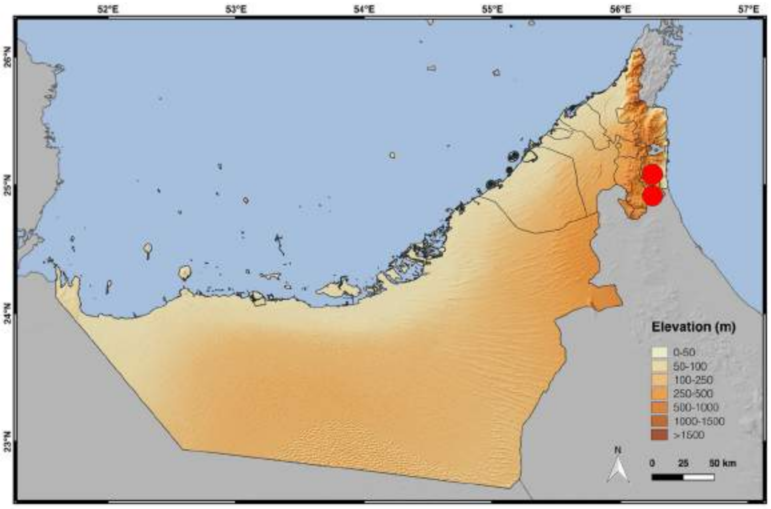

B

Useful information	
IUCN Category	NE/VU
Venomous	NO
Endemic	NO
Origin	Native

D

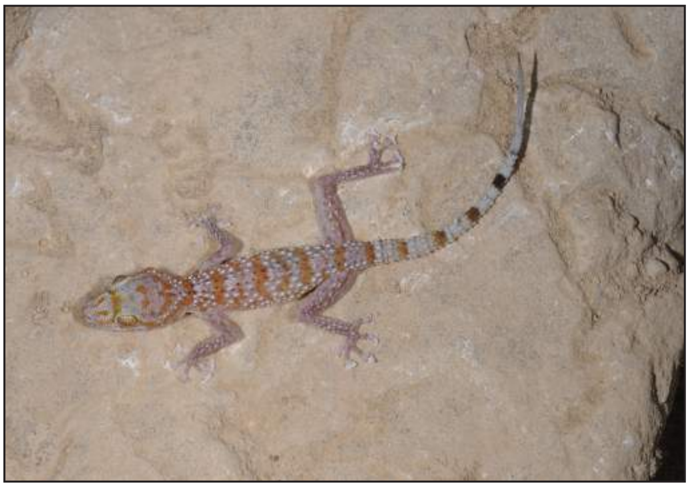

C

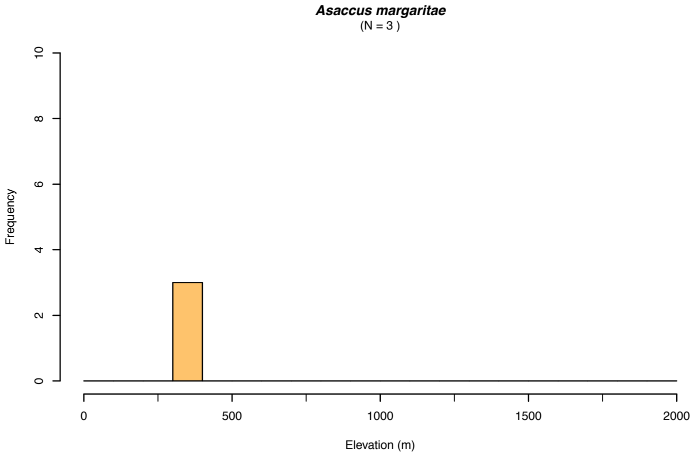

F

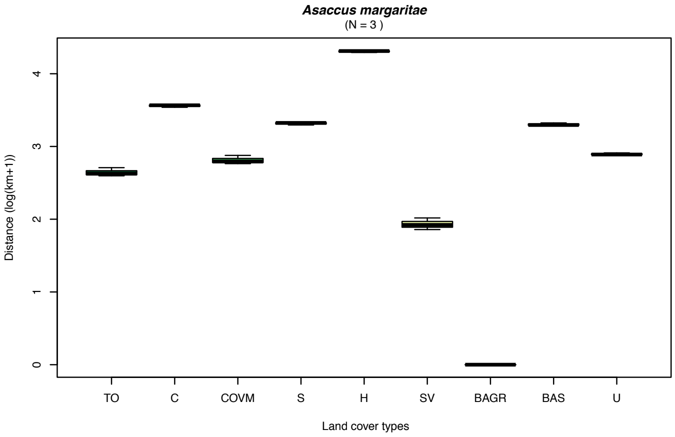

E

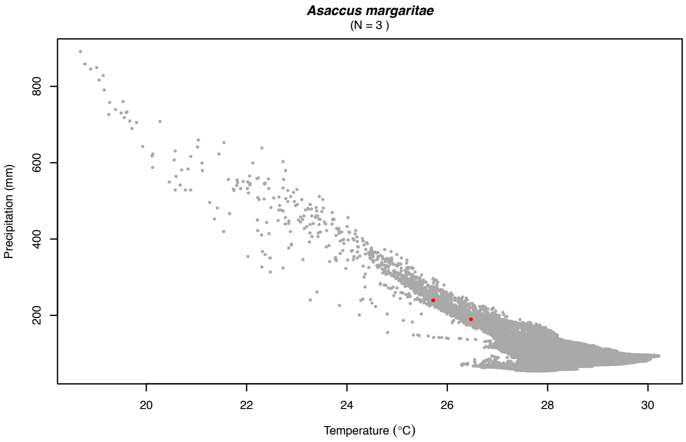

Ptyodactylus orlovi
Nazarov, Melnikov & Melnikova, 2013

S22: Species information
Lizards
Geckos, Phyllodactylidae

A

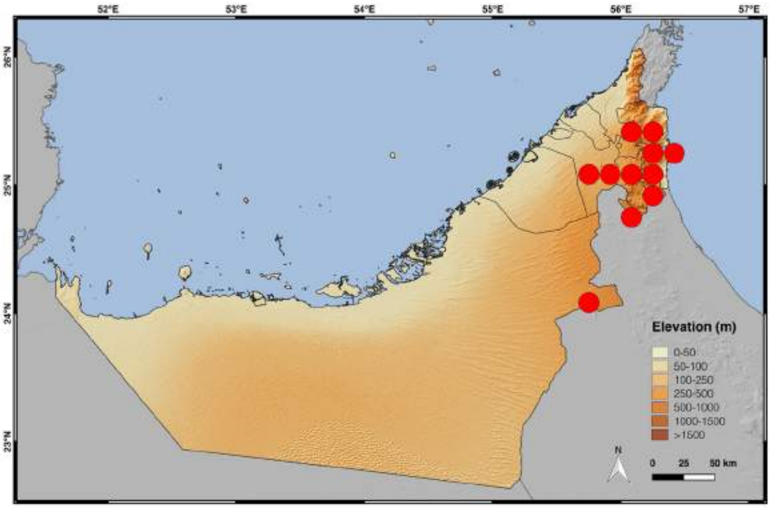

B

Useful information

IUCN Category	NE/LC
Venomous	NO
Endemic	NO
Origin	Native

C

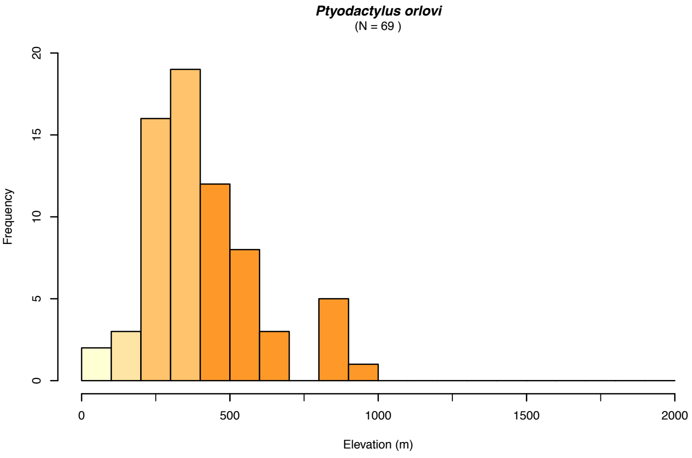

D

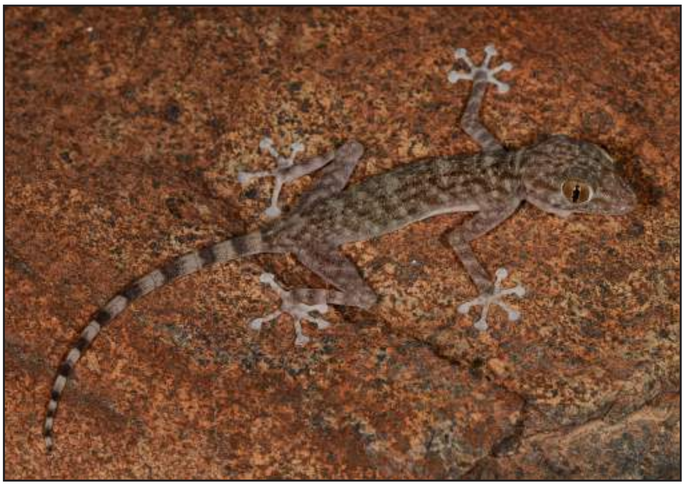

E

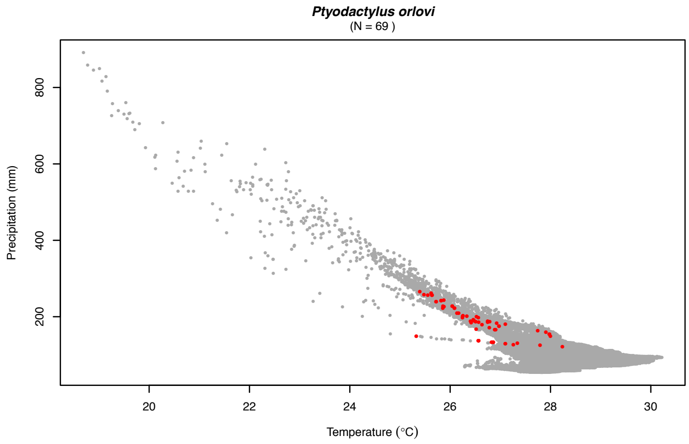

F

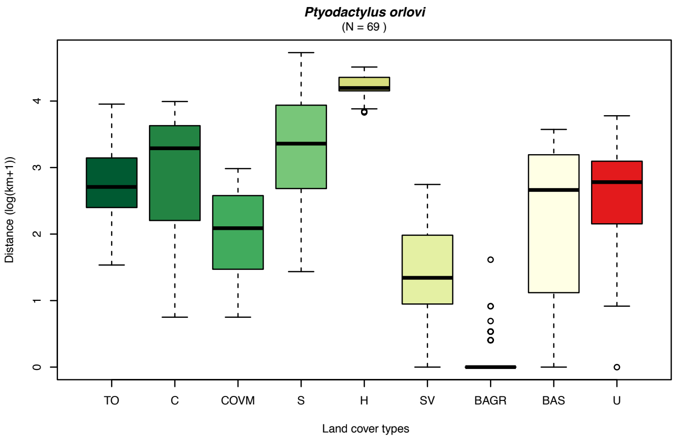

S23: Species information

Lizards

Geckos, Phyllodactylidae

Ptyodactylus ruusaljibalicus

Simó-Riudalbas, Metallinou, Pous, Els, Jayasinghe, Pentek-Zakar, Wilms, Al-Saadi & Carranza, 2017

A

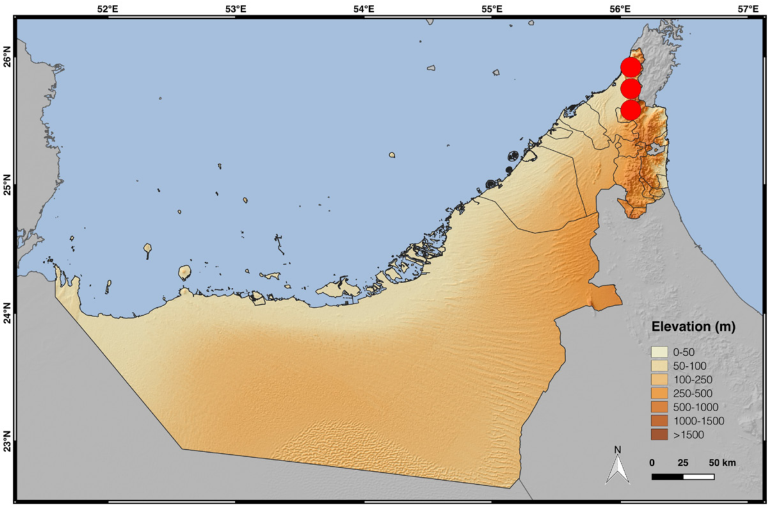

B

Useful information

IUCN Category	NE/LC
Venomous	NO
Endemic	NO
Origin	Native

D

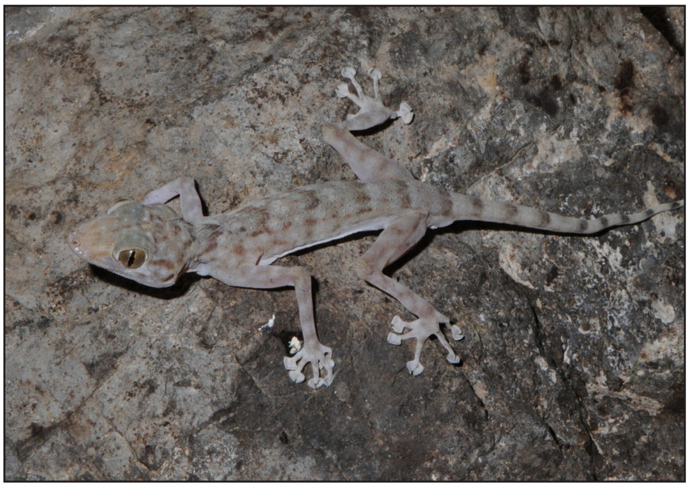

C

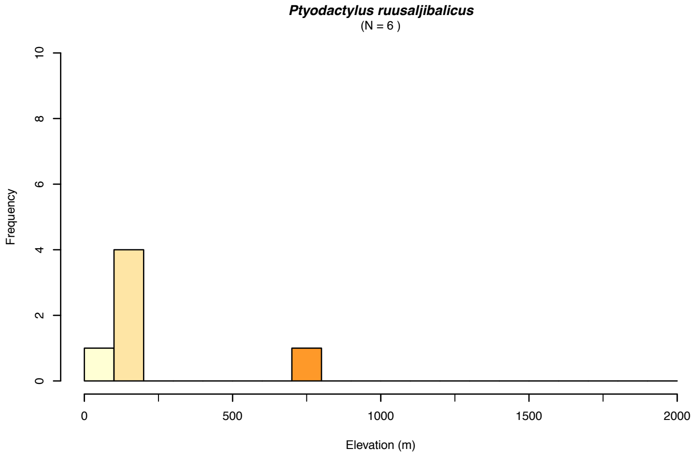

F

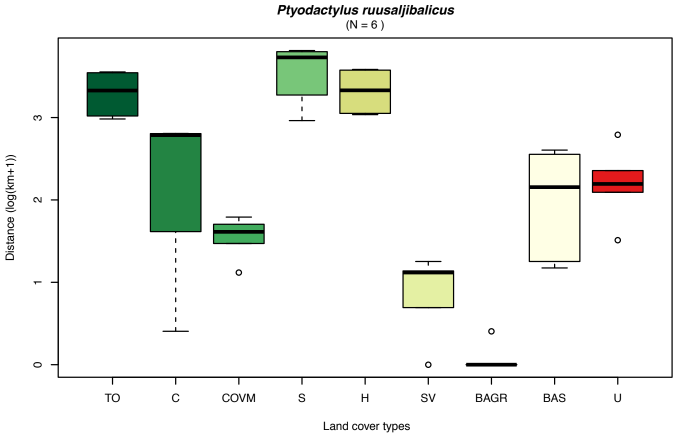

E

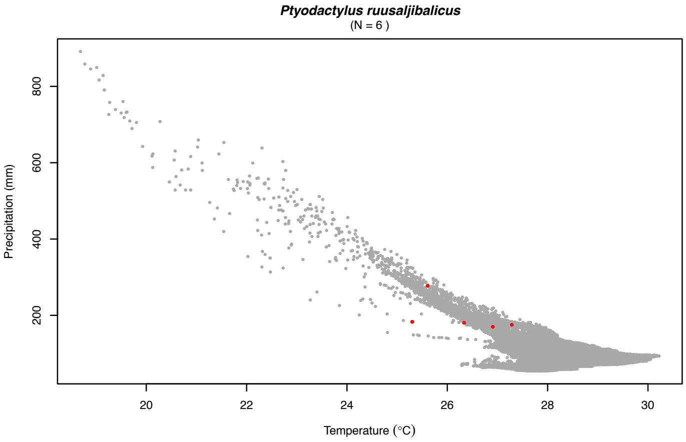

Pristurus carteri (Gray, 1863)

S24: Species information
Lizards
Geckos, Sphaerodactylidae

A

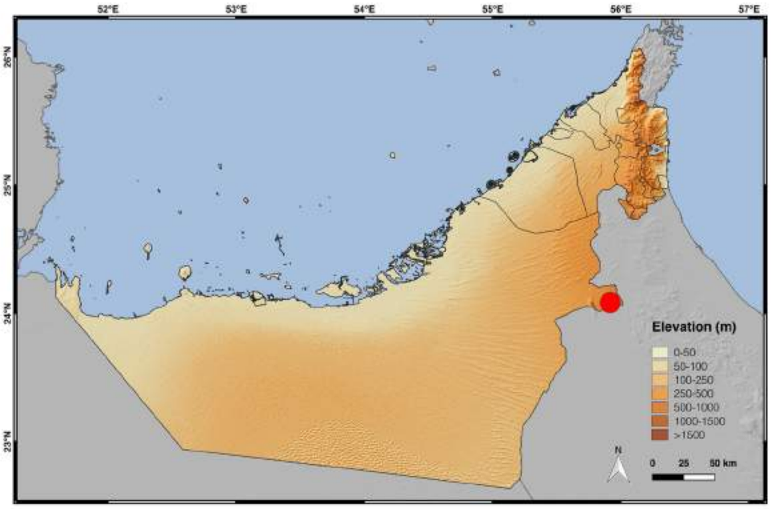

B

Useful information

IUCN Category	LC/CR
Venomous	NO
Endemic	NO
Origin	Native

C

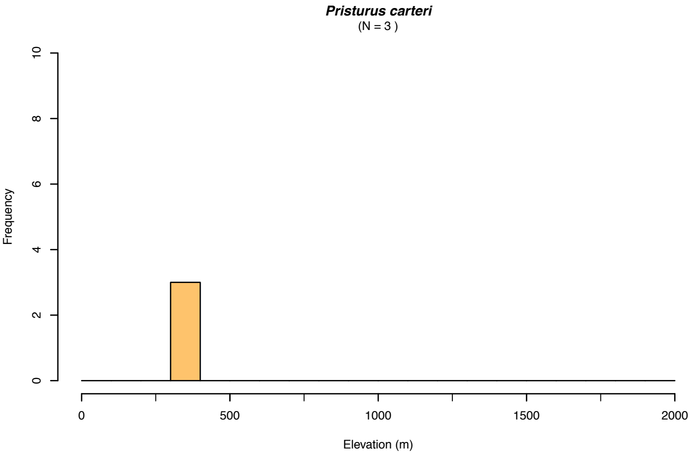

D

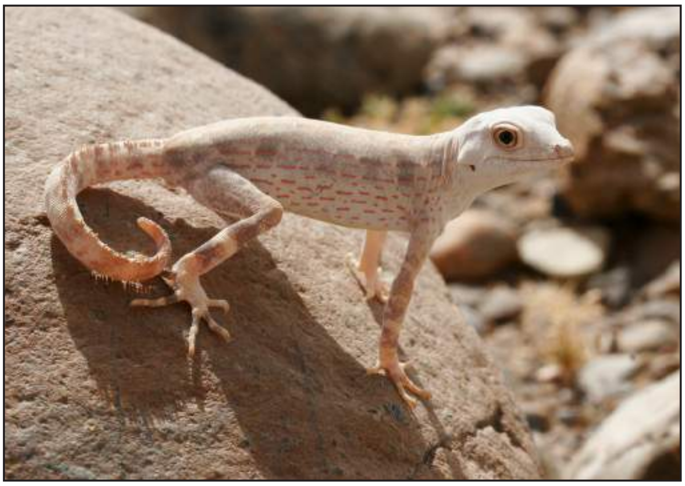

E

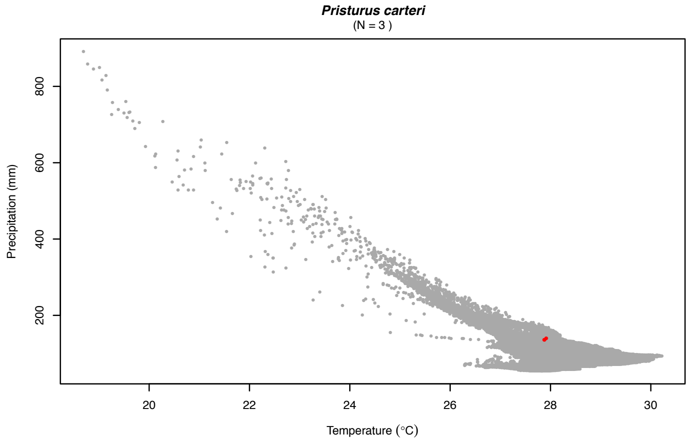

F

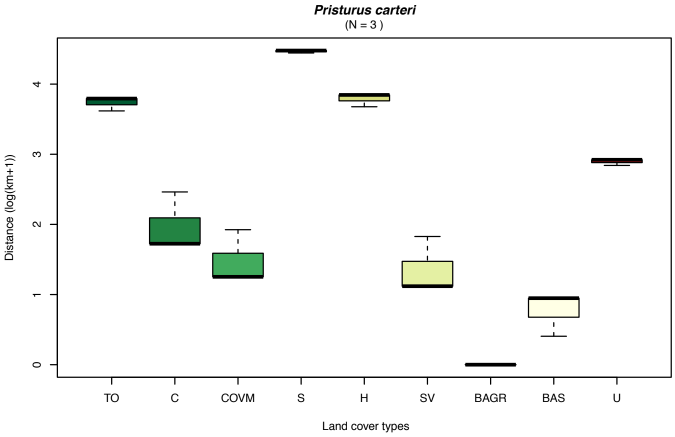

S25: Species information

Lizards

Geckos, Sphaerodactylidae

Pristurus celerrimus
Arnold, 1977

B

Useful information

IUCN Category	LC/LC
Venomous	NO
Endemic	NO
Origin	Native

A

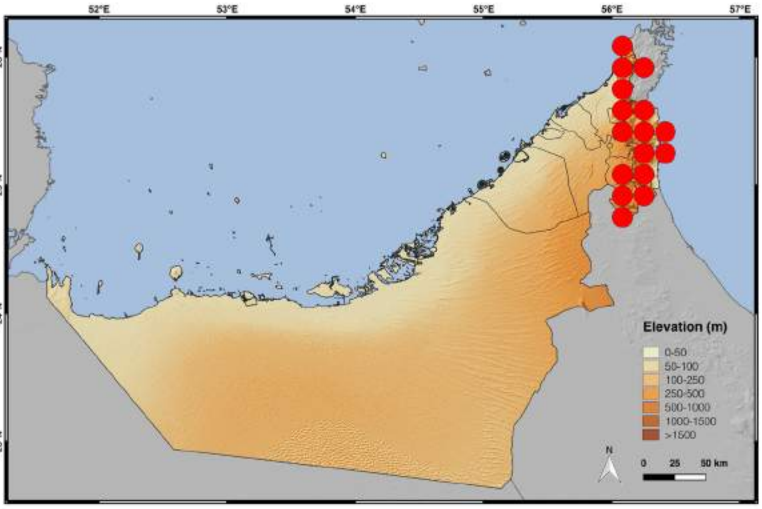

D

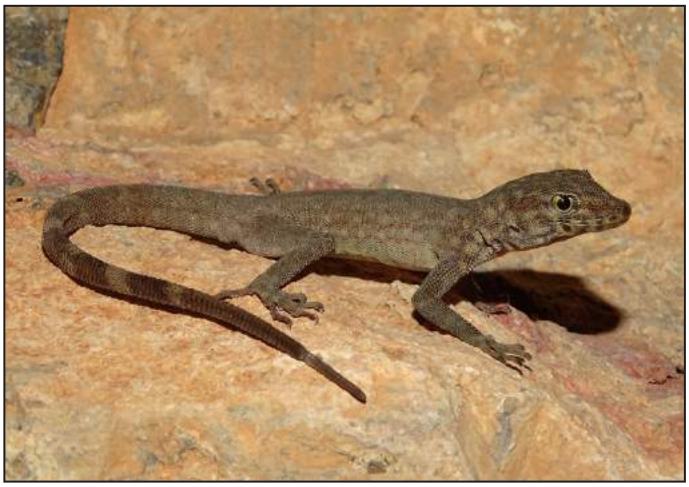

C

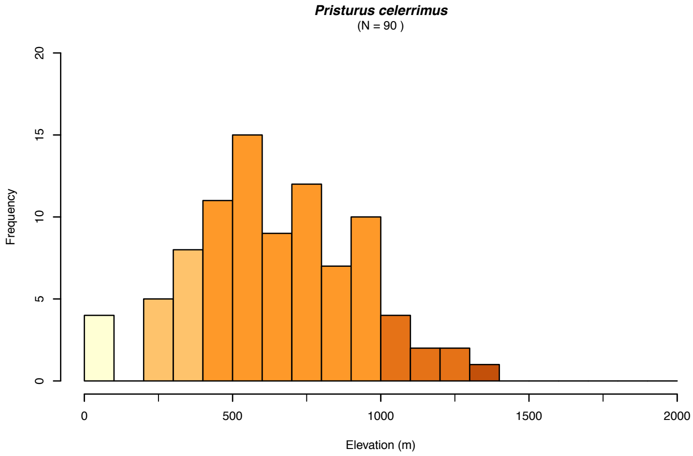

F

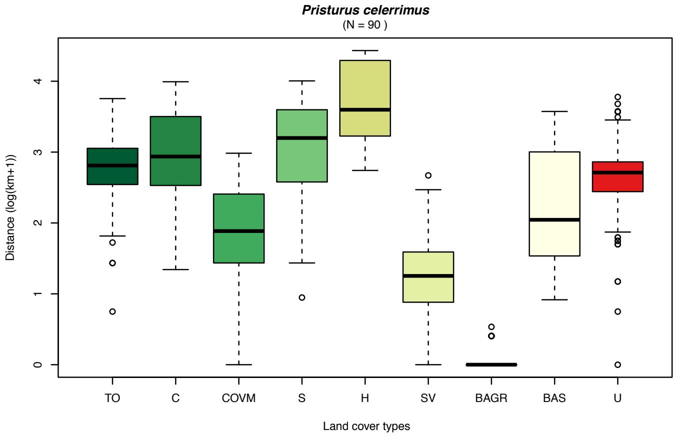

E

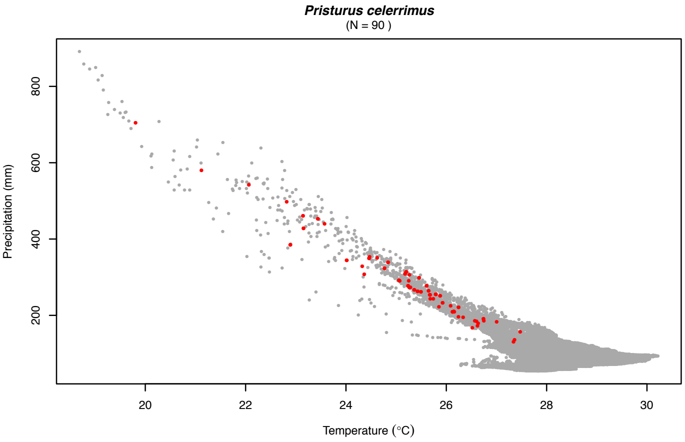

Pristurus minimus
Arnold, 1977

S26: Species information
Lizards
Geckos, Sphaerodactylidae

A

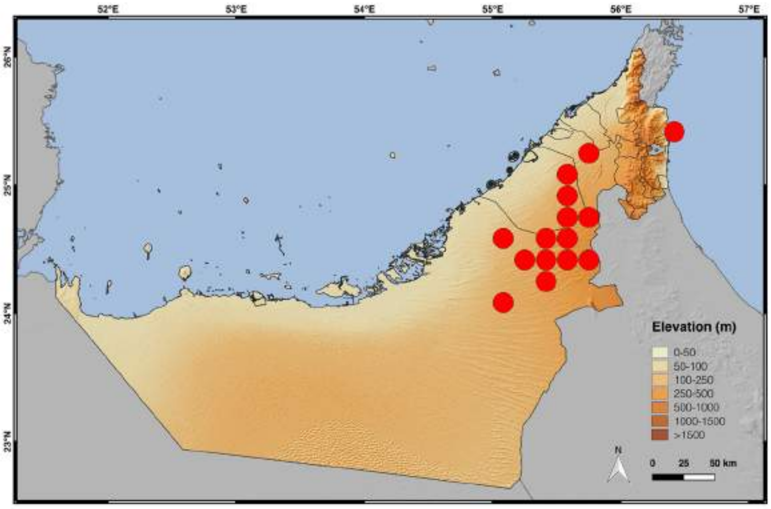

B

Useful information

IUCN Category	LC/LC
Venomous	NO
Endemic	NO
Origin	Native

C

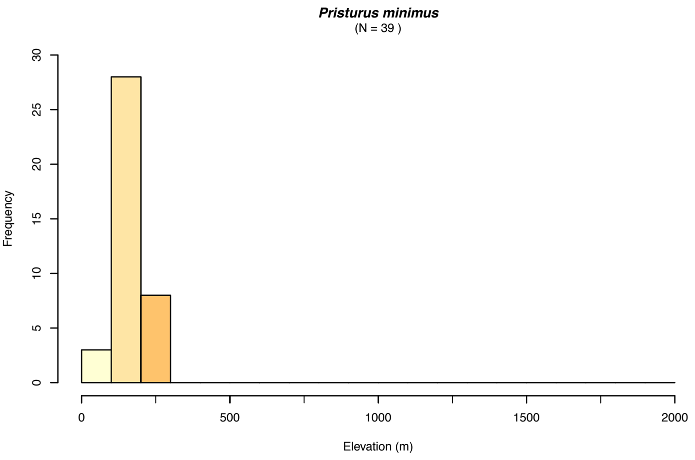

D

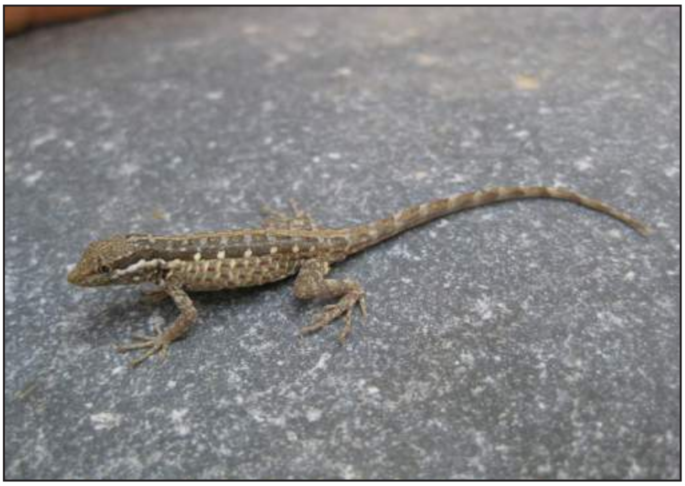

E

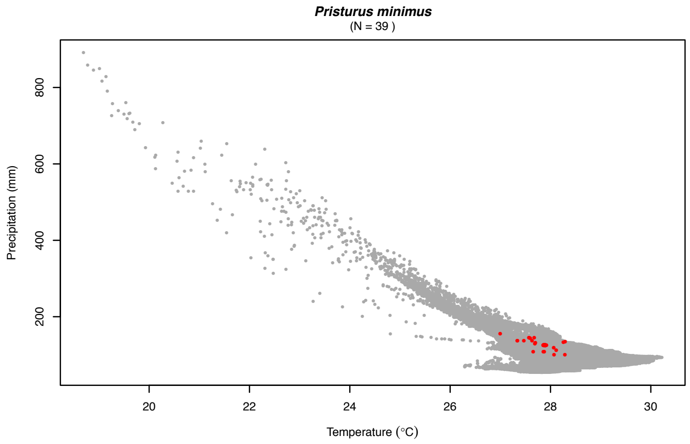

F

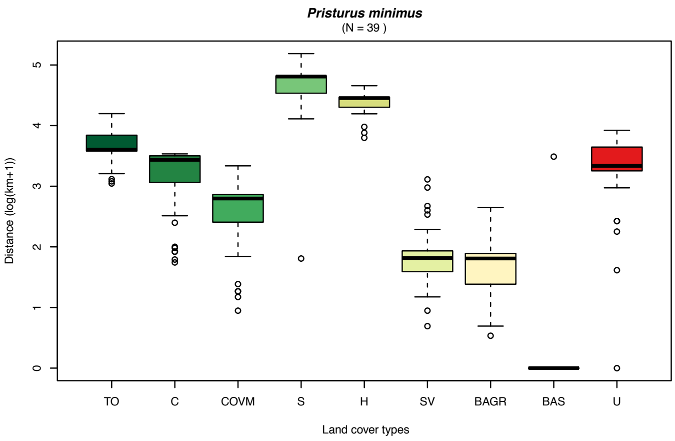

S27: Species information

Lizards

Geckos, Sphaerodactylidae

Pristurus rupestris-sp. 3

Undescribed

A

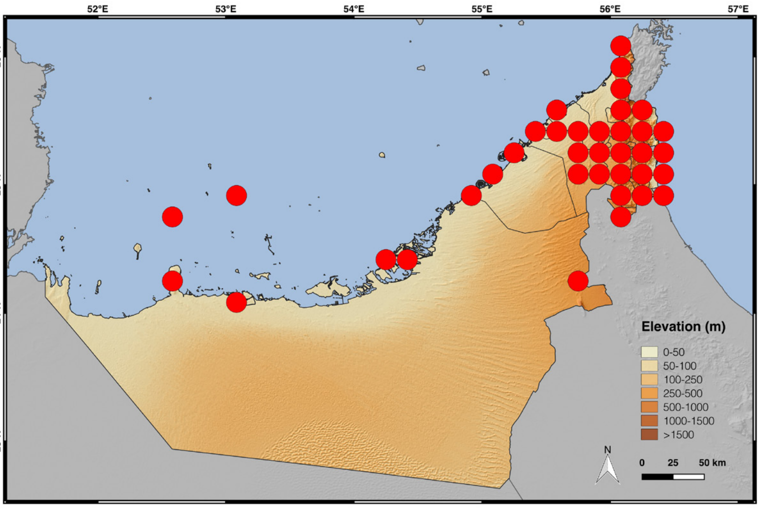

B

Useful information

IUCN Category	NE/LC
Venomous	NO
Endemic	NO
Origin	Native

D

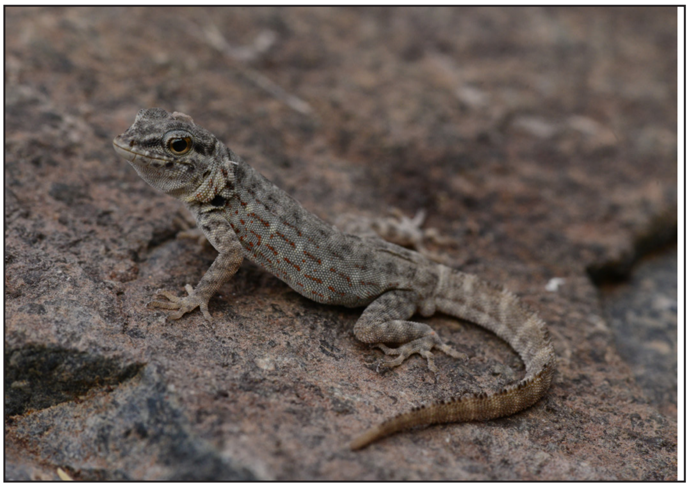

C

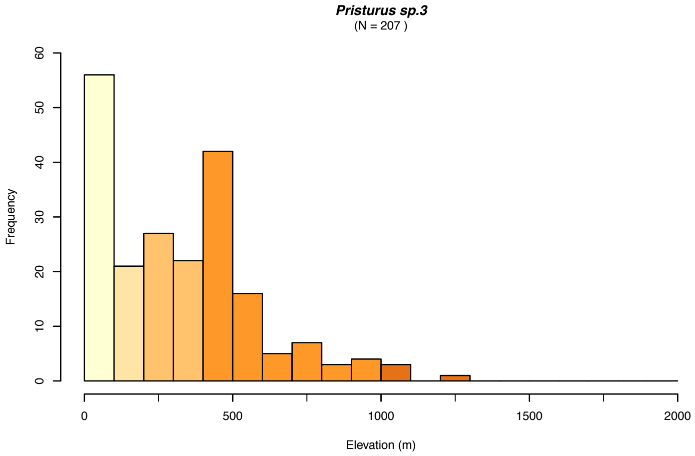

F

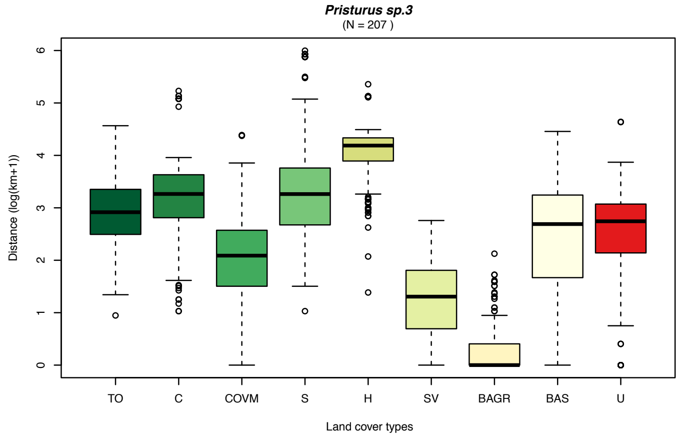

E

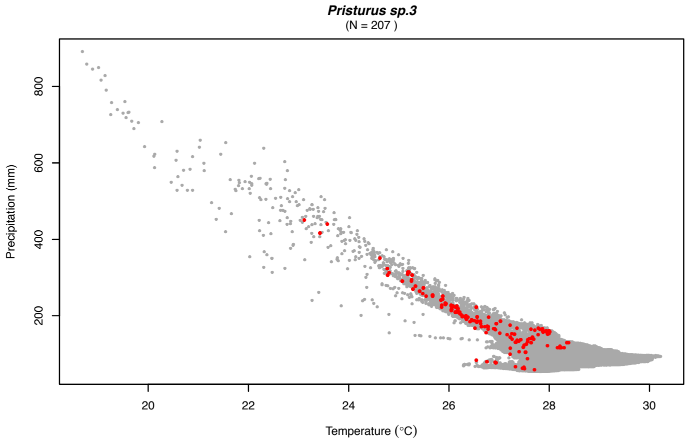

Teratoscincus keyserlingii Strauch, 1863

S28: Species information
Lizards
Geckos, Sphaerodactylidae

A

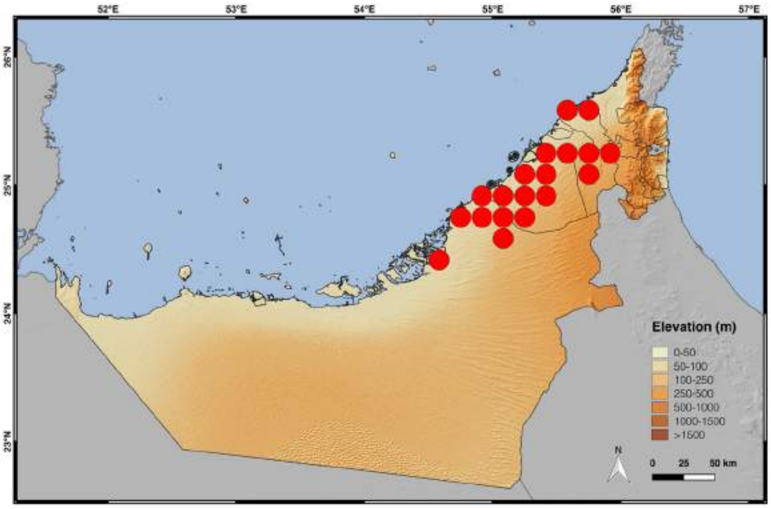

B

Useful information

IUCN Category	LC/CR
Venomous	NO
Endemic	NO
Origin	Native

C

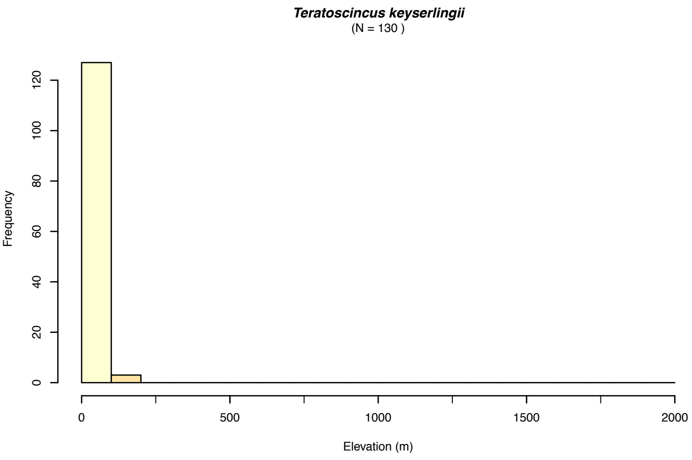

D

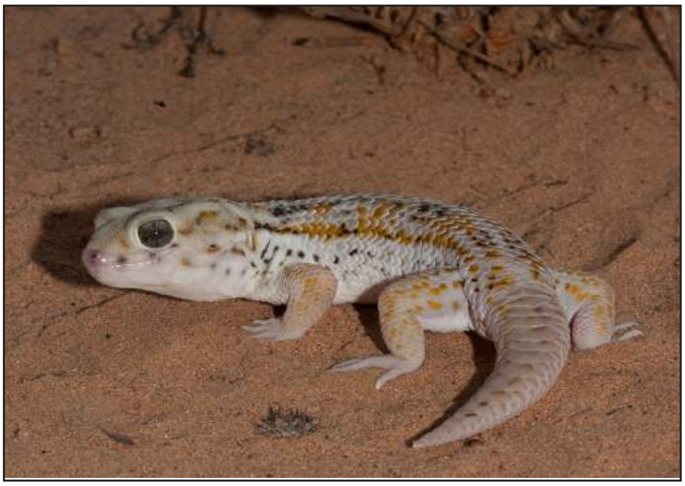

E

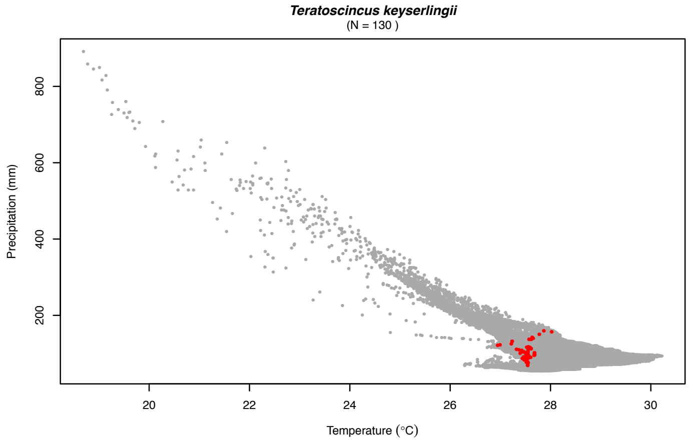

F

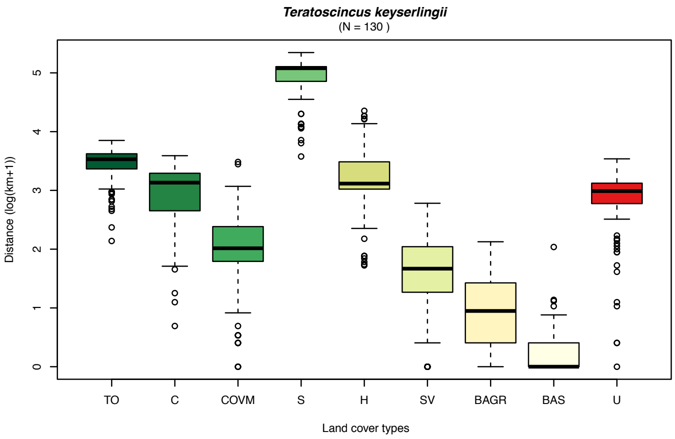

S29: Species information

Lizards

Lacertids, Lacertidae

Acanthodactylus blanfordii

Boulenger, 1918

A

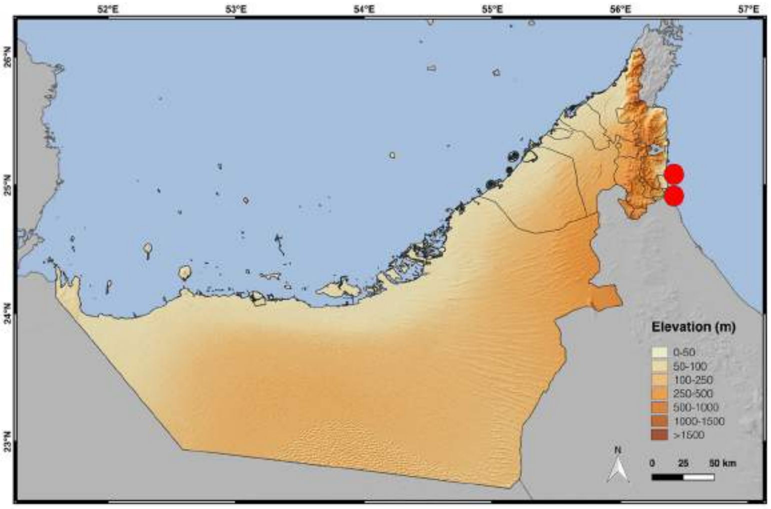

B

Useful information	
IUCN Category	LC/VU
Venomous	NO
Endemic	NO
Origin	Native

D

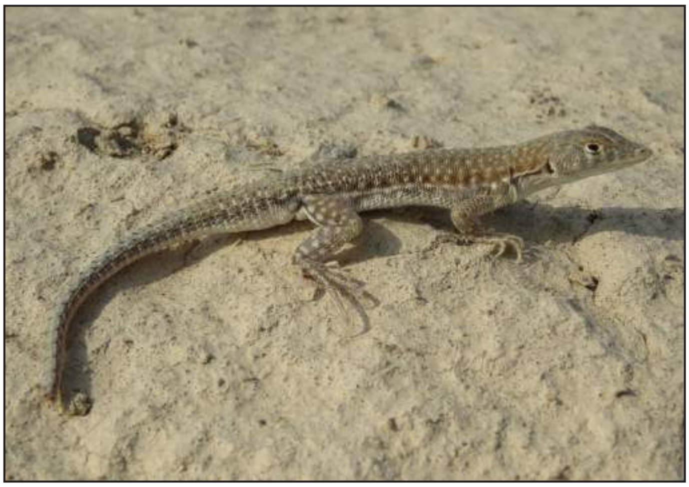

C

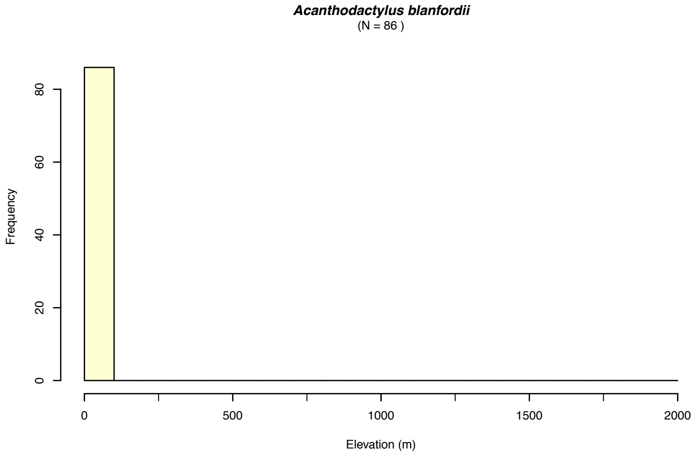

F

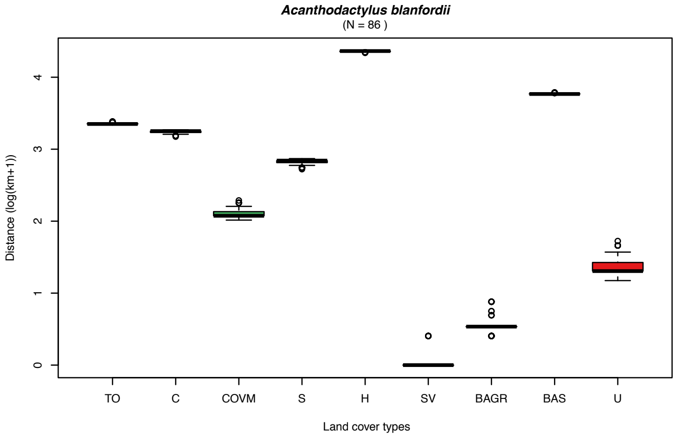

E

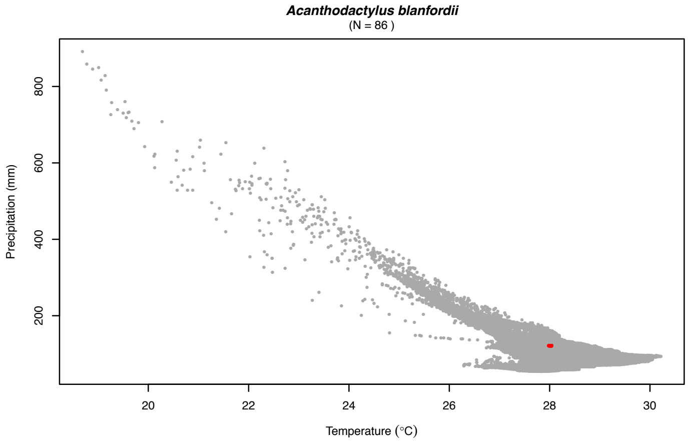

Lizards
Agamids
Geckos
Lacertids
Skinkis
Amphisbaenids
Varanids
Snakes
Boidae
Colubridae
Lamprophiidae
Leptotyphlopidae
Typhlopidae
Viperidae

Acanthodactylus boskianus asper (Audouin, 1829)

S30: Species information
Lizards
Lacertids, Lacertidae

A

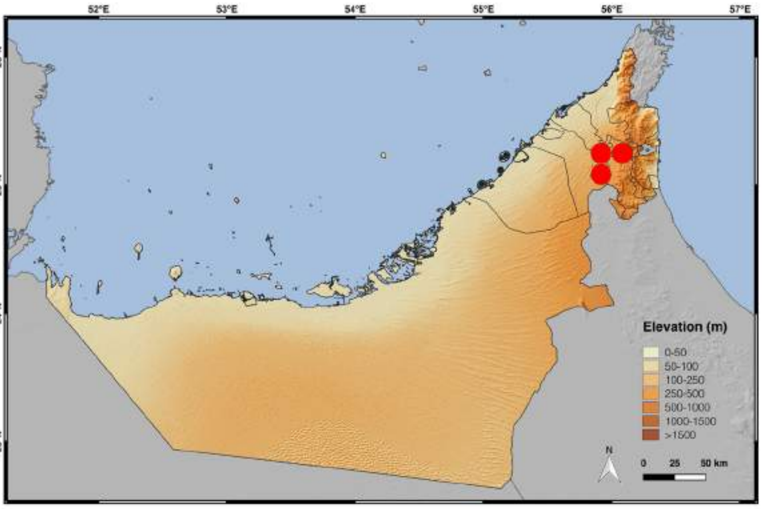

B

Useful information

IUCN Category	LC/NT
Venomous	NO
Endemic	NO
Origin	Native

C

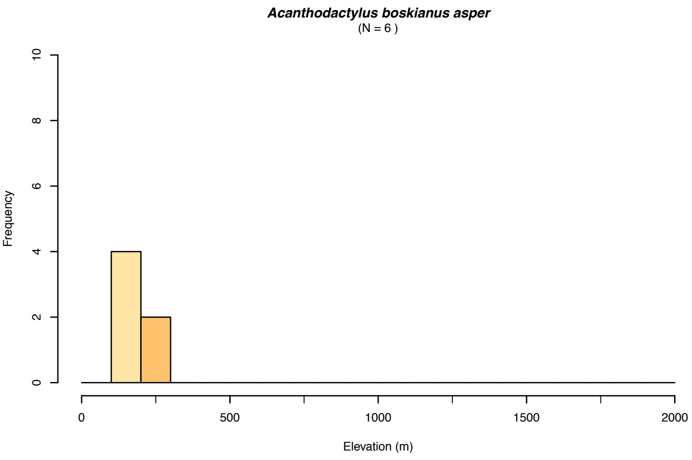

D

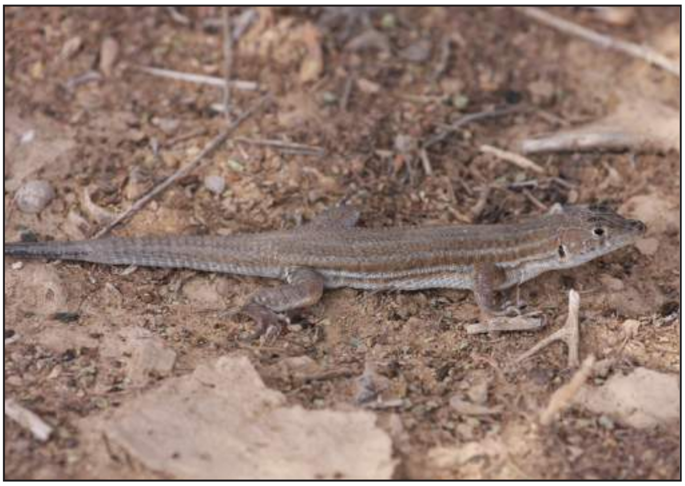

E

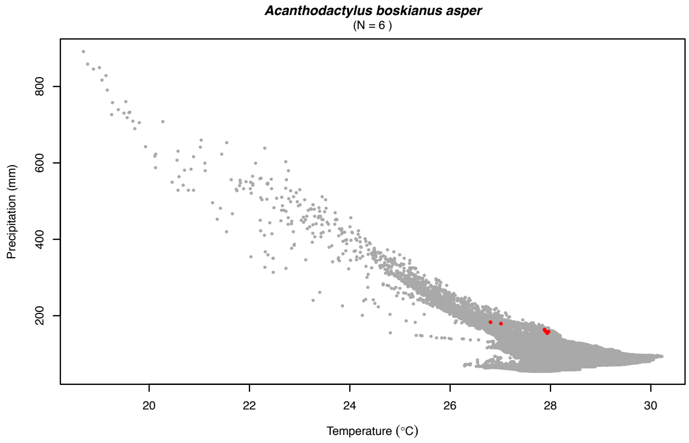

F

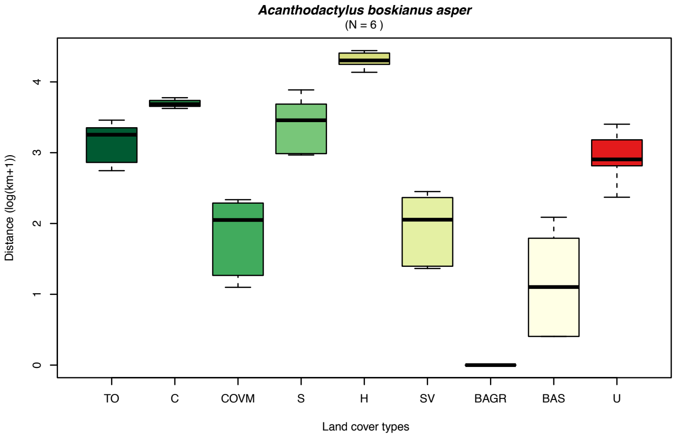

S31: Species information

Lizards

Lacertids, Lacertidae

Acanthodactylus gongrorhynchatus

Leviton & Anderson, 1967

A

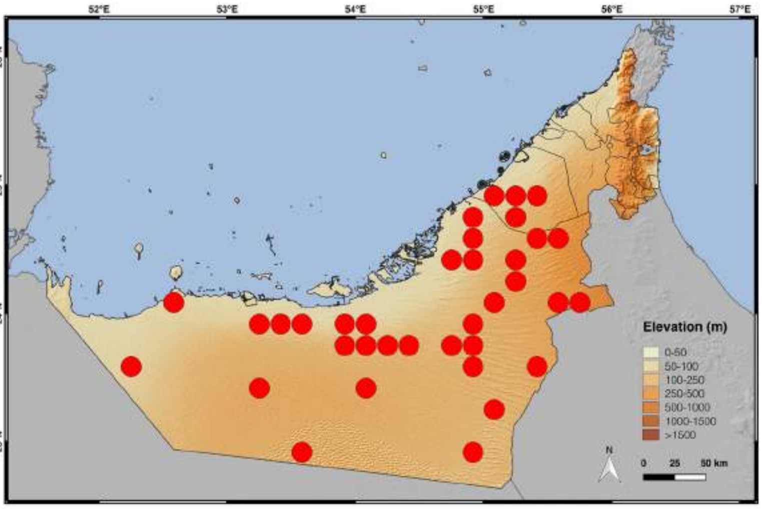

B

Useful information	
IUCN Category	DD/LC
Venomous	NO
Endemic	NO
Origin	Native

D

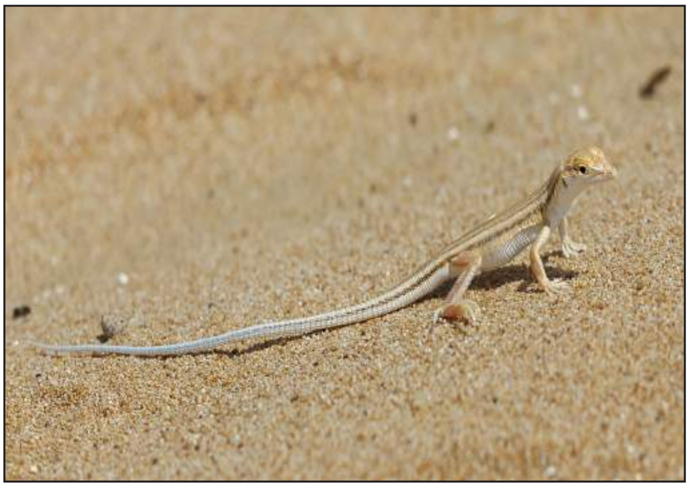

C

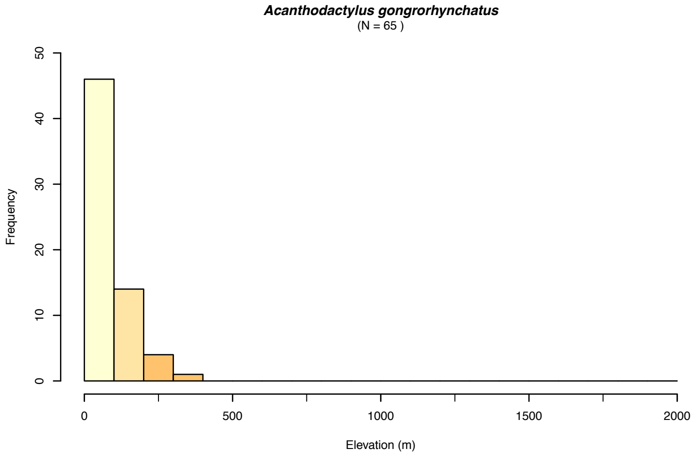

F

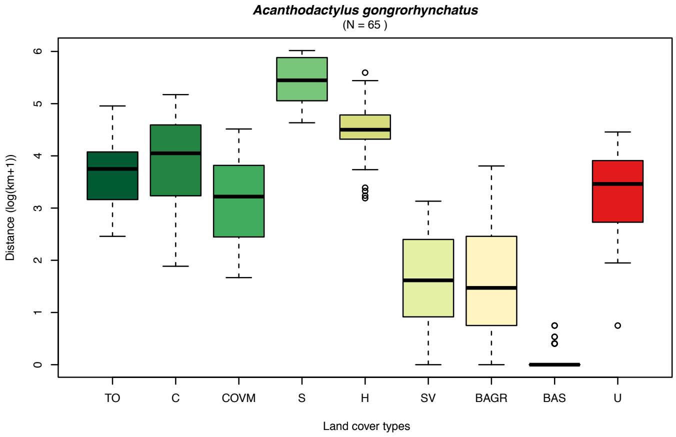

E

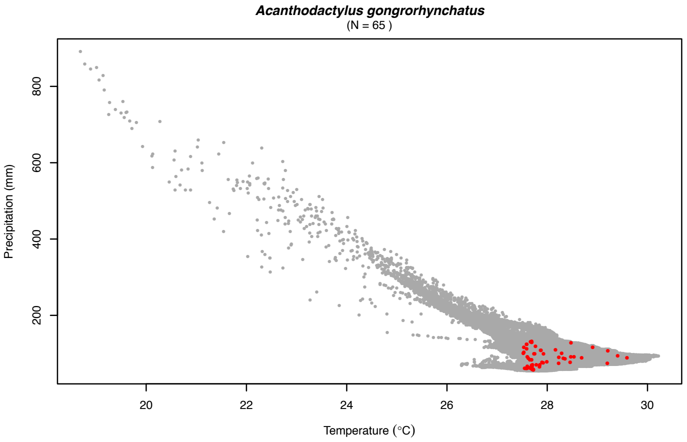

Acanthodactylus haasi
Leviton & Anderson, 1967

S32: Species information
Lizards
Lacertids, Lacertidae

A

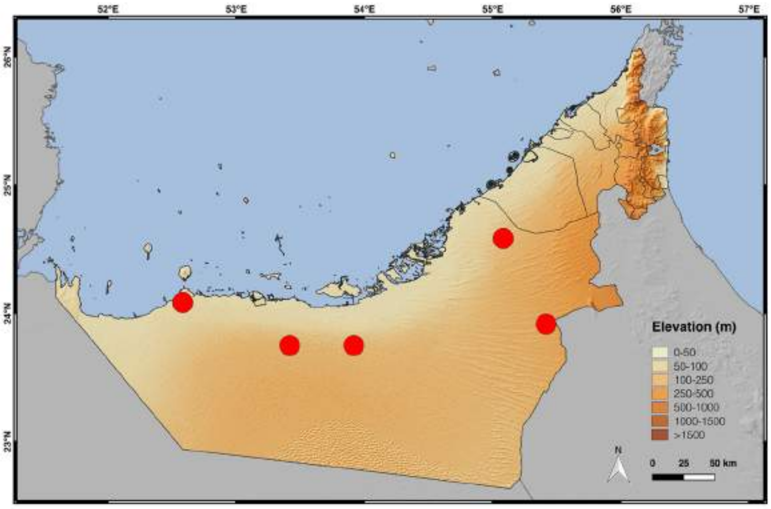

B

Useful information	
IUCN Category	LC/DD
Venomous	NO
Endemic	NO
Origin	Native

C

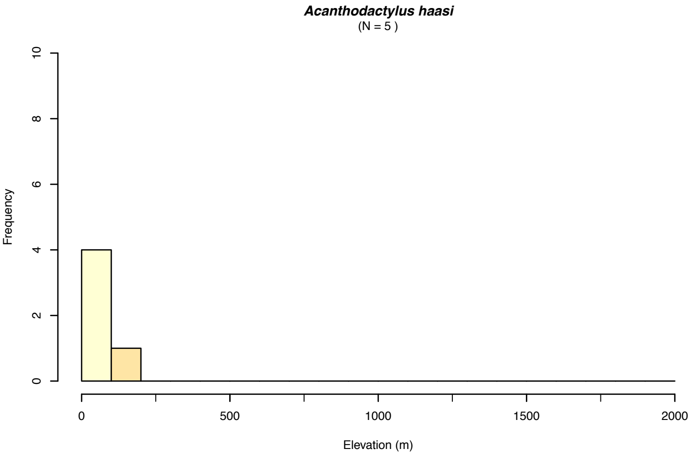

D

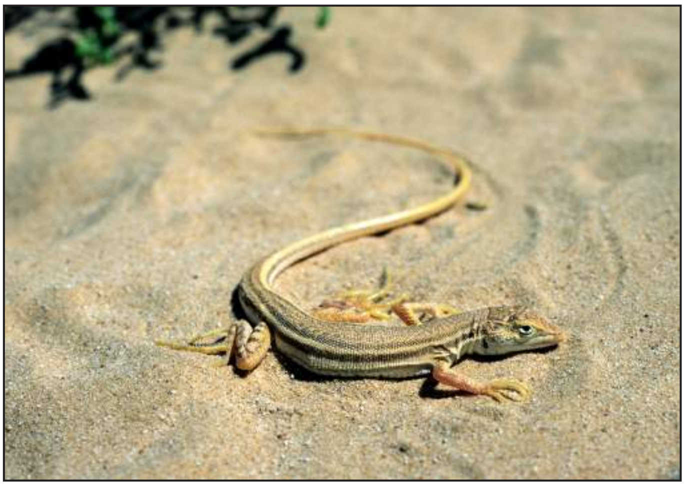

E

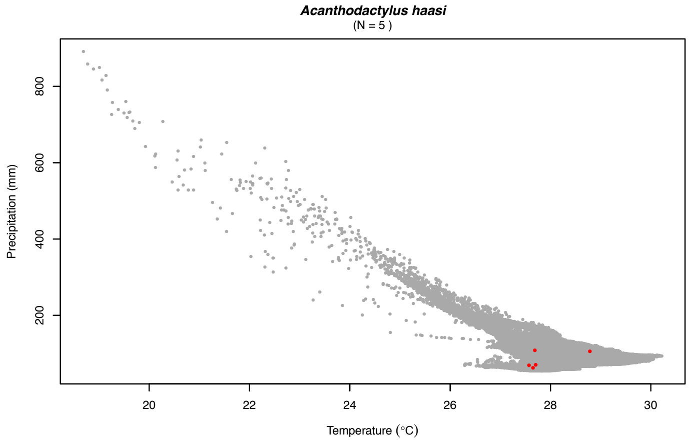

F

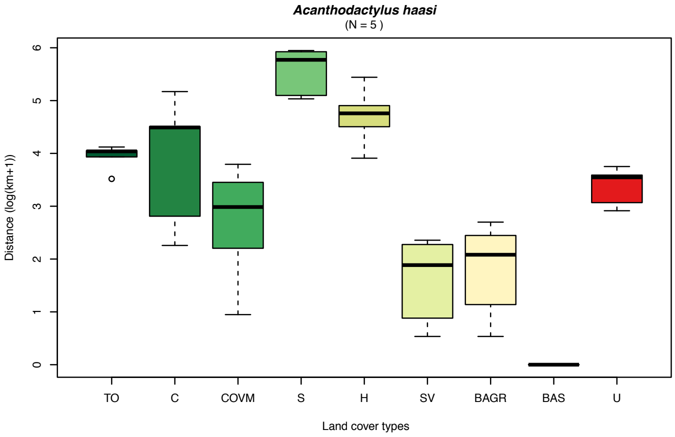

S33: Species information
Lizards
Lacertids, Lacertidae

Acanthodactylus opheodurus
Arnold, 1980

B

Useful information	
IUCN Category	LC/DD
Venomous	NO
Endemic	NO
Origin	Native

A

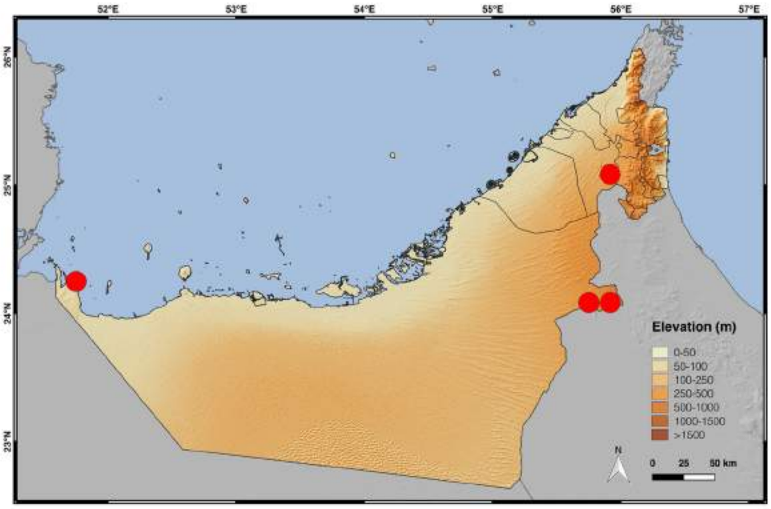

D

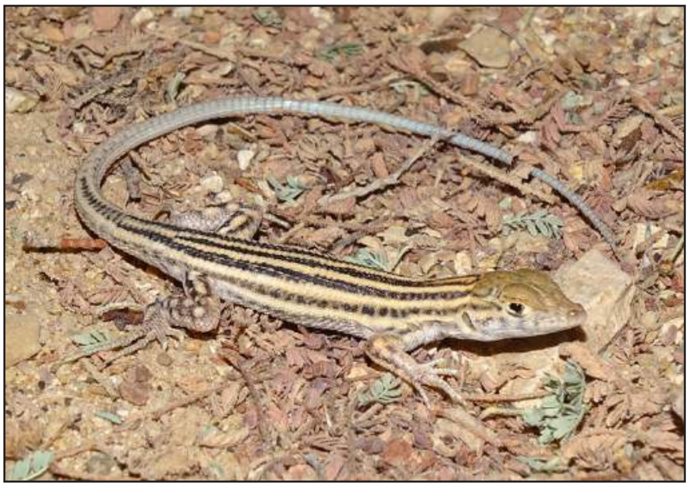

C

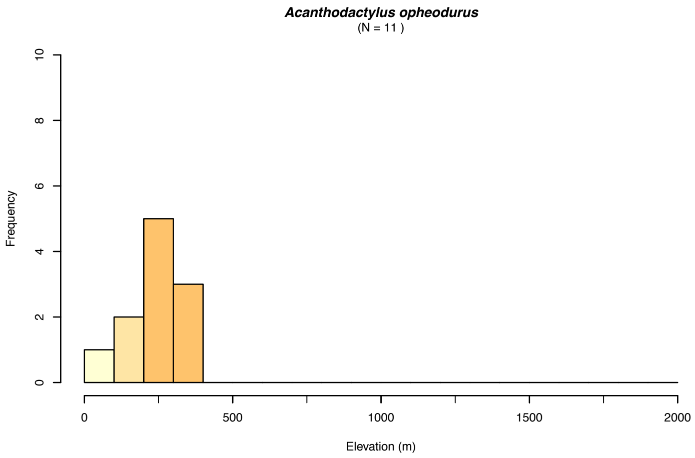

F

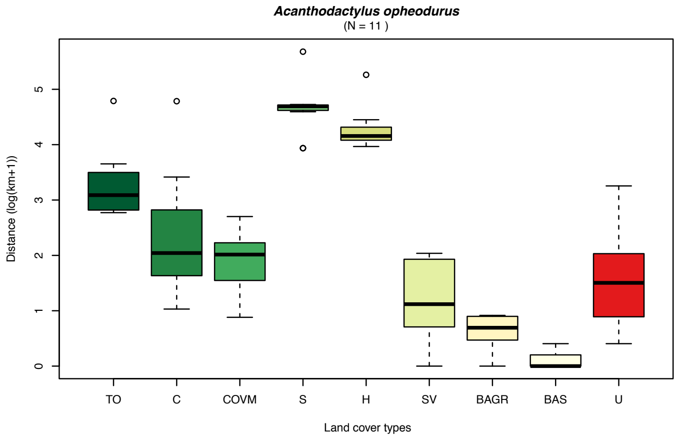

E

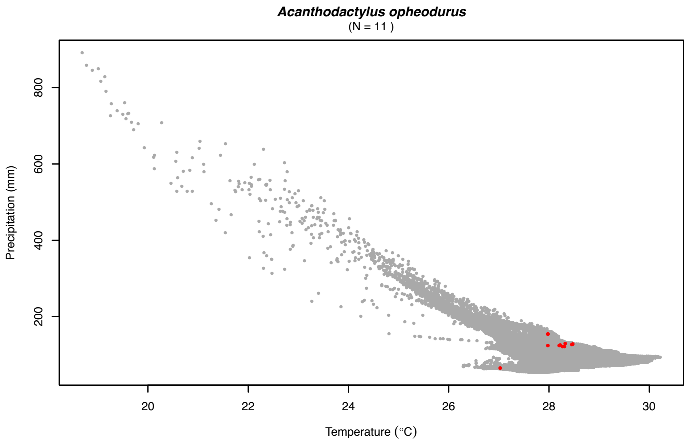

Acanthodactylus schmidti Haas, 1957

S34: Species information

Lizards
Lacertids, Lacertidae

A

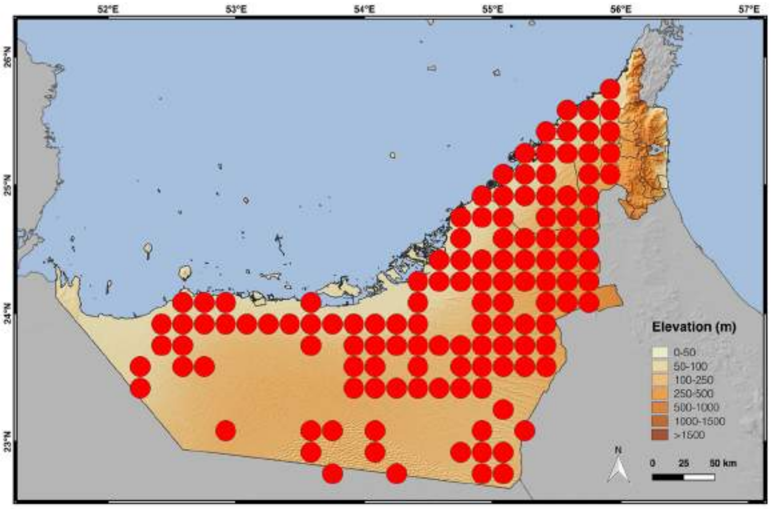

B

Useful information	
IUCN Category	LC/LC
Venomous	NO
Endemic	NO
Origin	Native

C

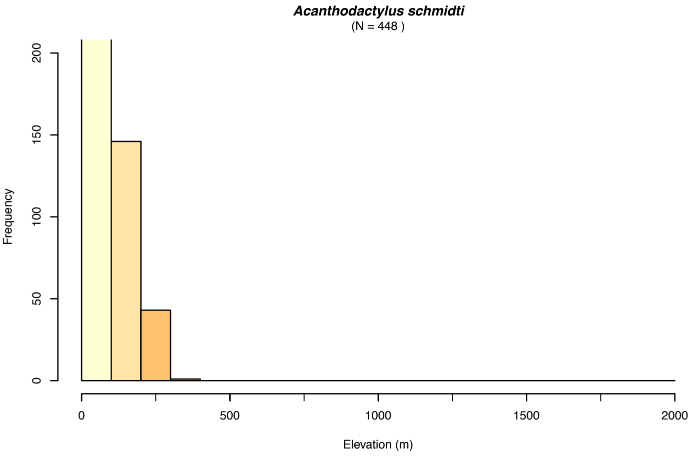

D

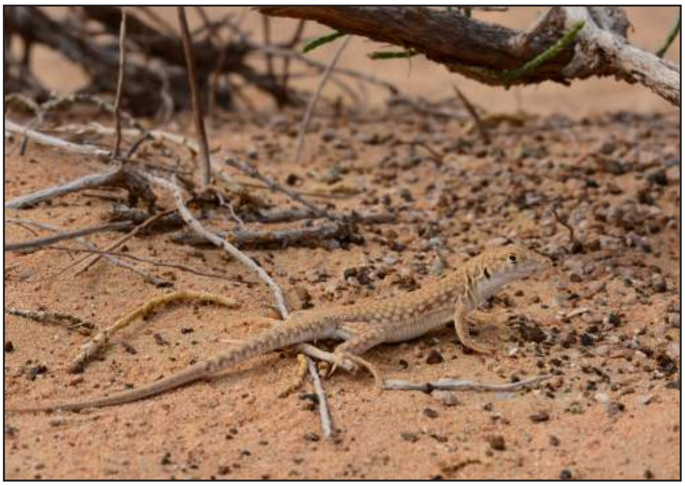

E

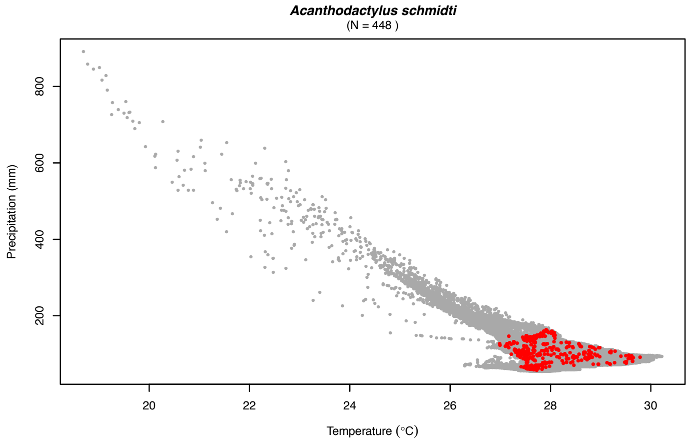

F

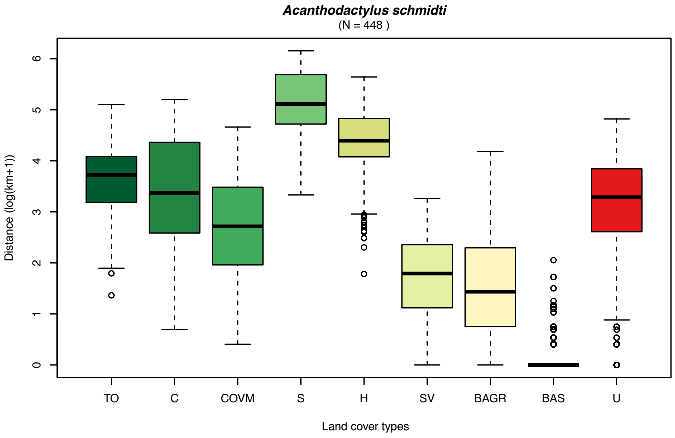

S35: Species information

Lizards
Lacertids, Lacertidae

Mesalina adramitana (Boulenger, 1917)

A

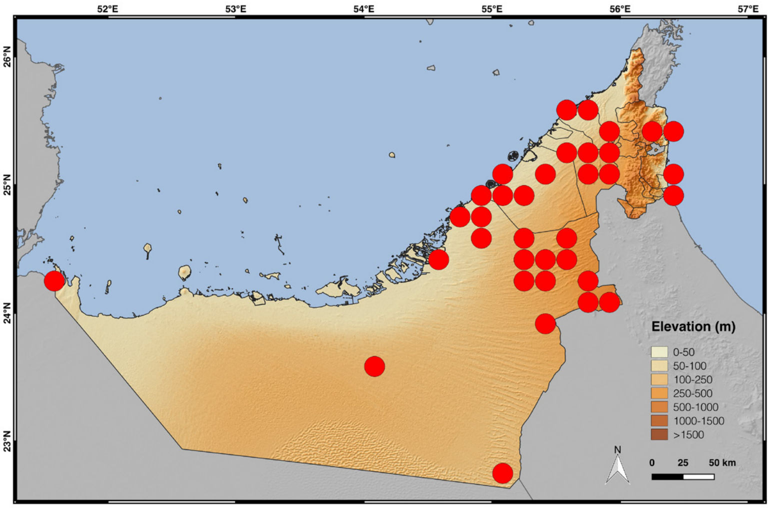

B

Useful information

IUCN Category	LC/LC
Venomous	NO
Endemic	NO
Origin	Native

D

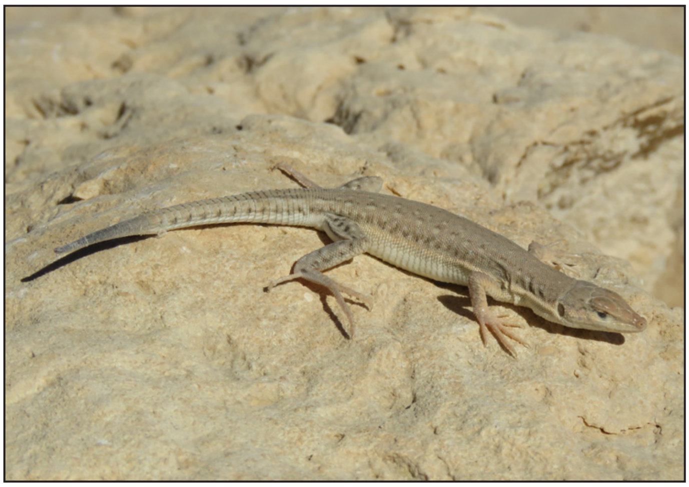

C

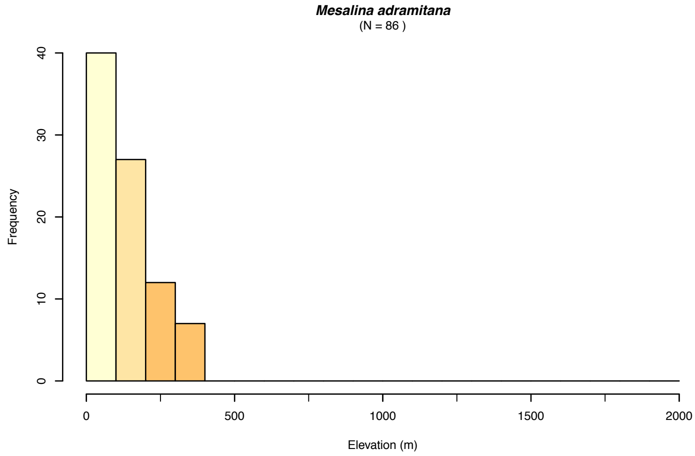

F

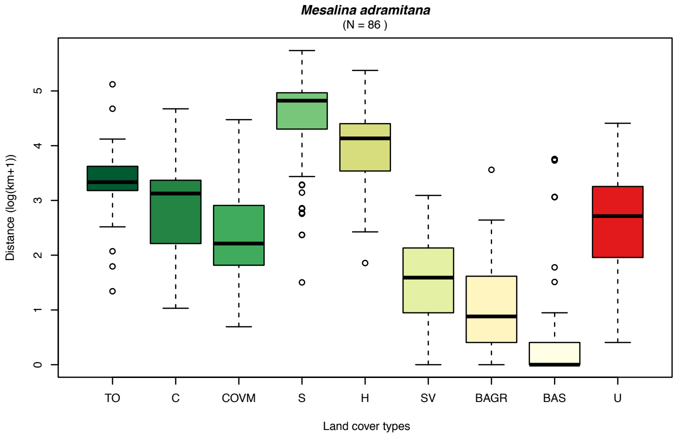

E

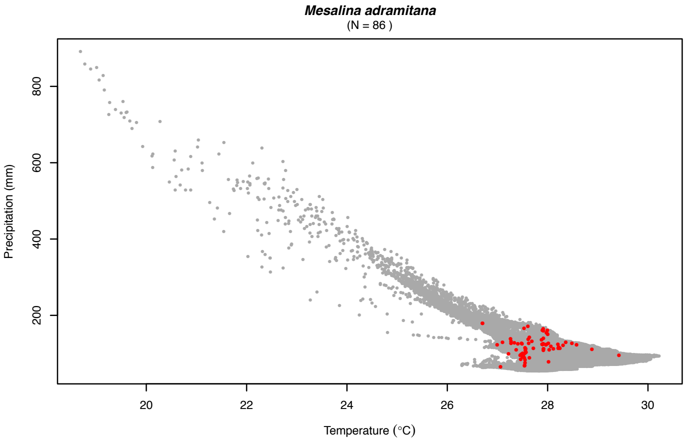

Mesalina brevirostris
Blanford, 1874

S36: Species information
Lizards
Lacertids, Lacertidae

A

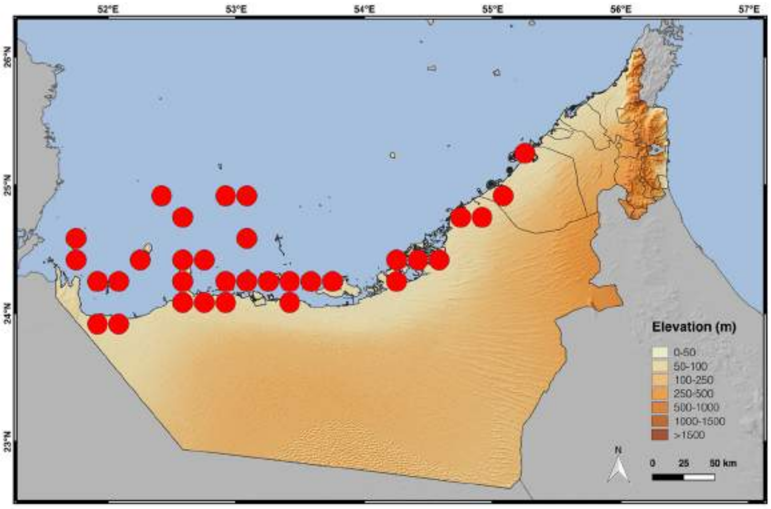

B

Useful information	
IUCN Category	LC/LC
Venomous	NO
Endemic	NO
Origin	Native

C

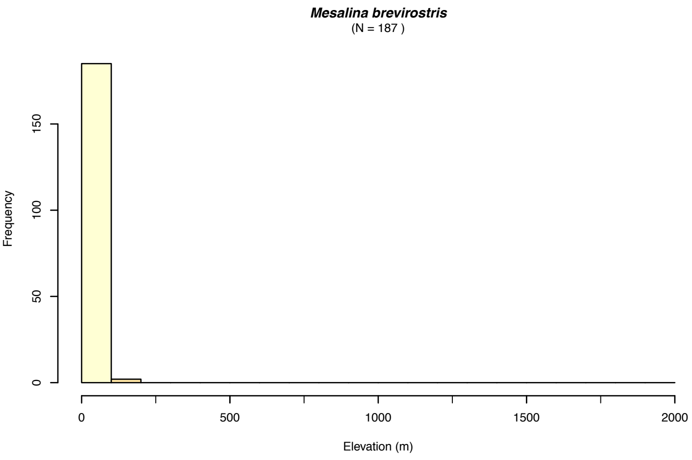

D

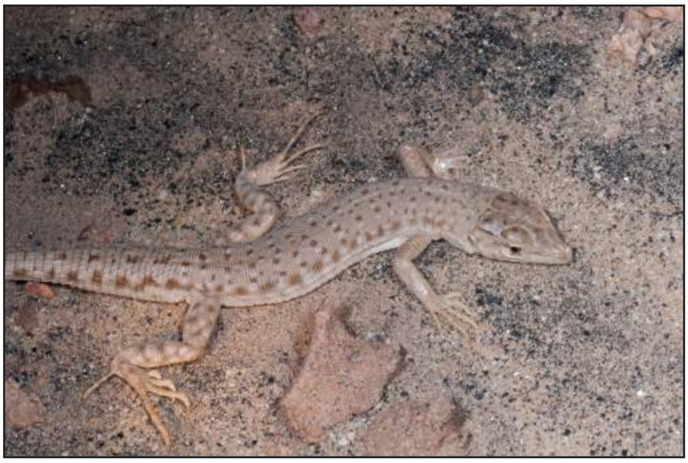

E

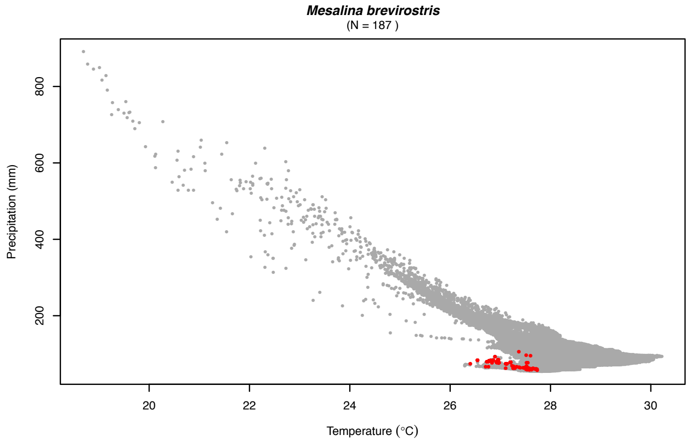

F

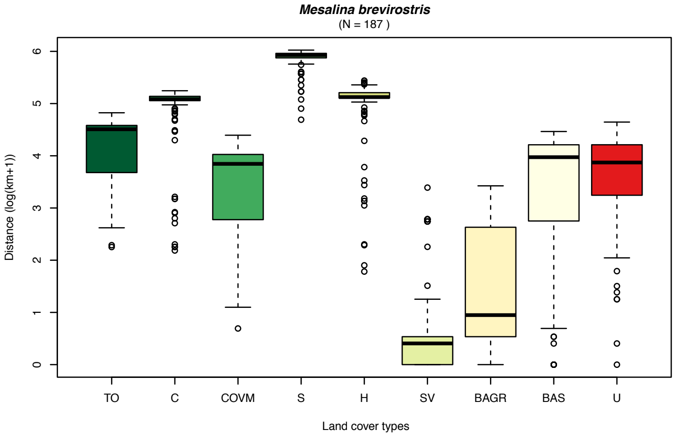

S37: Species information

Lizards

Lacertids, Lacertidae

Omanosaura cyanura (Arnold, 1972)

A

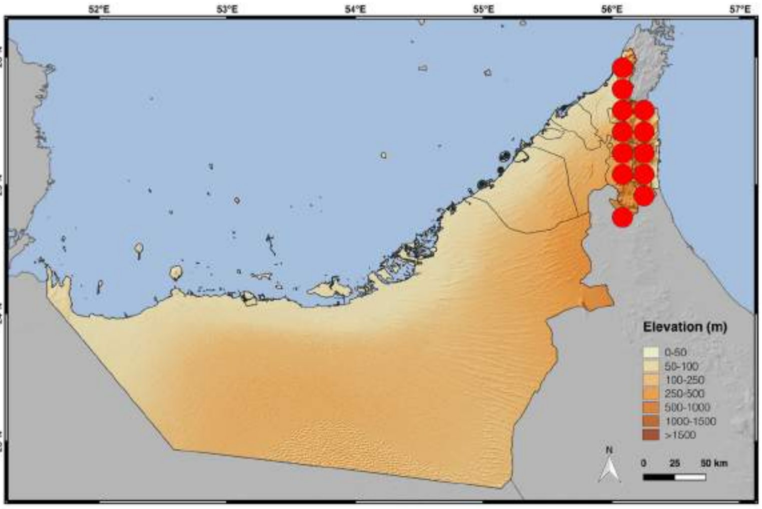

B

Useful information

IUCN Category	LC/LC
Venomous	NO
Endemic	NO
Origin	Native

D

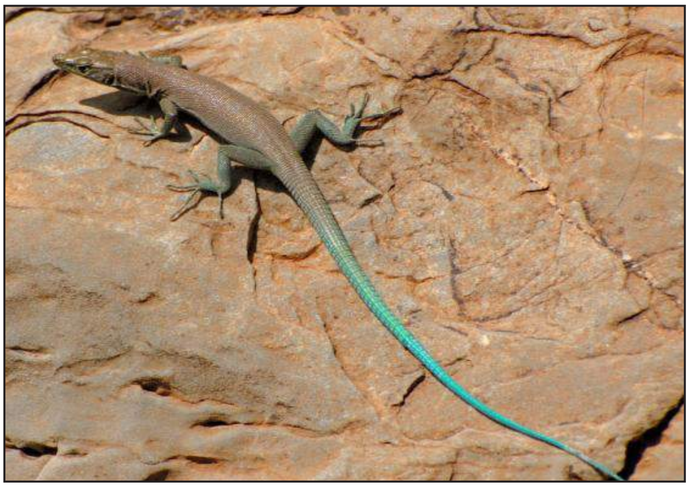

C

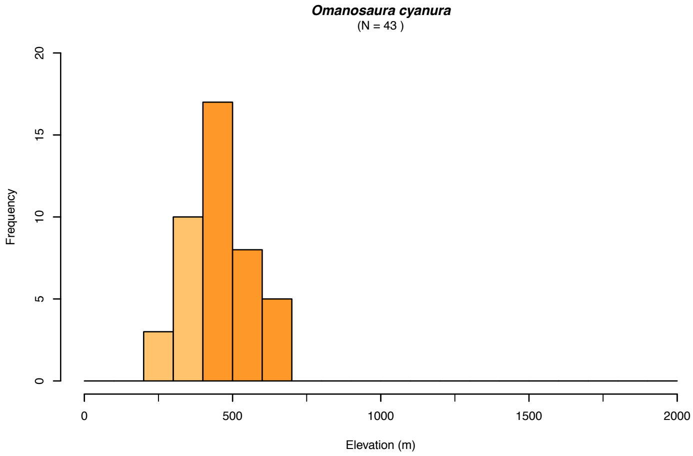

F

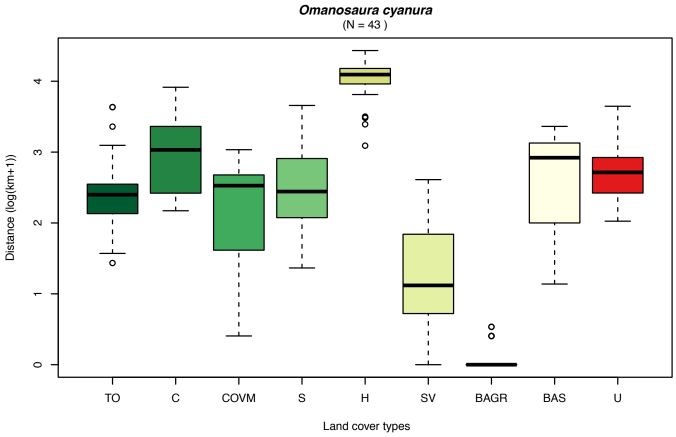

E

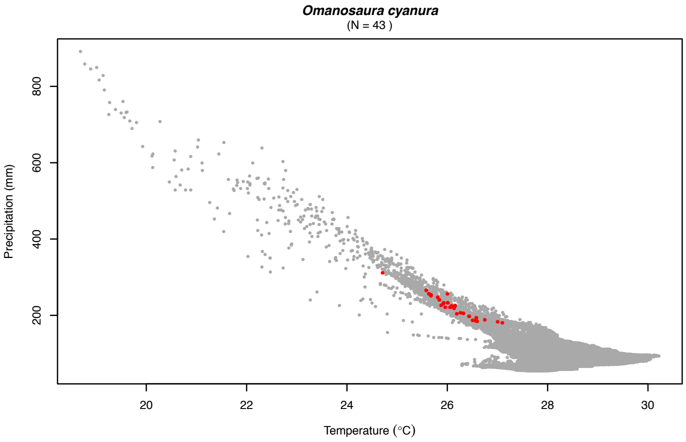

Omanosaura jayakari (Boulenger, 1887)

S38: Species information

Lizards

Lacertids, Lacertidae

A

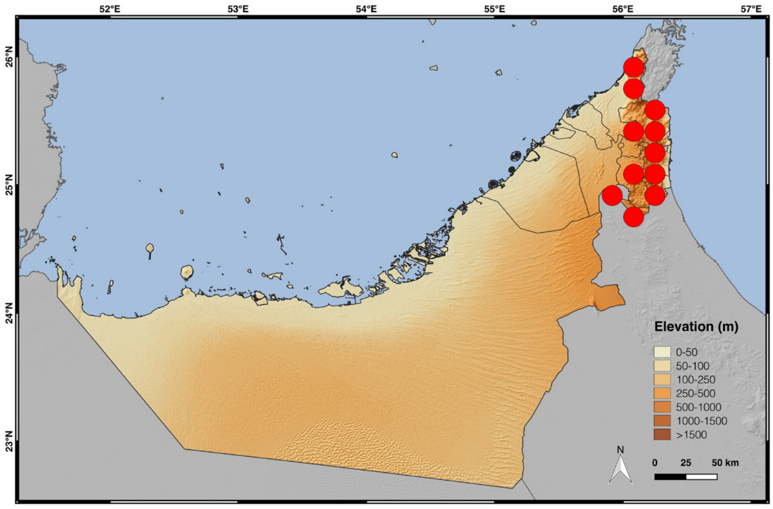

B

Useful information

IUCN Category	LC/LC
Venomous	NO
Endemic	NO
Origin	Native

C

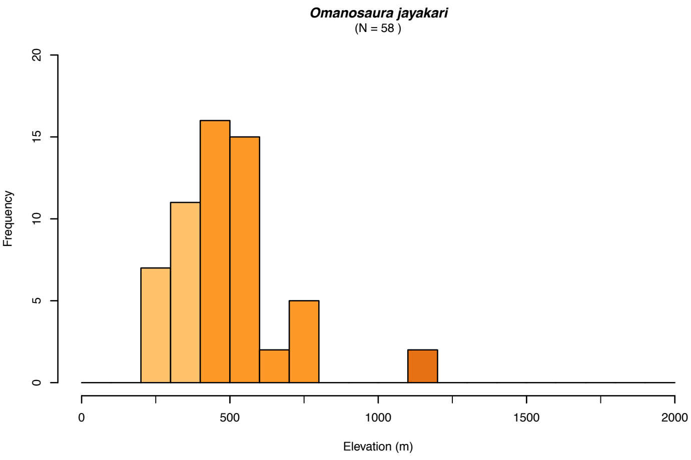

D

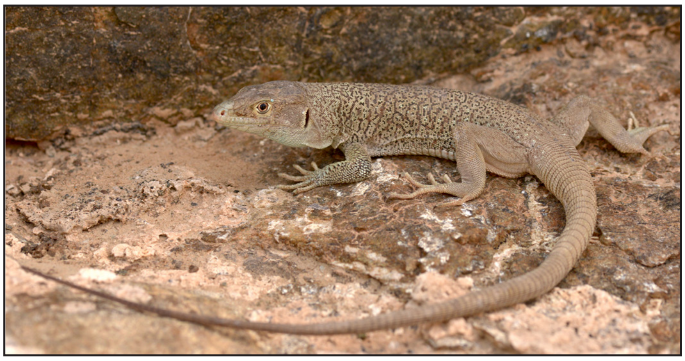

E

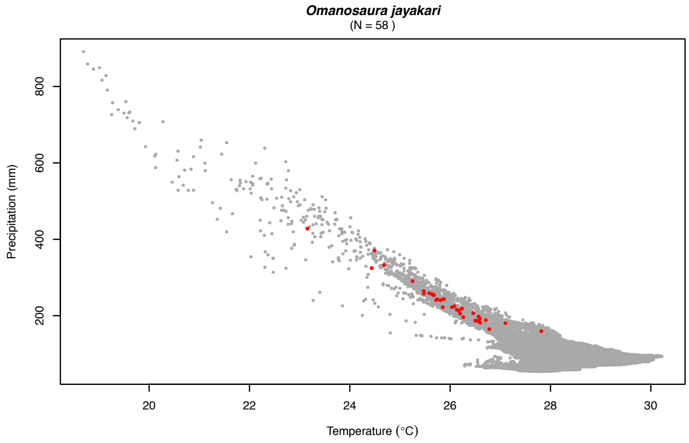

F

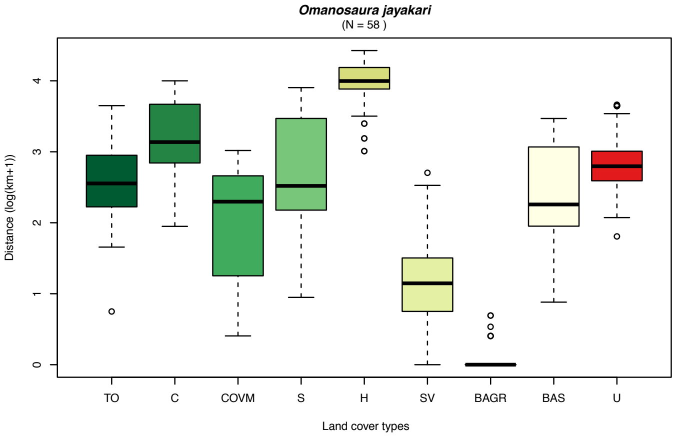

S39: Species information

Lizards

Skinks, Scincidae

Ablepharus pannonicus (Fitzinger, 1824)

A

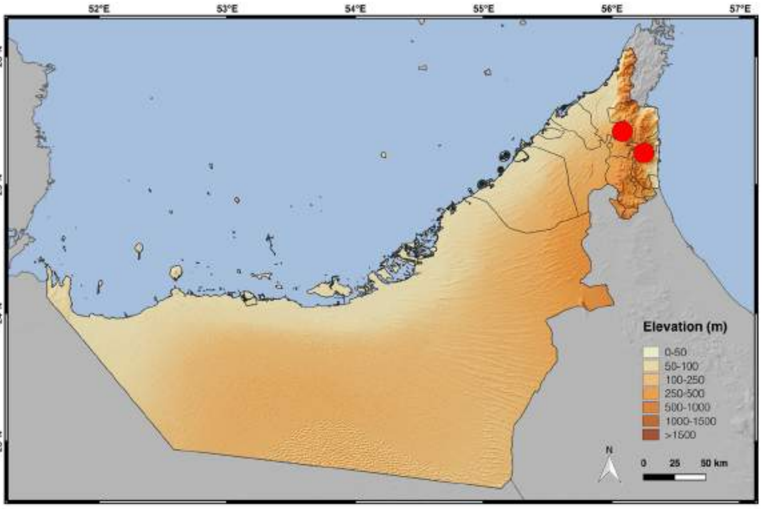

B

Useful information	
IUCN Category	LC/LC
Venomous	NO
Endemic	NO
Origin	Native

D

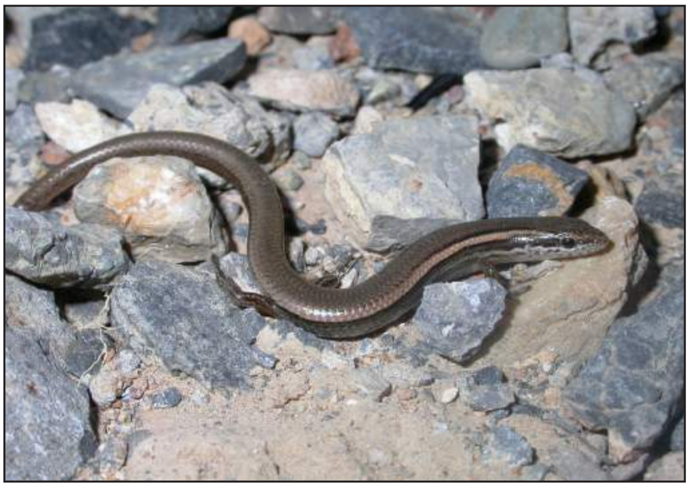

C

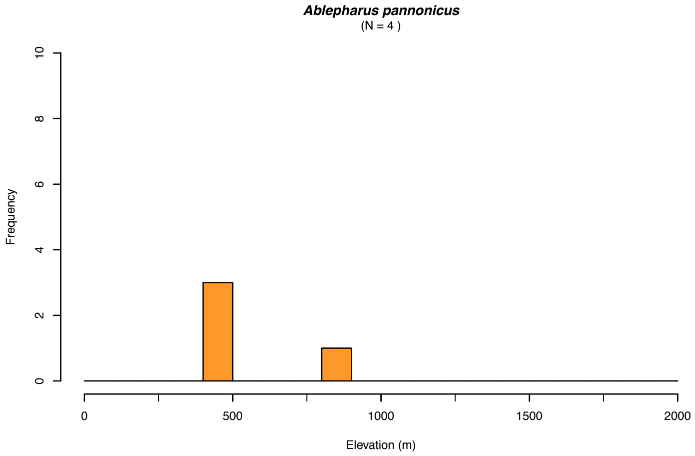

F

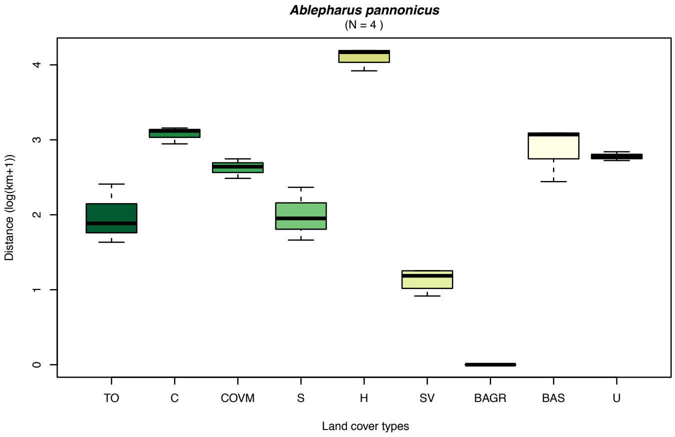

E

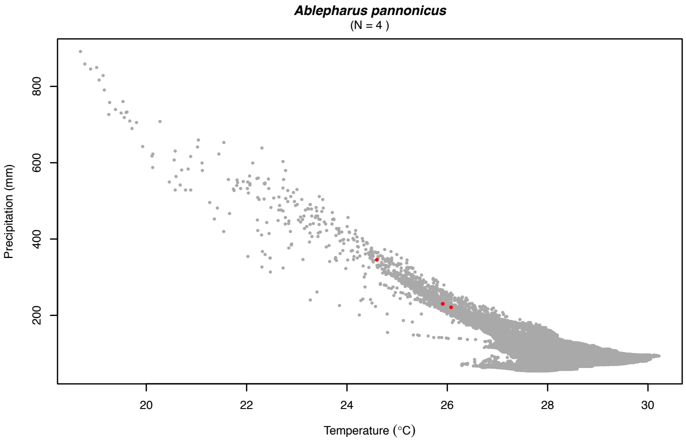

Chalcides ocellatus ocellatus (Forskål, 1775)

S40: Species information

Lizards

Skinks, Scincidae

A

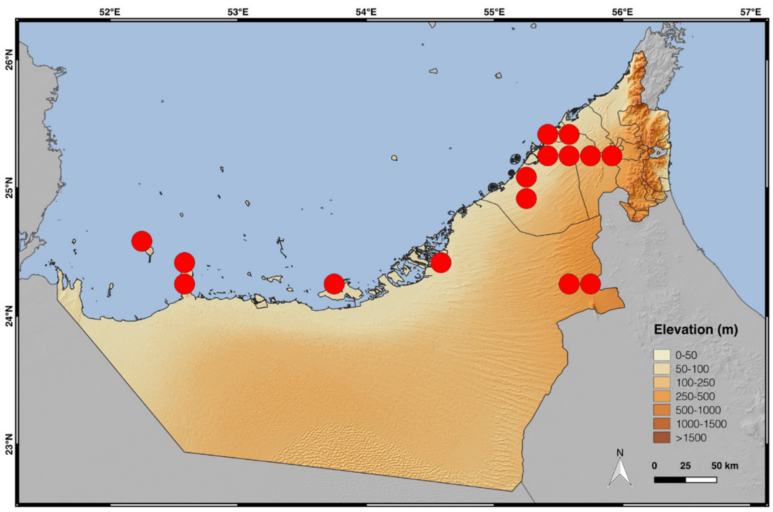

B

Useful information	
IUCN Category	LC/NA
Venomous	NO
Endemic	NO
Origin	Introduced

C

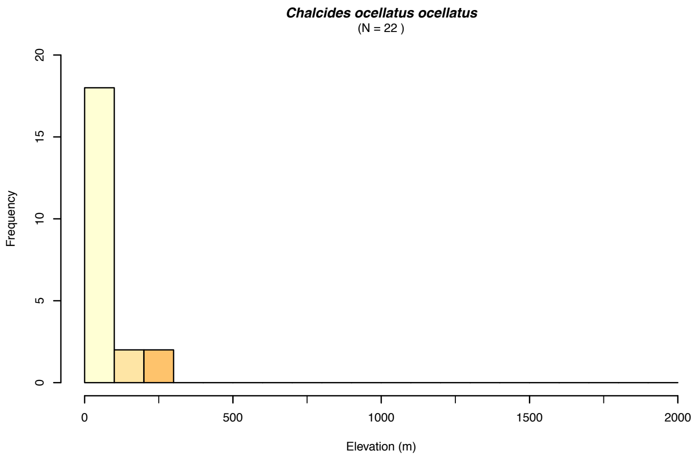

D

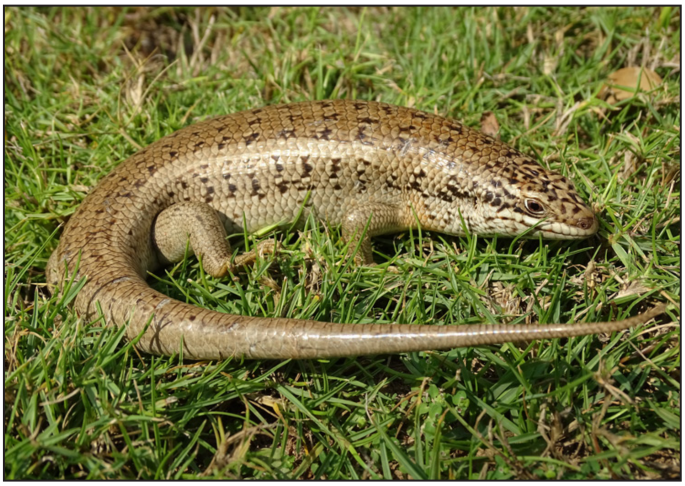

E

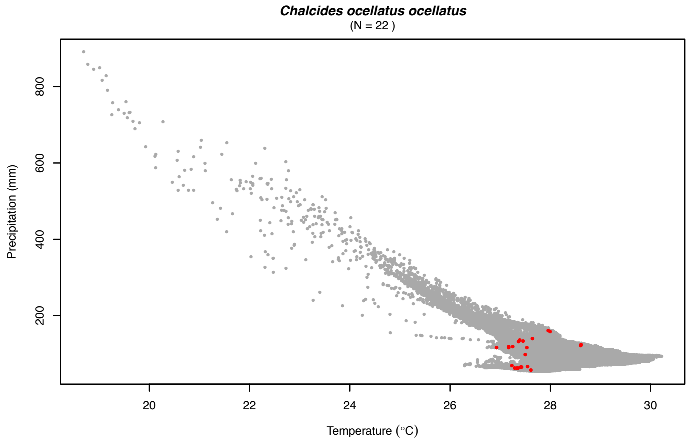

F

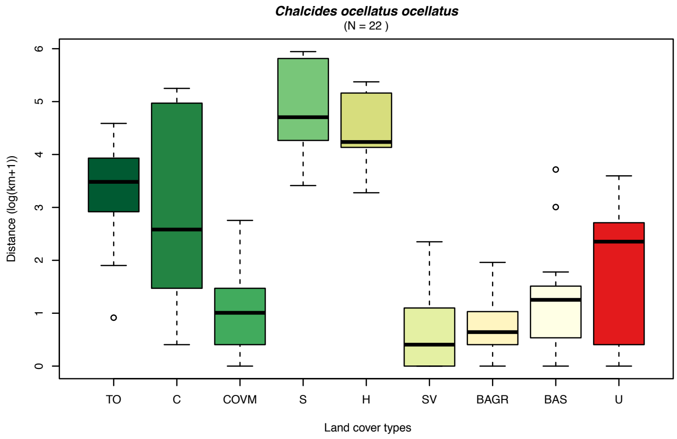

S41: Species information

Lizards
Skinks, Scincidae

Heremites septemtaeniatus (Reuss, 1834)

A

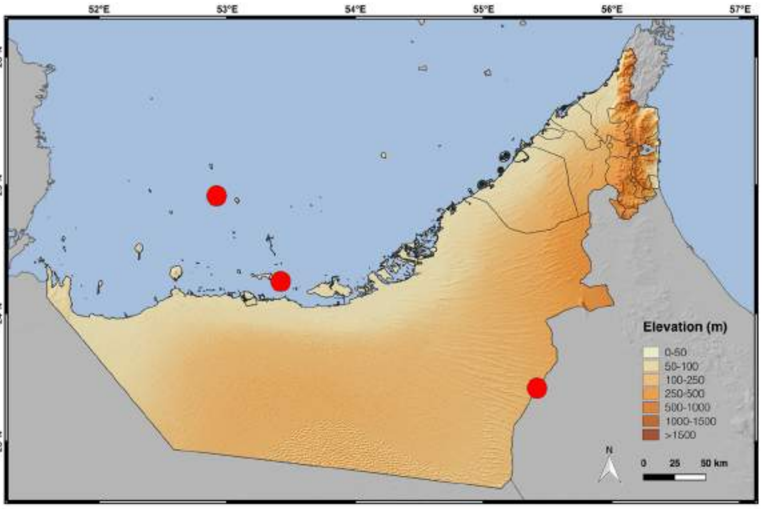

B

Useful information	
IUCN Category	LC/DD
Venomous	NO
Endemic	NO
Origin	Native

D

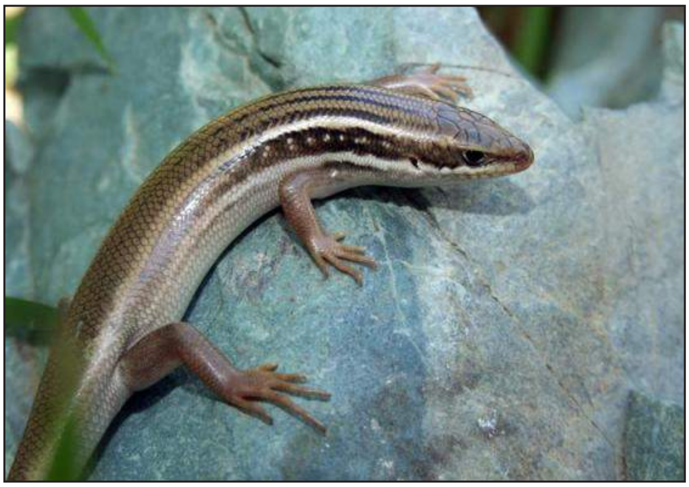

C

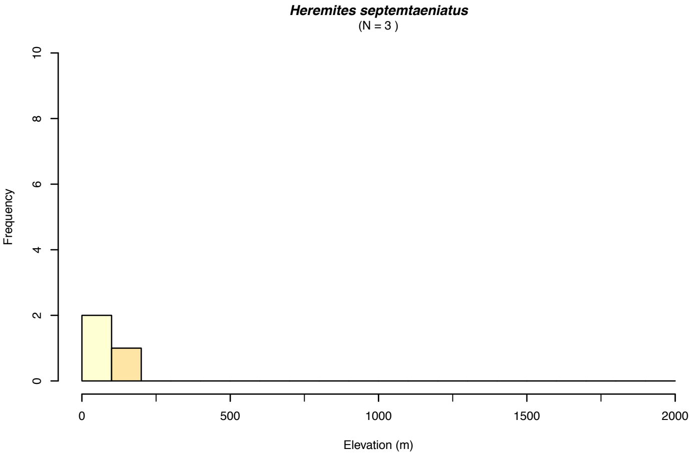

F

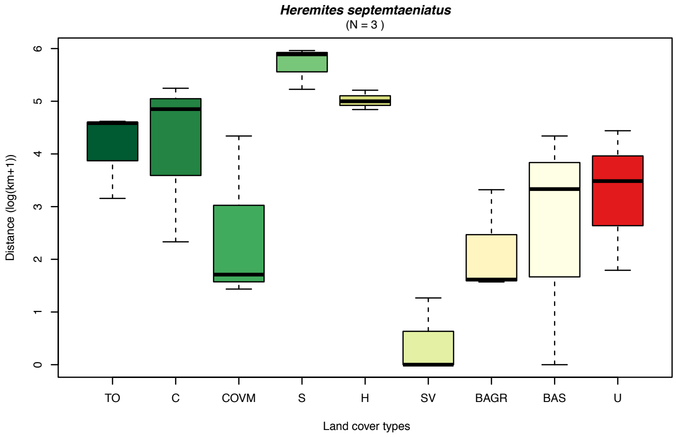

E

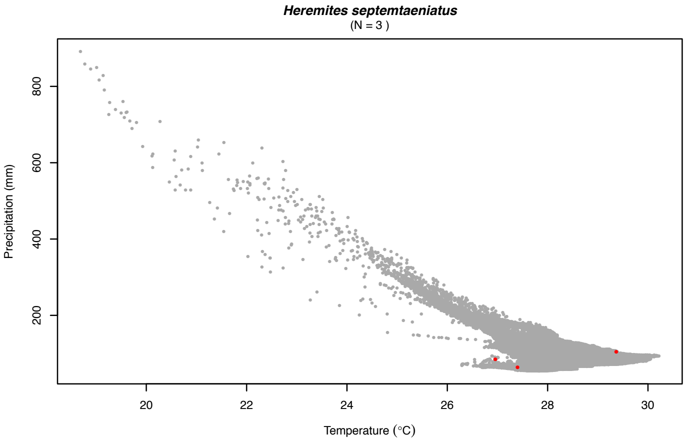

Scincus mitranus
Anderson, 1871

S42: Species information
Lizards
Skinks, Scincidae

A

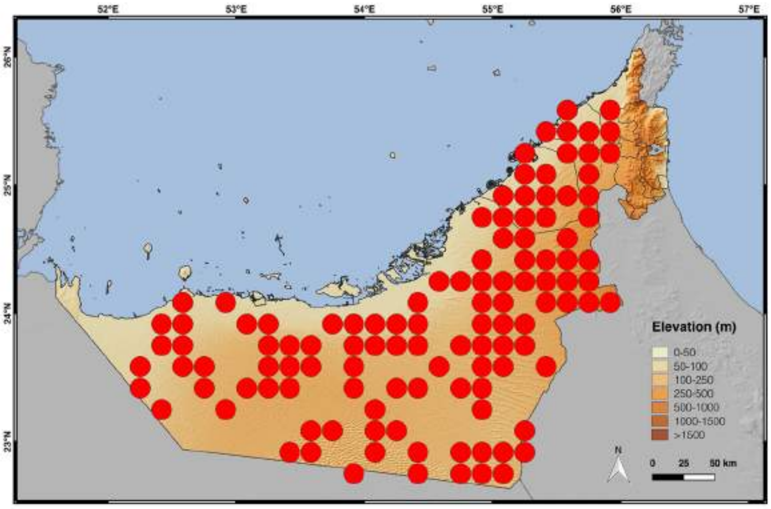

B

Useful information

IUCN Category	LC/LC
Venomous	NO
Endemic	NO
Origin	Native

C

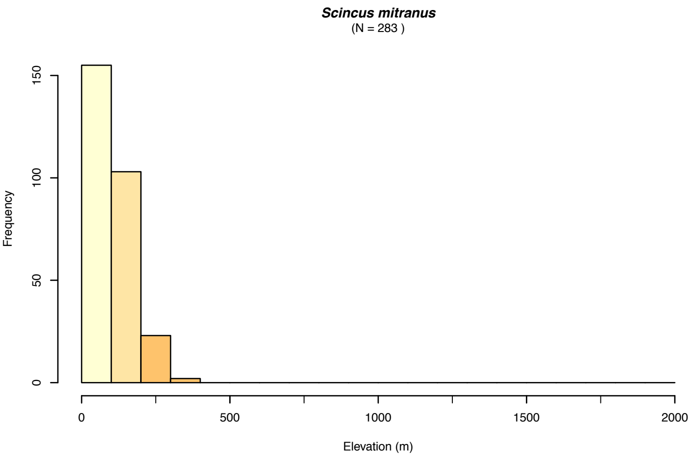

D

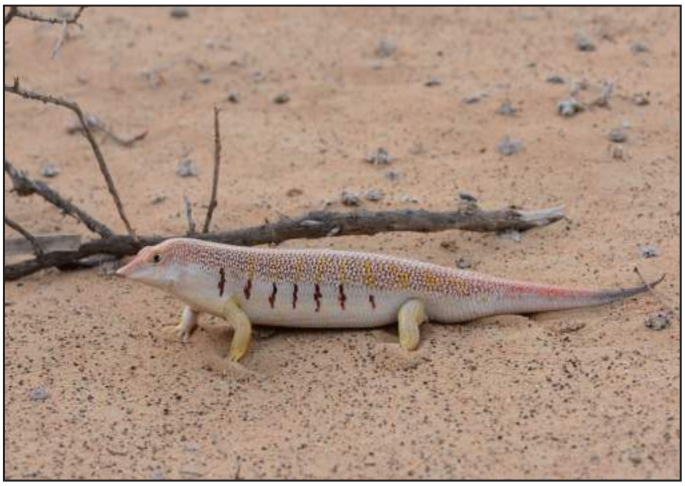

E

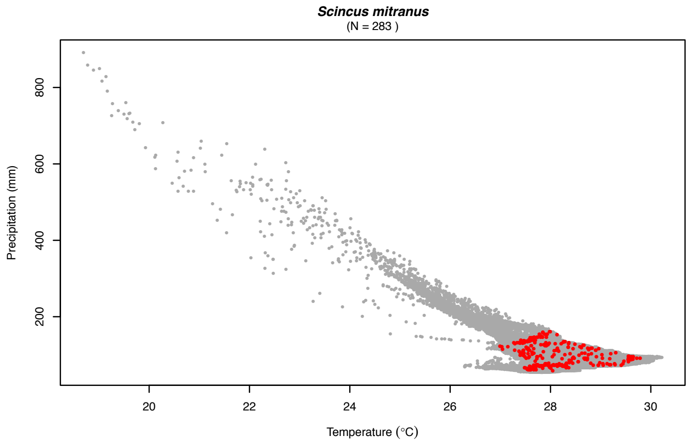

F

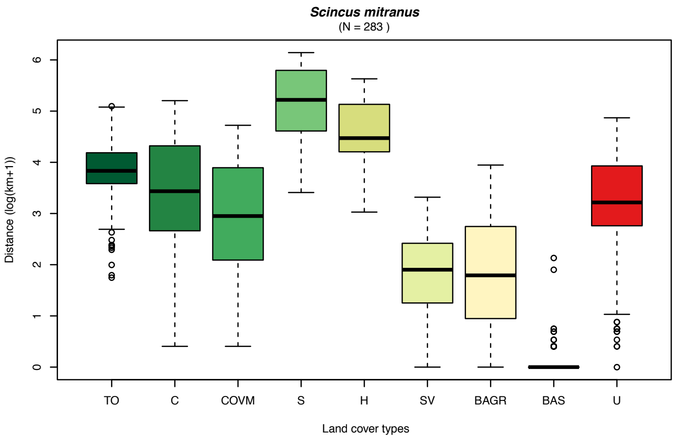

S43: Species information

Lizards

Skinks, Scincidae

Scincus scincus conirostris
Blanford, 1881

A

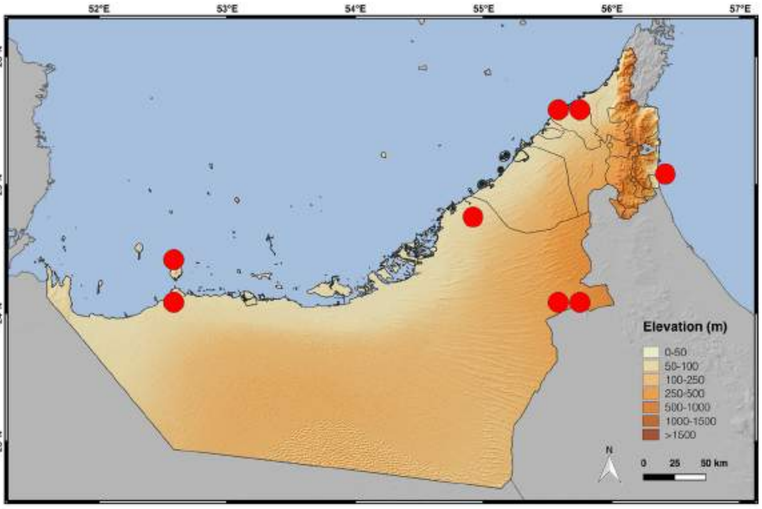

B

Useful information	
IUCN Category	LC/DD
Venomous	NO
Endemic	NO
Origin	Native

D

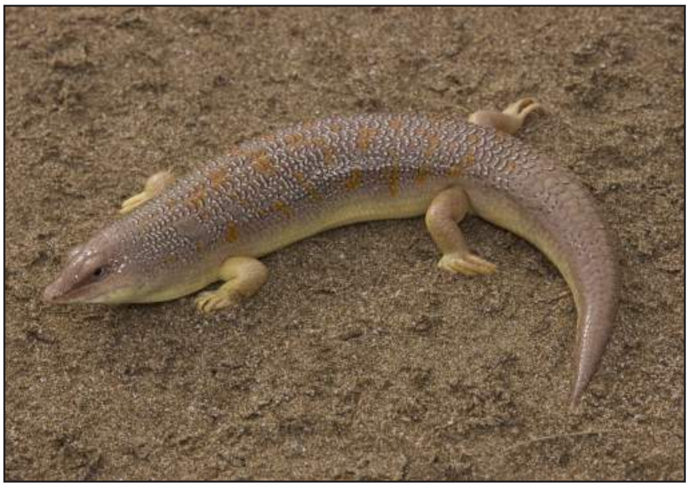

C

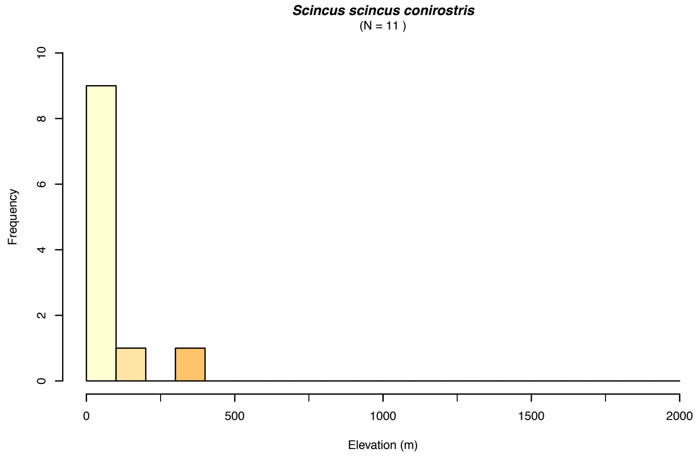

F

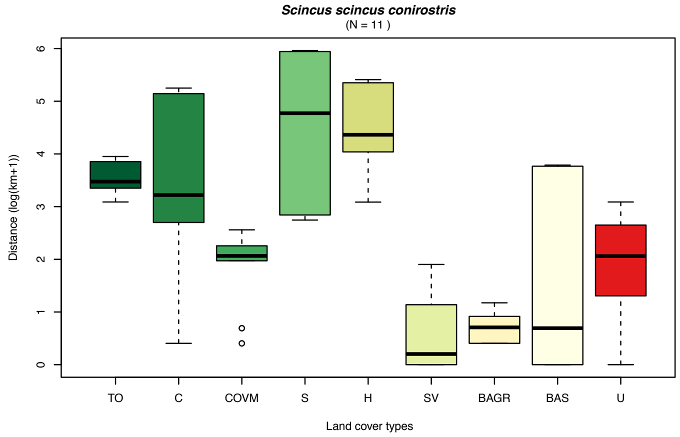

E

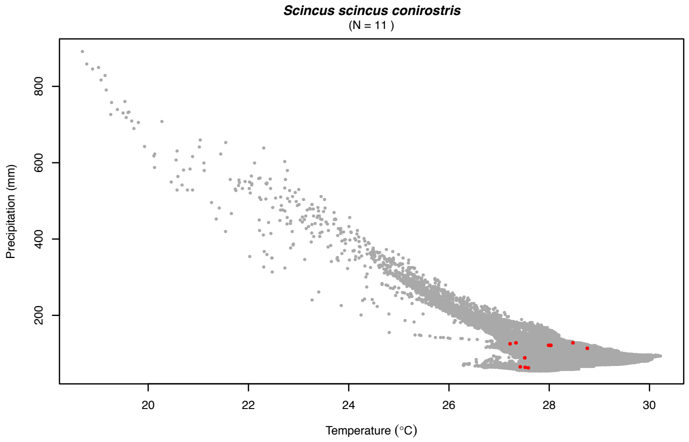

Trachylepis tessellata (Anderson, 1895)

S44: Species information

Lizards

Skinks, Scincidae

A

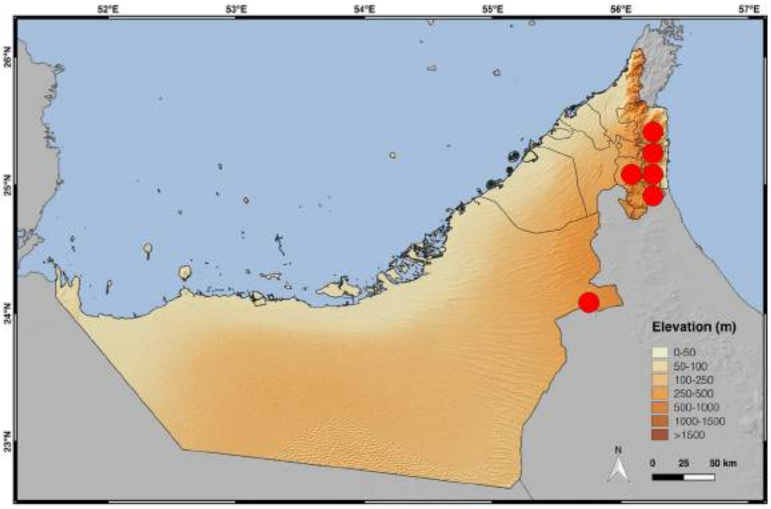

B

Useful information	
IUCN Category	LC/LC
Venomous	NO
Endemic	NO
Origin	Native

C

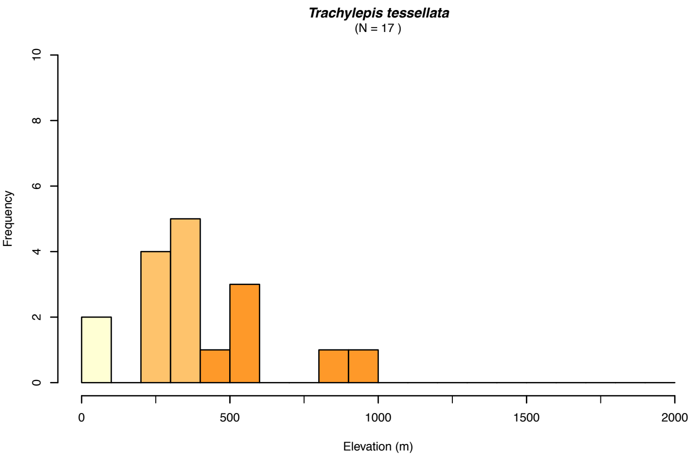

D

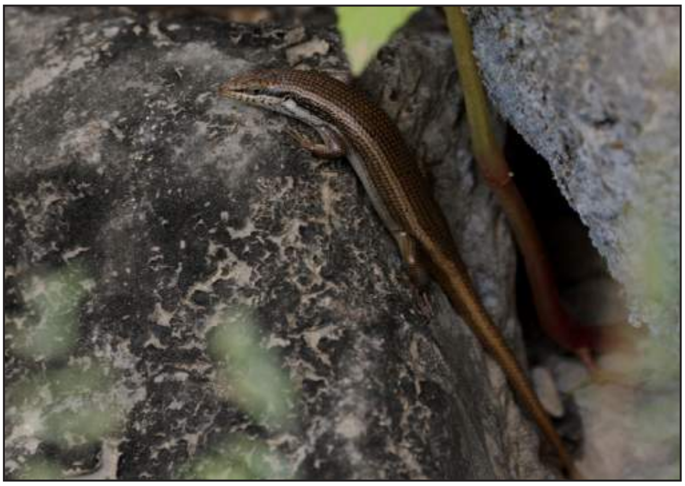

E

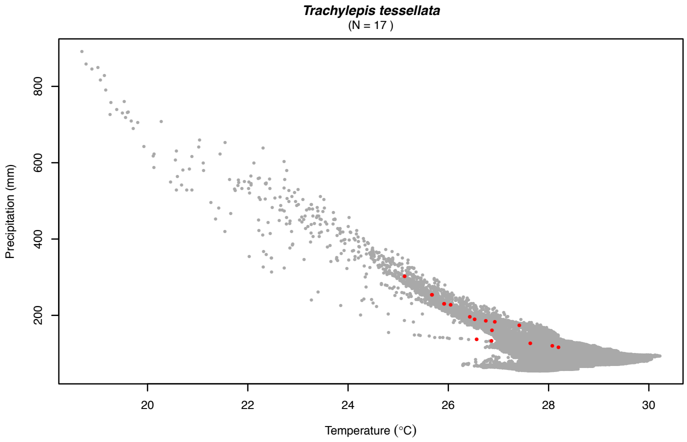

F

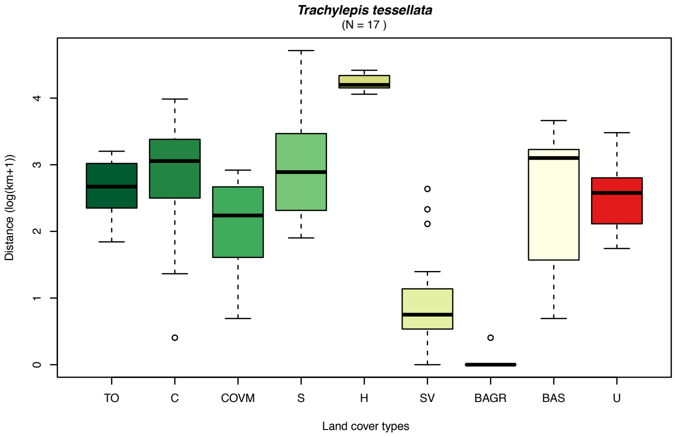

S45: Species information

Lizards
Amphisbaenids, Trogonophidae

Diplometopon zarudnyi
Nikolsky, 1907

A

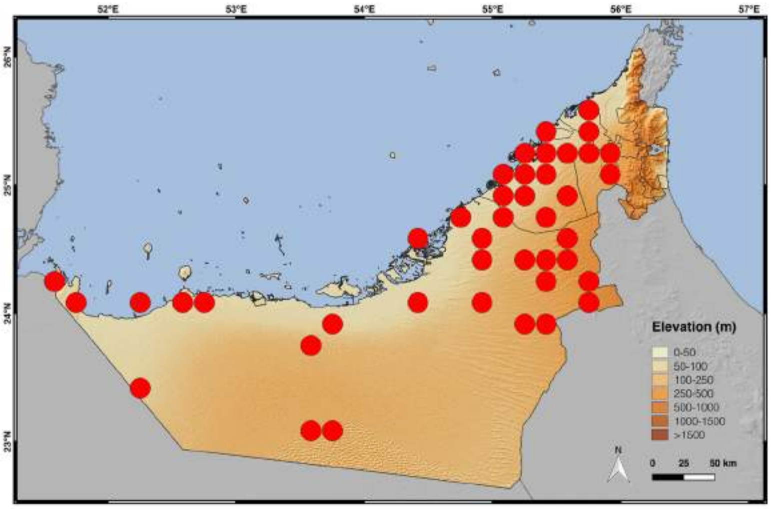

B

Useful information	
IUCN Category	LC/LC
Venomous	NO
Endemic	NO
Origin	Native

D

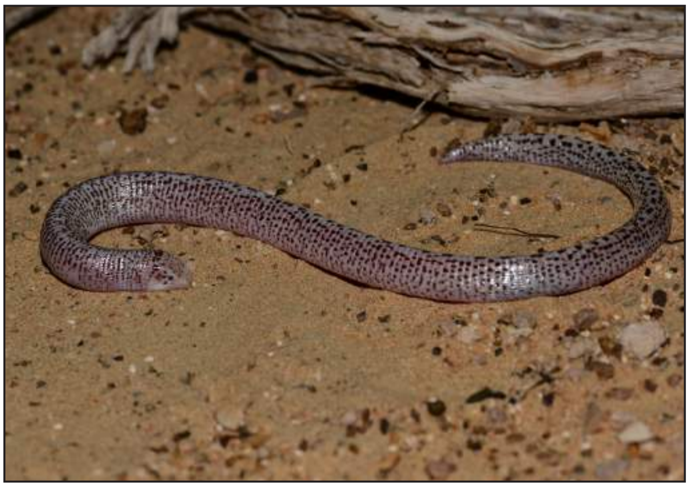

C

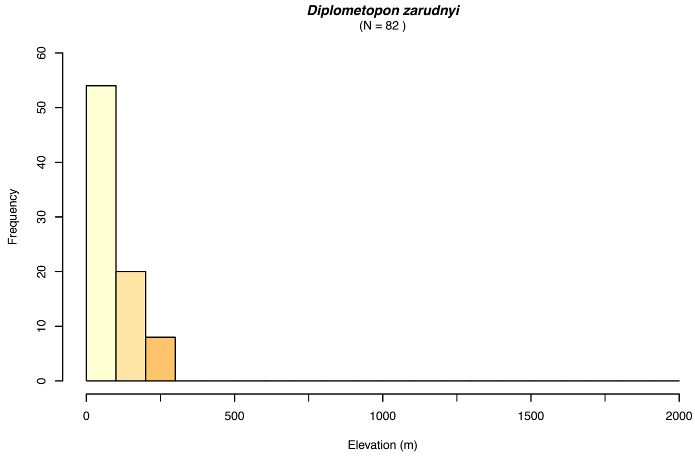

F

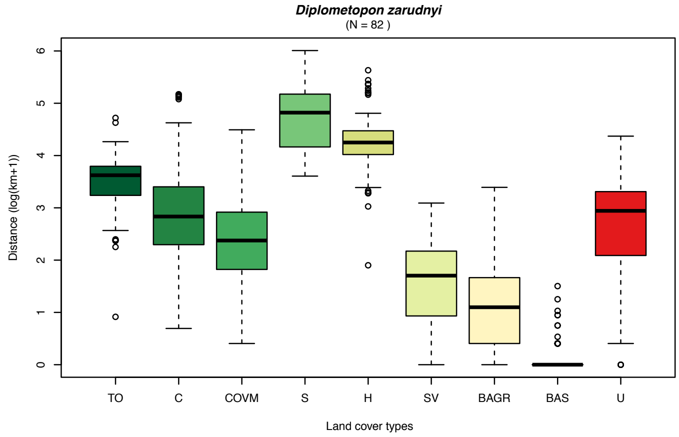

E

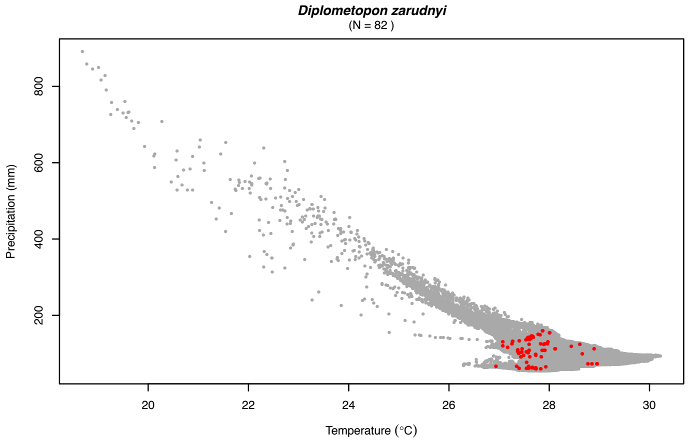

Varanus griseus griseus (Daudin, 1803)

S46: Species information

Lizards
Varanids, Varanidae

A

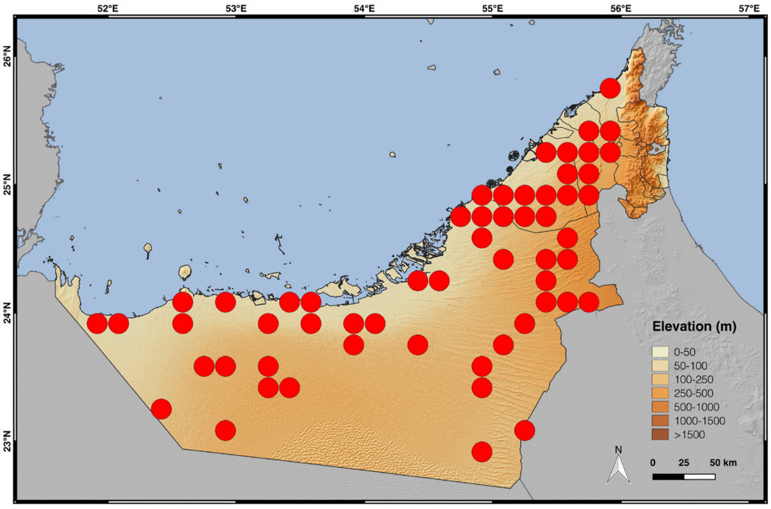

B

Useful information	
IUCN Category	LC/LC
Venomous	NO
Endemic	NO
Origin	Native

C

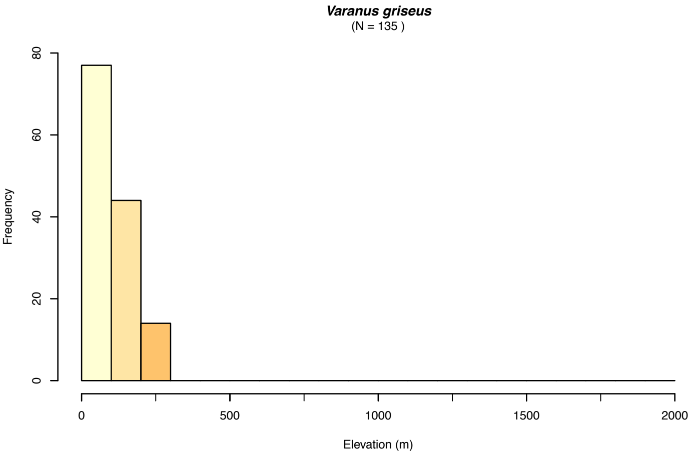

D

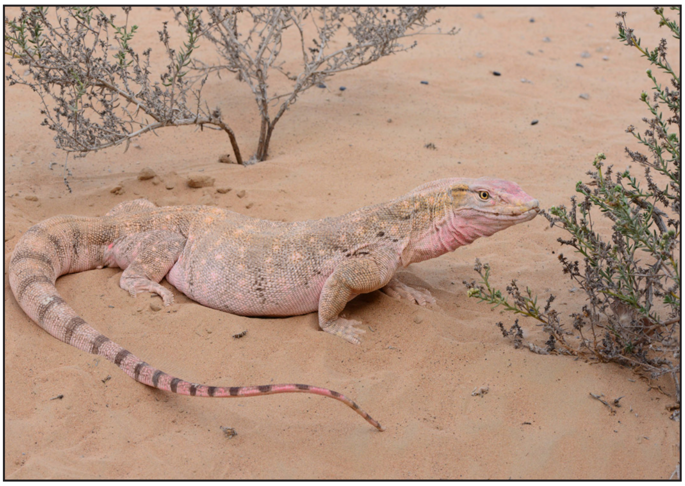

E

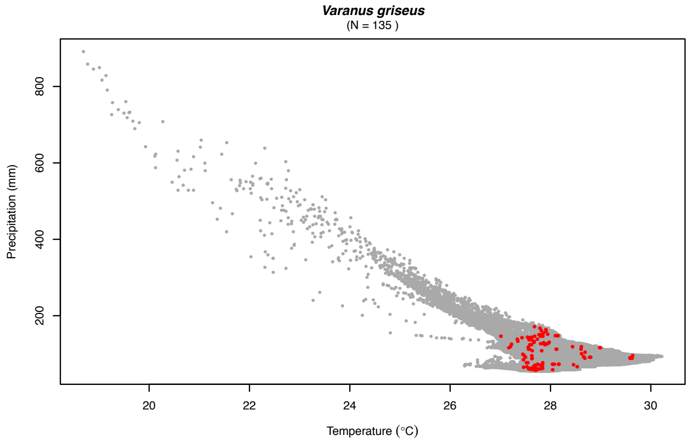

F

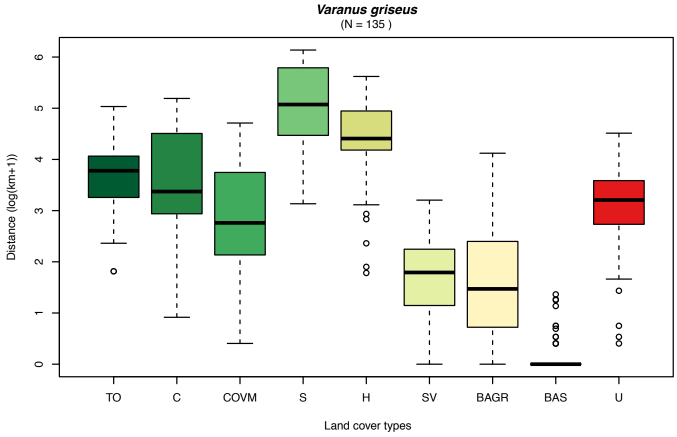

S47: Species information

Snakes

Boidae

Eryx jayakari
Boulenger, 1888

A

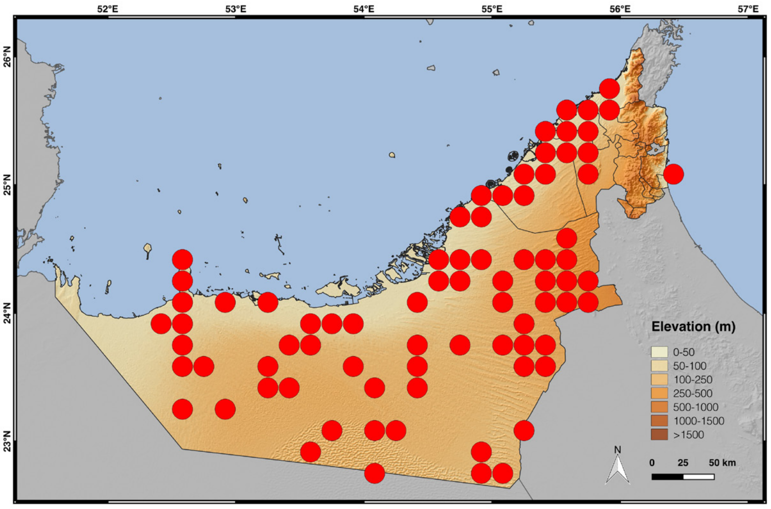

B

Useful information	
IUCN Category	LC/LC
Venomous	NO
Endemic	NO
Origin	Native

D

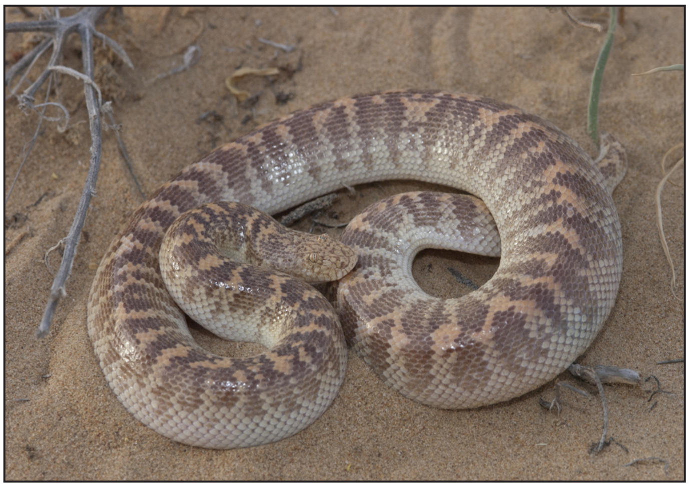

C

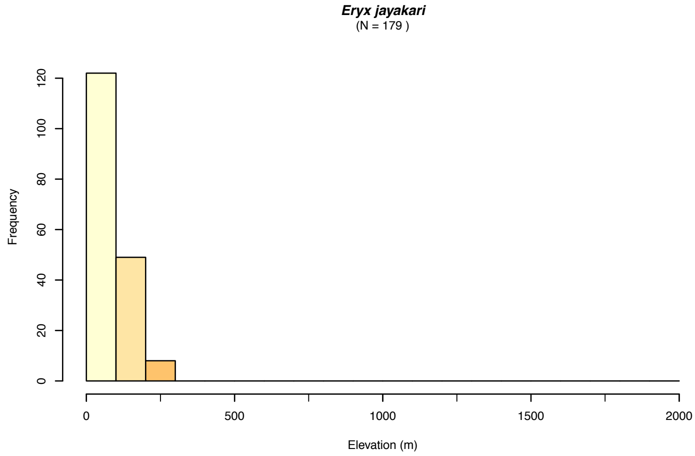

F

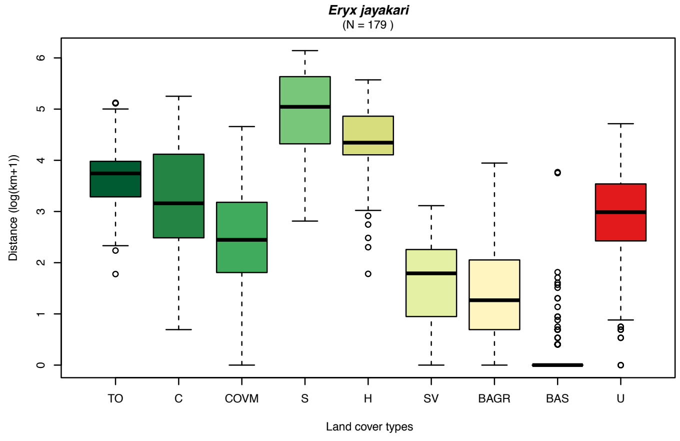

E

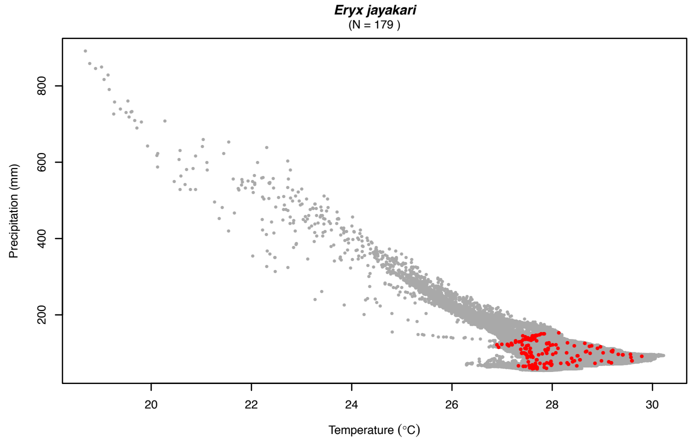

Lizards
Agamids
Geckos
Lacertids
Skinkis
Amphisbaenids
Varanids
Snakes
Boidae
Colubridae
Lamprophiidae
Leptotyphlopidae
Typhlopidae
Viperidae

Lytorhynchus diadema diadema (Duméril, Bibron & Duméril, 1854)

S48: Species information

Snakes
Colubridae

A

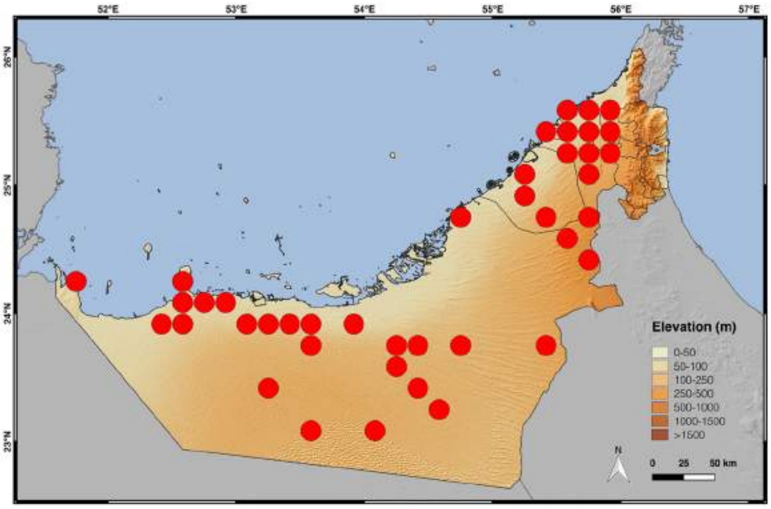

B

Useful information

IUCN Category	LC/LC
Venomous	NO
Endemic	NO
Origin	Native

C

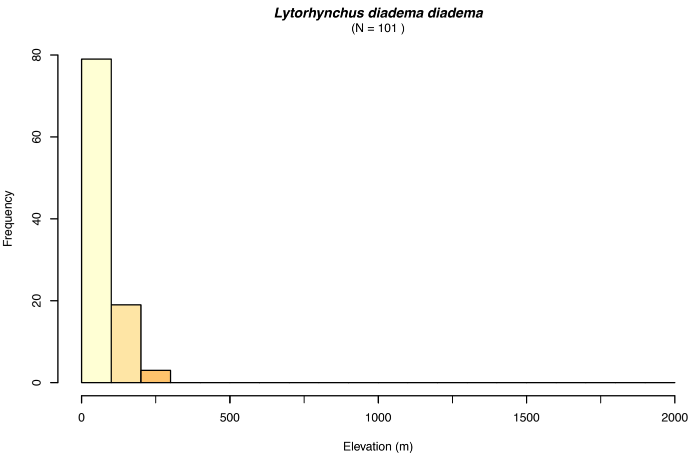

D

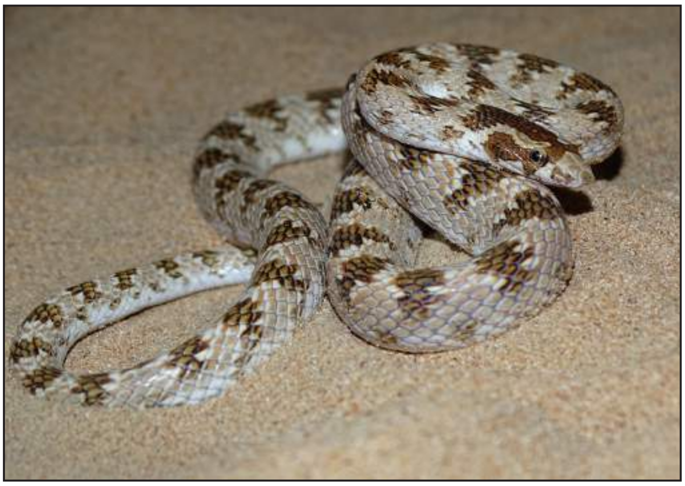

E

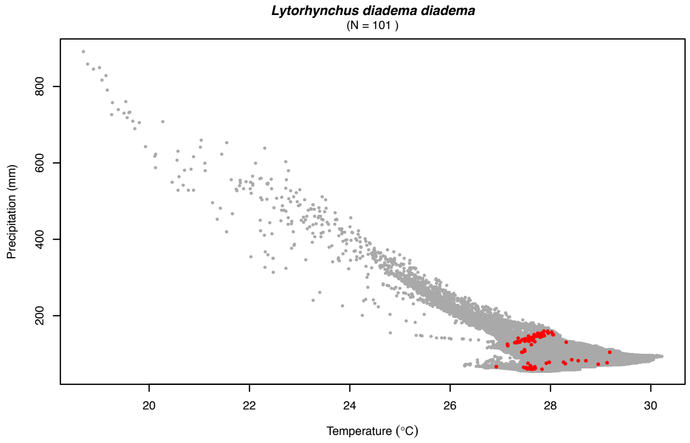

F

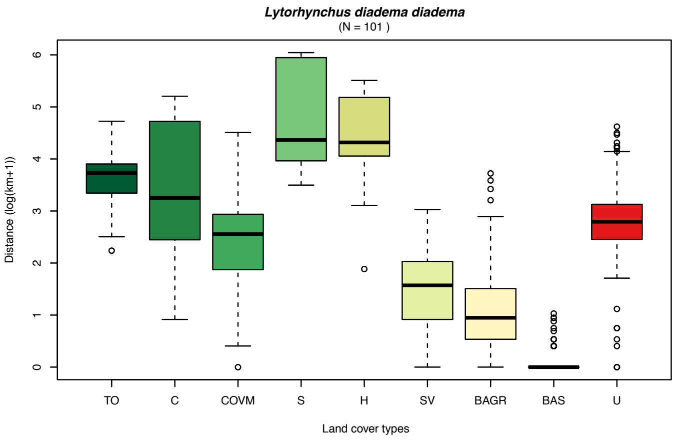

S49: Species information

Snakes

Colubridae

Platyceps rhodorachis rhodorachis (Jan & Filippi, 1865)

A

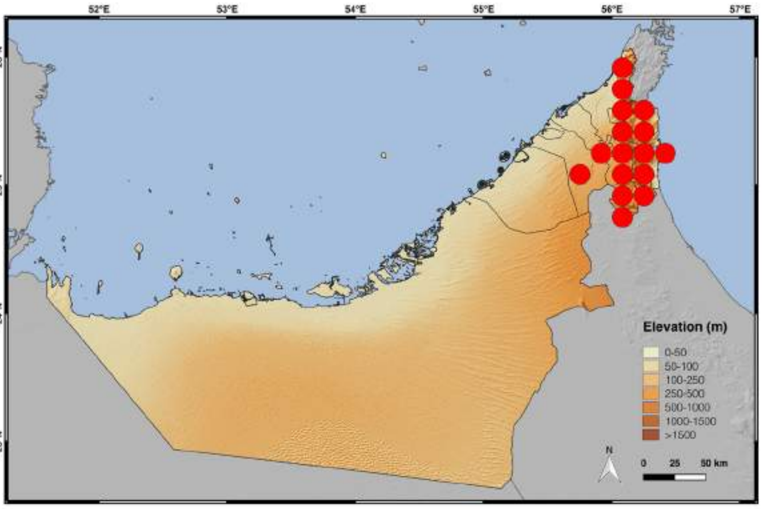

B

Useful information

IUCN Category	LC/LC
Venomous	NO
Endemic	NO
Origin	Native

D

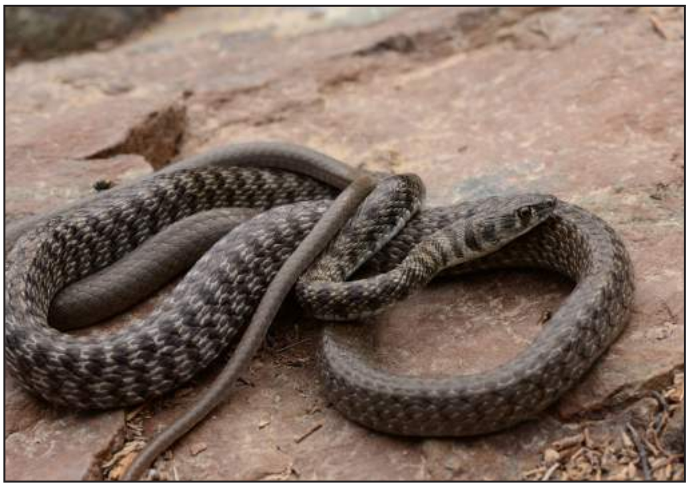

C

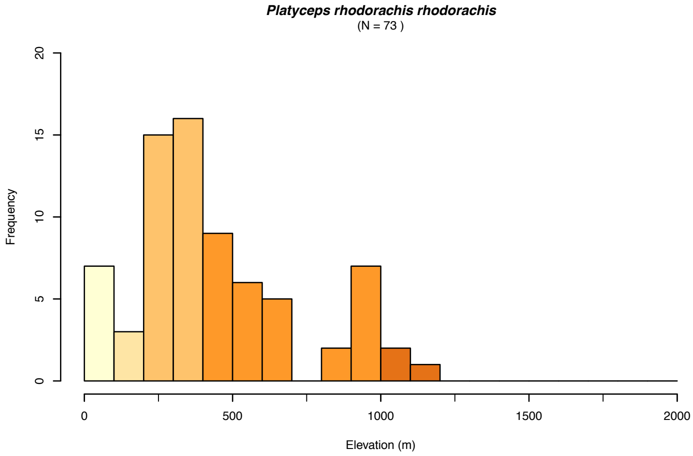

F

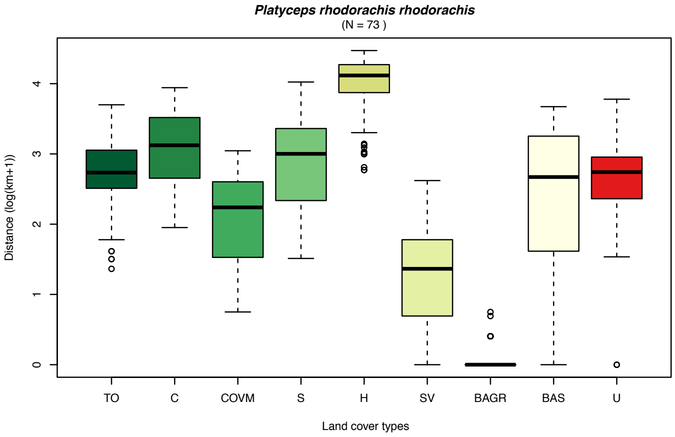

E

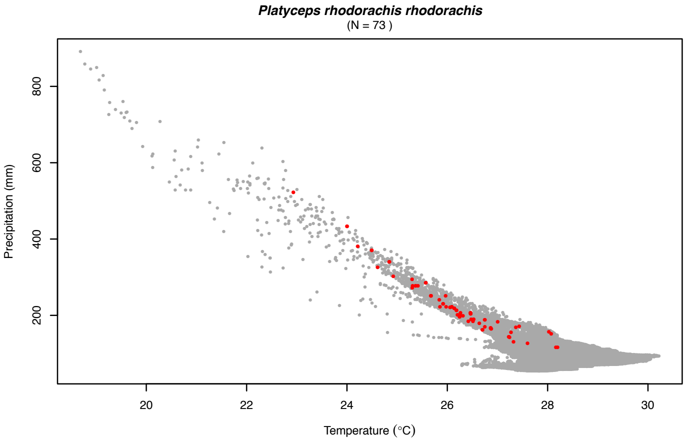

Platyceps ventromaculatus (Gray, 1834)

S50: Species information

Snakes

Colubridae

A

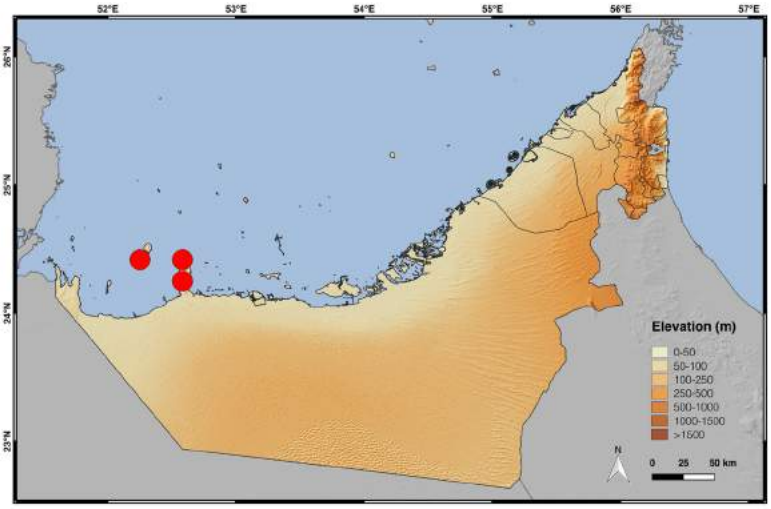

B

Useful information	
IUCN Category	LC/VU
Venomous	NO
Endemic	NO
Origin	Native

C

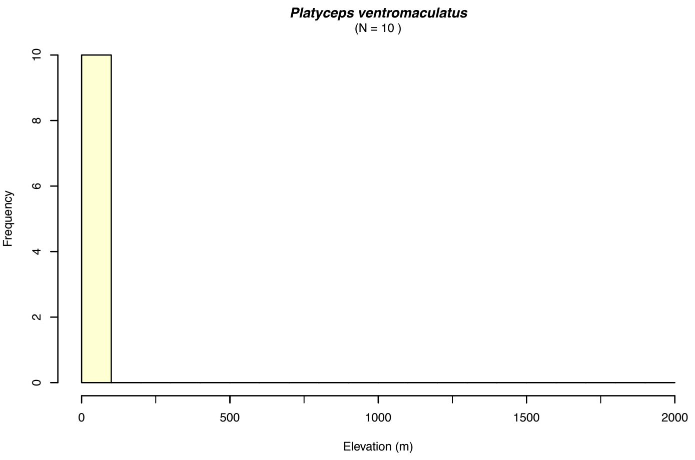

D

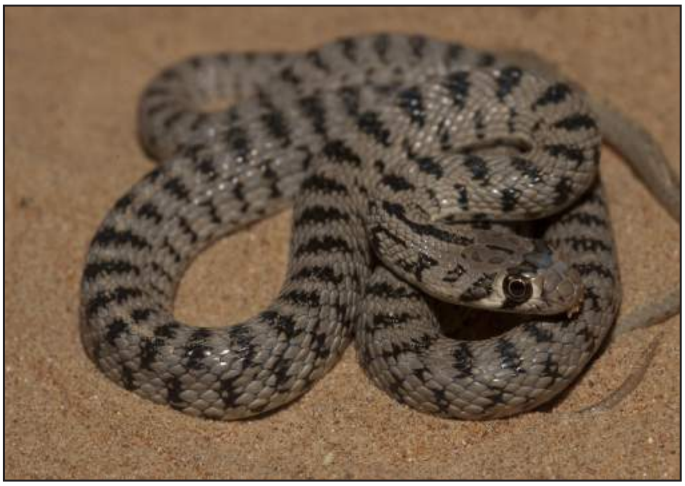

E

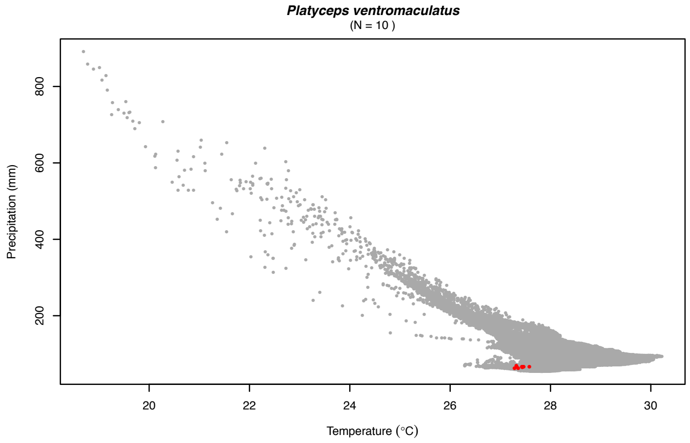

F

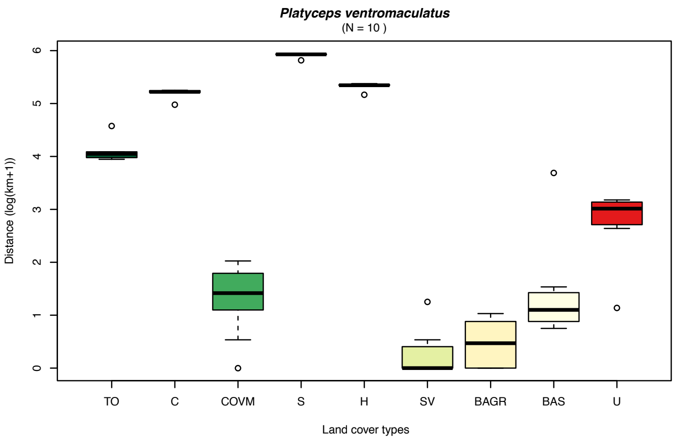

S51: Species information

Snakes
Colubridae

Spalerosophis diadema cliffordii
(Schlegel, 1837)

A

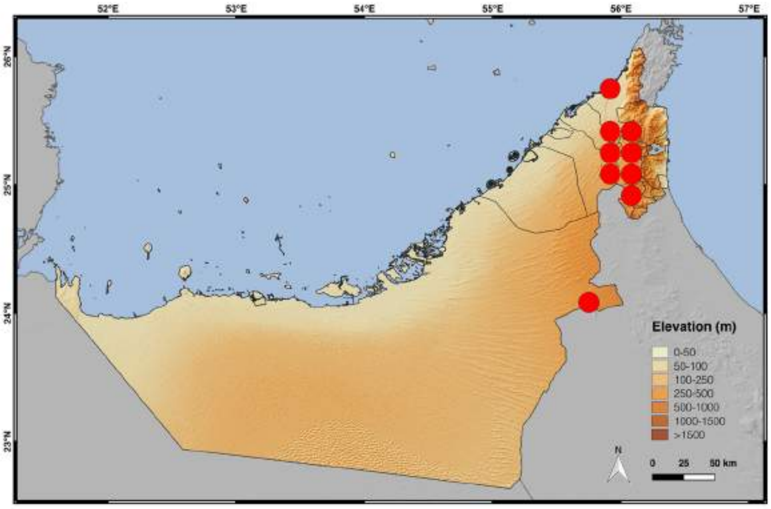

B

Useful information	
IUCN Category	LC/LC
Venomous	NO
Endemic	NO
Origin	Native

D

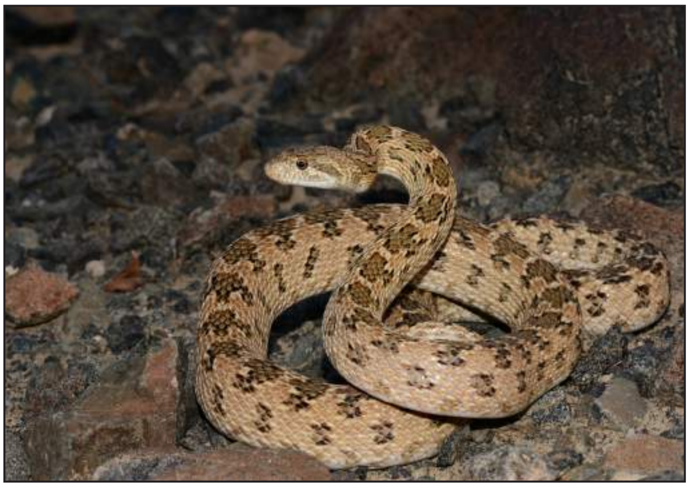

C

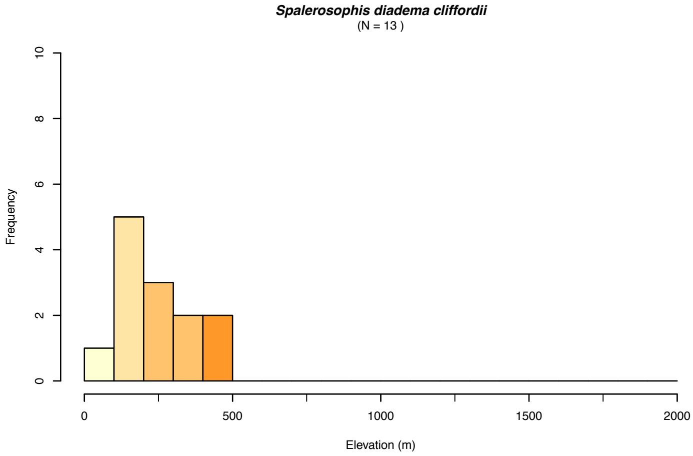

F

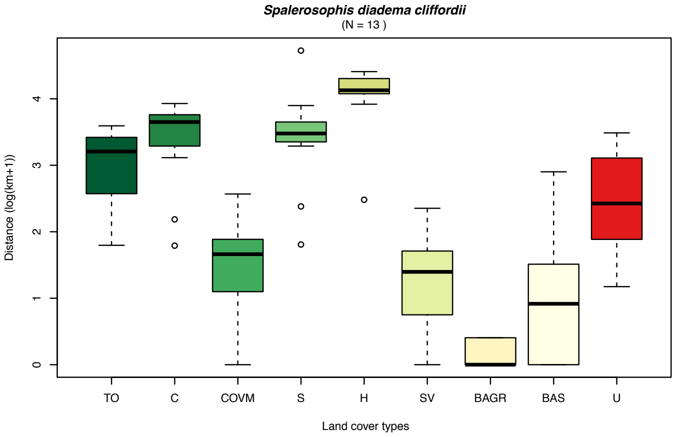

E

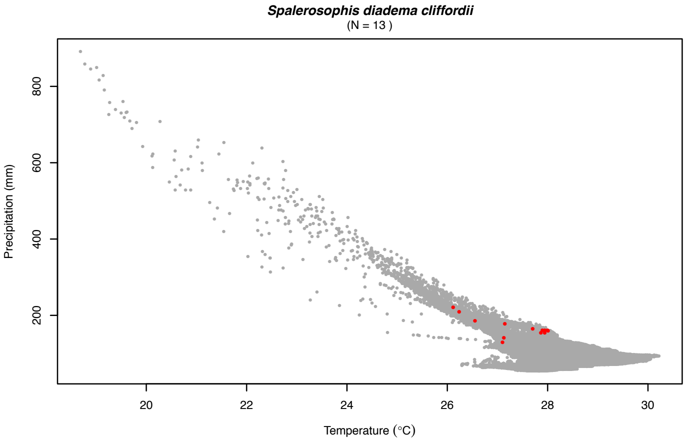

Lizards Agamids Geckos Lacertids Skinks Amphisbaenids Varanids Snakes Boidae Colubridae Lamprophiidae Leptotyphlopidae Typhlopidae Viperidae

Telescopus dhara dhara (Forskål, 1775)

S52: Species information

Snakes
Colubridae

A

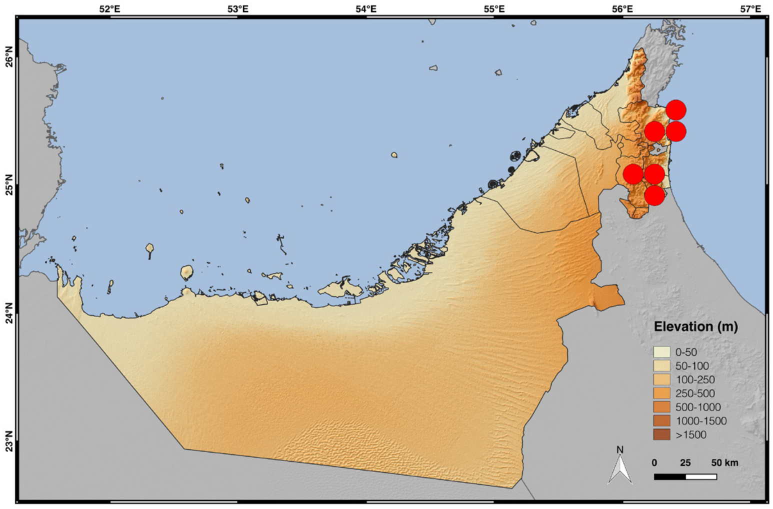

B

Useful information	
IUCN Category	LC/LC
Venomous	Slightly
Endemic	NO
Origin	Native

C

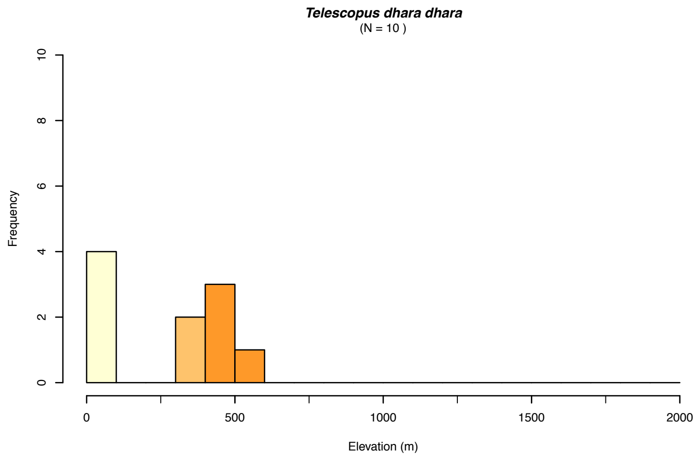

D

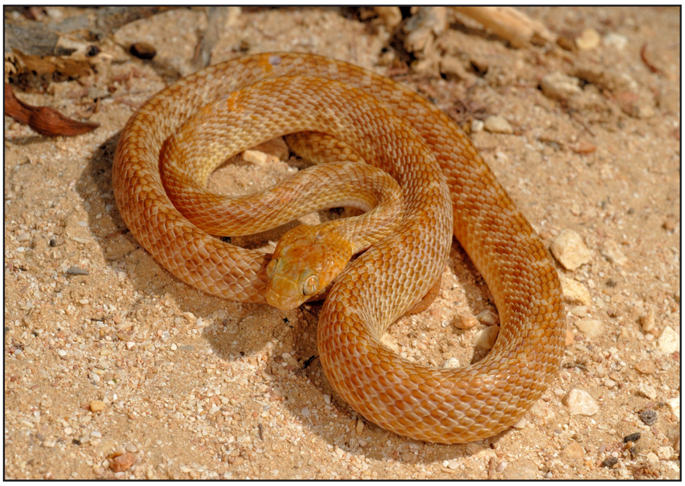

E

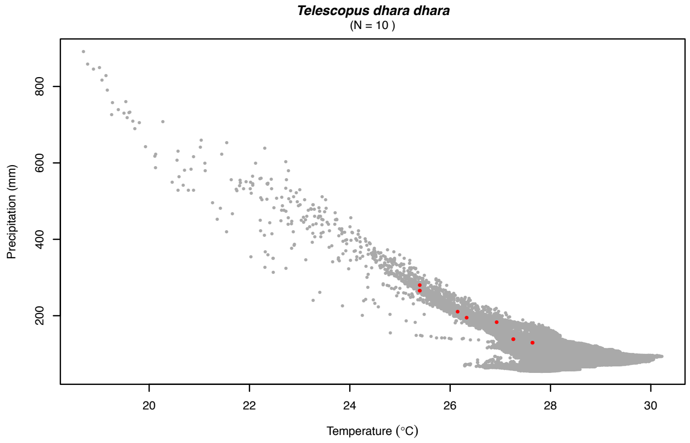

F

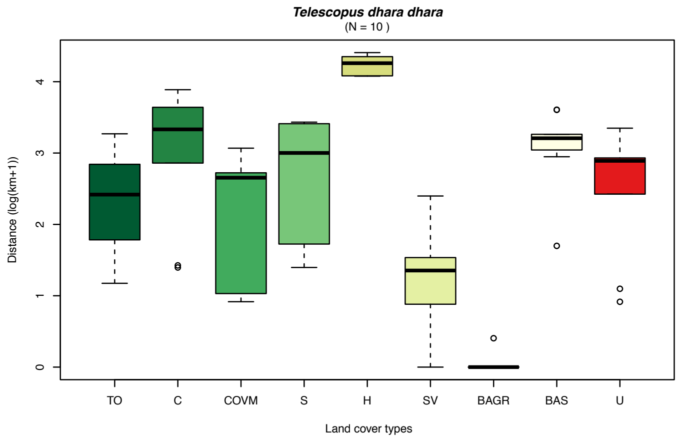

S53: Species information

Snakes

Lamprophidae

Psammophis schokari (Forskål, 1775)

A

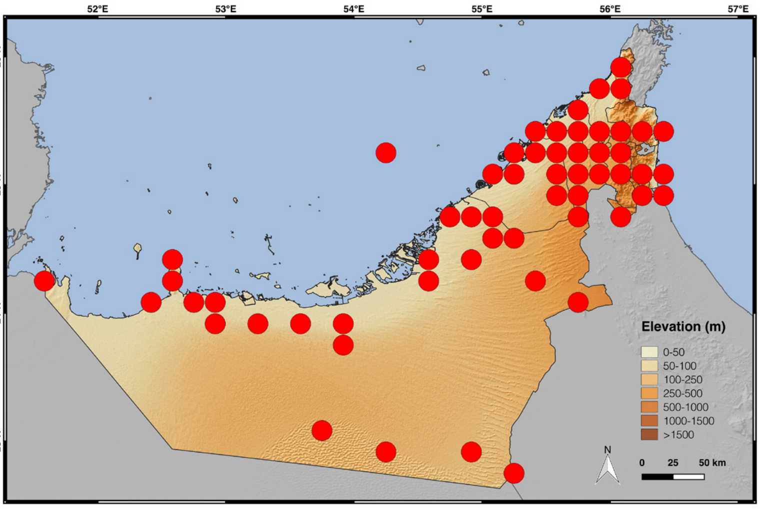

B

Useful information	
IUCN Category	LC/LC
Venomous	Slightly
Endemic	NO
Origin	Native

D

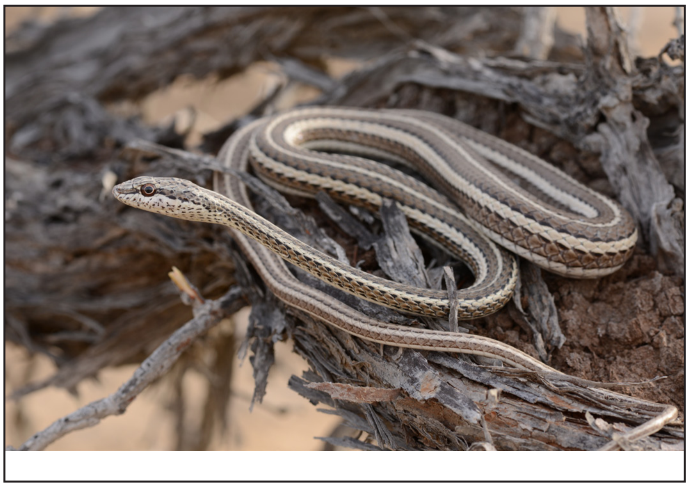

C

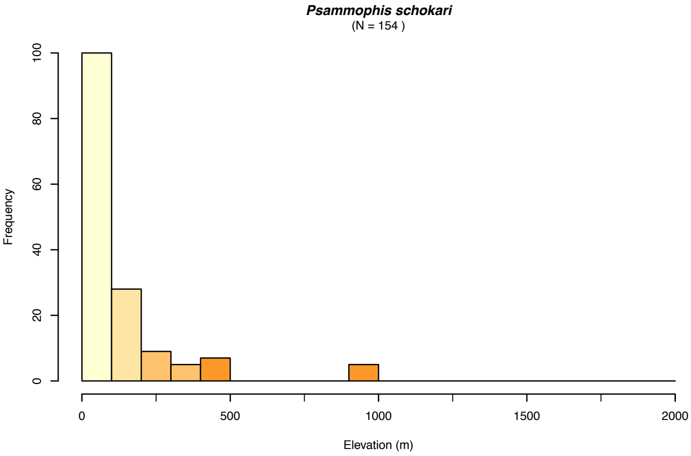

F

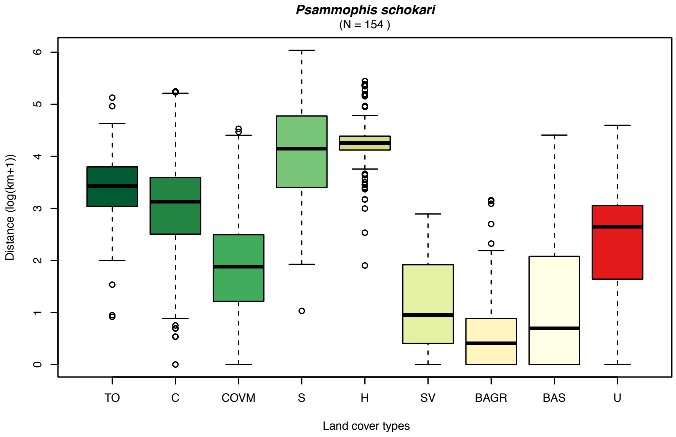

E

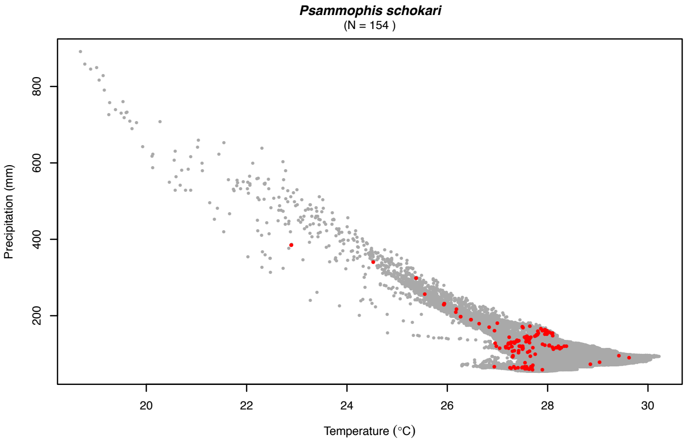

Lizards
Agamids
Geckos
Lacertids
Skinkis
Amphisbaenids
Varanids
Snakes
Boidae
Colubridae
Lamprophiidae
Leptotyphlopidae
Typhlopidae
Viperidae

Rhagerhis moilensis (Reuss, 1834)

S54: Species information
Snakes
Lamprophiidae

A

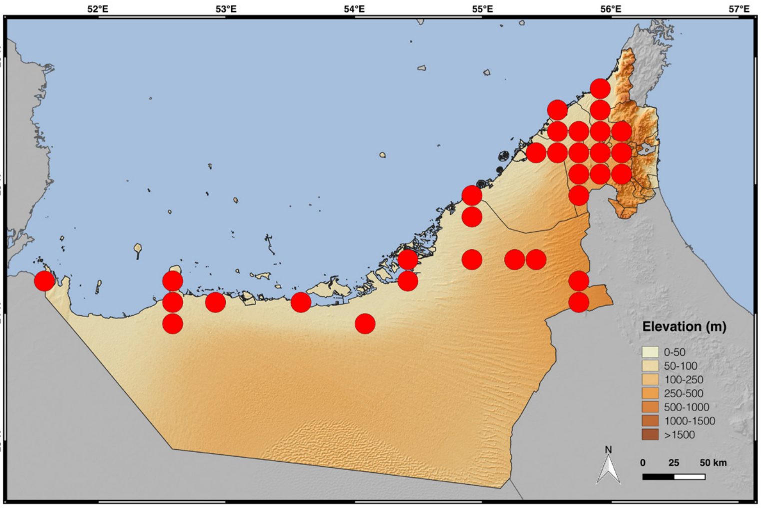

B

Useful information

IUCN Category	LC/LC
Venomous	Slightly
Endemic	NO
Origin	Native

C

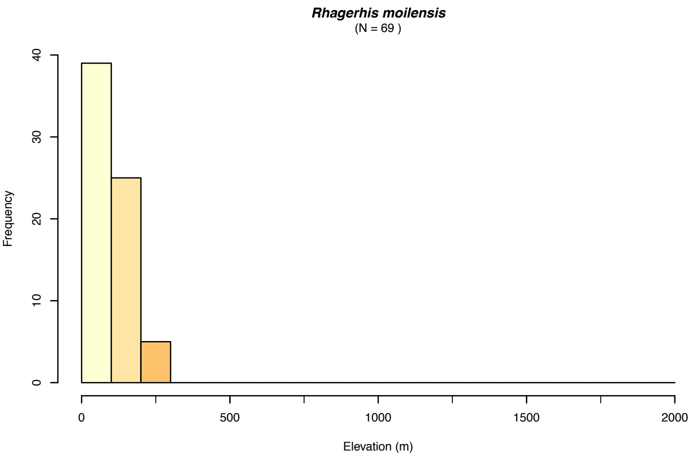

D

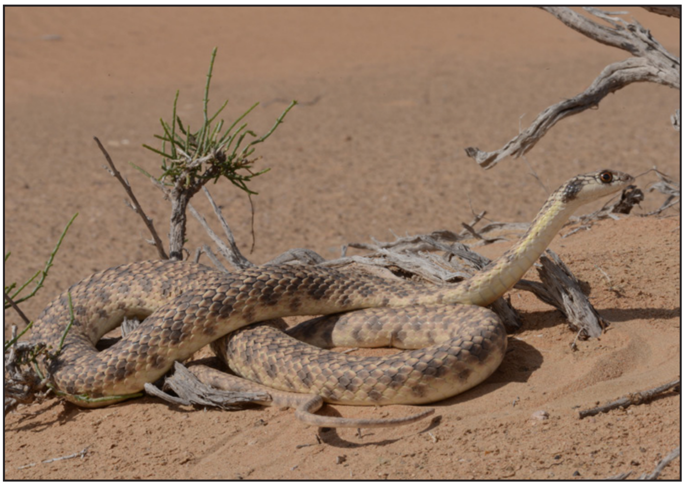

E

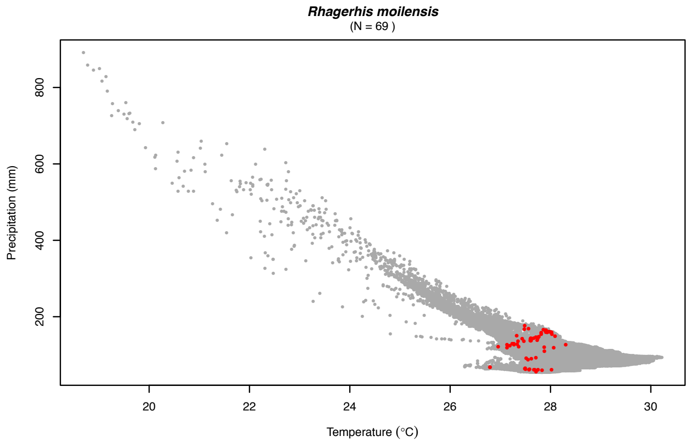

F

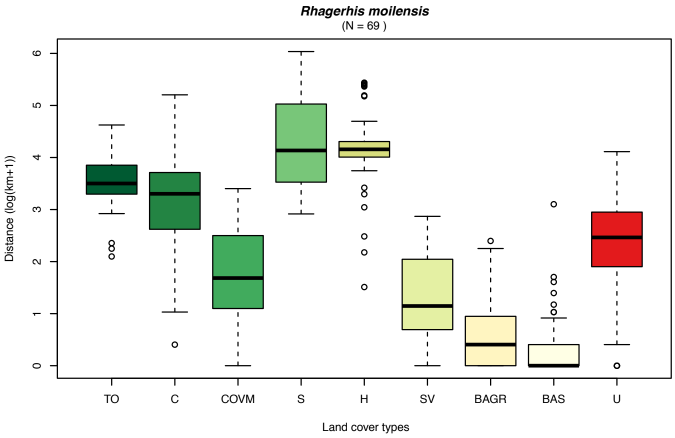

S55: Species information

Snakes

Leptotyphlopidae

Myriopholis macrorhyncha (Jan, 1860)

A

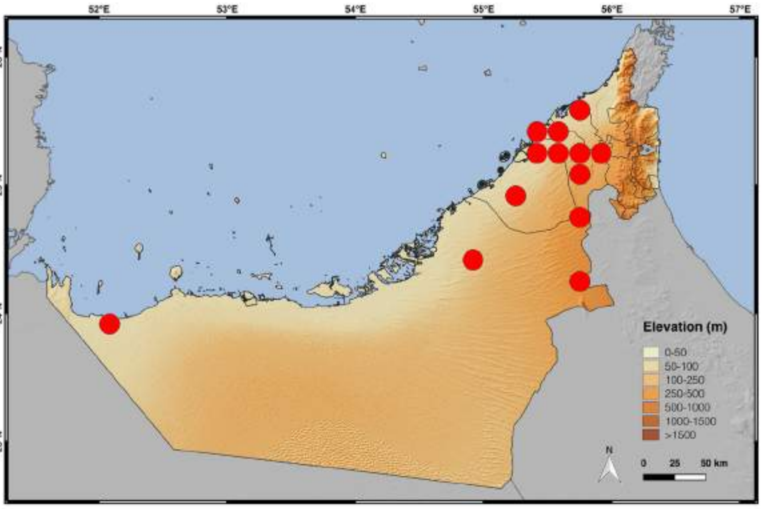

B

Useful information	
IUCN Category	LC/LC
Venomous	NO
Endemic	NO
Origin	Native

D

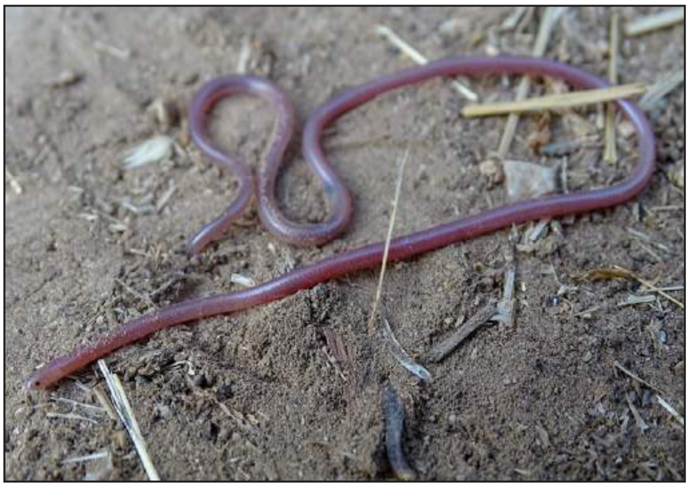

C

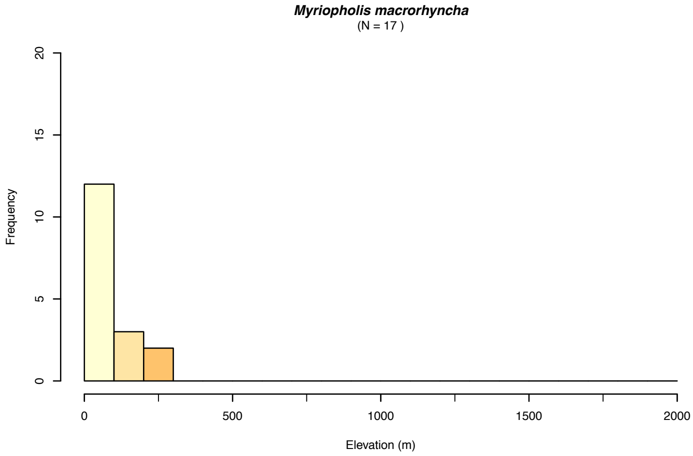

F

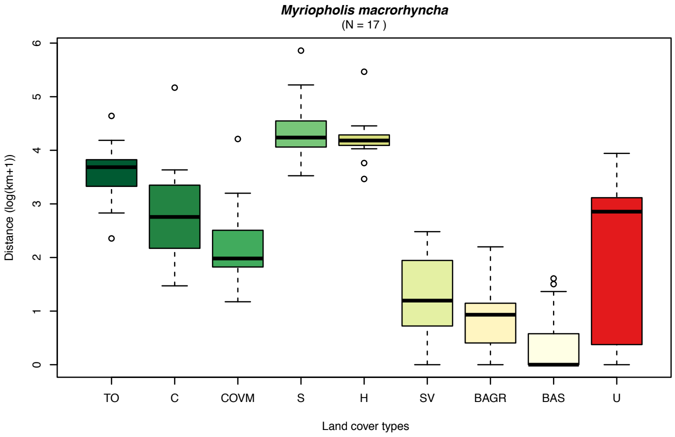

E

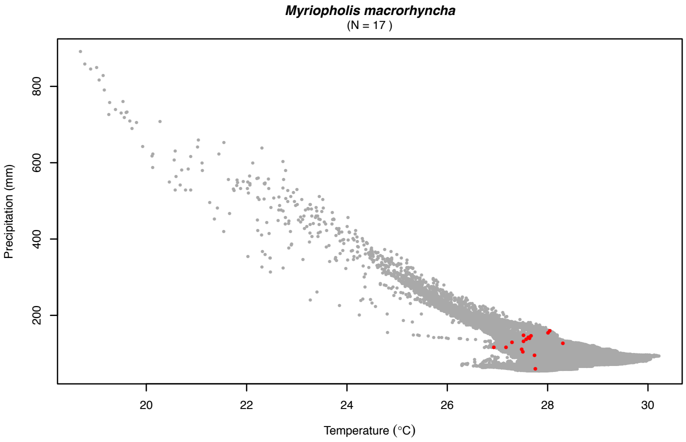

Lizards Agamids Geckos Lacertids Skinks Amphisbaenids Varanids Snakes Boidae Colubridae Lamprophiidae Leptotyphlopidae Typhlopidae Viperidae

Indotyphlops braminus (Daudin, 1803)

S56: Species information

Snakes

Typhlopidae

A

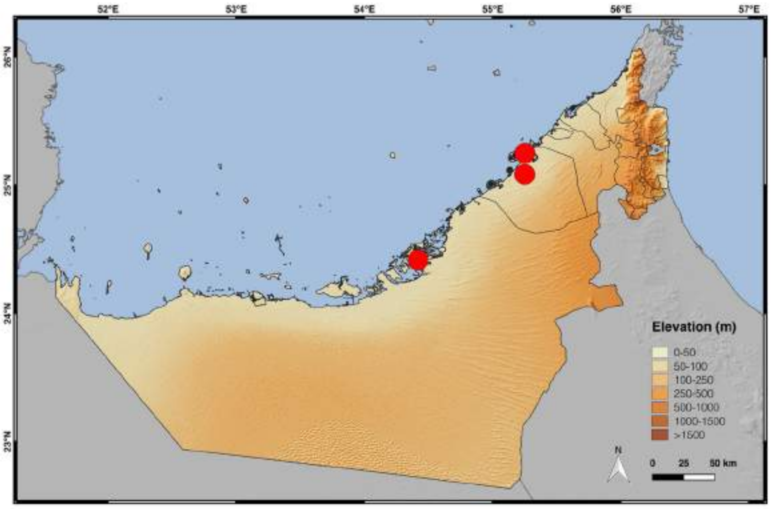

B

Useful information

IUCN Category	LC/NA
Venomous	NO
Endemic	NO
Origin	Introduced

C

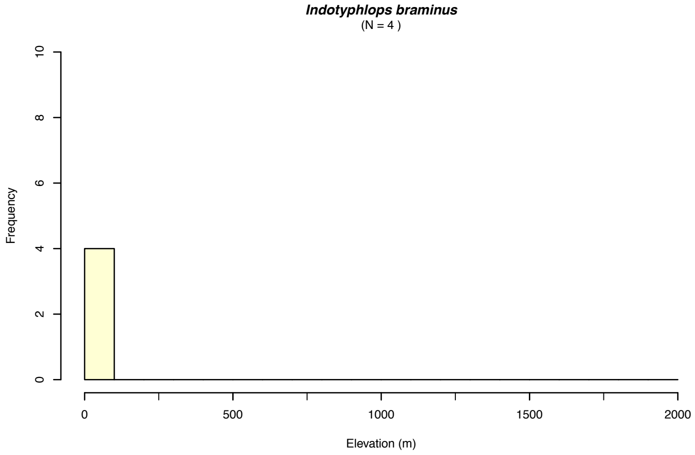

D

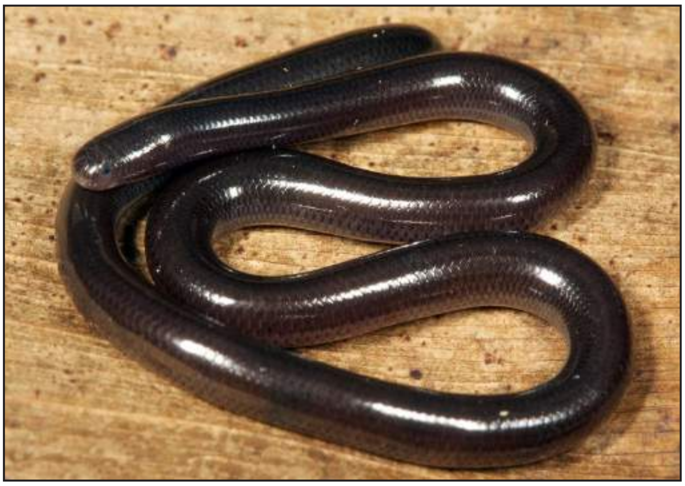

E

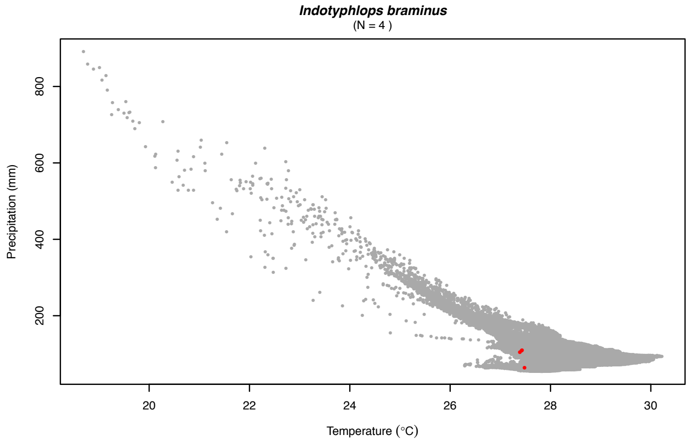

F

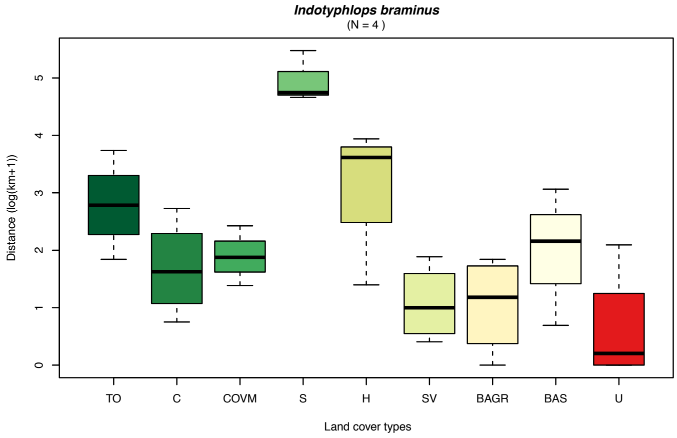

S57: Species information

Snakes
Viperidae

Cerastes gasperettii gasperettii
Leviton & Anderson, 1967

A

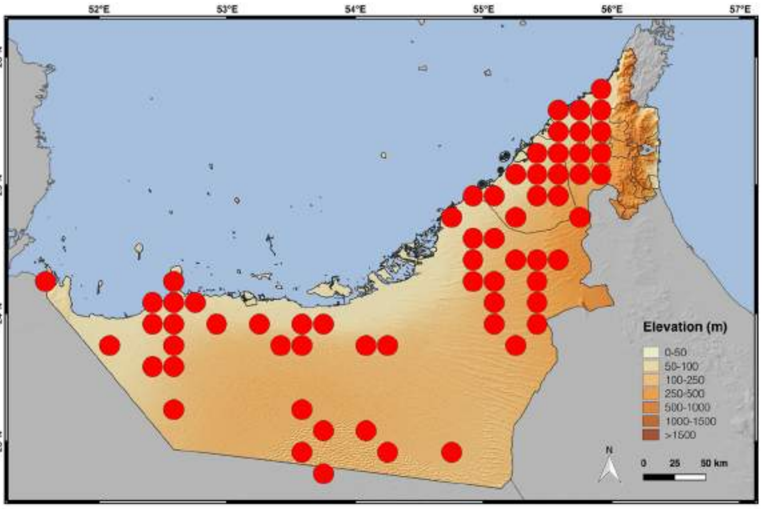

B

Useful information

IUCN Category	LC/LC
Venomous	YES
Endemic	NO
Origin	Native

D

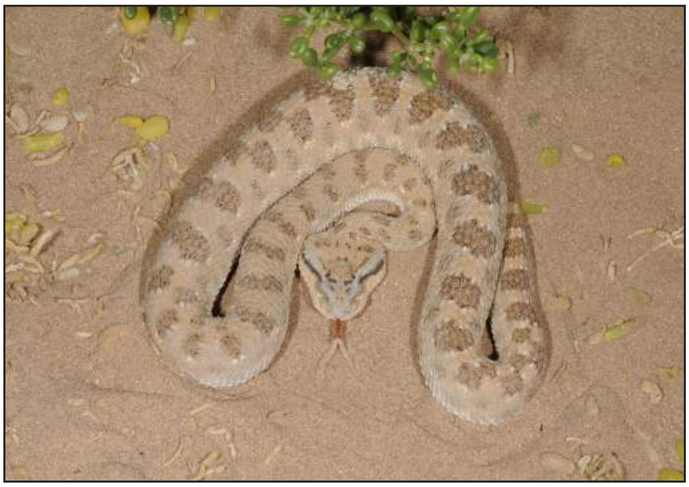

C

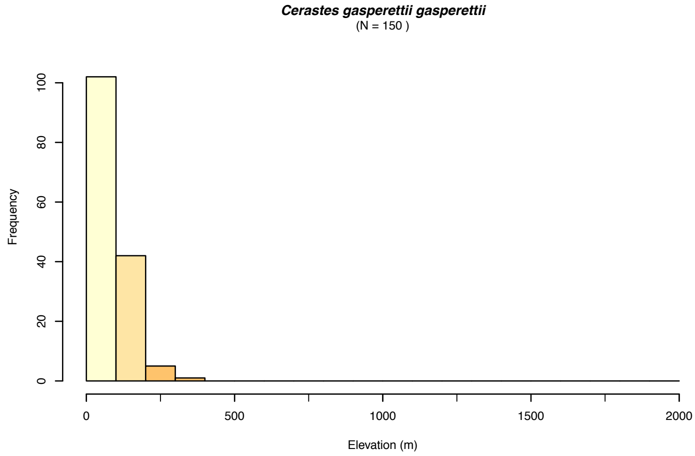

F

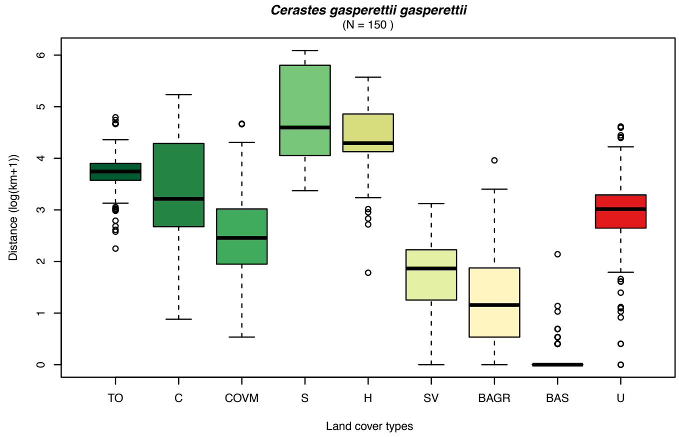

E

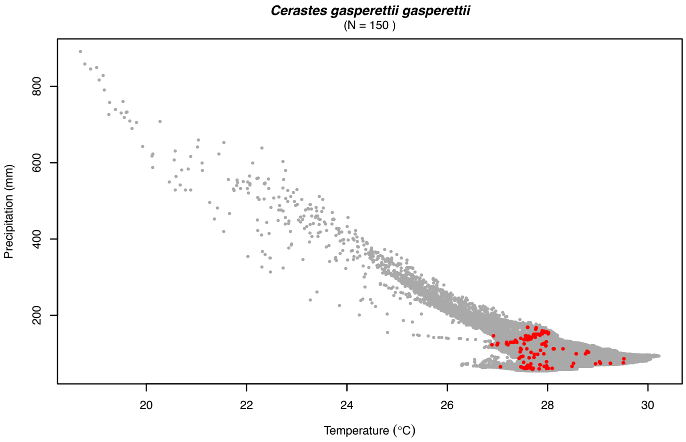

Echis carinatus sochureki Stemmler, 1969

S58: Species information

Snakes

Viperidae

A

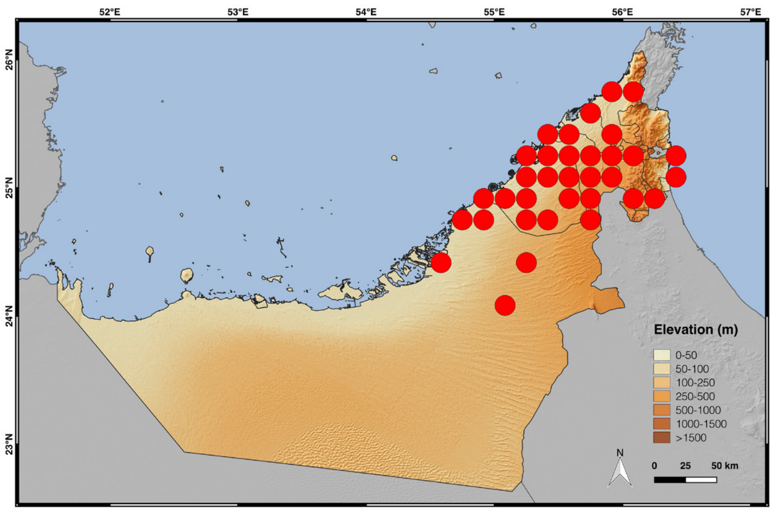

B

Useful information	
IUCN Category	LC/LC
Venomous	YES
Endemic	NO
Origin	Native

C

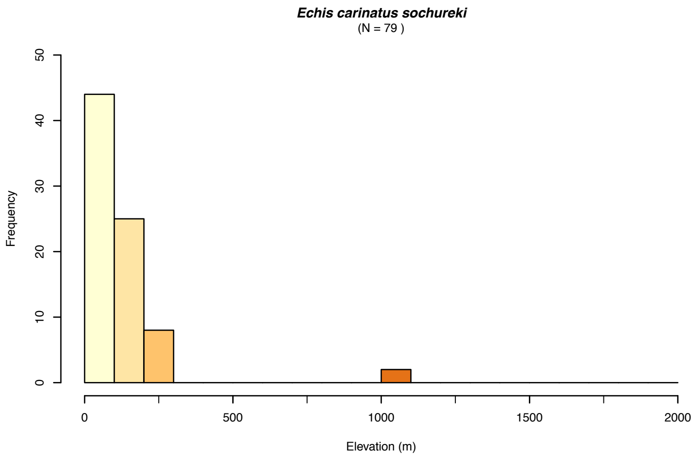

D

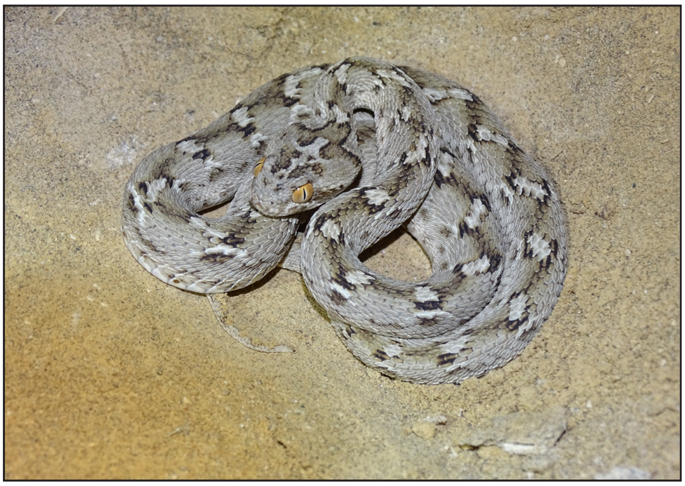

E

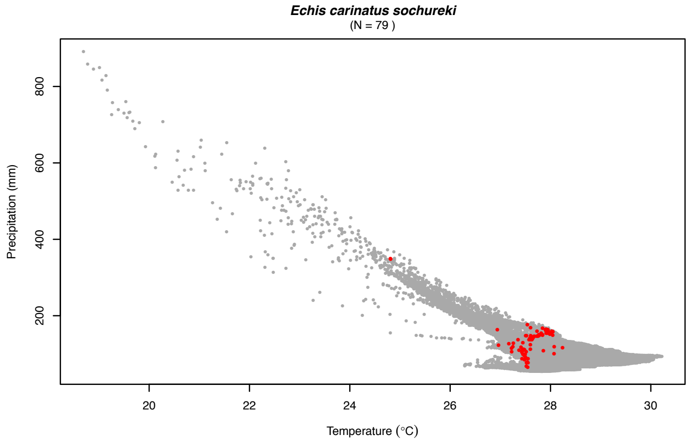

F

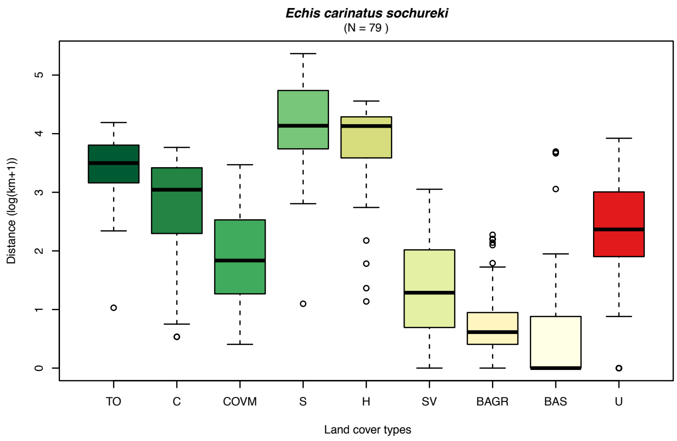

S59: Species information

Snakes

Viperidae

Echis omanensis

Babocsay, 2004

A

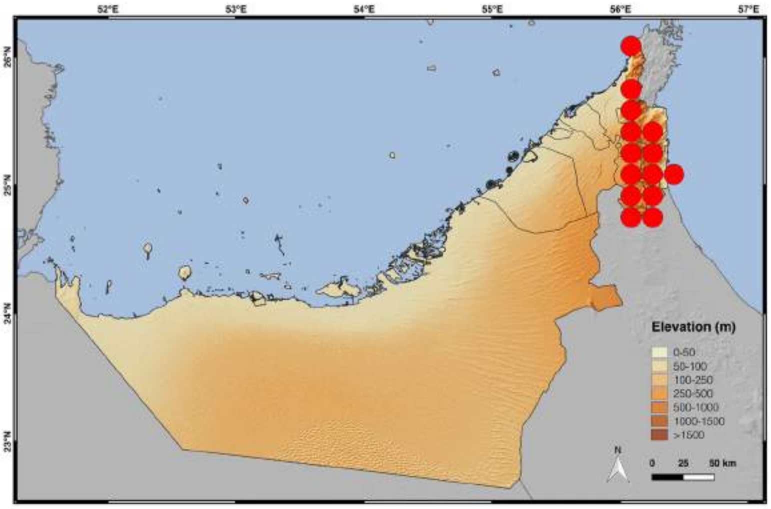

B

Useful information	
IUCN Category	LC/LC
Venomous	YES
Endemic	NO
Origin	Native

D

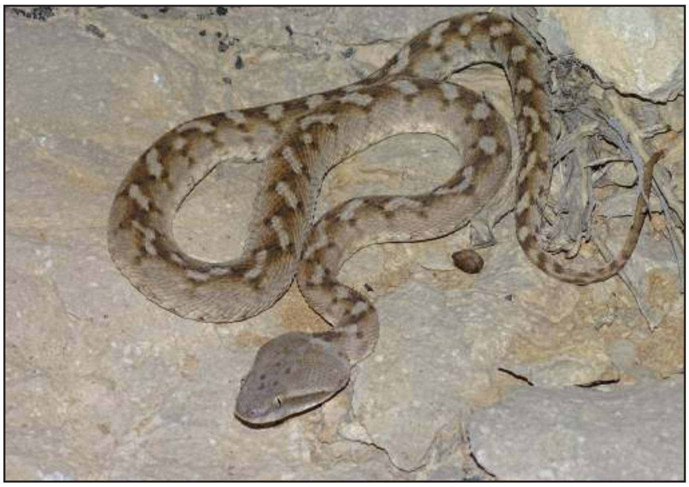

C

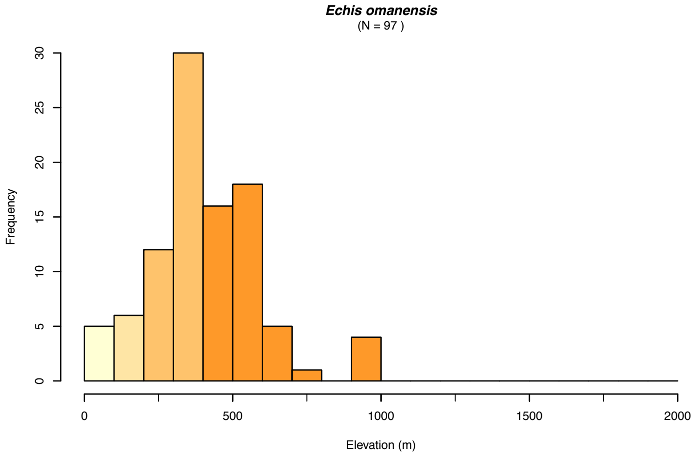

F

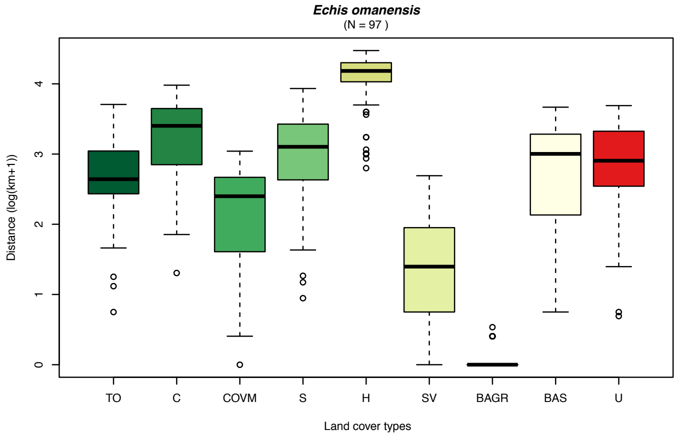

E

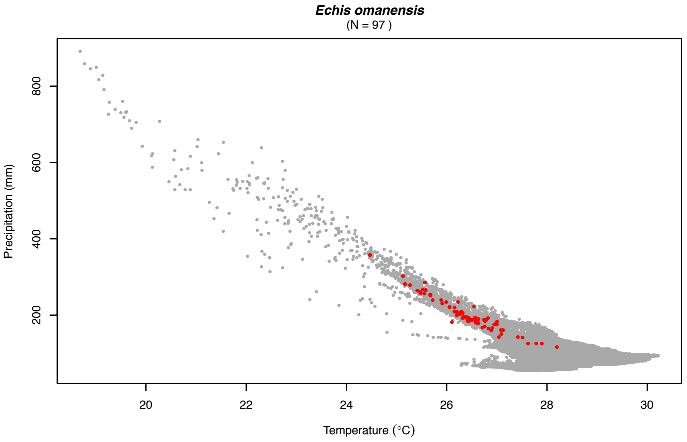

Lizards
Agamids
Geckos
Lacertids
Skinks
Amphisbaenids
Varanids
Snakes
Boidae
Colubridae
Lamprophiidae
Leptotyphlopidae
Typhlopidae
Viperidae

Pseudocerastes persicus
Duméril, Bibron & Duméril, 1854

S60: Species information

Snakes

Viperidae

A

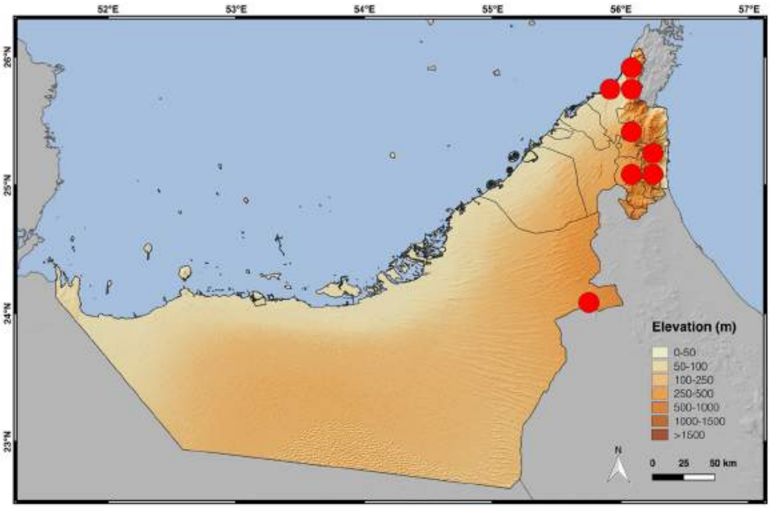

B

Useful information	
IUCN Category	LC/LC
Venomous	YES
Endemic	NO
Origin	Native

C

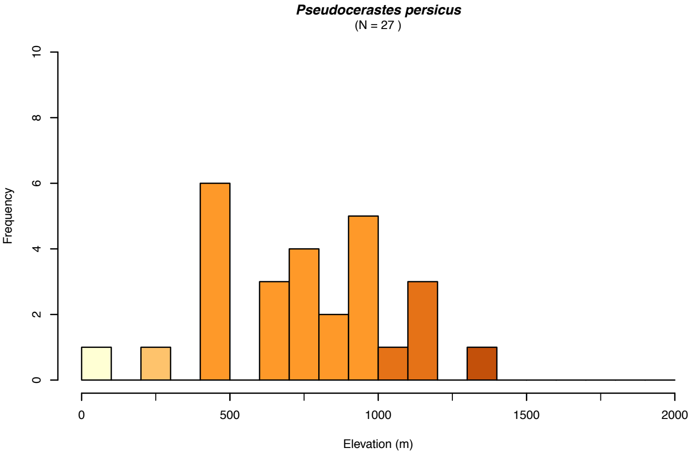

D

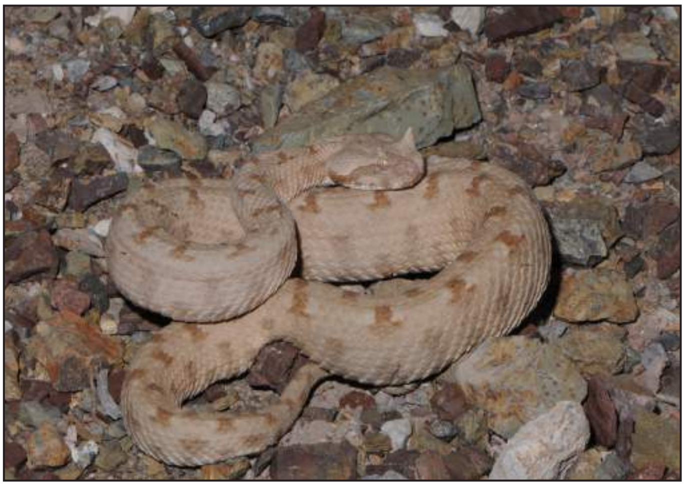

E

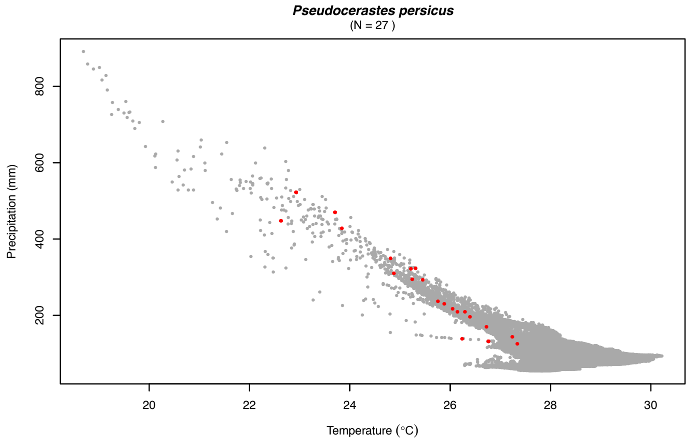

F

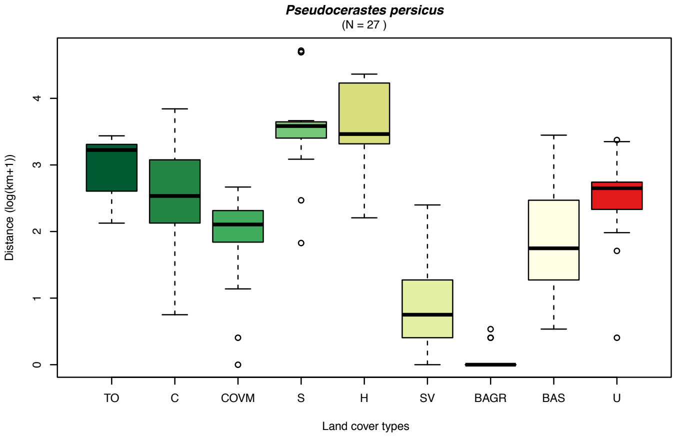

S61-S62: Species distribution models

Plates contain independent maps for each species showing the species' potential distribution inferred with a species distribution model (SDM) and using four ecological and environmental variables (see materials and methods 2.8). For each species there are two maps: **A**) a map of the modeled species distribution at a resolution of 10 arc-minutes (~18 km) and **B**) a map of the modeling at a 1km² resolution including the species' point records in black.

Eight out of the sixty species of reptiles did not have the minimum of 5 records to infer their SDM with the modeling approach. In these cases, the species' potential distribution was calculated with the minimum convex polygon (MCP) between all presence point data of each species. Maps with the MCP of each of the eight species and all their occurrence point records (red dots) were created (**C**).

Hemidactylus robustus

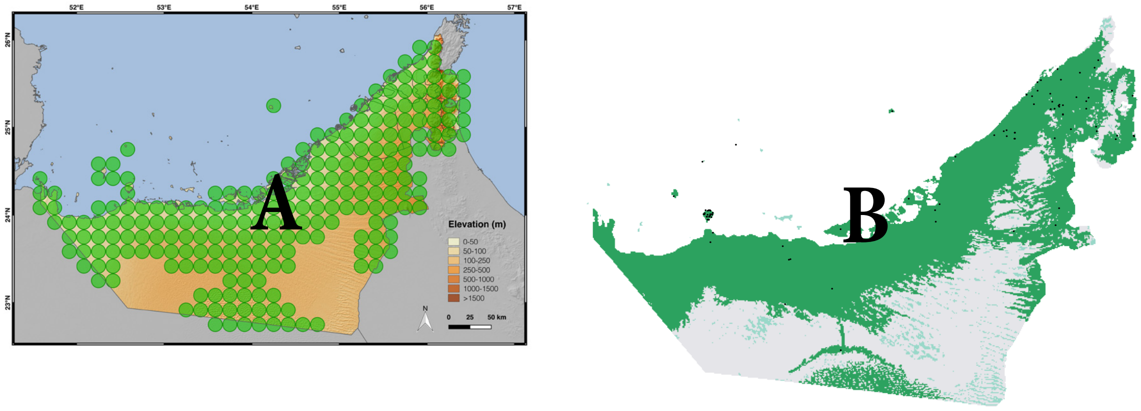

Hemidactylus persicus

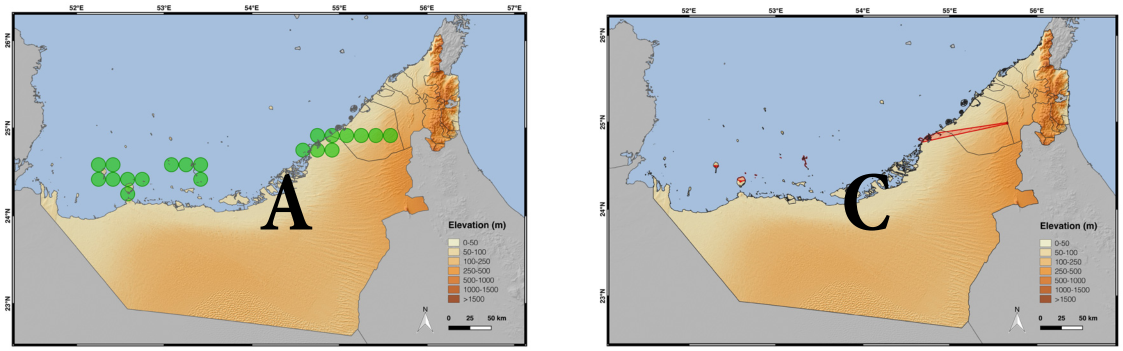

Species distribution models

Phrynocephalus arabicus

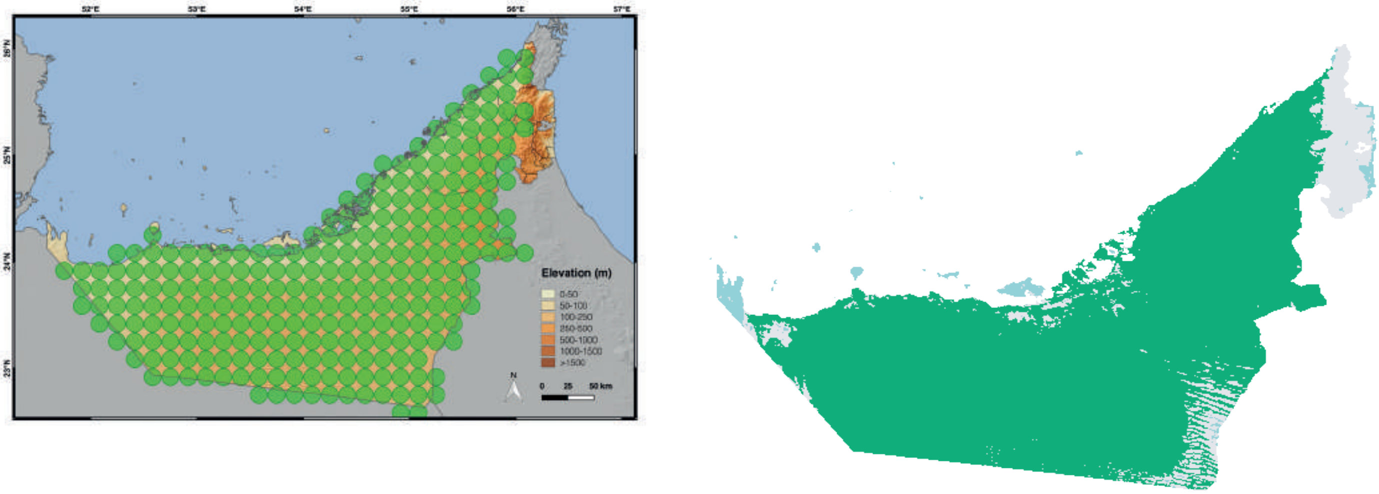

Phrynocephalus maculatus

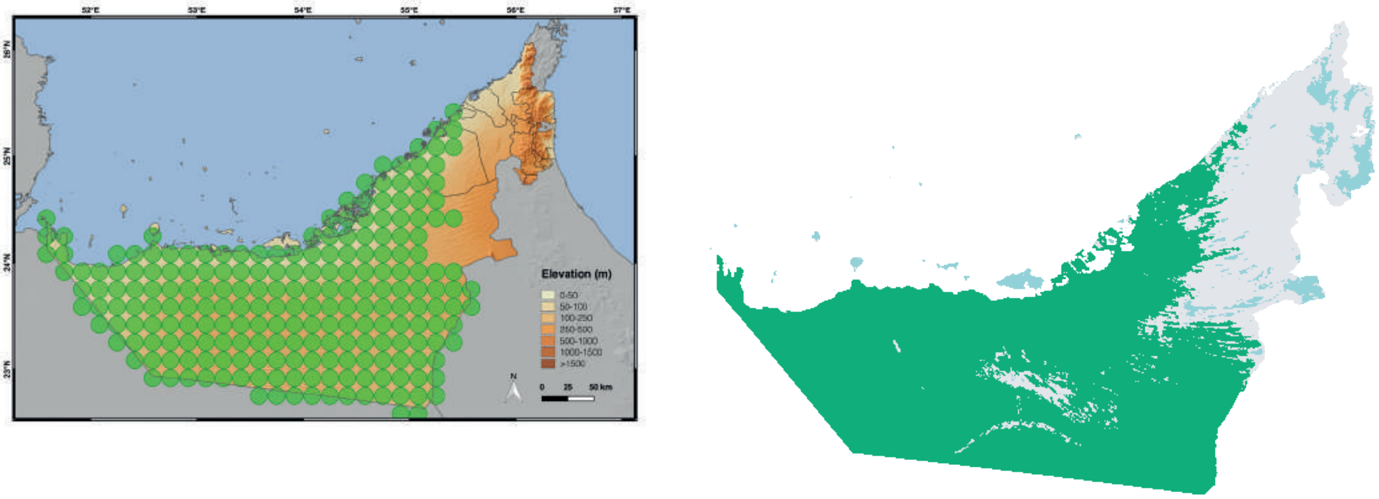

Pseudotrapelus jensvindumi

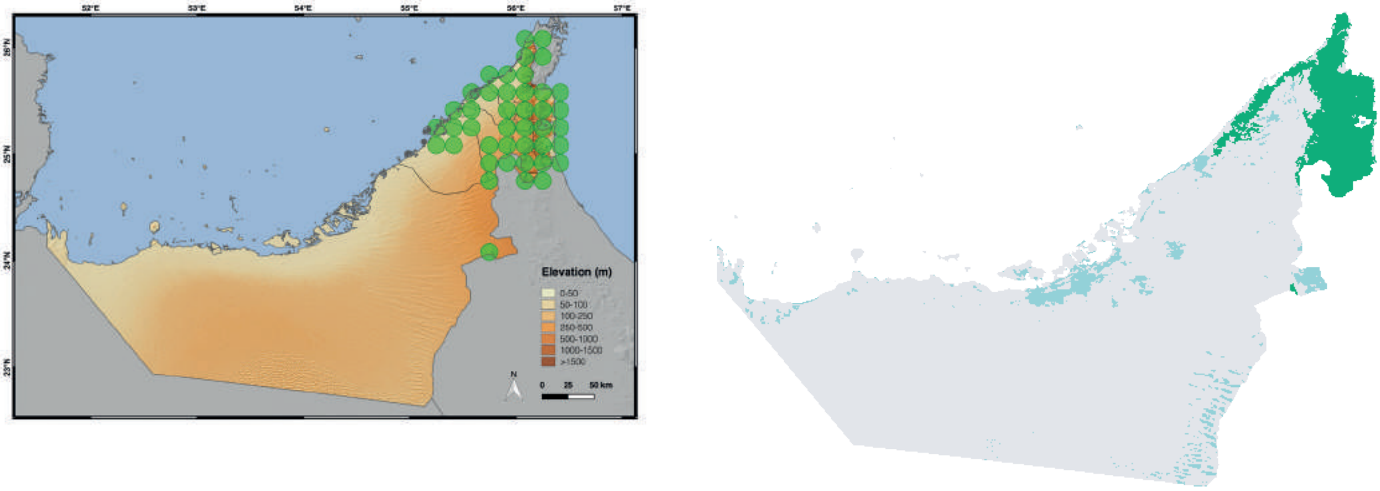

Species distribution models

Trapelus flavimaculatus

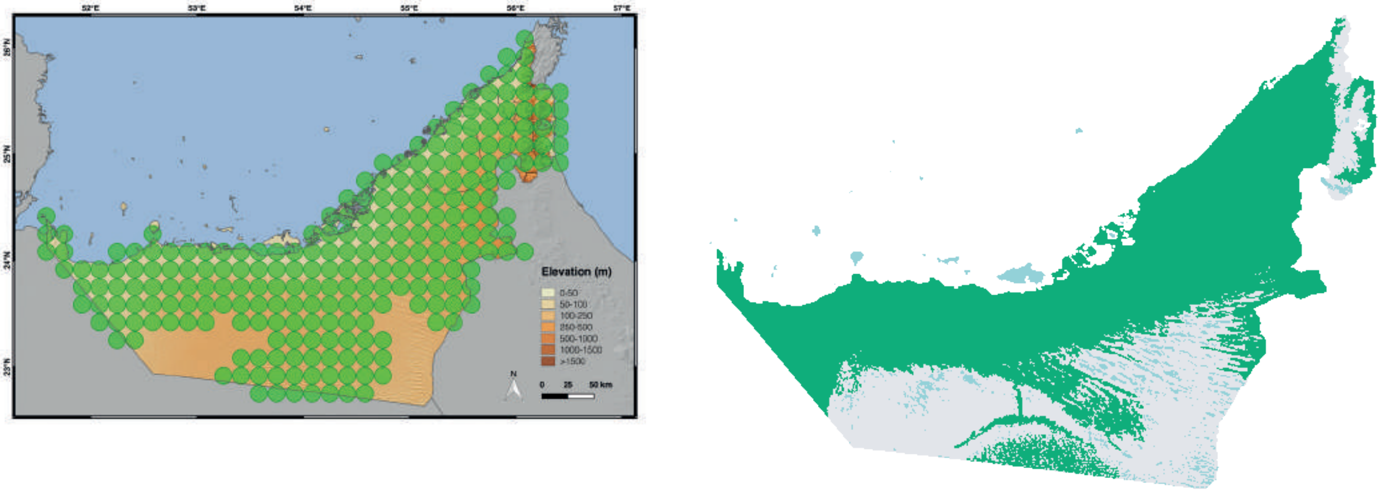

Uromastyx aegyptia leptieni

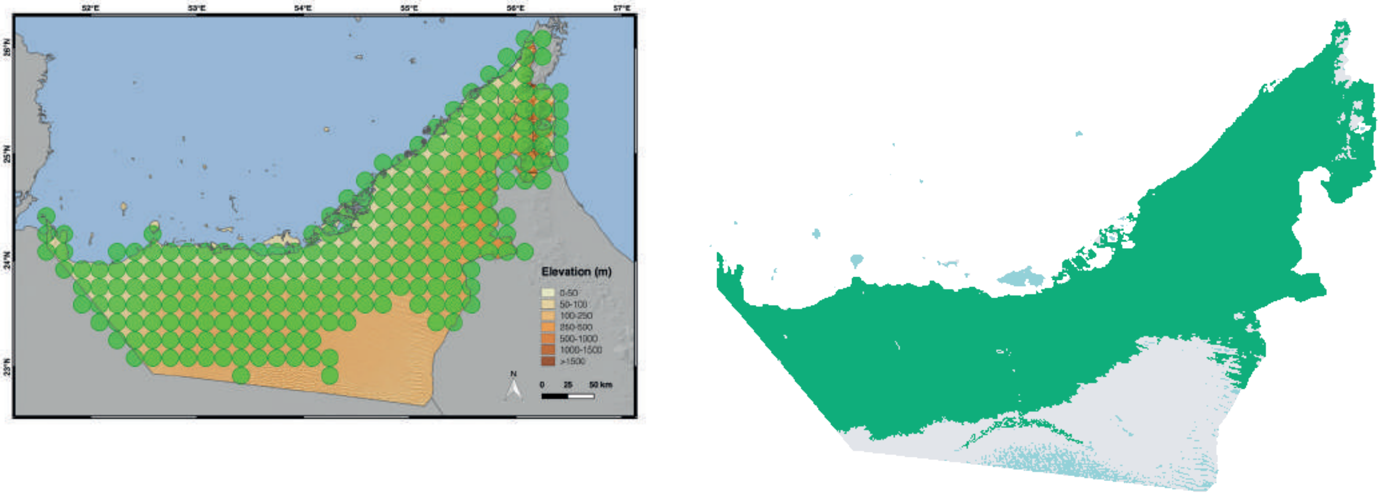

Uromastyx aegyptia microlepis

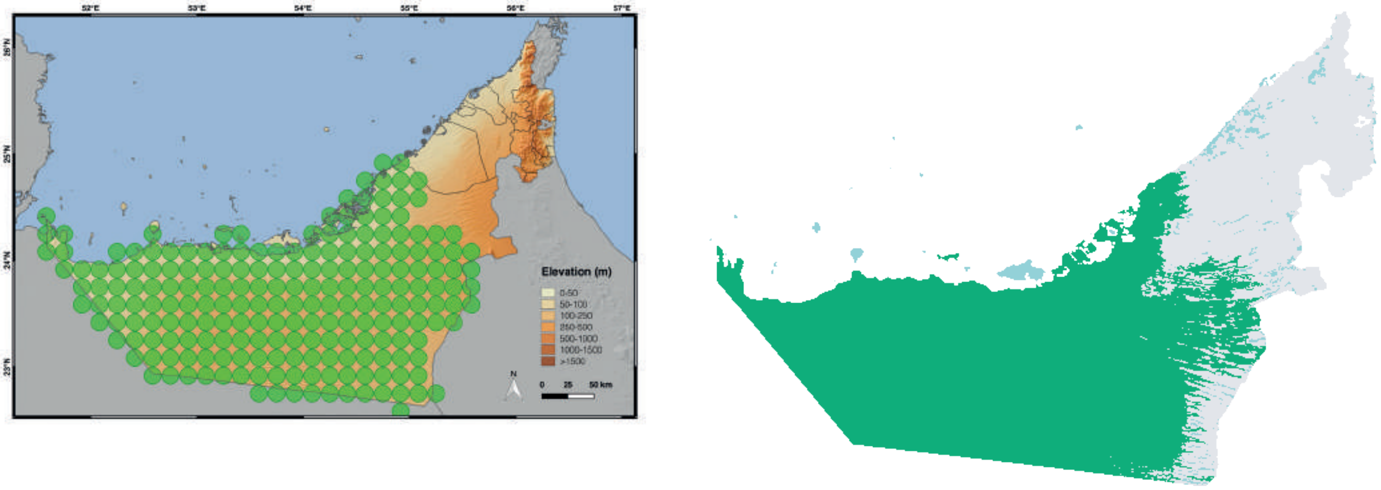

Species distribution models

Bunopus tuberculatus

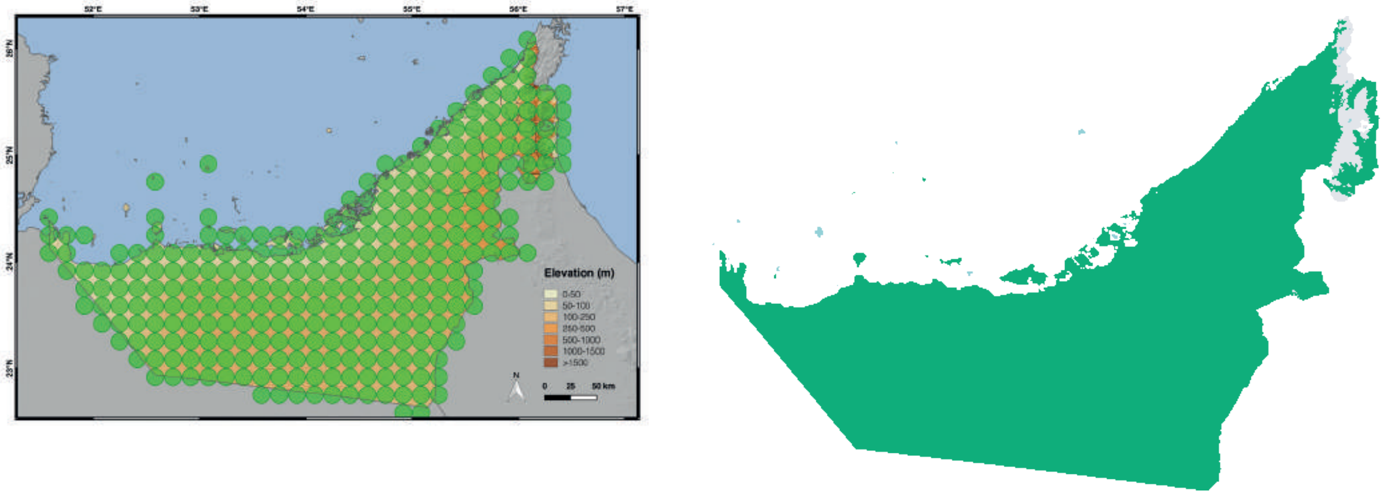

Cyrtopodion scabrum

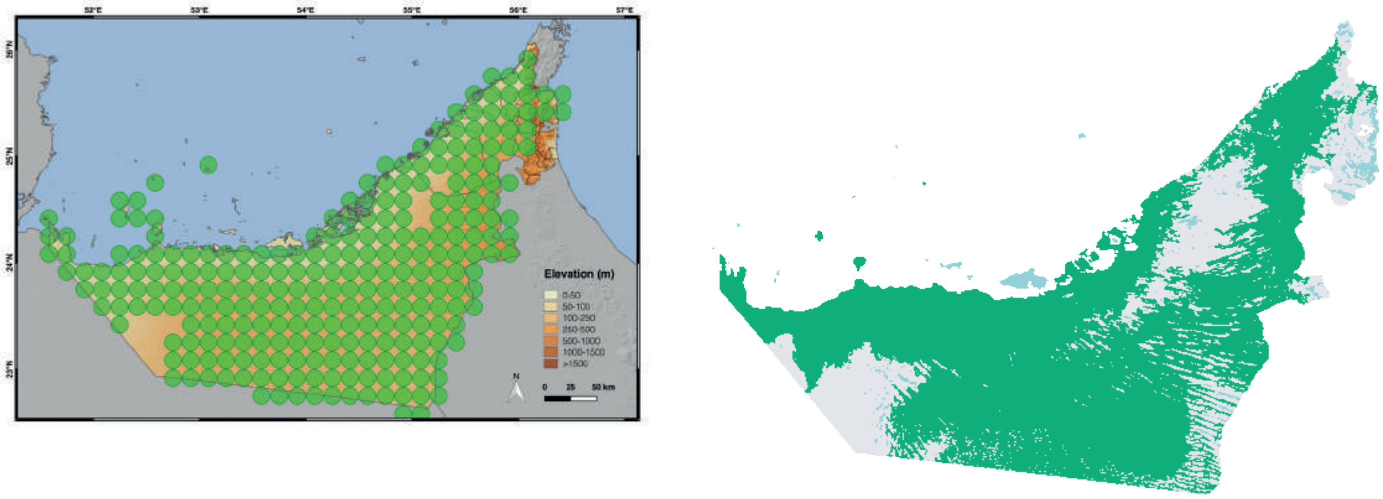

Hemidactylus flaviviridis

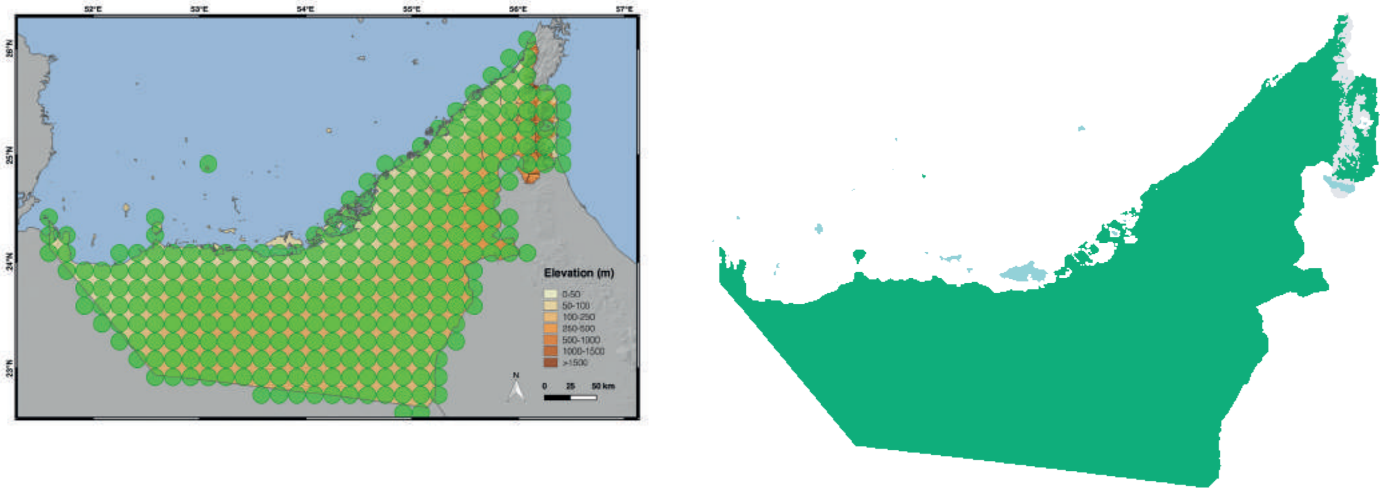

Species distribution models

Hemidactylus persicus

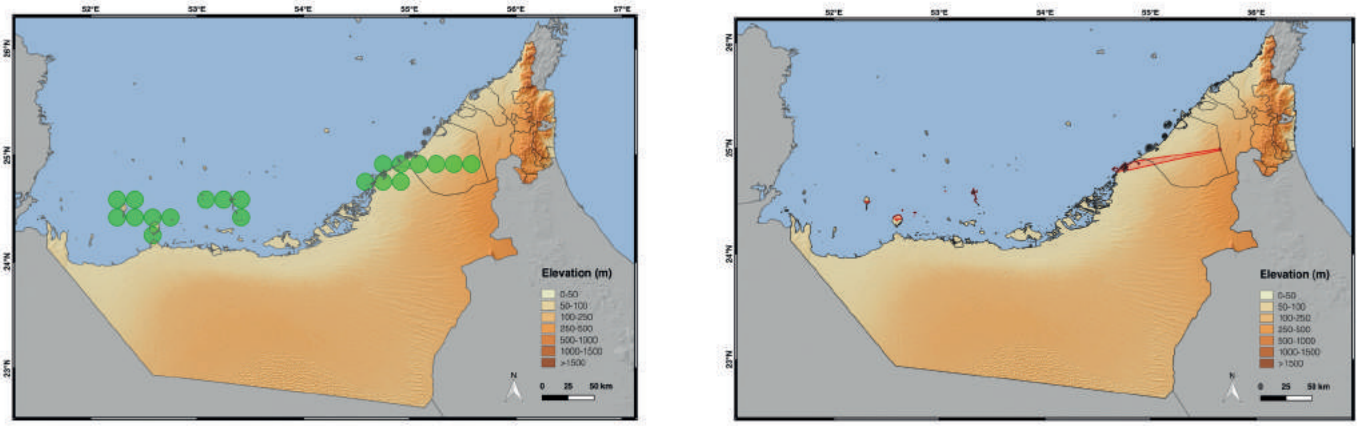

Hemidactylus robustus

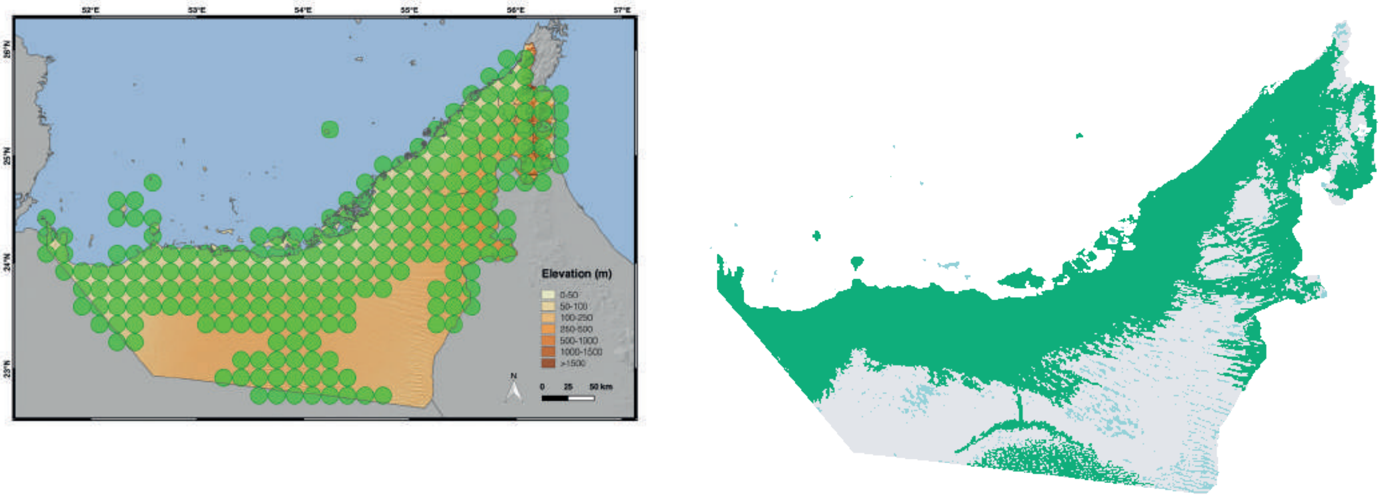

Pseudoceramodactylus khobarensis

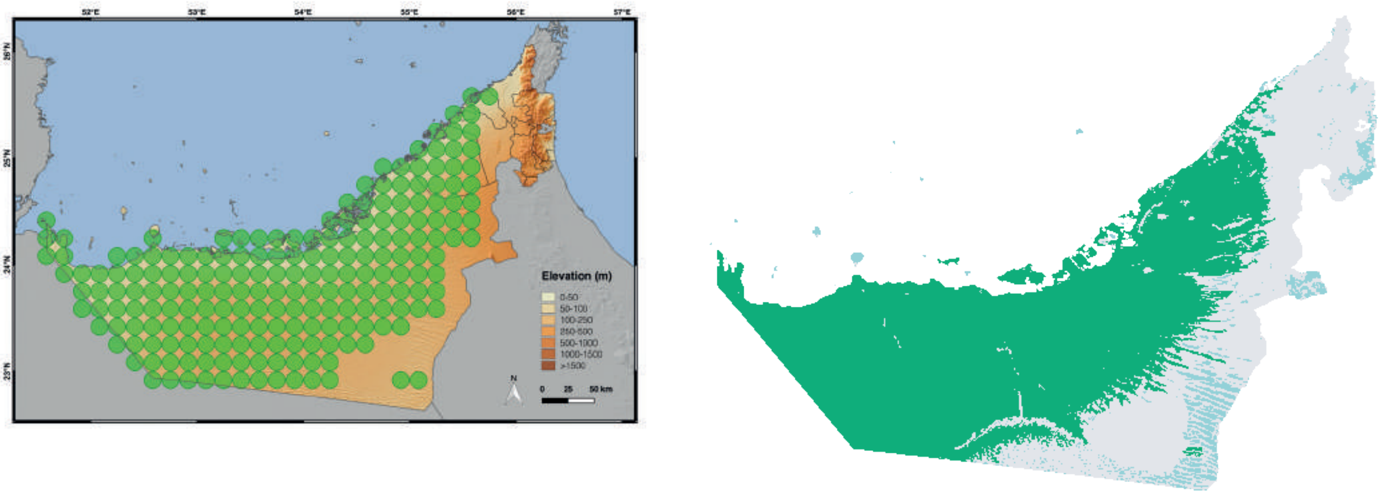

Species distribution models

Stenodactylus arabicus

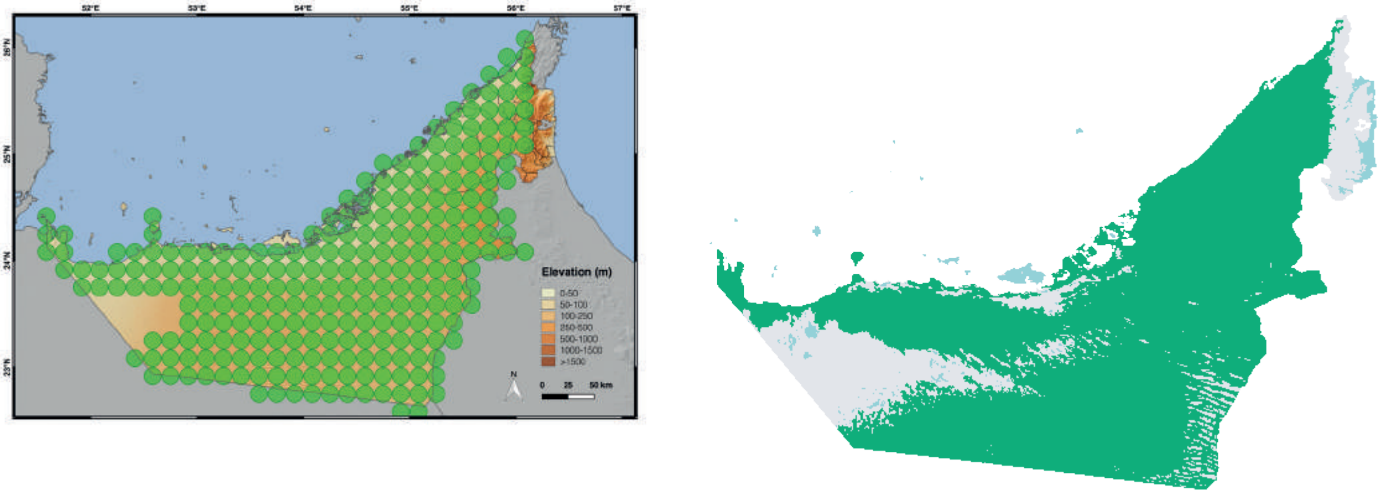

Stenodactylus doriae

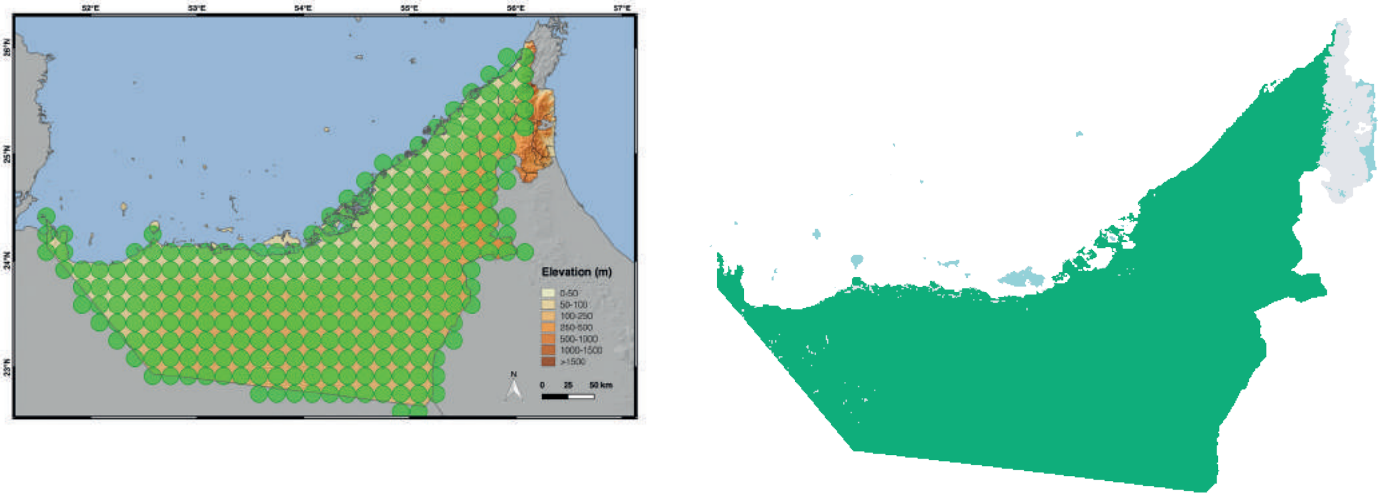

Stenodactylus leptocosymbotes

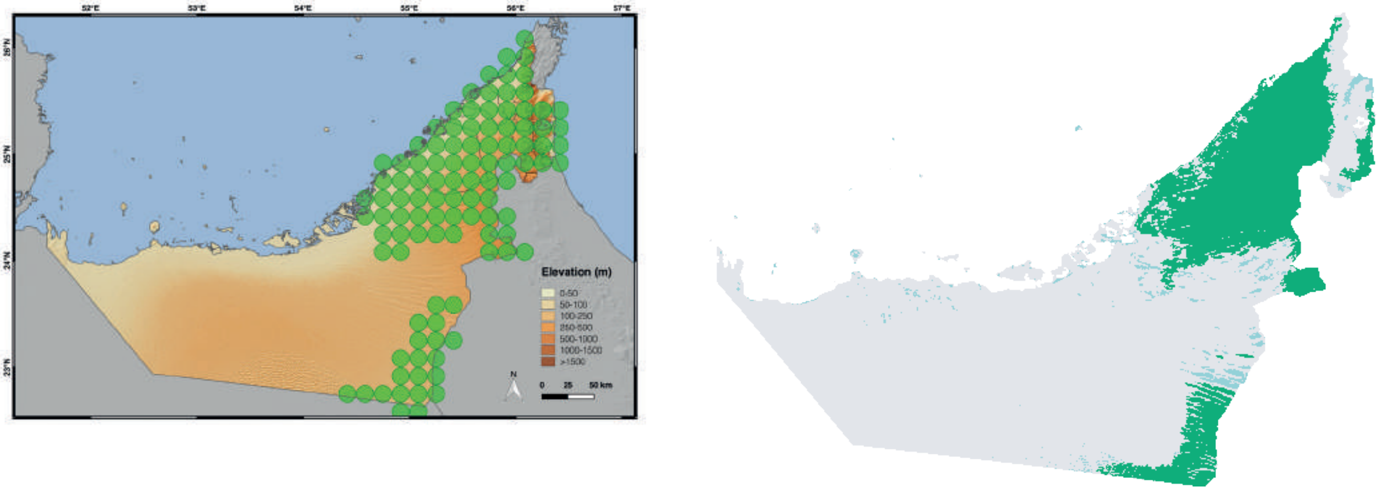

Species distribution models

Stenodactylus slevini

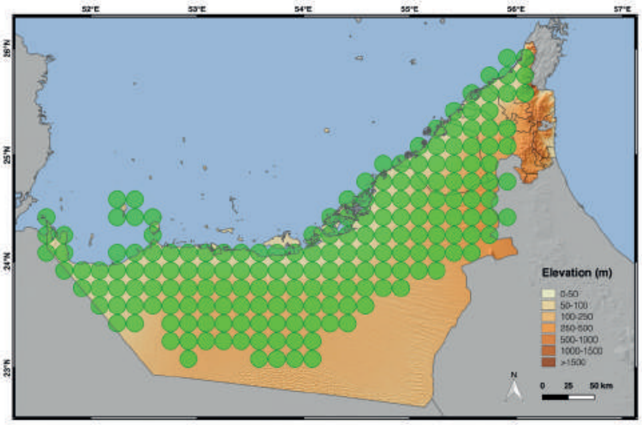

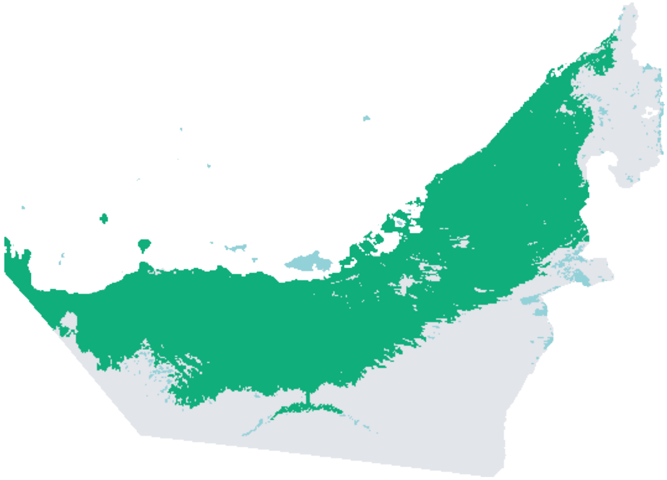

Trachydactylus hajarensis

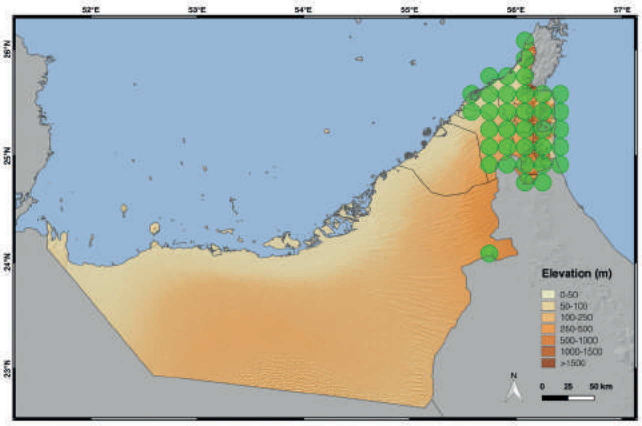

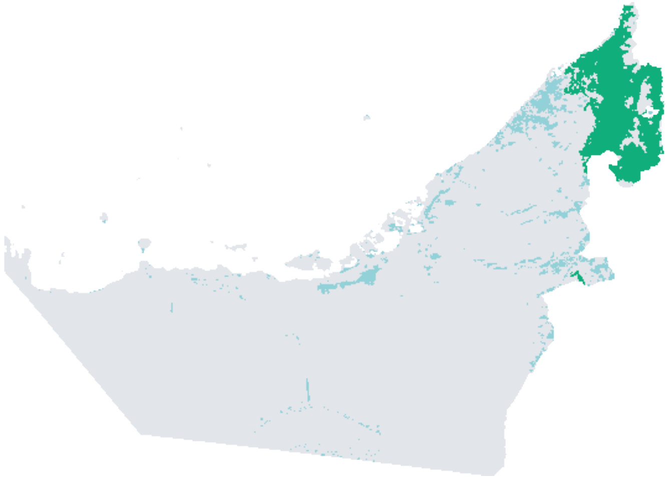

Asaccus caudivolvulus

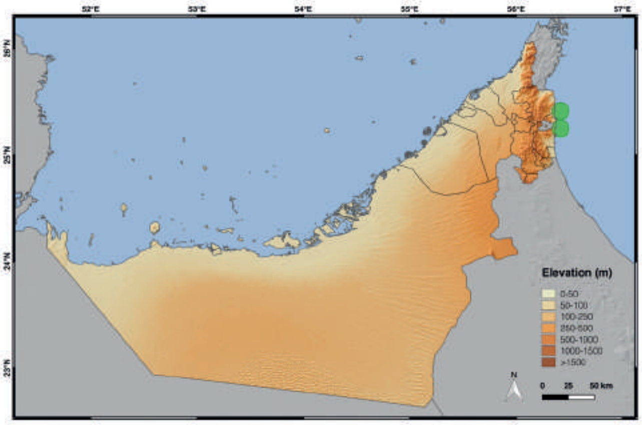

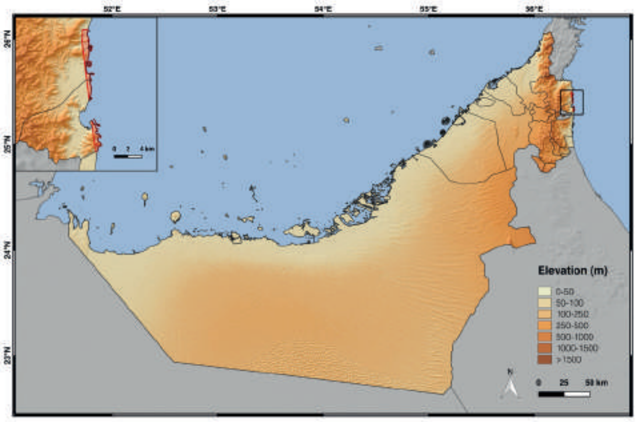

Species distribution models

Asaccus gallagheri

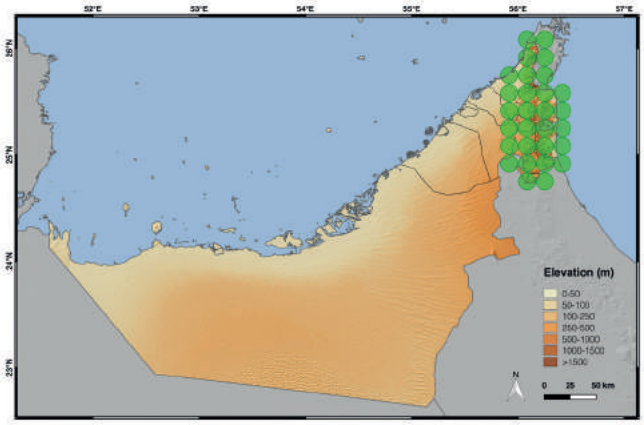

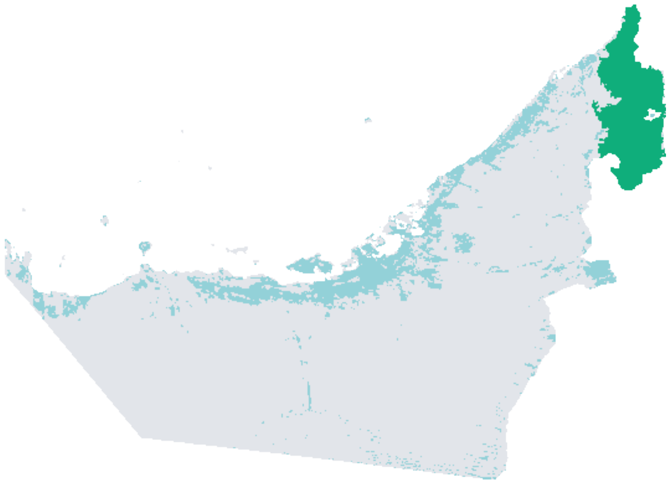

Asaccus gardneri

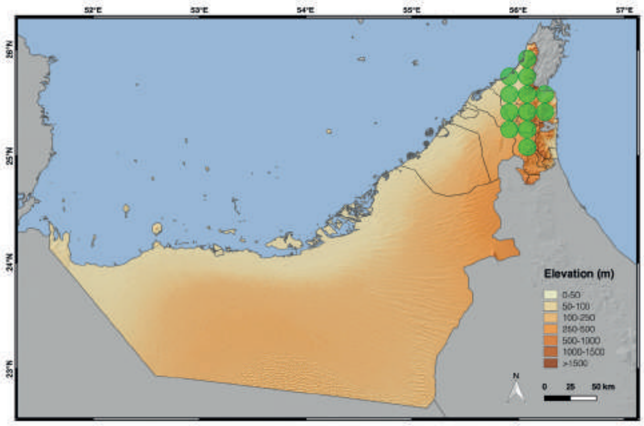

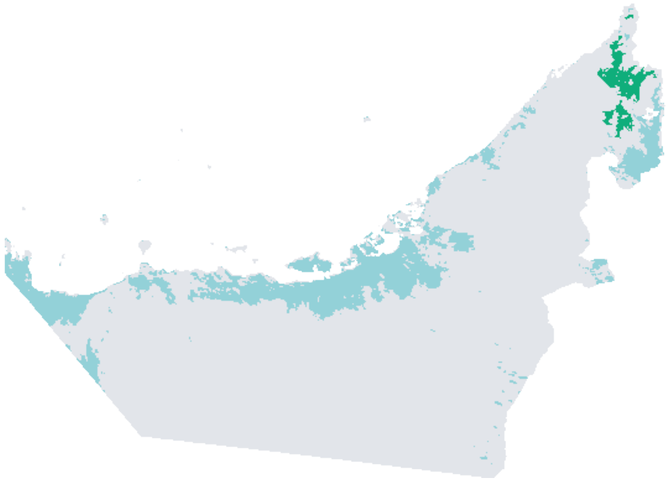

Asaccus margaritae

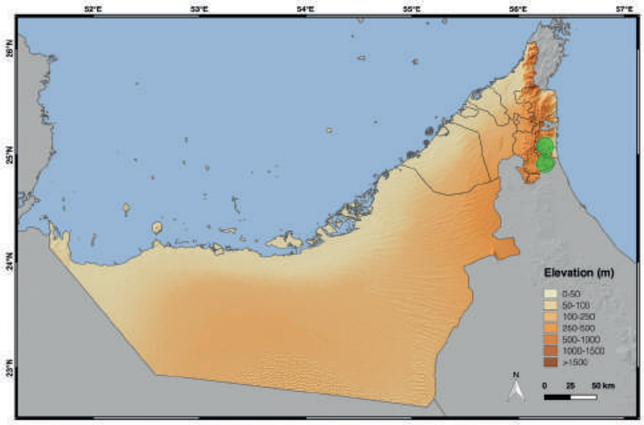

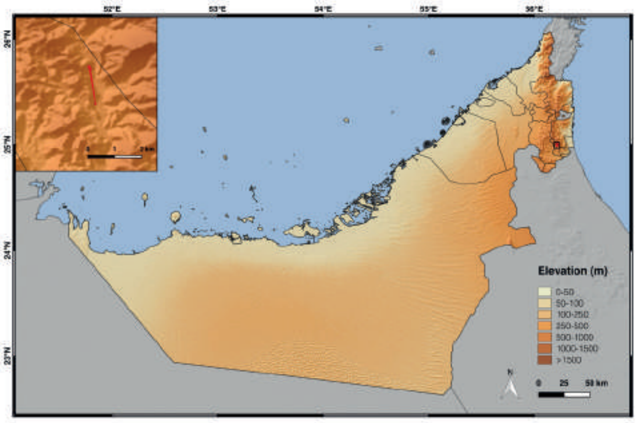

Species distribution models

Ptyodactylus orlovi

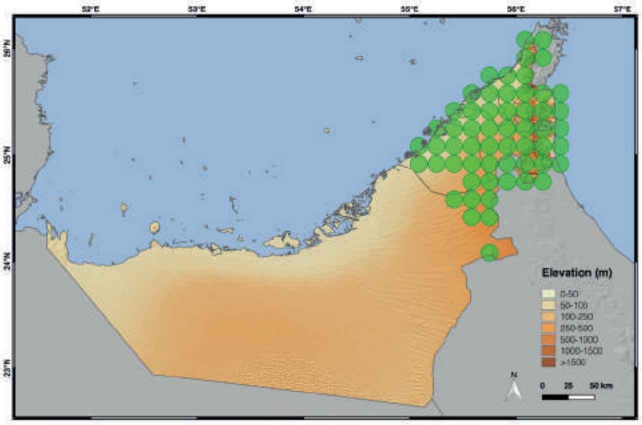

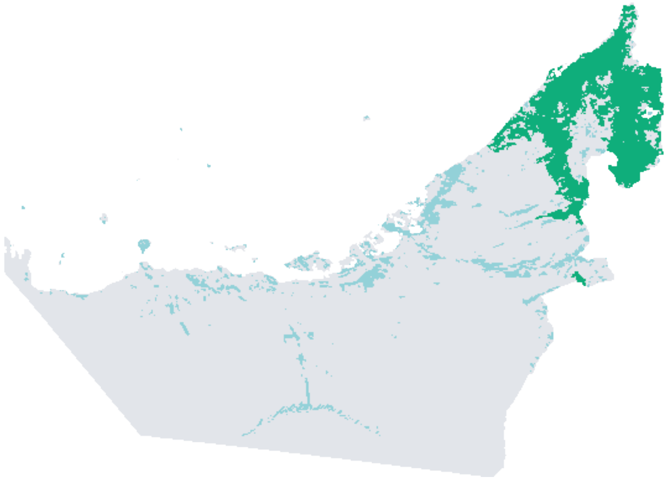

Ptyodactylus ruusaljibalicus

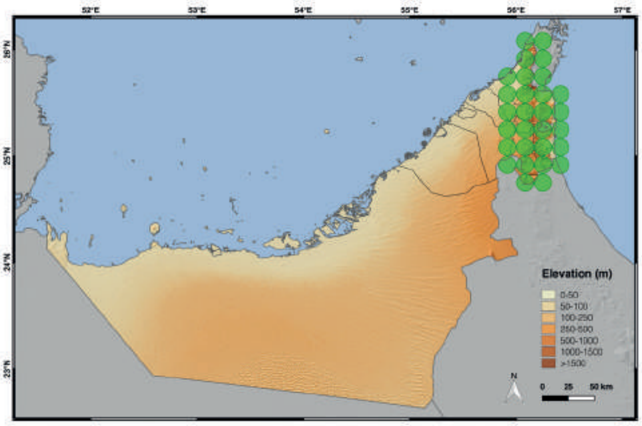

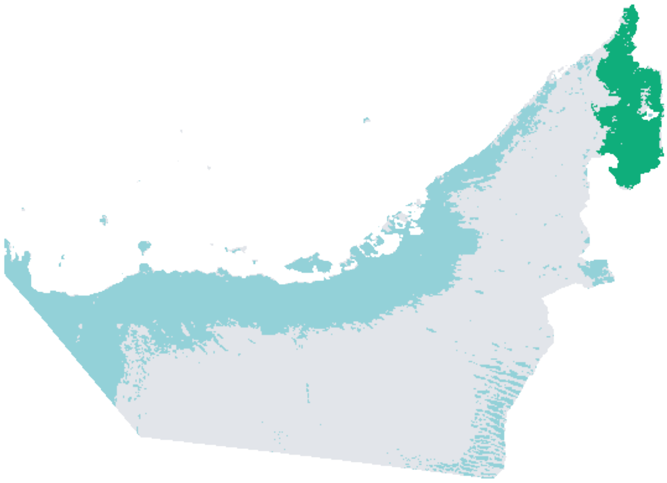

Pristurus carteri

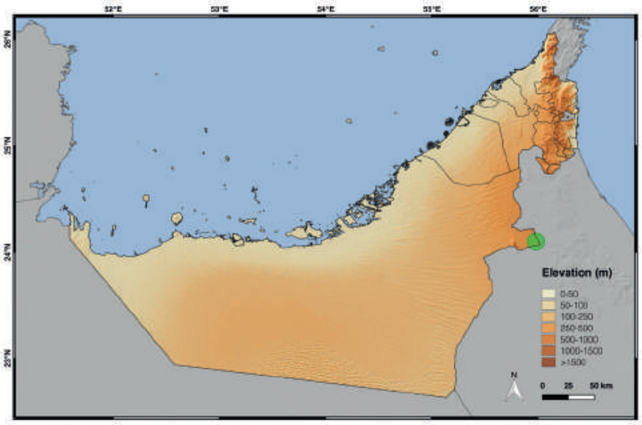

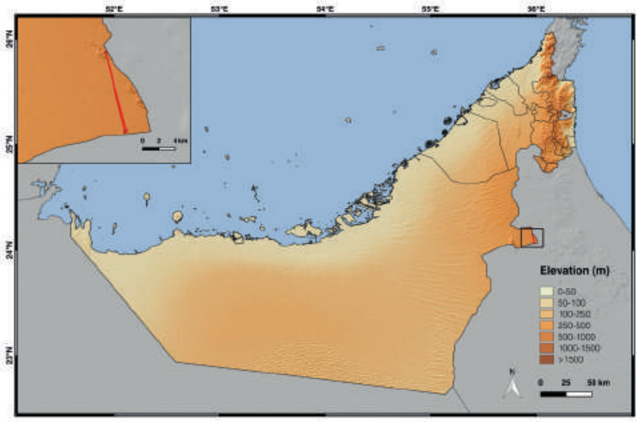

S69: Species distribution models

Pristurus celerrimus

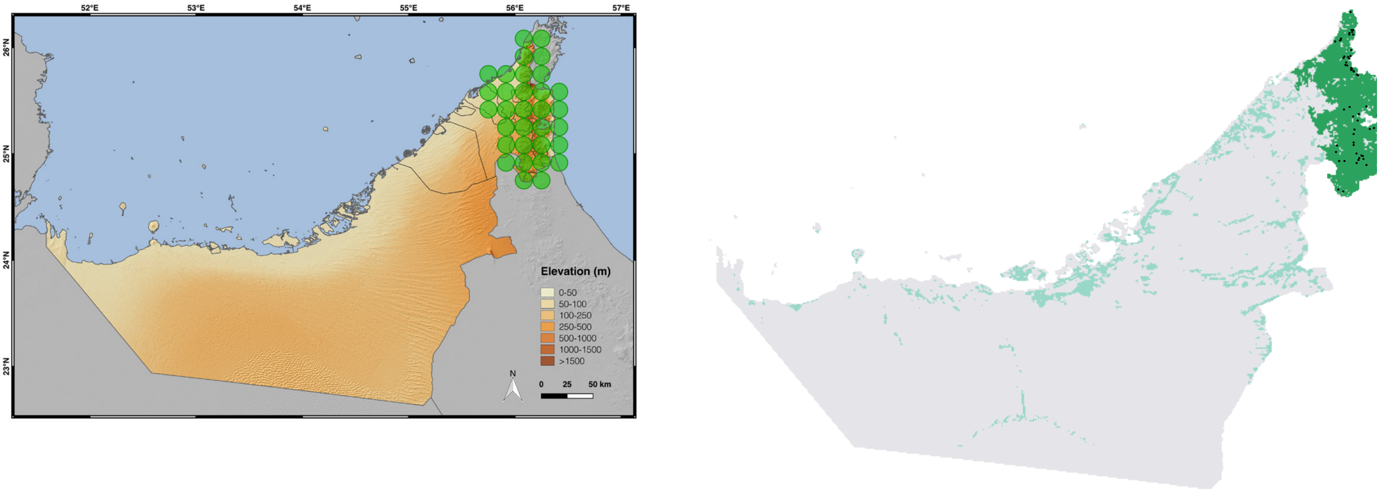

Pristurus minimus

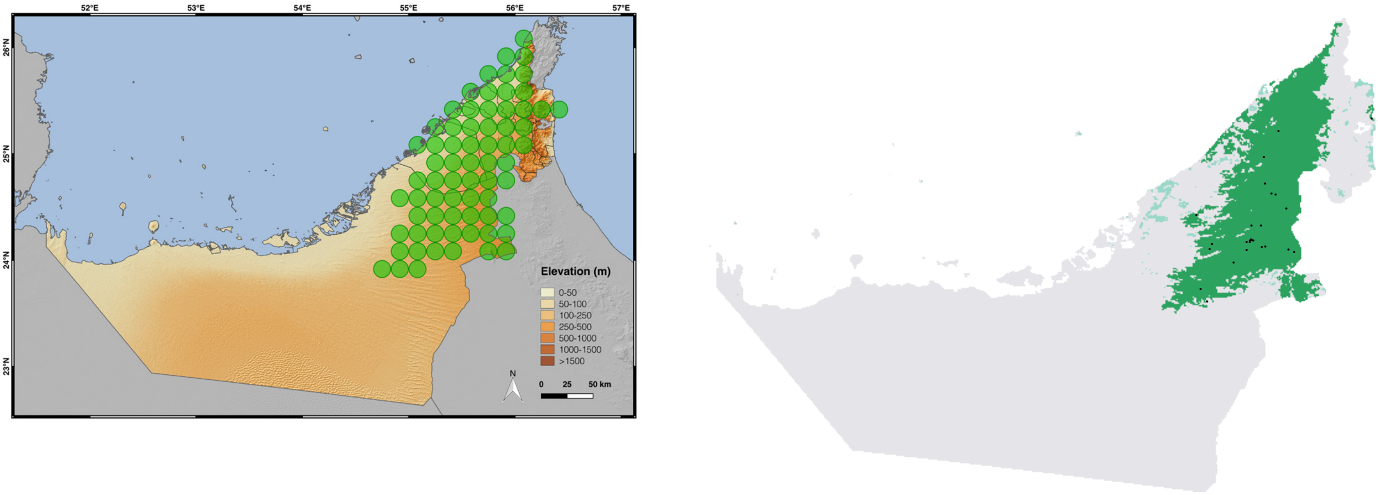

Pristurus rupestris-sp. 3

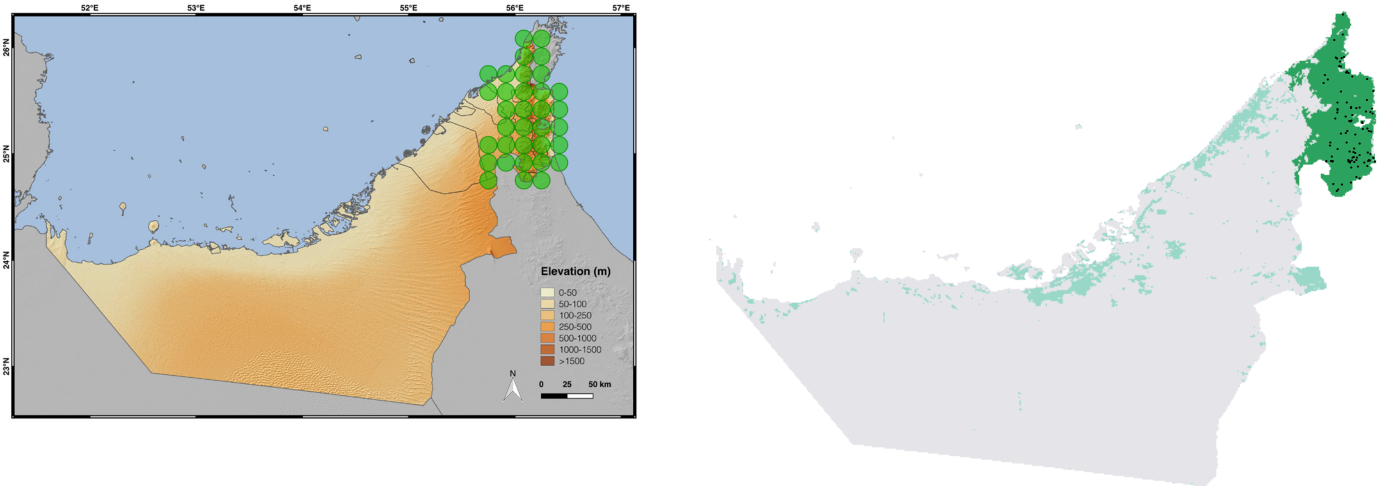

S70: Species distribution models

Teratoscincus keyserlingii

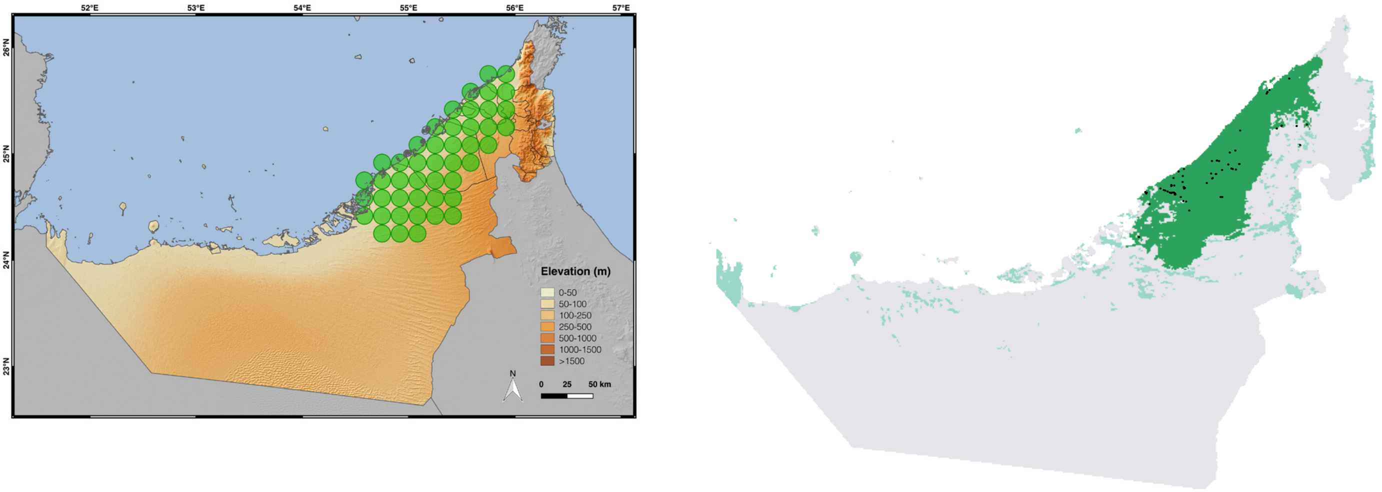

Acanthodactylus blanfordii

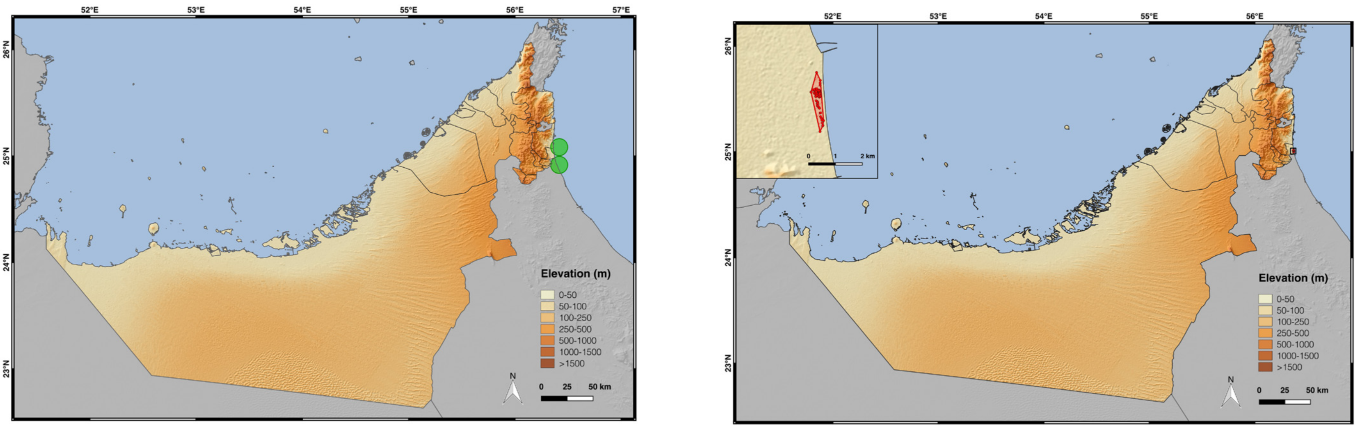

Acanthodactylus boskianus asper

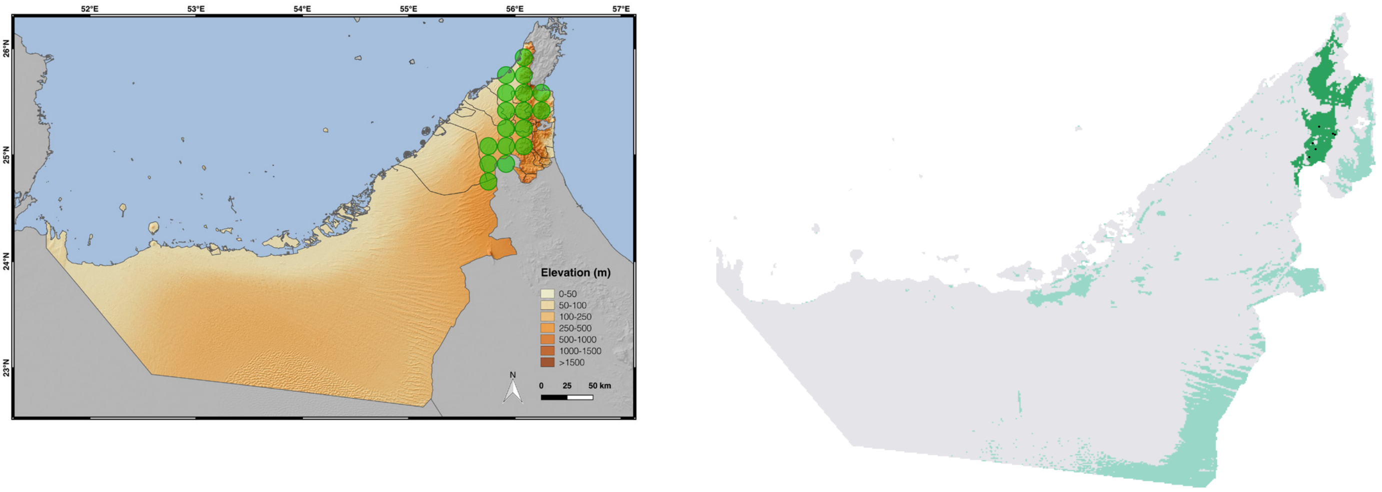

Species distribution models

Acanthodactylus gongrorhynchatus

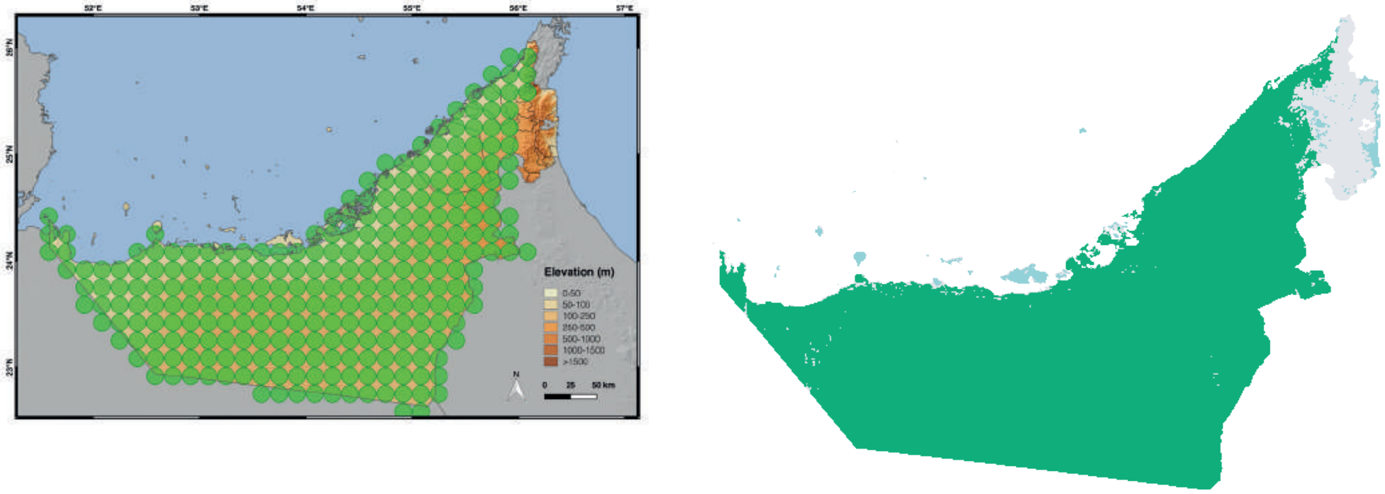

Acanthodactylus haasi

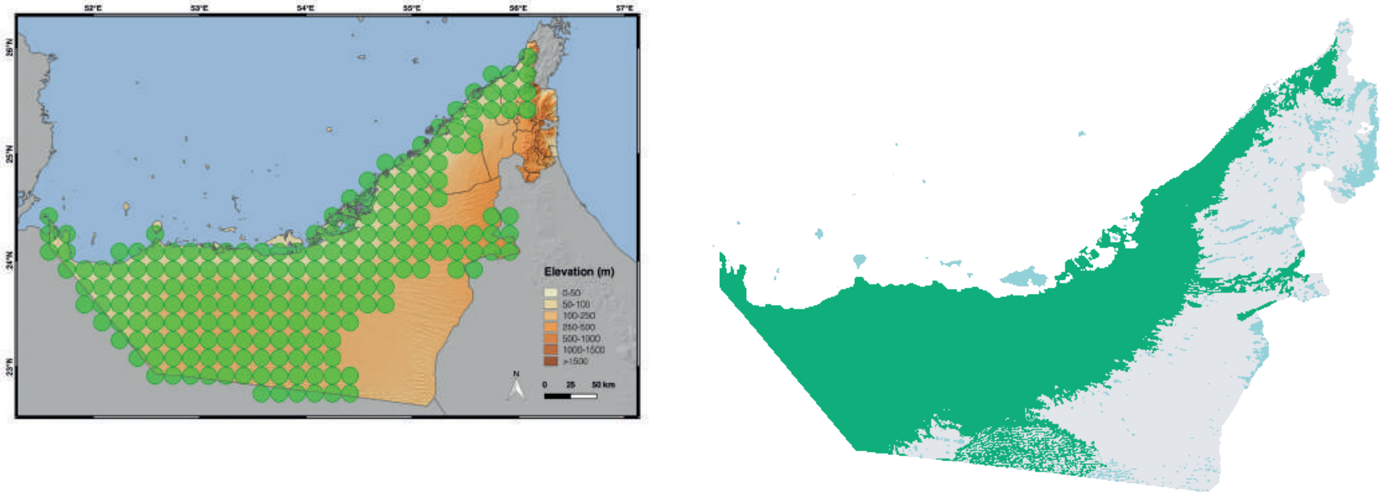

Acanthodactylus opheodorus

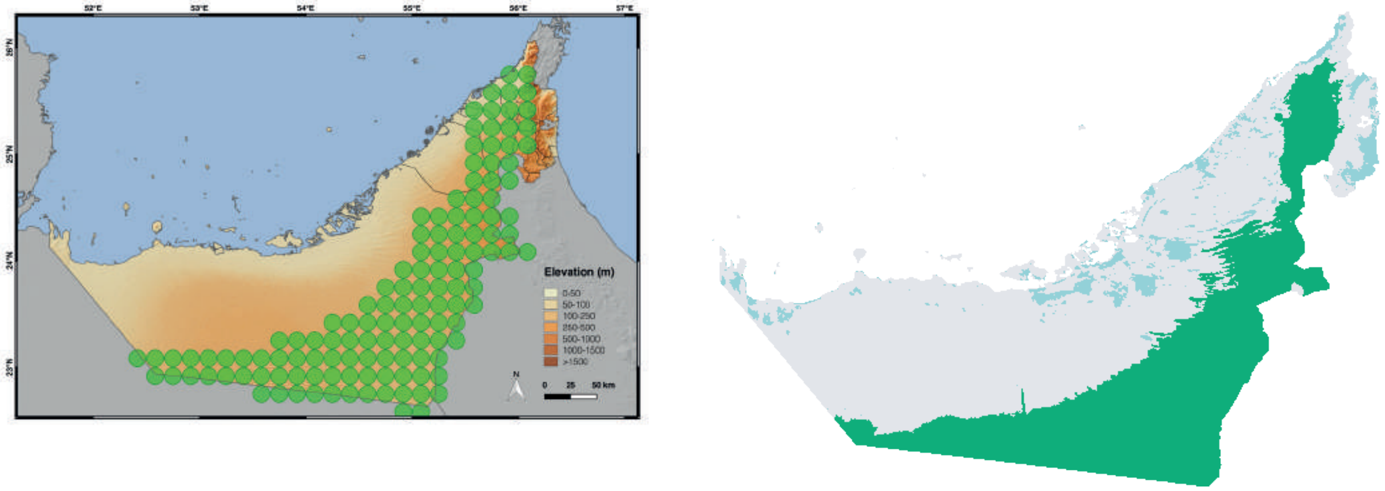

Species distribution models

Acanthodactylus schmidti

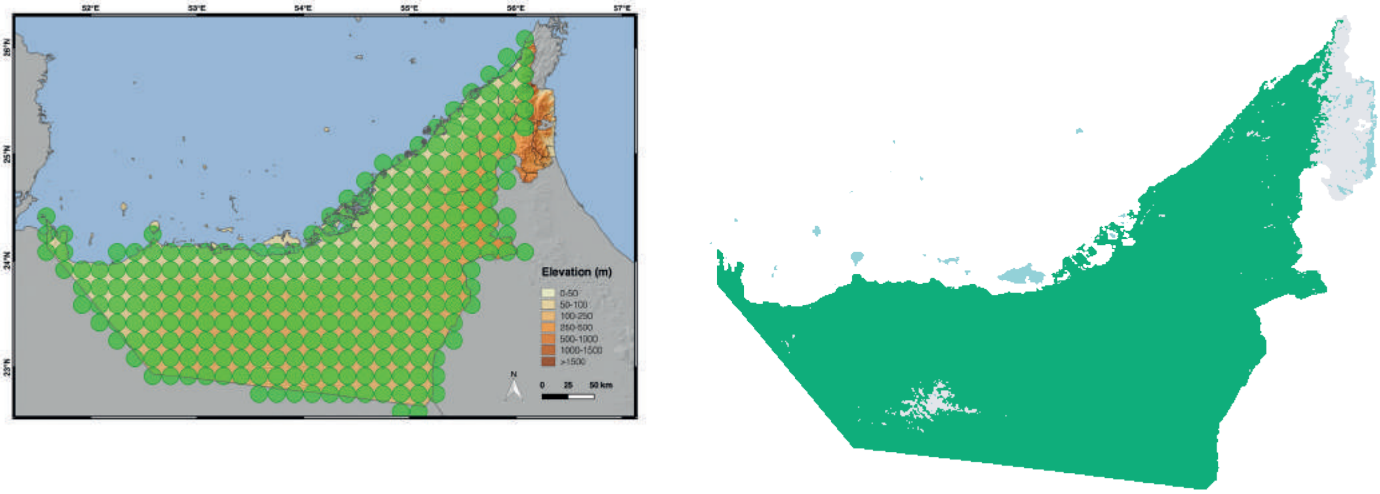

Mesalina adramitana

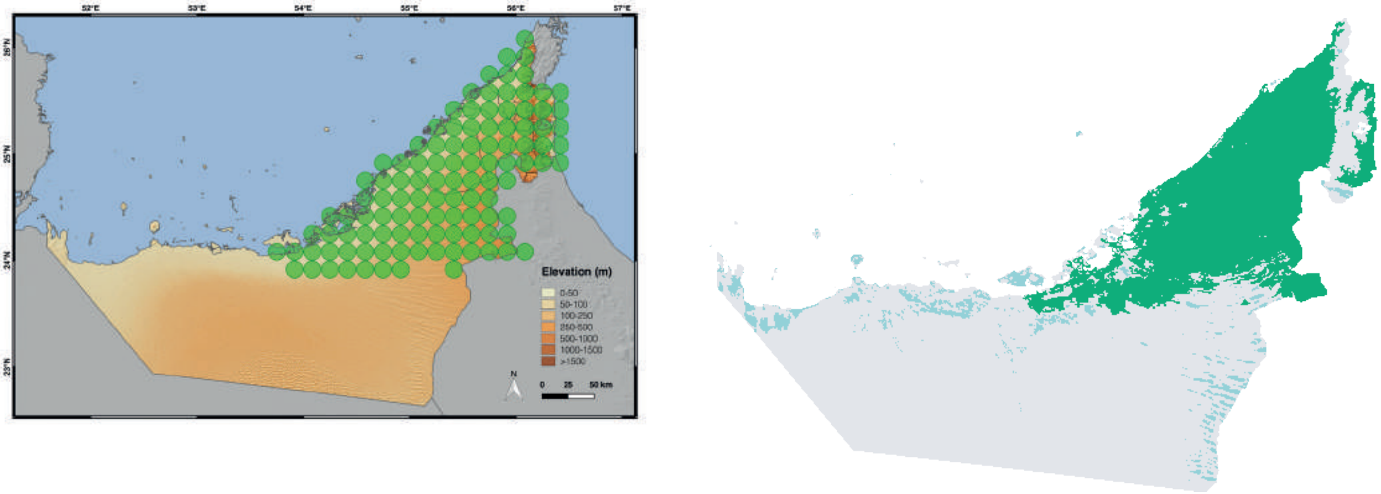

Mesalina brevirostris

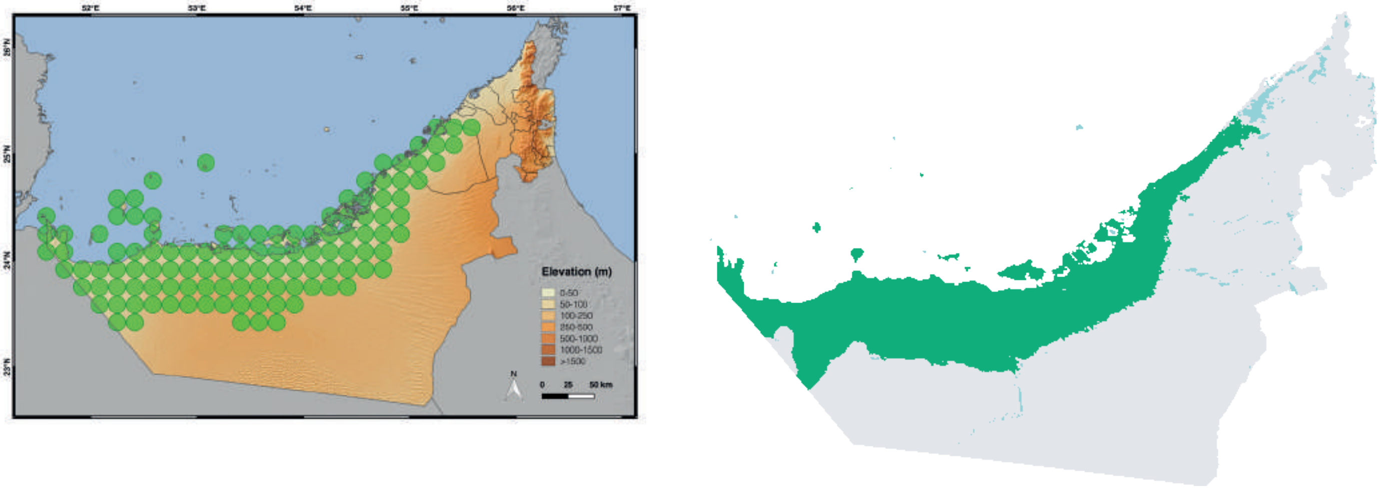

Species distribution models

Omanosaura cyanura

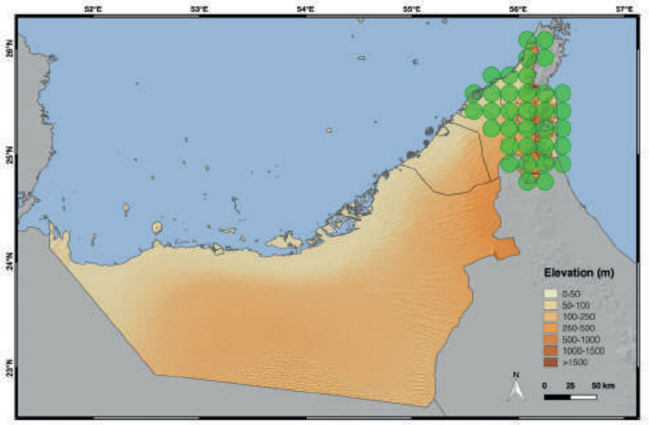

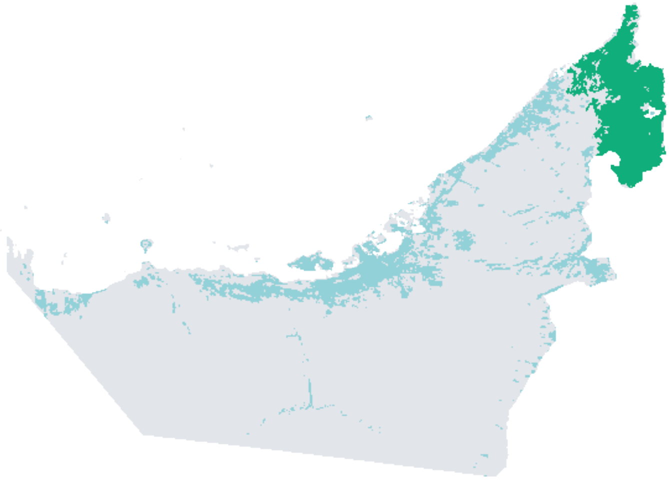

Omanosaura jayakari

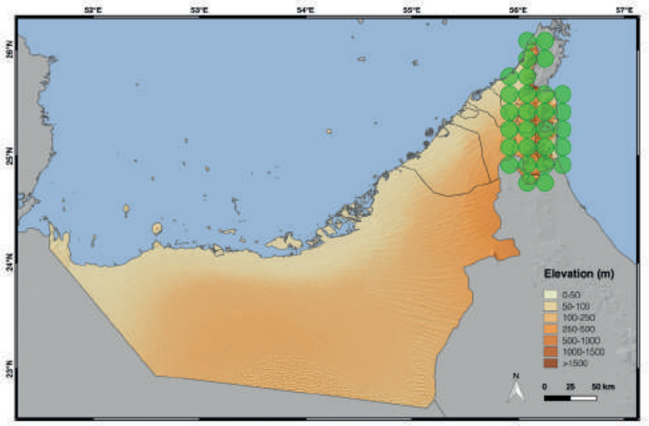

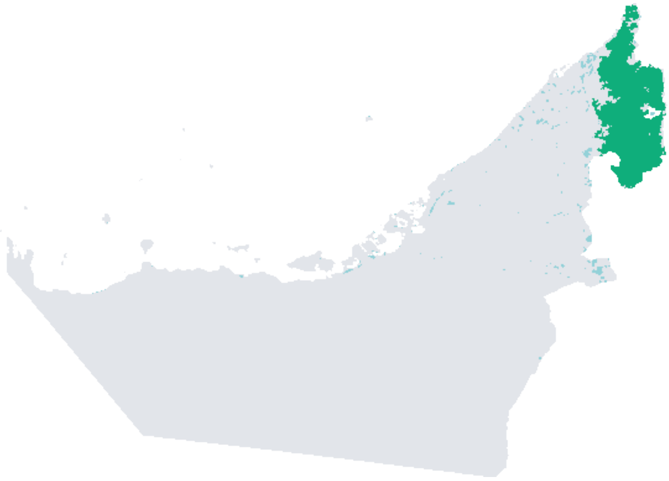

Ablepharus pannonicus

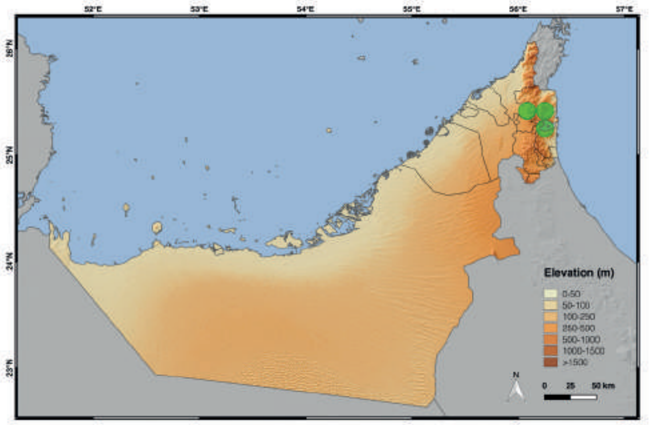

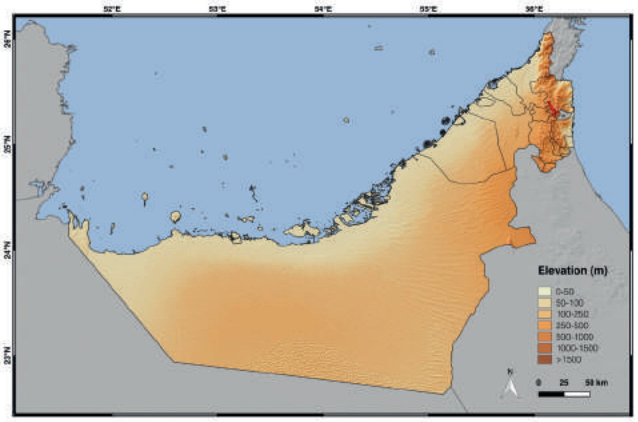

Species distribution models

Chalcides ocellatus ocellatus

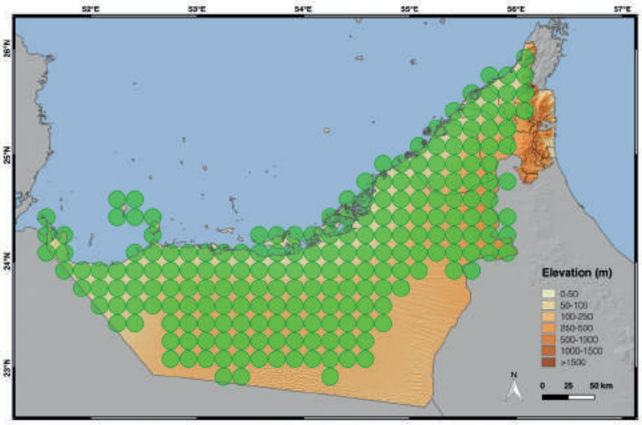

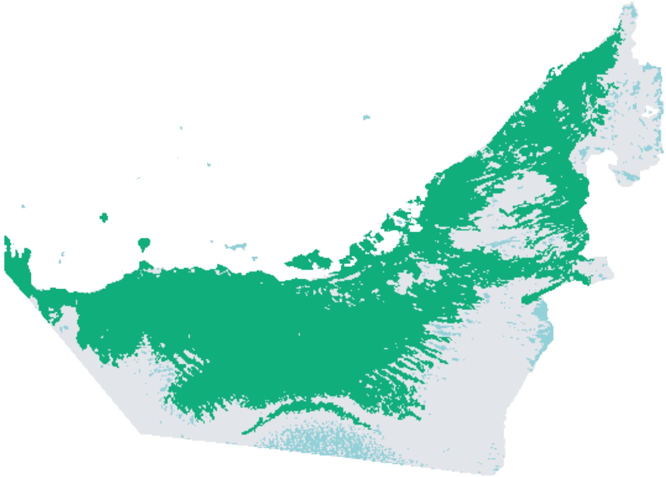

Heremites septemtaeniatus

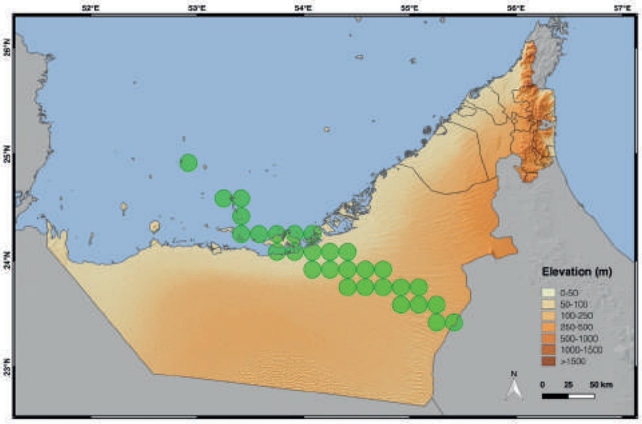

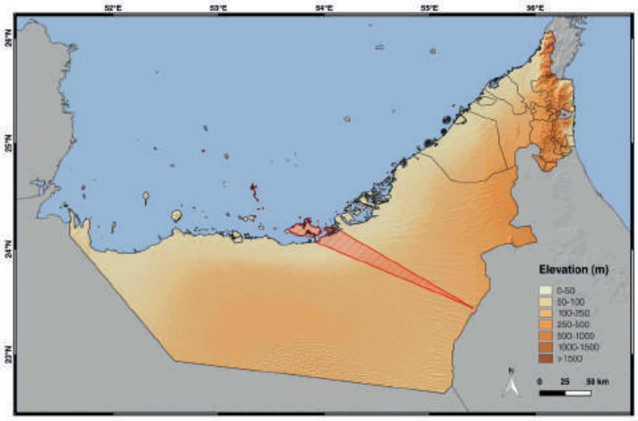

Scincus mitranus

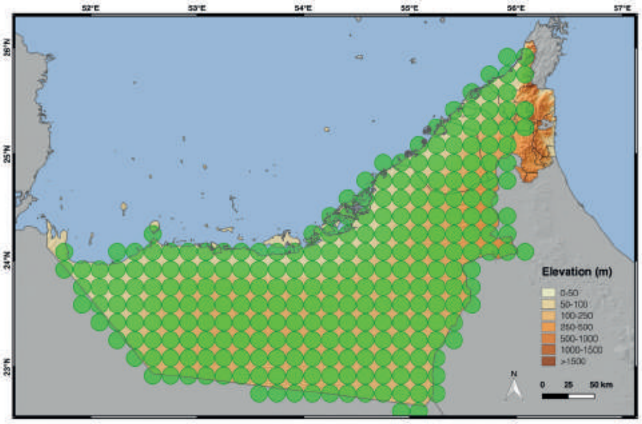

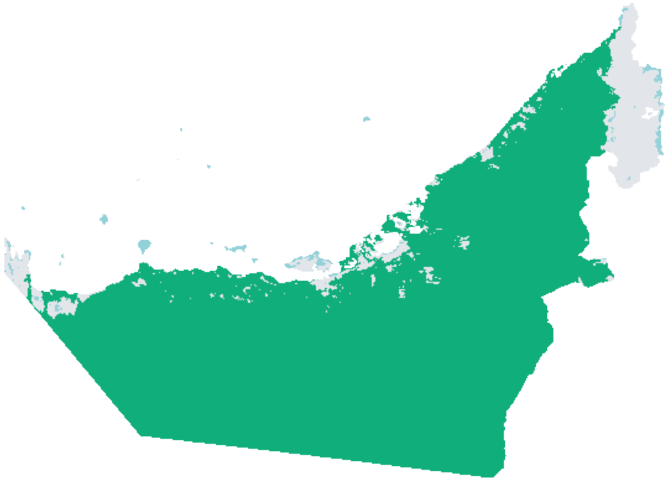

Species distribution models

Scincus scincus conirostris

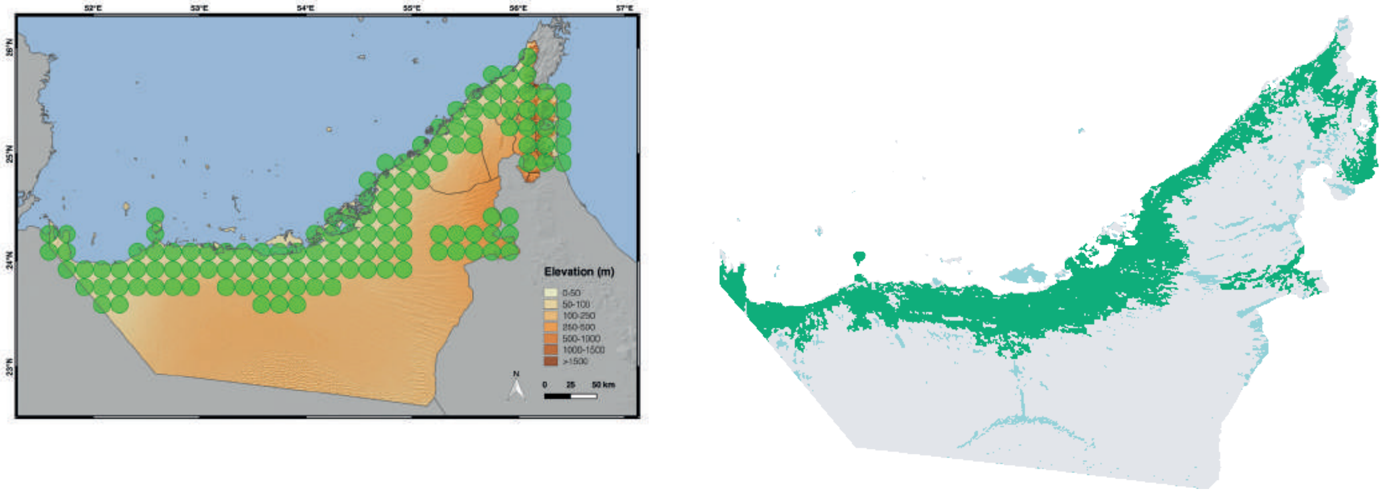

Trachylepis tessellata

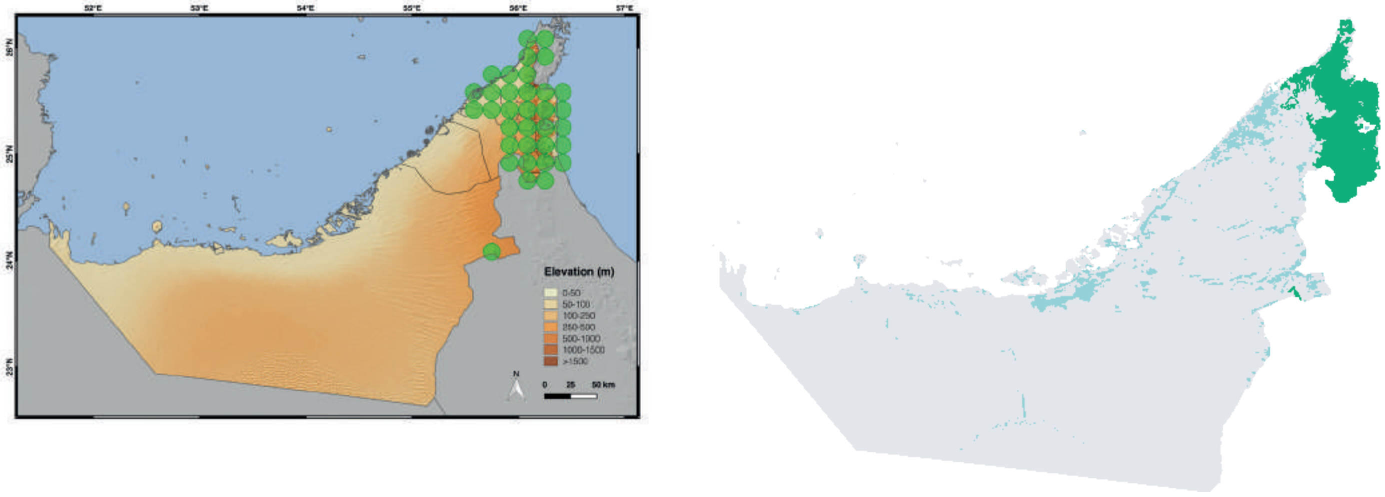

Diplometopon zarudnyi

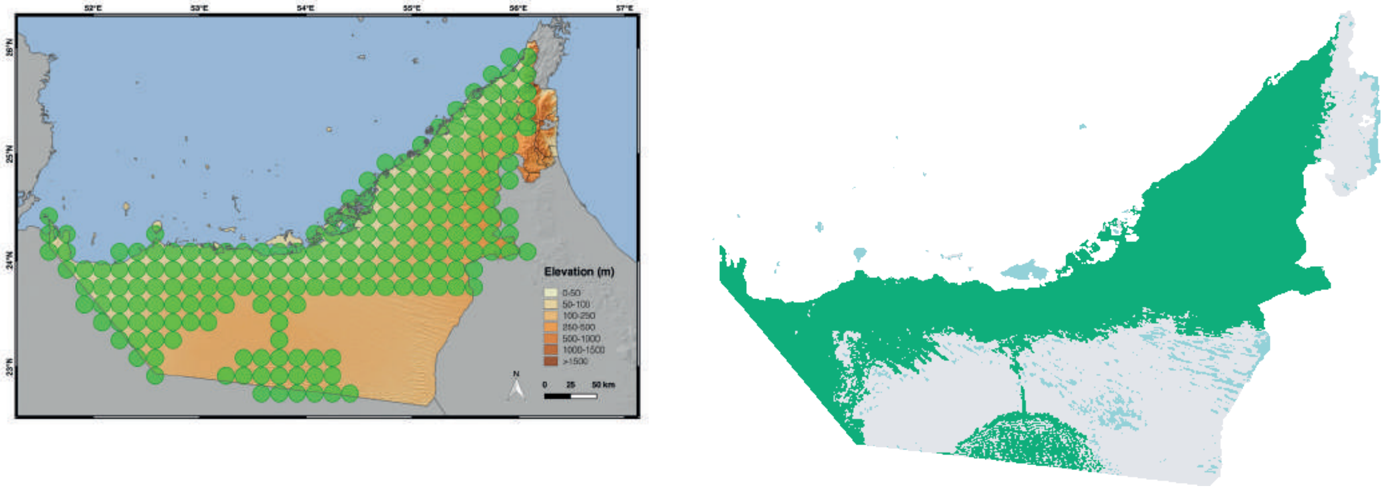

S76: Species distribution models

Varanus griseus griseus

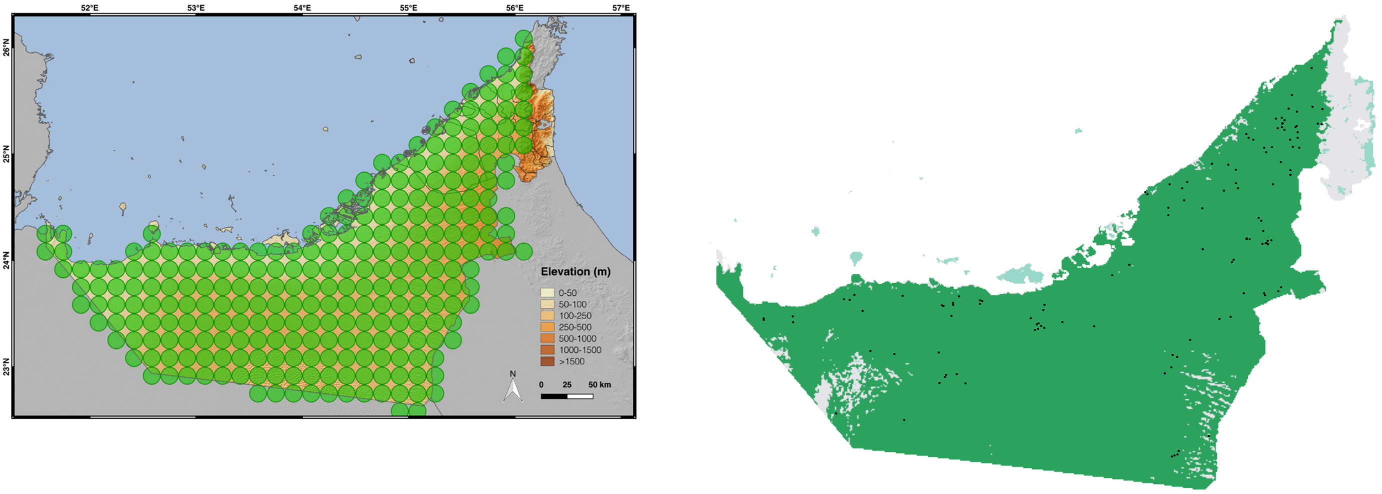

Eryx jayakari

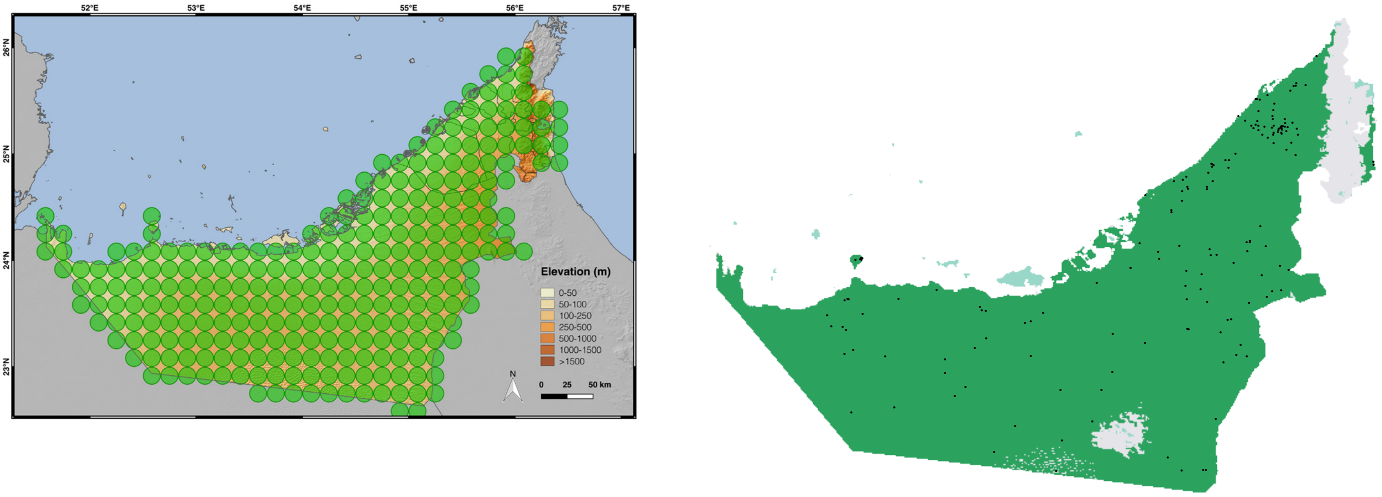

Lytorhynchus diadema diadema

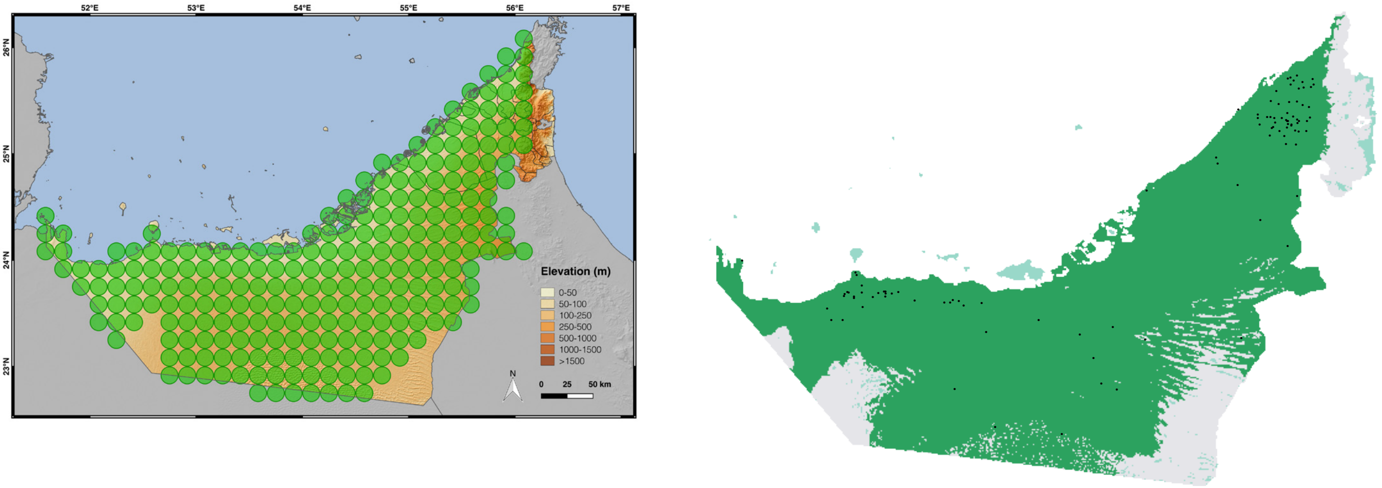

Species distribution models

Platyceps rhodorachis rhodorachis

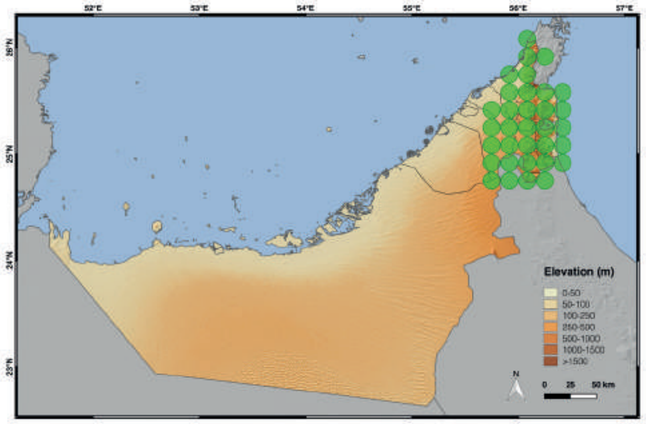

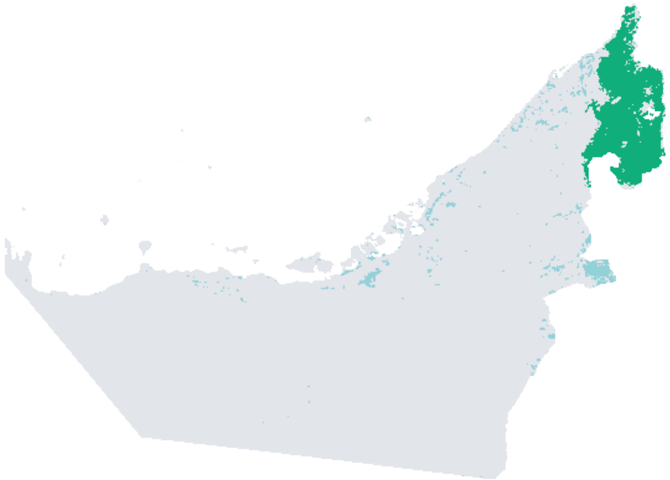

Platyceps ventromaculatus

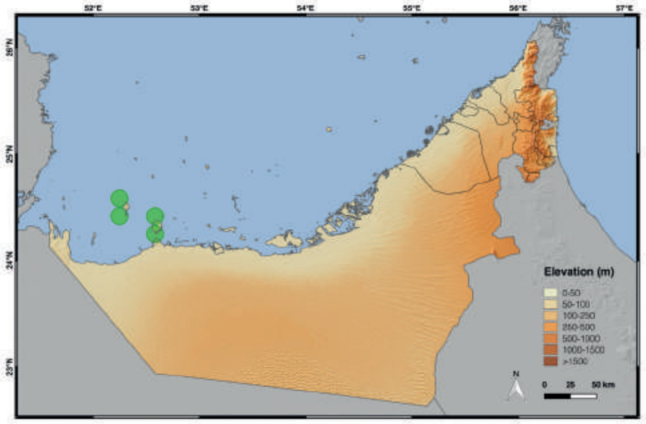

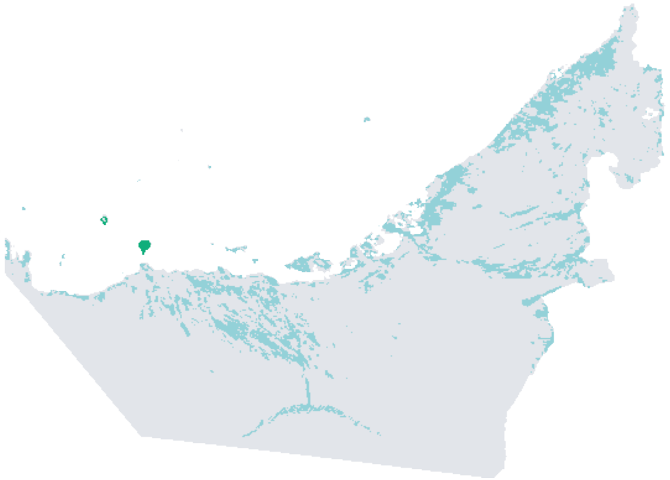

Spalerosophis diadema cliffordii

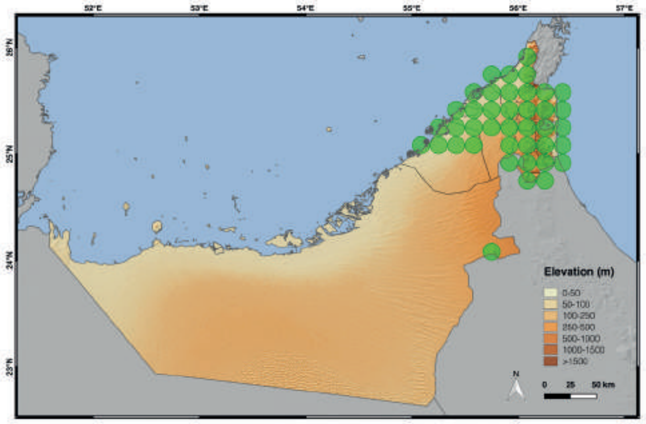

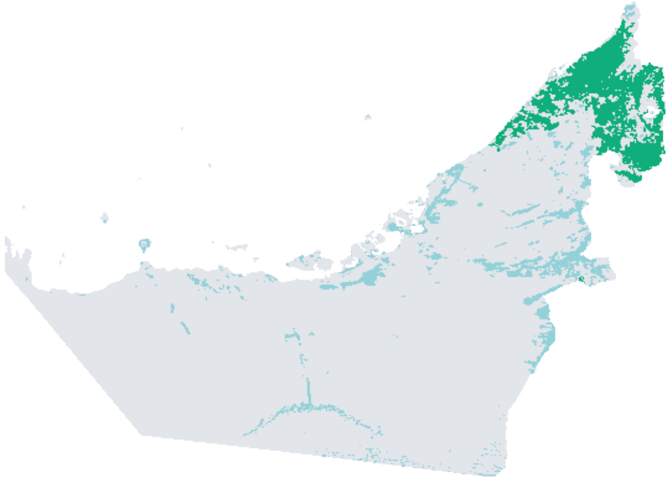

Species distribution models

Telescopus dhara dhara

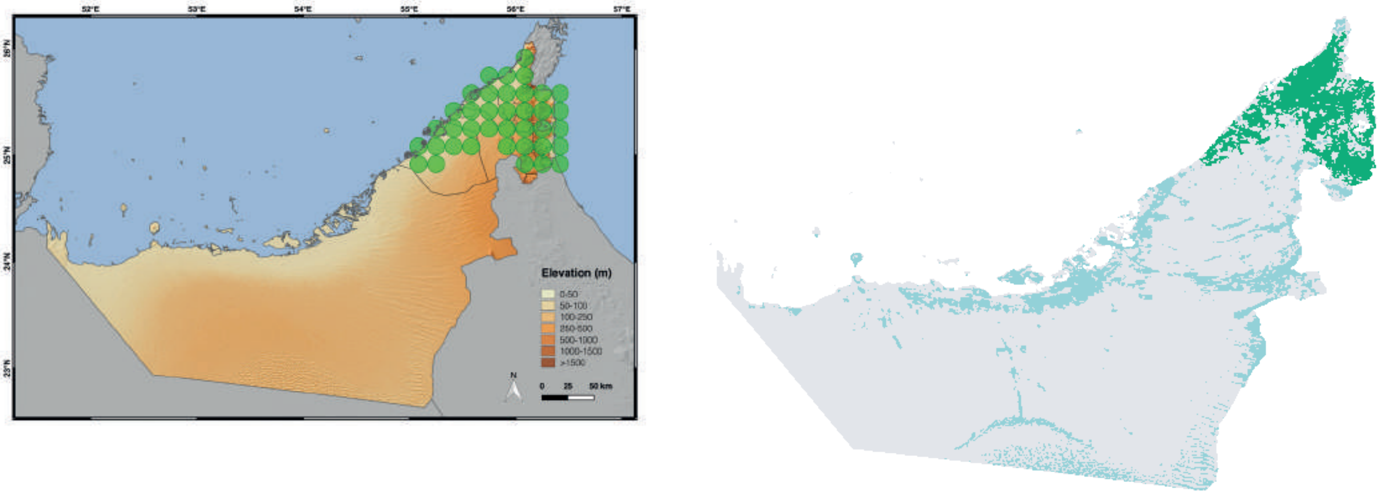

Psammophis schokari

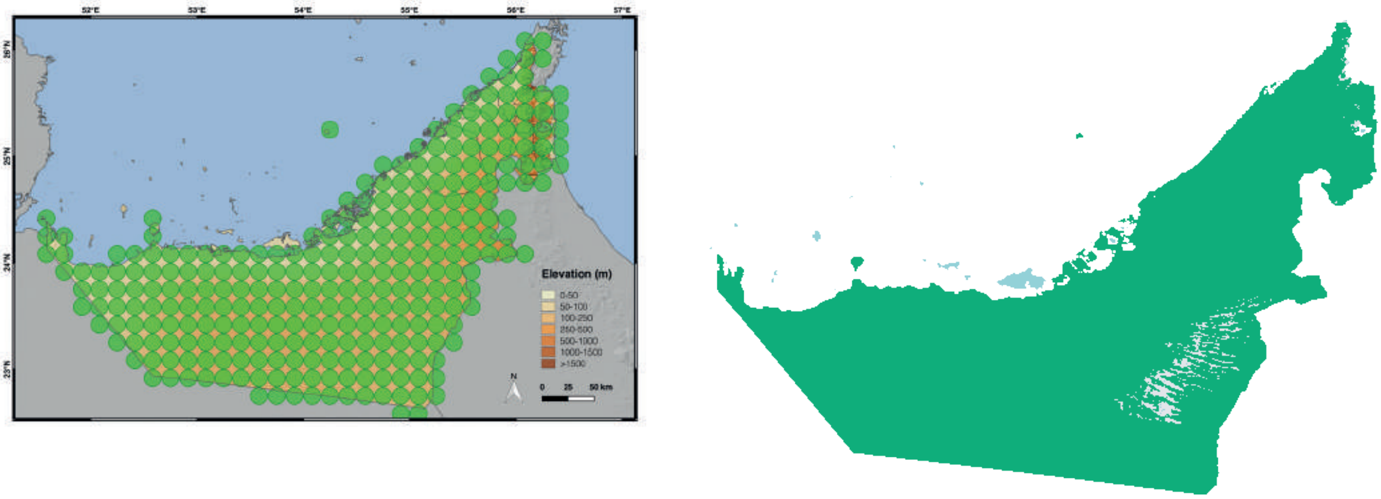

Rhagerhis moilensis

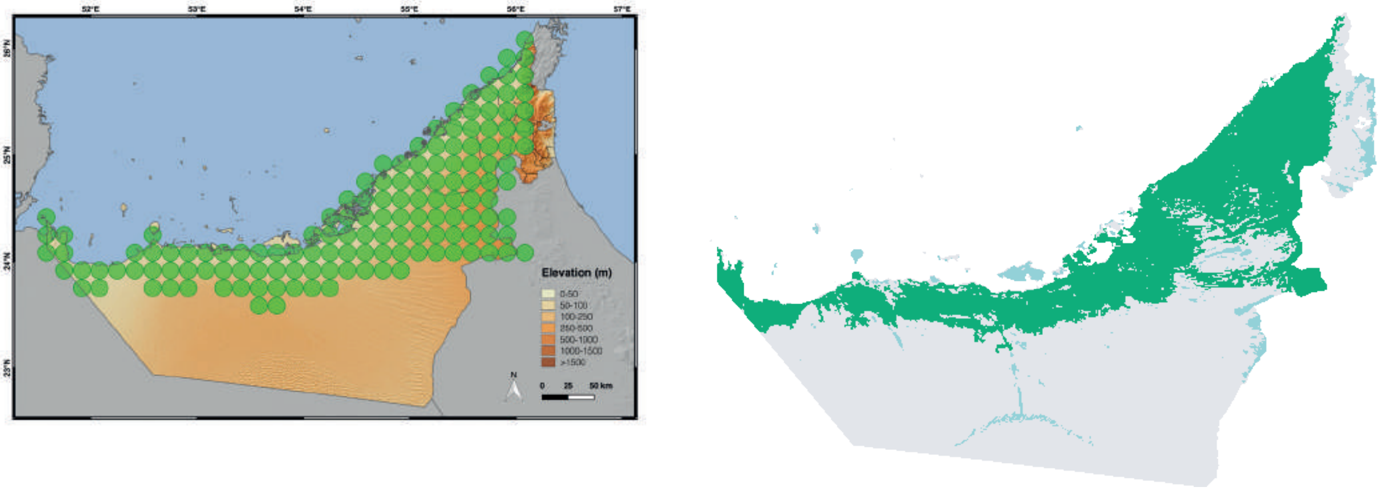

Species distribution models

Myriopholis macrorhyncha

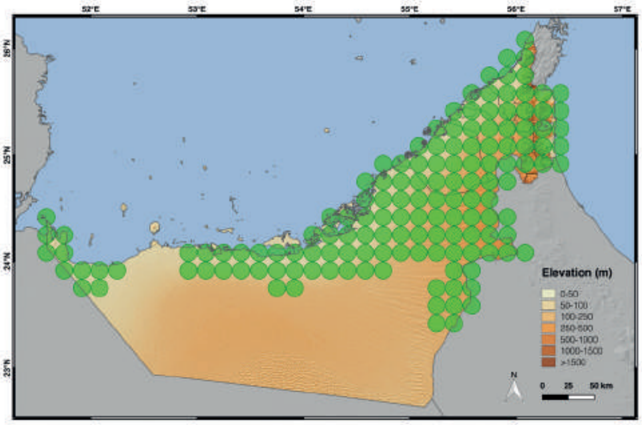

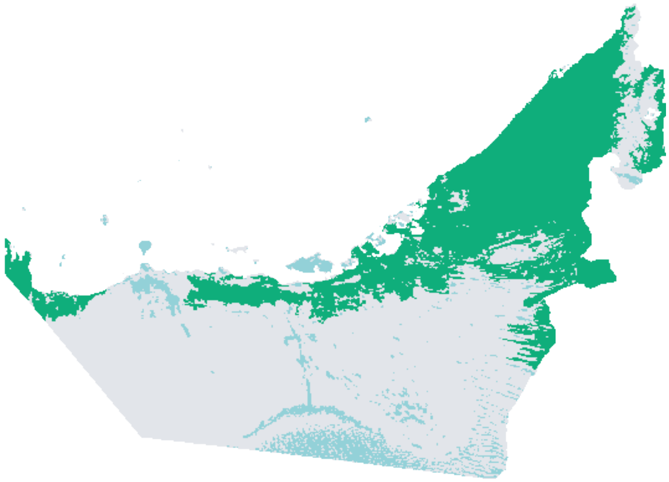

Indotyphlops braminus

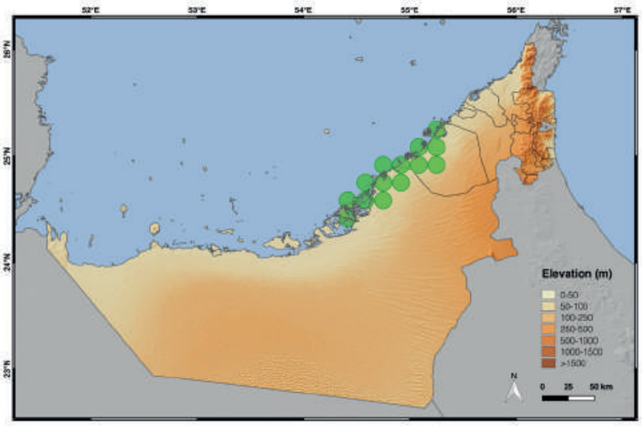

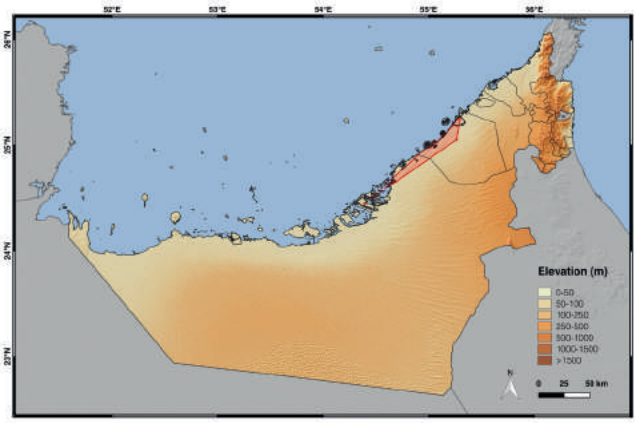

Cerastes gasperettii gasperettii

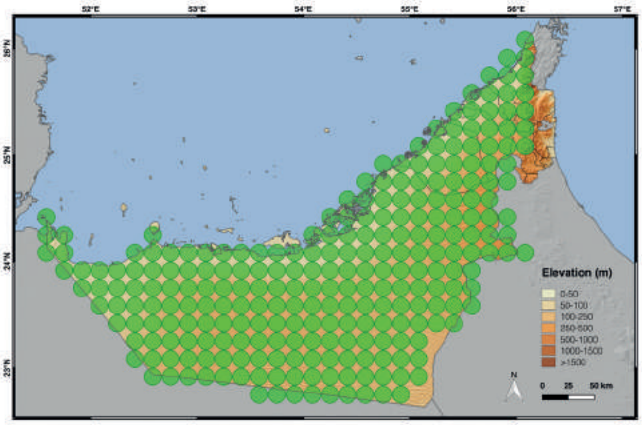

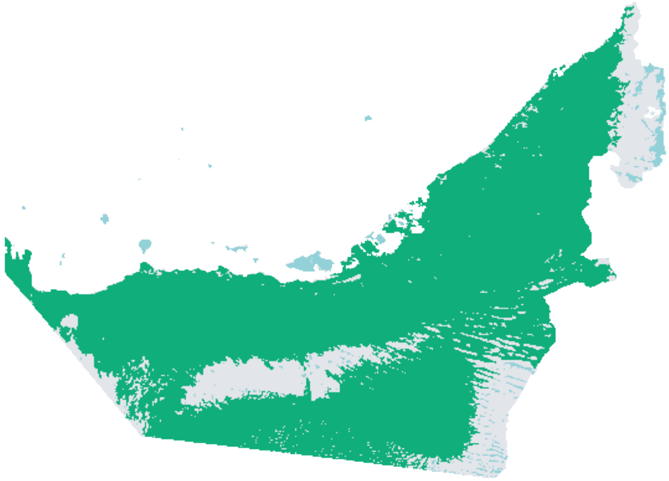

Species distribution models

Echis carinatus sochureki

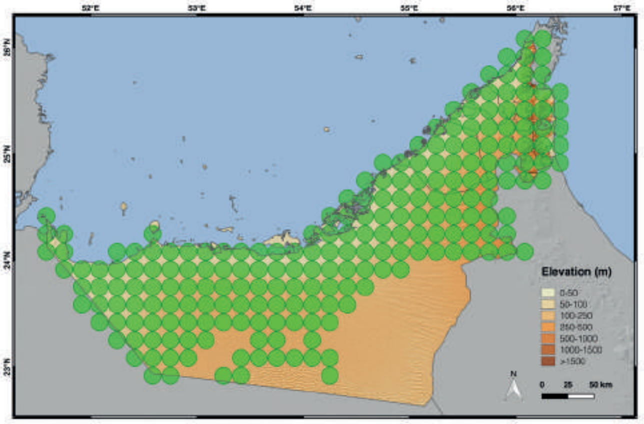

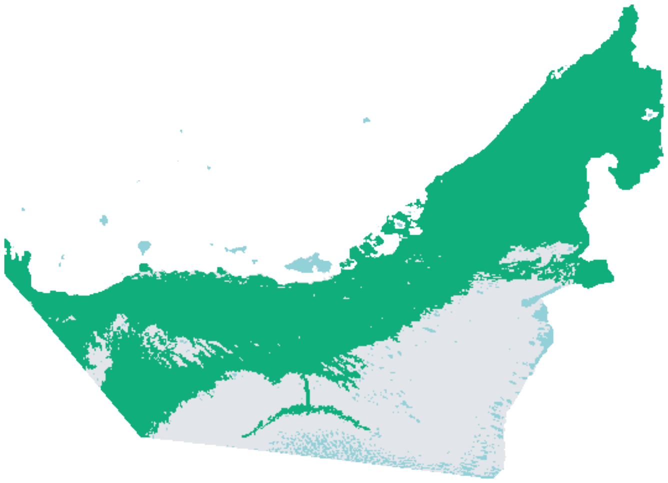

Echis omanensis

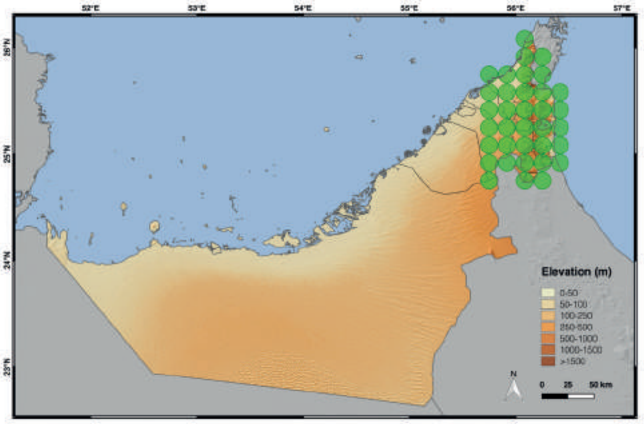

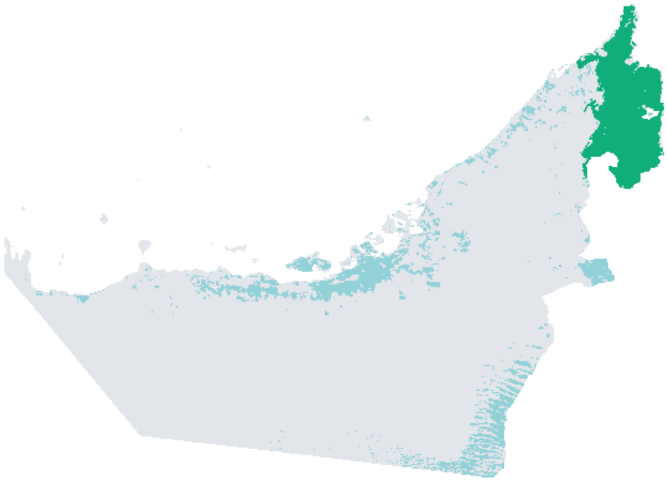

Pseudocerastes persicus

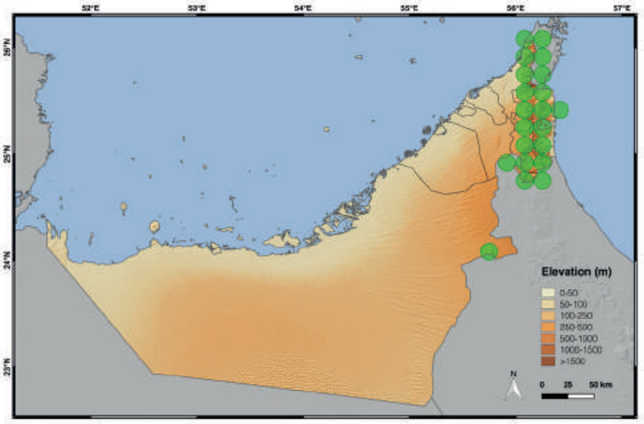

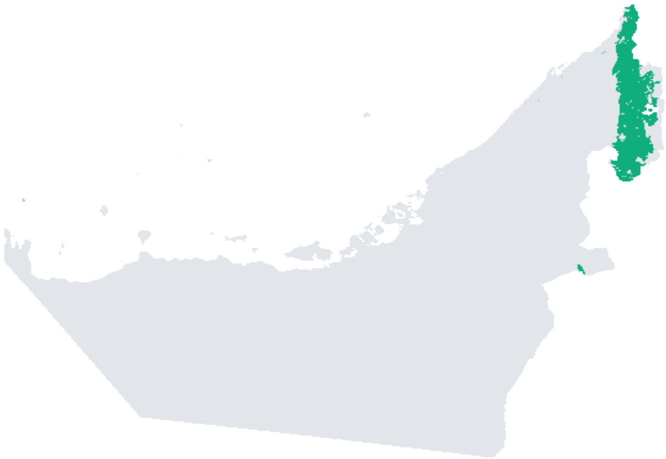

Supplement: S1 Appendix — An appendix showing photographs, distribution and ecological preferences of the 60 species of terrestrial reptiles of the UAE. Species distribution models (SDMs) inferred for each species are also included. Credits for maps: OpenStreetMap contributors, SRTM. (PDF) [file pone.0216273.s001.pdf]
