# Supplementary material for: An integrative assessment of the diversity, phylogeny, distribution, and conservation of the terrestrial reptiles (Sauropsida, Squamata) of the United Arab Emirates
Source: PLoS One. 2019 May 2;14(5):e0216273. doi: 10.1371/journal.pone.0216273 (PMC6497385; doi:10.1371/journal.pone.0216273)
Supplement: S2 Appendix — List of the 13 calibration points used to calibrate the phylogenetic tree of Squamata and including all the UAE terrestrial species. Each calibration point includes the author and all the parameters used to implement the calibration point in BEAST. (PDF) [file pone.0216273.s002.pdf]

S2 APPENDIX. CALIBRATION POINTS

1- The split between *Podarcis pityusensis* and *P. lilfordi* during the Messinian Salinity Crisis. Normal distribution, mean: 5.32, stdev: 0.05 (Carranza and Arnold, 2012; Tamar et al., 2016).

2- The split between the Asian *Naja* clade and its African sister clade dates back to a minimum age of 16 my based on the presence of characteristic apomorphies of the Asian clade in the fossil record (Szyndlar and Rage, 1990; Wüster et al., 2008). Lognormal distribution, offset: 16, mean: 1, stdev: 1.

3- Fossil evidence suggests that the initial divergence of the Eurasian viper clade (excluding *Pseudocerastes* and *Eristicophis*) had begun by 20 my (Szyndlar and Rage, 1999). Lognormal distribution, offset: 20, mean: 1, stdev: 1.

4- The initial divergence of three South American populations of the Neotropical pitviper genus *Porthidium*, which almost certainly invaded South America and diverged there after the uplift of the Isthmus of Panamá, approximately 3.5 my (Wüster et al., 2002). Normal distribution, mean: 3.5, stdev: 1.

5- The fossil *Tuberculacerata* from the Medicine Pole Hills in the Chadronian Formation (33.9-38 Ma) of North Dakota (Smith, 2006) was used to calibrate the node defining crown Phrynosomatidae at approximately 36 my (Smith 2006 suggests the fossils are mid-Chadronian in age). Lognormal distribution, offset: 32.4, mean: 1.2, stdev:1.4.

6- The split between *Phelsuma ornata* from Mauritius and *Phelsuma inexpectata* from Reunion islands. This event was set to a soft maximum of 8.9 my based on the age of the oldest rocks of Mauritius, the oldest island in the Mascarenes (Moore et al., 2011). The prior was set by an Exponential distribution, offset: 0, mean: 3.

7- The initiation of the diplodactyloid radiation in New Caledonia was placed as a soft maximum of 37 my, because geological and biological data show that the island was probably under water until this time (Garcia-Porta and Ord, 2013; Nattier et al., 2011; Papadopoulou et al., 2013; Pillon, 2012). A Normal distribution, mean: 20 Ma, stdev: 10

8- The divergence between Hydrosaurinae and Amphibolurinae from Agaminae and Draconinae during the Late Cretaceous. Normal distribution, mean: 70, stdev: 3.5 (Amer and Kumazawa, 2005; Townsend et al., 2011).

9- The split between *Gallotia* and *Psammodromus*, coinciding with de age of the oldest islands Fuerteventura and Lanzarote. Normal Distribution, mean: 18, stdev: 2 (Carranza and Arnold, 2012; Tamar et al., 2016).

10- The stem amphisbaenian Hodzhhakulia (Gao and Nessov, 1998) from the Aptian-Albian boundary (112 ± 1.0 my) was used to calibrate the node defining Lacertibaenia (Amphisbaenia and Lacertidae). Lognormal distribution, offset: 110, mean: 1.8, stdev:1.3.

11- The stem scincomorph *Balnealacerta* (Evans, 1998) from the Middle Jurassic (164.7 ± 4.0 my) was used to calibrate the node defining Unidentata (all squamates except Gekkota and Dibamidae). We note that Scincomorpha is traditionally defined to encompass Scincidae, Cordylidae, Xantusiidae, Teiidae, Gymnophthalmidae, and Lacertidae, which is a paraphyletic assemblage in our tree. There is some disagreement amongst morphologists/paleontologists as to which subgroup(s) among these taxa Balnealacerta and other "scincomorphs" from the same site are more closely related to. From the possible alternatives, we chose the deepest (i.e., more conservative in terms of pushing divergence dates further into the past) node for our calibration point. Lognormal distribution, offset: 161, mean: 1.8, stdev: 1.

12- The oldest known rhynchocephalians (Evans et al., 2001; Sues and Olsen, 1990) from the Ladinian-Carnian boundary (228 ± 2.0 my) were used to calibrate the node defining Lepidosauria

(Rhynchocephalia, represented by *Sphenodon punctatus*, and Squamata, all other sampled taxa). Lognormal distribution, offset: 224, mean: 2.6, stdev: 1.2.

13- The divergence of Leiolepidinae from the remaining agamids during the Late Cretaceous. Normal distribution, mean: 82, stdev: 3.5 (Amer and Kumazawa, 2005; Townsend et al., 2011; Wiens et al., 2006).

REFERENCES

Amer, S.A.M., Kumazawa, Y., 2005. Mitochondrial DNA sequences of the Afro-Arabian spiny-tailed lizards (genus *Uromastyx*; family Agamidae): phylogenetic analyses and evolution of gene arrangements. Biol. J. Linn. Soc. 85, 247–260.

Carranza, S., Arnold, E.N., 2012. A review of the geckos of the genus *Hemidactylus* (Squamata: Gekkonidae) from Oman based on morphology, mitochondrial and nuclear data, with descriptions of eight new species. Zootaxa. 3378, 1–95.

Evans, S.E., 1998. Crown-group lizards from the Middle Jurassic of Britain. Palaeontogr. A 250, 1–32.

Evans, S.E., Prasad, G.V.R., Manhas, B.K., 2001. Rhynchocephalians (Diapsida: Lepidosauria) from the Jurassic Kota Formation of India. Zool. J. Linn. Soc. 133, 309–334.

Gao, K.-Q., Nessov, L.A., 1998. Early Cretaceous Squamates from the Kyzylkum Desert, Uzbekistan.(With 4 figures). Neues Jahrb. fur Geol. und Palaontologie-Abhandlungen 207, 289–310.

Garcia-Porta, J., Ord, T.J., 2013. Key innovations and island colonization as engines of evolutionary diversification: a comparative test with the Australasian diplodactyloid geckos. J. Evol. Biol. 26, 2662–2680.

Moore, J., White, W.M., Paul, D., Duncan, R.A., Abouchami, W., Galer, S.J.G., 2011. Evolution of shieldbuilding and rejuvenescent volcanism of Mauritius. J. Volcanol. Geotherm. Res. 207, 47–66.

Nattier, R., Robillard, T., Desutter-Grandcolas, L., Couloux, A., Grandcolas, P., 2011. Older than New Caledonia emergence? A molecular phylogenetic study of the Eneopterine crickets (Orthoptera: Grylloidea). J. Biogeogr. 38, 2195–2209.

Papadopoulou, A., Cardoso, A., Gómez-Zurita, J., 2013. Diversity and diversification of Eumolpinae (Coleoptera: Chrysomelidae) in New Caledonia. Zool. J. Linn. Soc. 168, 473–495.

Pillon, Y., 2012. Time and tempo of diversification in the flora of New Caledonia. Bot. J. Linn. Soc. 170, 288–298.

Smith, K.T., 2006. A diverse new assemblage of Late Eocene squamates (Reptilia) from the Chadron formation of North Dakota, USA. Palaeontol. Electron. 9, 1–44.

Sues, H.-D., Olsen, P.E., 1990. Triassic vertebrates of Gondwanan aspect from the Richmond Basin of Virginia. Science. 249, 1020–1023.

Szyndlar, Z., Rage, J.C., 1999. Oldest fossil vipers (Serpentes: Viperidae) from the old World. Kaupia 8, 9–20.

Szyndlar, Z., Rage, J.C., 1990. West Paleartic cobras of the genus *Naja* (serpentes: Elapidae): interrelationships among extinct and extant species. Amphibia-Reptilia. 11, 385–400.

Tamar, K., Carranza, S., Sindaco, R., Moravec, J., Trape, J.F., Meiri, S., 2016. Out of Africa: Phylogeny and biogeography of the widespread genus *Acanthodactylus* (Reptilia: Lacertidae). Mol. Phylogenet. Evol. 103, 6–18. https://doi.org/10.1016/j.ympev.2016.07.003

Townsend, T.M., Mulcahy, D.G., Noonan, B.P., Sites Jr, J.W., Kuczynski, C.A., Wiens, J.J., Reeder, T.W., 2011. Phylogeny of iguanian lizards inferred from 29 nuclear loci, and a

comparison of concatenated and species-tree approaches for an ancient, rapid radiation. Mol. Phylogenet. Evol. 61, 363–380.

Wiens, J.J., Brandley, M.C., Reeder, T.W., 2006. Why does a trait evolve multiple times within a clade? Repeated evolution of snakelike body form in squamate reptiles. Evolution. 60, 123–141.

Wüster, W., Peppin, L., Pook, C.E., Walker, D.E., 2008. A nesting of vipers: Phylogeny and historical biogeography of the Viperidae (Squamata: Serpentes). Mol. Phylogenet. Evol. 49, 445–459. https://doi.org/10.1016/j.ympev.2008.08.019

Wüster, W., Salomão, M.D.G., Quijada-Mascareñas, J.A., Thorpe, R.S., Duckett, G.J., Puorto, M.G., Theakston, R., Warrell, D., 2002. Origins and evolution of the South American pitviper fauna: evidence from mitochondrial DNA sequence analysis. Biol. Vipers 111–128.
