# Supplementary figures and images for: An integrative assessment of the diversity, phylogeny, distribution, and conservation of the terrestrial reptiles (Sauropsida, Squamata) of the United Arab Emirates
Source: PLoS One. 2019 May 2;14(5):e0216273. doi: 10.1371/journal.pone.0216273 (PMC6497385; doi:10.1371/journal.pone.0216273)

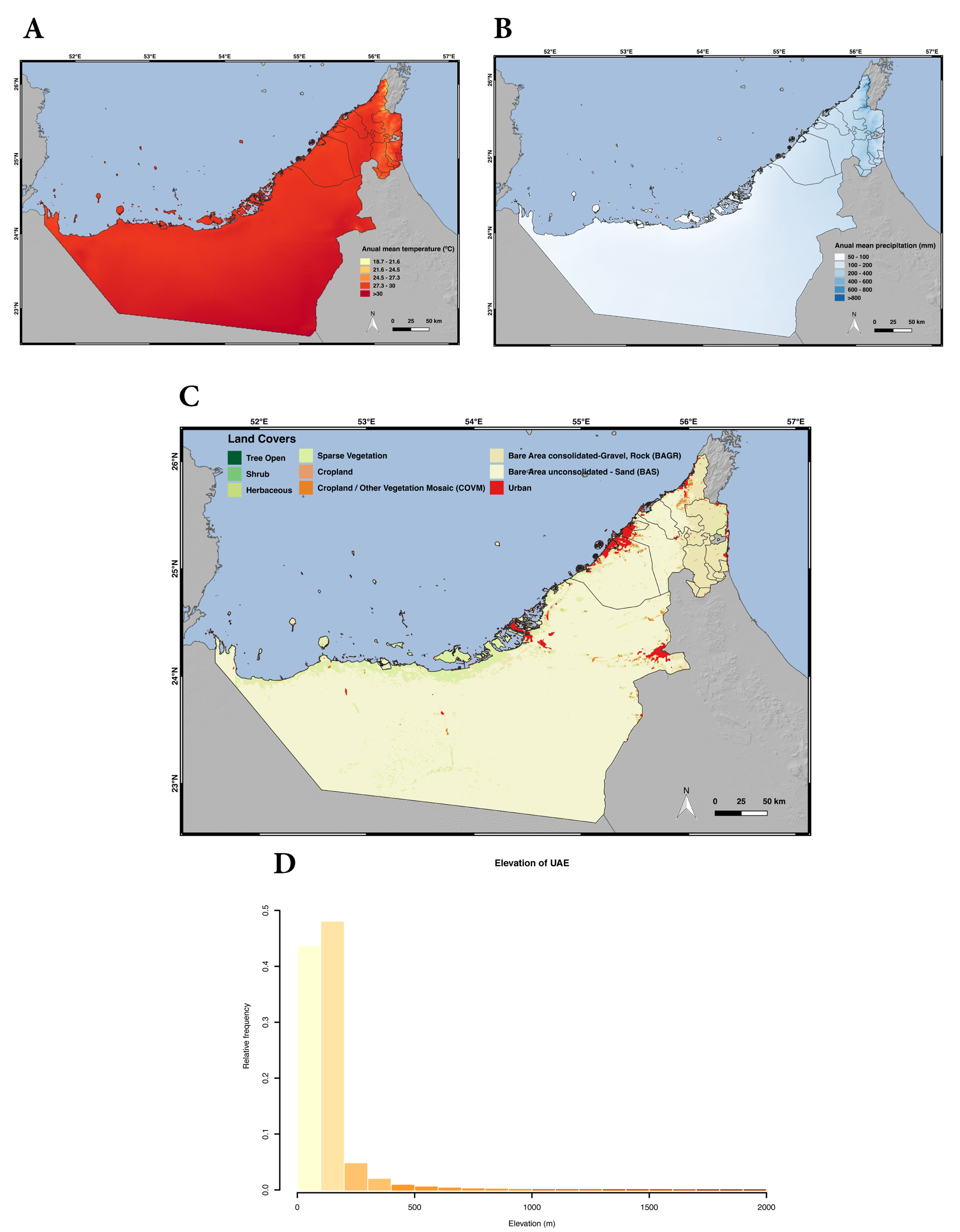

Supplement: S1 Fig — A) Map of annual mean temperature in °C (BIO1); B) Map of annual mean precipitation in mm (BIO12); C) Map of land cover types (as of 2008); D) Graph of the frequency of elevations divided into 100-m bins. Credits: OpenStreetMap contributors, SRTM. (TIF) [file pone.0216273.s004.tif]

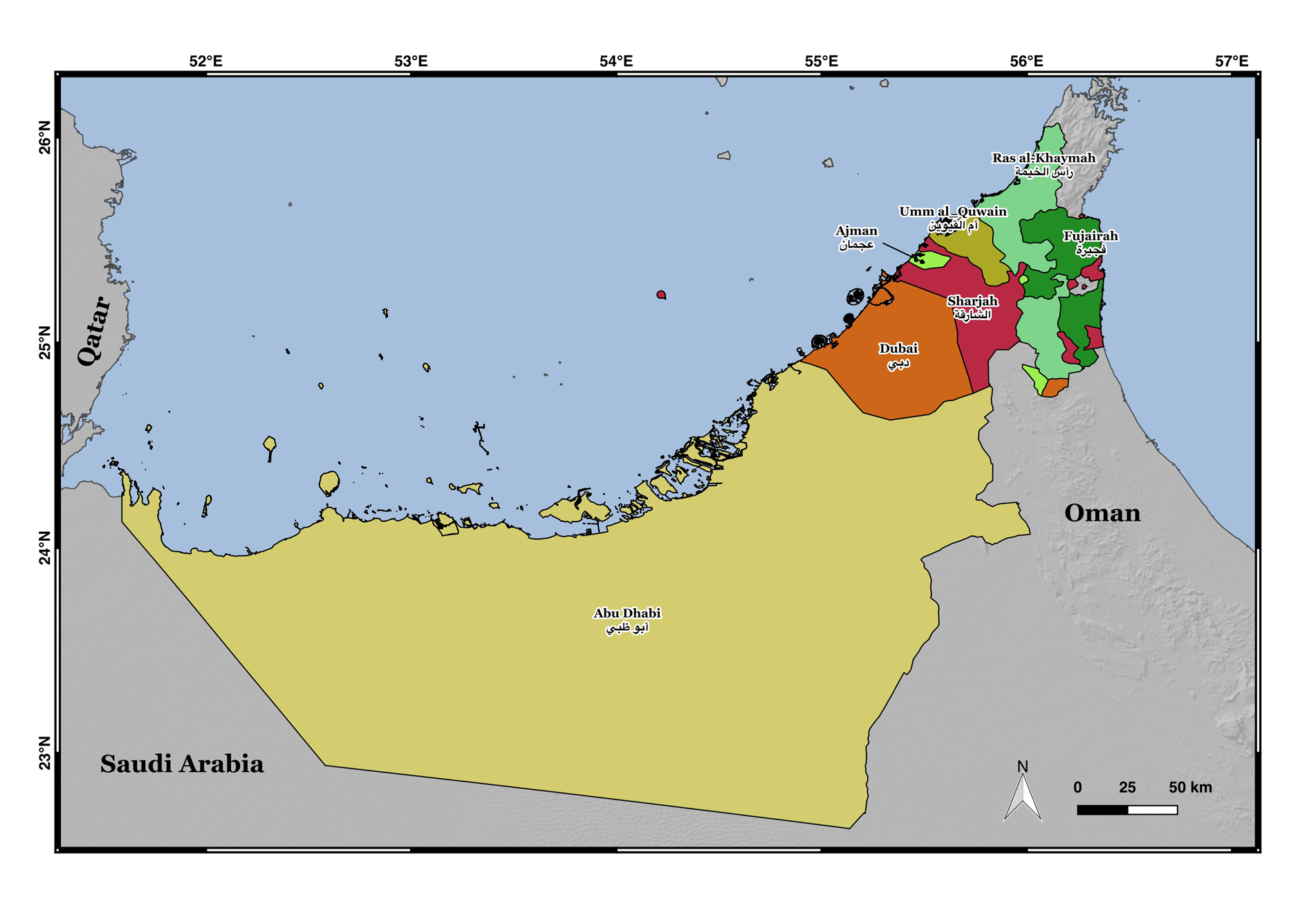

Supplement: S2 Fig — Map showing the political borders of the UAE and among the seven emirates. Credits: OpenStreetMap contributors, SRTM. (TIF) [file pone.0216273.s005.tif]

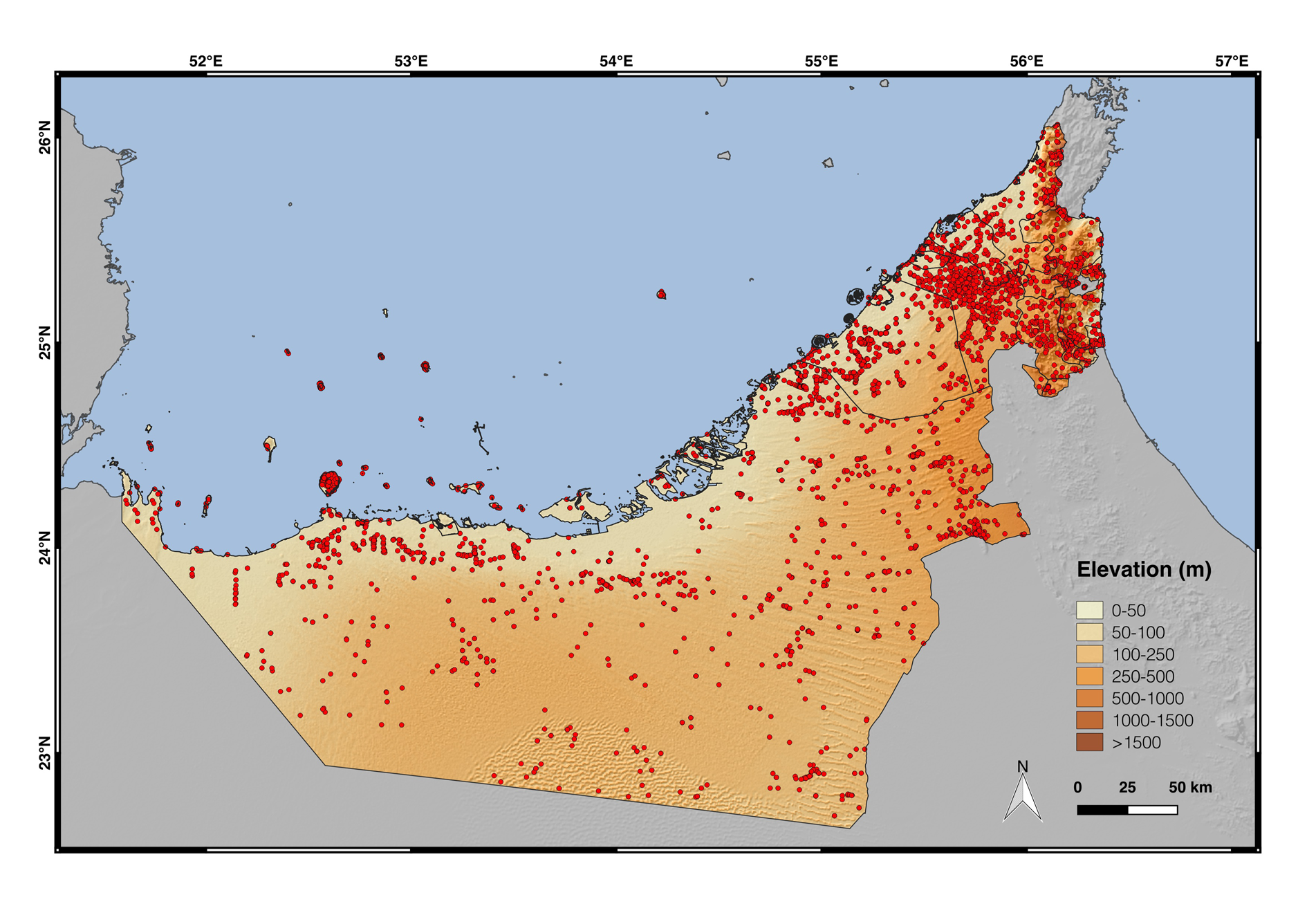

Supplement: S3 Fig — Map with all the 5,535 occurrence points of UAE terrestrial reptiles used in the present study. Credits: OpenStreetMap contributors, SRTM. (TIF) [file pone.0216273.s006.tif]

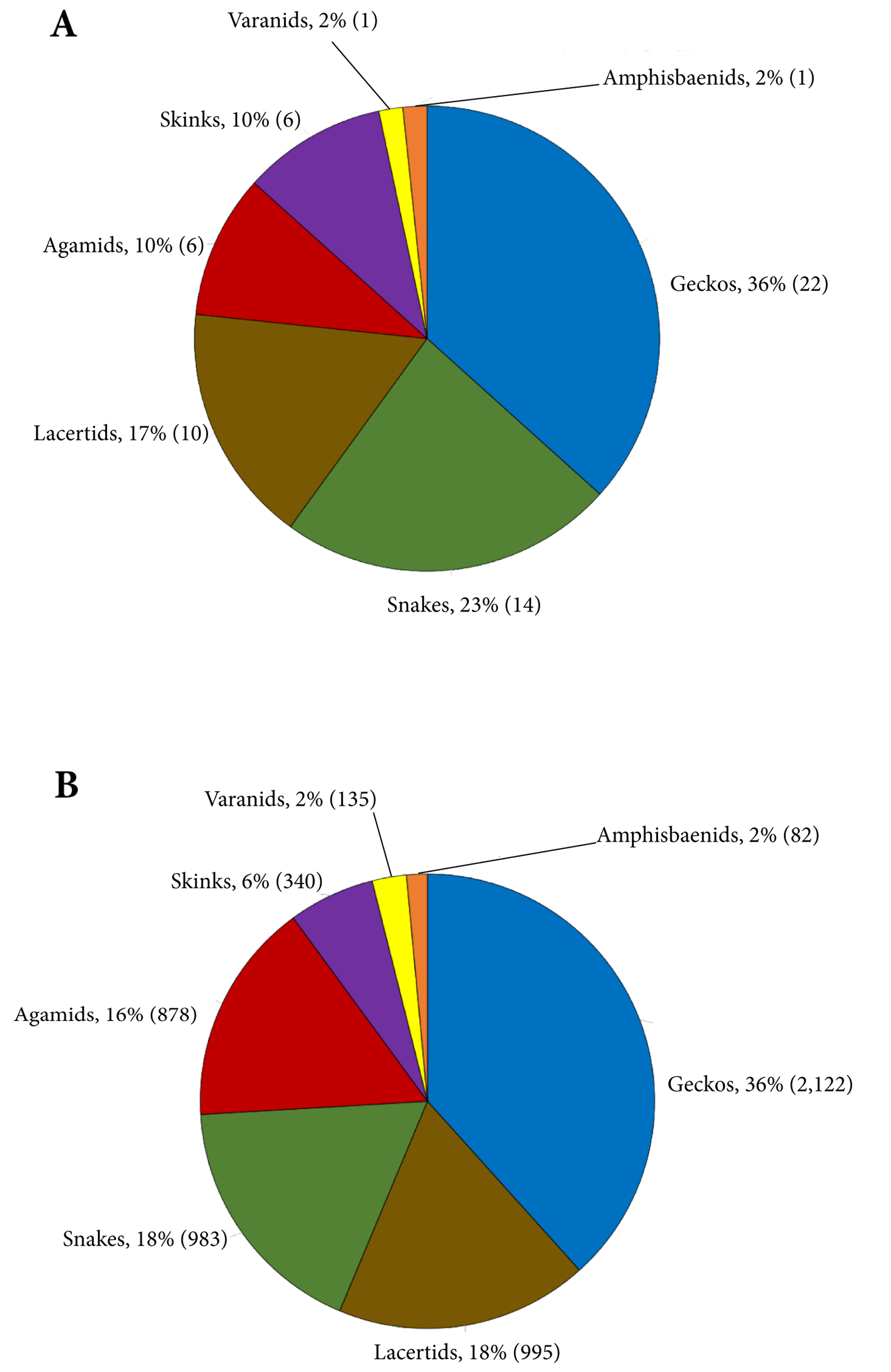

Supplement: S4 Fig — Pie charts showing higher taxonomic composition of the UAE terrestrial reptile species (A) and the number of observations for each of the taxonomic groups used in this study (B). Numbers in parenthesis are the number of species (A) and observations (B). (TIF) [file pone.0216273.s007.tif]

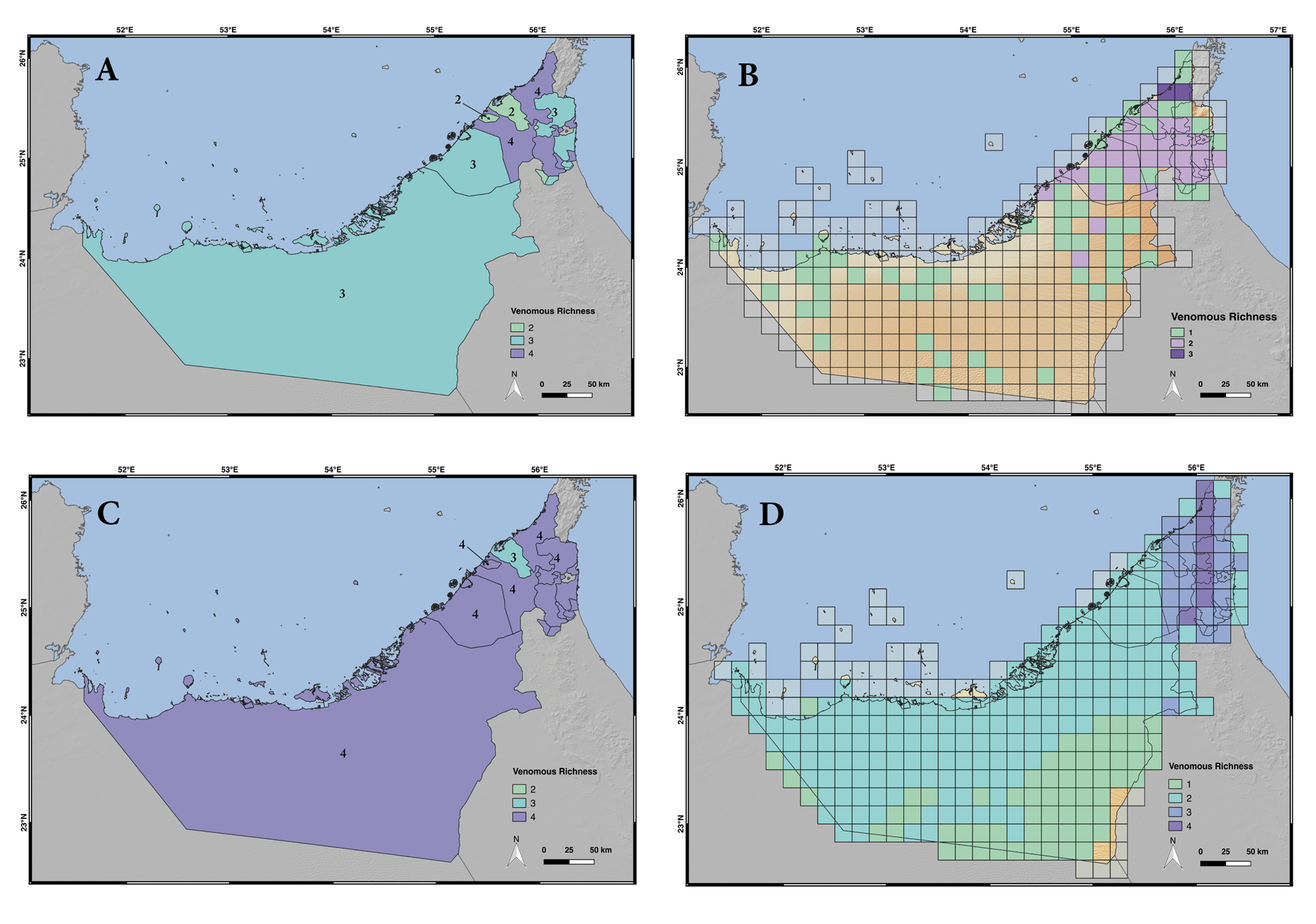

Supplement: S5 Fig — A) Venomous species richness by emirate inferred with the occurrence point data; B) Venomous species richness by a 10 arc-min grid inferred with the occurrence point data; C) Venomous species richness by emirate inferred with the species distribution models; D) Venomous species richness by a 10 arc-min grid inferred with the species distribution models. The four species of medically important venomous species of UAE terrestrial reptiles are the snakes Cerastes gasperettii gasperettii, Echis carinatus sochureki, Echis omanensis and Pseudocerastes persicus. Credits: OpenStreetMap contributors, SRTM. (TIF) [file pone.0216273.s008.tif]

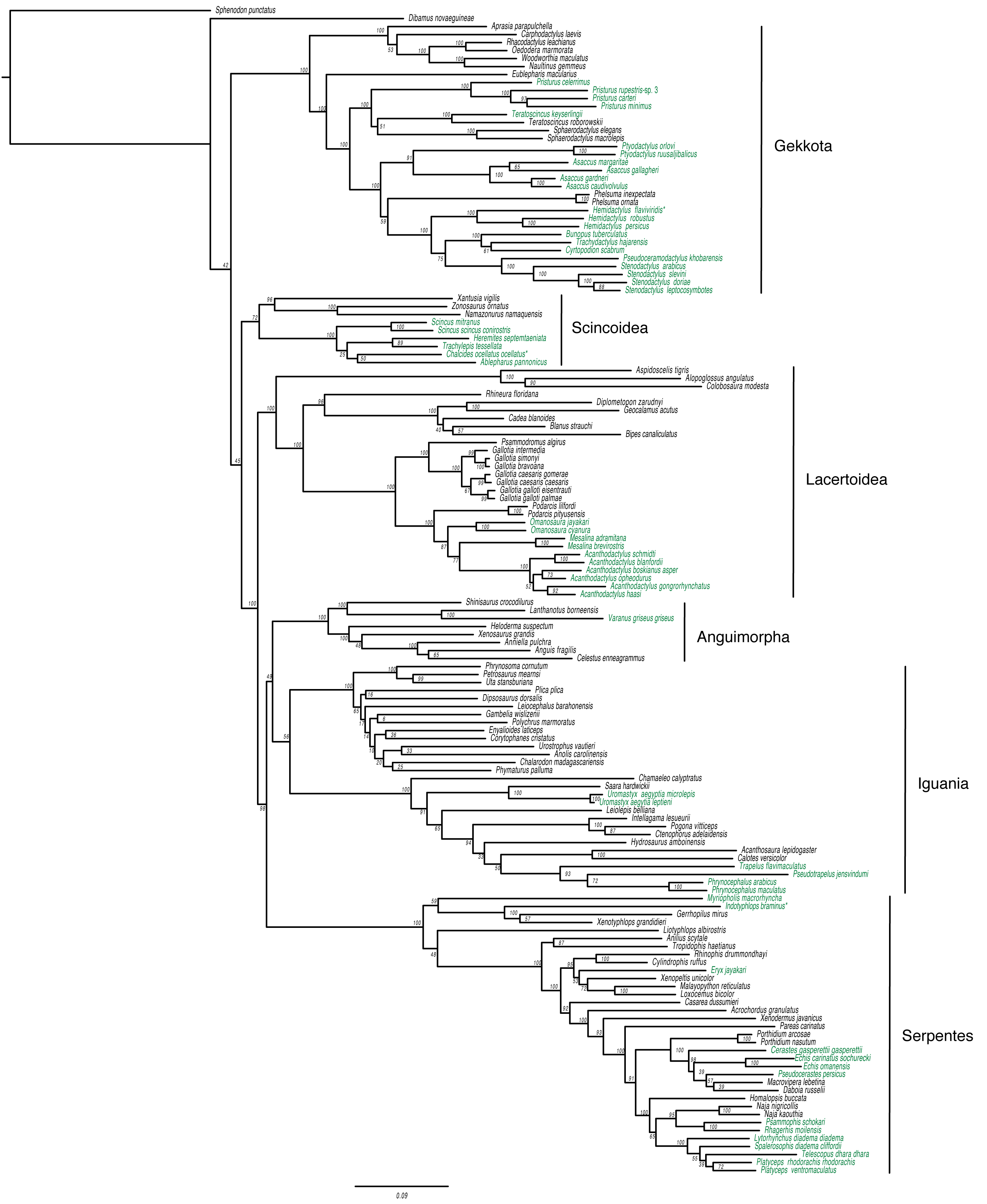

Supplement: S6 Fig — Phylogenetic tree based on the concatenated dataset of 15 genes and 146 species of Squamata including all 60 UAE terrestrial reptiles (highlighted in green) and one outgroup. Asterisks highlight the three introduced species. (TIF) [file pone.0216273.s009.tif]

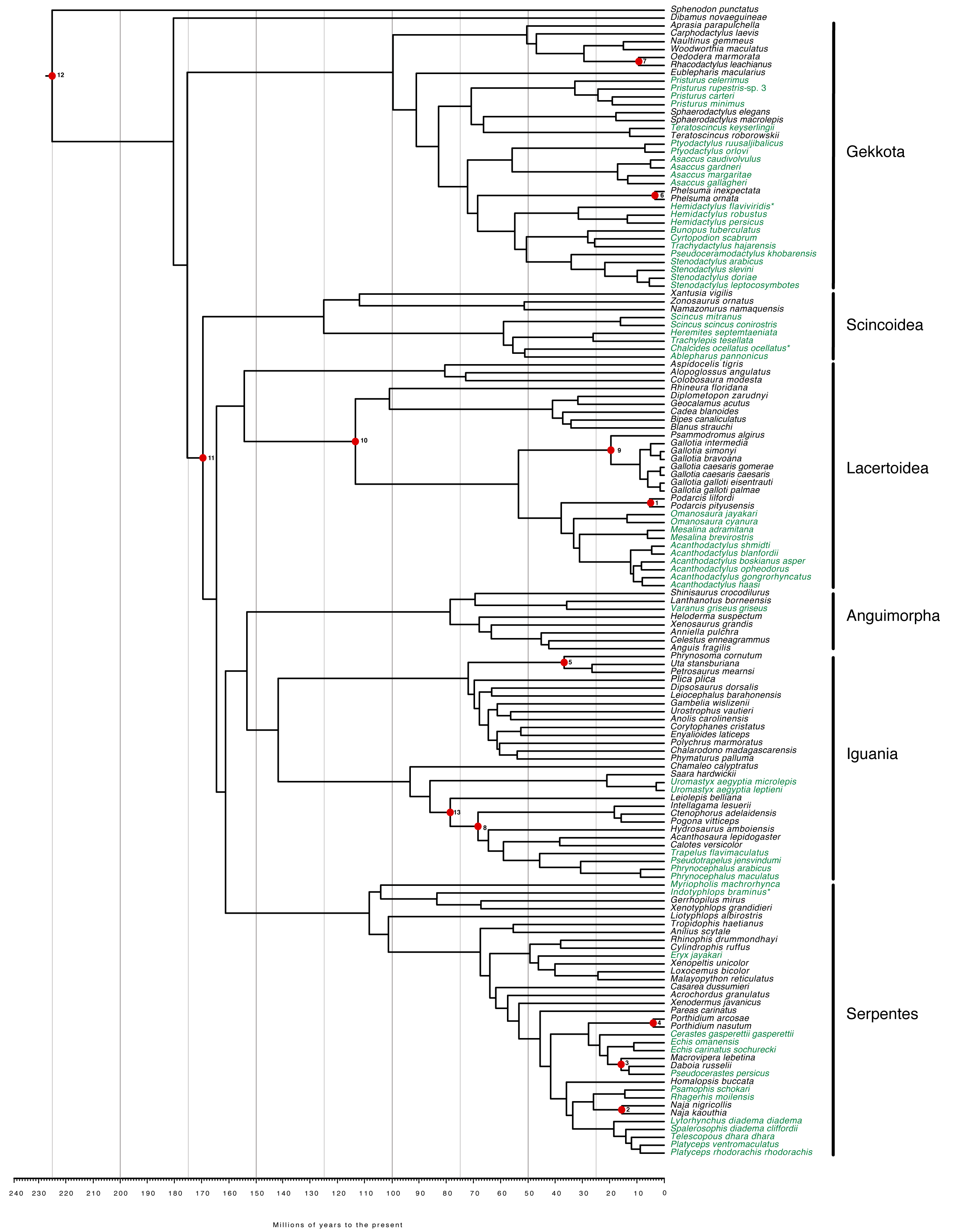

Supplement: S7 Fig — Time calibrated tree based on the concatenated dataset of 15 genes and 146 species of Squamata including all 60 UAE terrestrial reptiles (highlighted in green) and one outgroup inferred with BEAST. The 13 calibration points are indicated as red circles and are labelled as in S2 Appendix which gives further details. The topology of the time calibrated tree was fixed to the ML tree shown in S6 Fig The scale below the tree is in millions of years. Asterisks highlight the three introduced species. (TIF) [file pone.0216273.s010.tif]

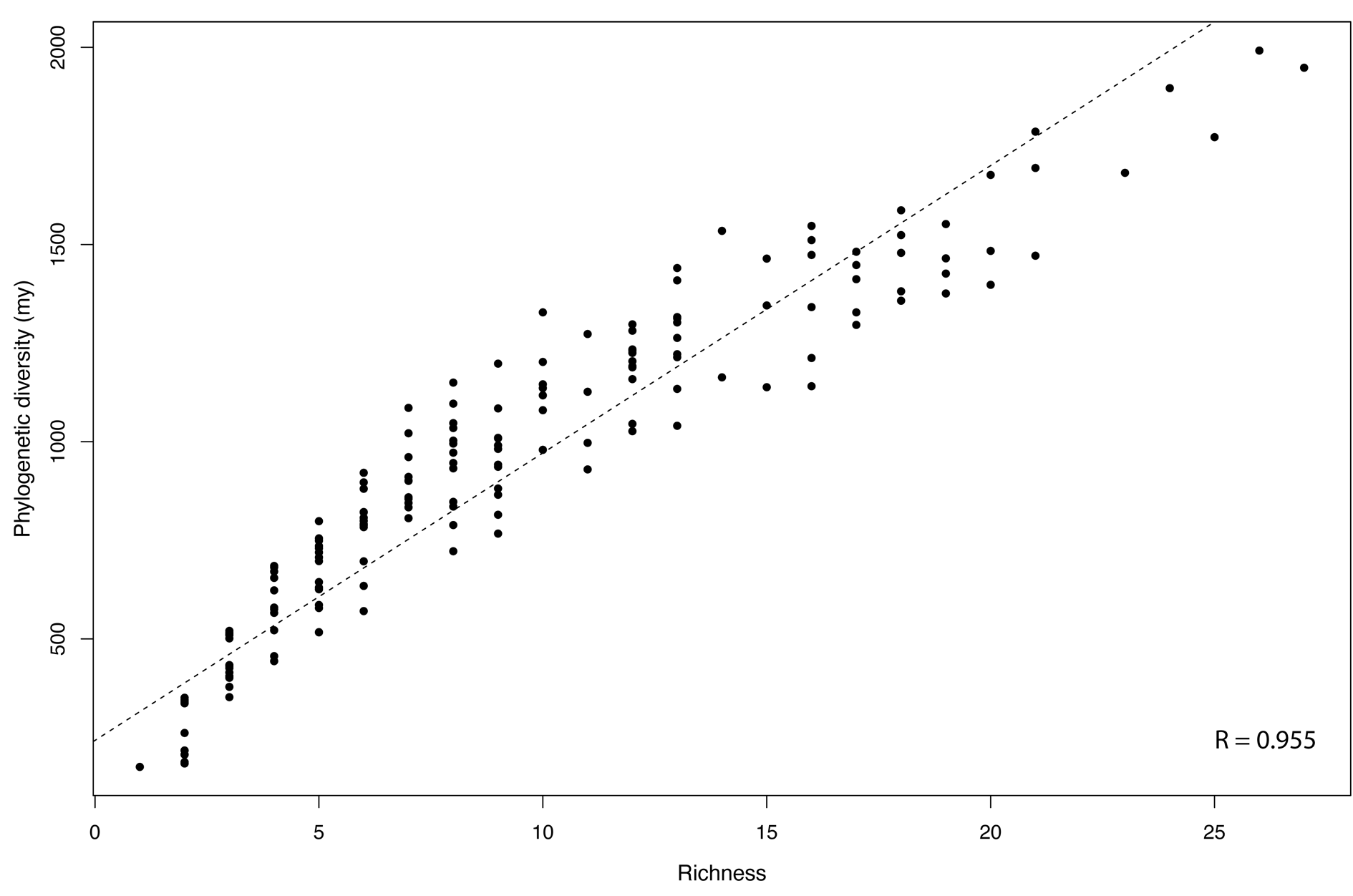

Supplement: S8 Fig — Points in the graph are cells of the 10 arc-min grid. The regression line was fitted using a linear model. (TIF) [file pone.0216273.s011.tif]
