# Supplementary material for: An integrative assessment of the diversity, phylogeny, distribution, and conservation of the terrestrial reptiles (Sauropsida, Squamata) of the United Arab Emirates
Source: PLoS One. 2019 May 2;14(5):e0216273. doi: 10.1371/journal.pone.0216273 (PMC6497385; doi:10.1371/journal.pone.0216273)
Supplement: S1 Table — For the UAE species the table includes species name, the country and specimen code. (PDF) [file pone.0216273.s012.pdf]

S1 Table. List of all the 146 species of reptiles included in the phylogenetic analyses. The table includes the species name, the species family and the country and specimen code for the reptiles of the UAE. Newly produced sequences for this study are in bold.

SPECIES	FAMILY	COUNTRY	CODE	12S	16S	BDNF	CMOS	Cytb	ND2	ND4	NT3	PDC	R35	RAG1	RAG2	MC1R	ACM4	COI
Ablepharus pannonicus	Scincidae	UAE		-	KX591478	-	AF039466	KX591441	AY607280	-	-	-	-	-	-	KX591401	-	-
Acanthodactylus blanfordii	Lacertidae	UAE	A291 a b	KX296905	-	-	KX297574	KX297016	-	-	-	-	-	-	-	KX297304	KX297379	-
Acanthodactylus boskianus asper	Lacertidae	UAE		GU225705	GU433291	-	EF632251	HM778107	-	HM778100	-	-	-	EF632206	-	KJ548089	KJ547914	-
Acanthodactylus gongrorhynchatus	Lacertidae	UAE	A128 a b	KX296909	-	-	KX297586	KX297125	-	-	-	-	-	-	-	KX297310	KX297459	-
Acanthodactylus haasi	Lacertidae	UAE	A302 a b	KX296904	-	-	KX297584	KX297030	-	-	-	-	-	-	-	KX297283	KX297405	-
Acanthodactylus opheodurus	Lacertidae	UAE	A209 a	KX296892	-	-	KX297559	KX296998	-	-	-	-	-	-	-	KX297277	KX297386	-
Acanthodactylus schmidti	Lacertidae	UAE	A228 a b	KX296910	-	-	KX297576	KX297019	-	-	-	-	-	-	-	KX297307	KX297383	-
Acanthosaura lepidogaster	Agamidae			KR092427	KR092427	JF806003	-	KR092427	KR092427	KR092427	JF804531	-	JF804578	JF806187	-	-	-	KR092427
Acrochordus granulatus	Acrochordidae			AB177879	AB177879	FJ433981	AF471124	AB177879	AB177879	AB177879	EU390905	-	EF144065	HM234060	EF144093	-	-	AB177879
Alopoglossus angulatus	Alopoglossidae			-	-	HQ876226	AF420847	KT254394	-	AF420909	JN568329	-	HQ876345	HQ876442	-	-	-	-
Anguis fragilis	Anguidae			EU443256	EU443256	JQ845041	AY099972	AY099996	AF085622	NC_012431	JQ844948	-	-	KF736847	-	-	-	KP697909
Anilius scytale	Aniliidae			AF512729	FJ755180	EU402625	AF544722	U69738	FJ755180	FJ755180	FJ434066	-	HQ876355	AY988072	FJ433891	-	-	-
Anniella pulchra	Anniellidae			-	-	EU445901	AY487350	AF195090	AF407537	AY620747	GU456008	-	HM161053	AY662605	DQ119636	-	-	KU985941
Anolis carolinensis	Dactyolidae			NC_010972	AB031978	EU402616	-	NC_010972	AB473620	EU747728	EU390900	-	HQ876334	FJ356739	-	-	-	KY800460
Aprasia parapulchella	Pygopodidae			NC_024557	NC_024557	-	AY134539	NC_024557	NC_024557	NC_024557	-	GU459741	-	HQ426260	HQ426433	-	HQ426339	NC_024557
Asaccus caudivolvulus	Phyllodactylidae	UAE	S7445	KX550491	-	-	KX550758	KX550584	HM212519	-	-	-	-	-	-	KX550844	KX550671	-
Asaccus gallagheri	Phyllodactylidae	UAE	CN2606	MG019457	-	-	MG019532	MG019662	-	-	-	-	-	-	-	MG019609	KX550794	-
Asaccus gardneri	Phyllodactylidae	UAE	CN3686	KX550448	-	-	KX550718	KX550544	-	-	-	-	-	-	-	KX550805	KX550632	-
Asaccus margaritae	Phyllodactylidae	UAE	CN742	KX550506	-	-	KX550773	KX550599	-	-	-	-	-	-	-	KX550859	KX550686	-
Aspidoscelis tigris	Teiidae			AF206585	AY046489	EU402619	AF039481	AF006288	U71332	AF026173	EU390903	HQ426250	HM161054	AY662620	HQ426512	-	HQ426423	-
Bipes canaliculatus	Bipedidae			EU203658	EU203658	FJ441849	FJ518700	AY605484	NC_006288	NC_006288	GU456033	-	DQ119614	FJ518701	-	-	-	-
Blanus strauchi	Blanidae			EU203660	FJ518702	FJ441847	AY444024	-	FJ518703	EF036463	EU203628	-	EU203630	AY444050	EU203634	-	-	-
Bunopus tuberculatus	Gekkonidae	UAE		EU589160	-	HQ443613	AF148706	EU589181	HQ443541	-	-	JQ945355	-	JQ945287	JQ945427	-	JQ945641	-
Cadea blanoides	Cadeidae			EU203661	EU203661	EU203612	EU203613	-	-	-	EU203629	-	EU203631	EU203662	EU203635	-	-	-
Calotes versicolor	Agamidae			NC_009683	NC_009683	DQ340705	AF137525	NC_009683	NC_009683	NC_009683	JX839246	-	JX839165	JN979993	-	-	-	NC_009683
Carphodactylus laevis	Carphodactylidae			AF090175	GU460142	-	EF534905	AF109565	AY369017	-	-	GU459744	-	EF534781	EF534947	-	EF534862	-
Casarea dussumieri	Bolyeriidae			AF544754	AF544827	EU402632	AF471114	U69755	-	-	EU390912	-	FJ433918	EU402840	FJ433894	-	-	-
Celestus enneagrammus	Diploglossidae			-	-	GU457853	-	-	AF085607	-	GU456009	-	JN703087	AY662604	-	-	-	-
Cerastes gasperettii gasperettii	Viperidae	UAE	Ceragasp01	**MK751370**	-	-	**MK751372**	**MK751373**	-	-	-	-	-	-	-	**MK751379**	-	-
Chalarodon madagascariensis	Opluridae			AB266748	EU099714	AY987972	AY987987	AB266748	AF528722	AB266748	JF804536	-	JF804582	FJ356745	-	-	-	JQ909351
Chalcides ocellatus ocellatus	Scincidae	UAE		JQ344273	AY649147	HM160584	AY818798	FJ972228	AY662557	JQ344297	-	-	HM161058	AY662638	-	-	-	-
Chamaeleo calyptratus	Chamaeleonidae			EF222192	EF222192	GU457847	HF570667	EF222192	EF222192	EF222192	GU456003	-	HQ876323	HF570766	-	-	-	EF222192
Colobosaura modesta	Gymnophthalmidae			AF420666	AF420733	EU108353	EU116677	EU116506	-	AF420887	JN568324	-	JN568502	EU108527	-	-	-	-
Corytophanes cristatus	Corytophanidae			-	-	JF806020	AF315390	-	AF528717	-	JF804541	-	JF804585	JF806205	-	-	-	-
Ctenophorus adelaidensis	Agamidae			-	-	DQ340740	DQ340692	-	FJ200009	-	JF804566	-	JF804602	JF806192	-	-	-	-
Cylindrophis ruffus	Cylindrophiidae			NC_007401	NC_007401	AY988037	AF471133	NC_007401	NC_007401	NC_007401	EU390915	-	JF804588	AY662613	-	-	-	NC_007401
Cyrtopodion scabrum	Gekkonidae	UAE		KC735098	HM040943	-	HQ426532	-	JX041345	-	-	HQ426186	-	HQ426275	HQ426448	-	HQ426355	AY677730
Daboia russelii	Viperidae			GQ225676	GQ398147	EU402636	AF471156	AF471076	NC_011391	DQ305477	EU390916	-	HQ876367	EU402843	-	-	-	KF698972
Dibamus novaeguineae	Dibamidae			-	KC621330	EU108355	EF450999	EU116508	FJ195390	FJ195390	EU108021	-	-	EU108529	-	-	-	-
Diplometopon zarudnyi	Trogonophidae	UAE		-	KJ624788	JN654798	AY444023	NC_006283	NC_006283	NC_006283	JN568326	-	JN568505	JN654858	-	-	-	-
Dipsosaurus dorsalis	Iguanidae			-	KC621331	GQ853275	AF148705	EU116509	AF049857	U66239	JF804545	-	HQ876329	FJ356747	-	KR026586	-	KU986065
Echis carinatus sochureki	Viperidae	UAE		GQ359605	GQ359690	-	-	GQ359441	-	EU852301	-	-	-	EU852325	-	-	-	EU852307
Echis omanensis	Viperidae	UAE		GQ359637	EU642581	-	-	EU642590	-	GQ359558	-	-	-	-	-	-	-	-
Enyalioides laticeps	Hoplocercidae			-	-	EU586761	-	-	EU586748	U66226	GU456004	-	JF804590	AY662593	-	-	-	-
Eryx jayakari	Boidae	UAE		HQ658419	-	DQ465566	DQ465565	-	-	-	-	-	-	DQ465567	-	-	-	-
Eublepharis macularius	Eublepharidae			NC_033383	NC_033383	GU457864	EU366458	NC_033383	NC_033383	NC_033383	GU456020	EF534816	HQ876372	EF534776	EF534942	-	EF534857	NC_033383
Gallotia bravoana	Lacertidae			AJ272395	-	-	AF435103	AJ272396	-	-	-	-	-	-	-	-	-	-
Gallotia caesaris caesaris	Lacertidae			AY151922	DQ298684	-	-	AY151843	-	-	-	-	-	-	-	-	-	KF003311
Gallotia caesaris gomerae	Lacertidae			AY151921	-	-	-	AY151842	-	-	-	-	-	-	-	-	-	KF003310
Gallotia galloti eisentrauti	Lacertidae			AY151918	DQ298685	-	AY152002	AY151839	-	-	-	-	-	-	-	-	-	KF003309
Gallotia galloti palmae	Lacertidae			AY151920	DQ298687	-	AY152004	AY151841	-	-	-	-	-	-	-	-	-	KF003308
Gallotia intermedia	Lacertidae			AY151923	DQ298681	-	AY152007	AY151844	-	-	-	-	-	-	-	-	-	-
Gallotia simonyi	Lacertidae			AY151924	DQ298682	-	AY152008	AF101219	-	-	-	-	-	-	-	-	-	-
Gambelia wislizenii	Crotaphytidae			NC_012831	AY217944	EU108358	EU116682	EU116511	NC_012831	NC_012831	JF804548	-	HQ876327	AY662600	-	-	-	KU985618
Geocalamus acutus	Amphisbaenidae			AB162909	FJ441724	FJ441907	FJ441784	NC_006285	FJ441967	NC_006285	JN568319	-	JN568486	JN654860	-	-	-	-

SPECIES	FAMILY	COUNTRY	CODE	12S	16S	BDNF	CMOS	Cytb	ND2	ND4	NT3	PDC	R35	RAG1	RAG2	MC1R	ACM4	COI
Gerrhopilus mirus	Gerrhopilidae			AM236345	AM236345	GU902394	-	KT316555	-	-	GU902566	-	-	GU902644	-	-	-	AM236345
Heloderma suspectum	Helodermatidae			NC_008776	NC_008776	FJ433955	AY662566	NC_008776	AF085603	NC_008776	GU456012	HQ426254	DQ119617	AY662606	DQ119635	-	HQ426427	KJ917183
Hemidactylus flaviviridis	Gekkonidae	UAE	NO ARID	KC818676	HM040937	-	JQ957126	KC818822	EU268356	EU268419	-	EU268325	-	HM559694	KC819026	JQ957253	HQ426366	KU567366
Hemidactylus persicus	Gekkonidae	UAE	Arid Clade	KC818691	-	-	KC818775	KC818842	-	-	-	-	-	KC818983	KC819044	KC818924	-	-
Hemidactylus robustus	Gekkonidae	UAE	Arid Clade	KC818698	-	-	KP238254	KC818849	EU054287	JQ957394	-	EU268345	-	KP238238	-	KP238252	HQ426374	KU567479
Heremites septemtaeniatus	Scincidae	UAE		-	KX364964	KX364972	KX364996	MF590363	-	-	-	-	-	KX365050	-	KX365022	-	-
Homalopsis buccata	Homalopsidae			EF395892	EF395868	EU402642	EF395940	EF395917	-	-	EU390922	-	EF144070	-	EF144097	-	-	LC075330
Hydrosaurus amboinensis	Agamidae			AB475096	NC_014178	-	-	NC_014178	NC_014178	NC_014178	-	-	-	-	-	-	-	NC_014178
Indotyphlops braminus	Typhlopidae	UAE		DQ343649	DQ343649	FJ433959	AY099980	DQ343649	AY662539	NC_010196	FJ434065	HQ426256	FJ433913	AY662612	FJ433889	-	-	DQ343649
Intellagama lesueurii	Agamidae			AB031974	AB031991	DQ340737	DQ340689	-	AF128463	-	JF804562	-	JF804599	AY662581	-	-	-	-
Lanthanotus borneensis	Lanthanotidae			-	-	GU457859	AY662564	-	AY662537	-	GU456015	-	-	AY662609	-	-	-	-
Leiocephalus barahonensis	Leiocephalidae			U39564	KU710307	HQ876223	DQ119594	KU710319	EF591774	-	JF804551	-	DQ119622	HQ876439	DQ119640	-	-	-
Leiolepis belliana	Agamidae			AB537554	AB537554	AY987965	FJ984253	-	AB537554	AB537554	JF804552	-	HQ876324	AY662587	-	-	-	AB537554
Liotyphlops albirostris	Anomalepididae			Z46461	AF366762	FJ433960	AF544727	AF544672	-	-	-	-	HQ876356	EU402853	FJ433890	-	-	-
Loxocemus bicolor	Loxocemidae			AF512737	AF512737	FJ433967	AY099969	AY099993	-	-	FJ434072	-	FJ433921	DQ465574	FJ433897	-	-	-
Lytorhynchus diadema diadema	Colubridae	UAE		AY647229	AY188064	-	AY187986	AY188025	-	-	-	-	-	-	-	-	-	-
Macrovipera lebetina	Viperidae			KX694561	KX694652	-	KX694785	AJ275713	-	DQ897729	KX695028	-	-	KX695096	-	-	-	KJ950727
Malayopython reticulatus	Pythonidae			Z46448	EF545062	FJ433969	AF544675	U69860	-	-	FJ434074	-	FJ433923	EU624119	FJ433899	-	-	KX012784
Mesalina adramitana	Lacertidae	UAE	CN1	**MK533819**	**MK533891**	-	**MK551589**	**MK551691**	-	-	-	-	-	**MK551675**	-	**MK551616**	**MK551568**	-
Mesalina brevirostris	Lacertidae	UAE	SPM001455U	KY967187	KY967128	-	KY967087	KY967153	-	-	-	-	-	**MK551679**	-	KY967109	**MK551570**	-
Myriopholis macrorhyncha	Leptotyphlopidae	UAE		GQ469245	GQ469245	GQ469187	GQ469072	GQ469115	-	-	GQ469026	-	-	GQ469049	-	-	-	-
Naja kaouthia	Elapidae		5	EU624235	JF357948	EU402654	AY058938	AF217835	-	EU624209	EU390930	-	JN703083	EU402857	-	-	-	AB920184
Naja nigricollis	Elapidae			EU624237	GQ359754	-	-	AF399746	-	DQ897697	-	-	-	-	-	-	-	KX012716
Namazonurus namaquensis	Platysaurinae			HQ167104	HQ167215	AY987981	AY217848	EU116507	-	KC621496	EU108020	-	-	EU108528	-	-	-	-
Naultinus gemmeus	Diplodactylidae		5	-	GU459962	-	JQ945592	-	GU459764	-	-	GU459563	-	GU459361	JQ945486	-	JQ945699	-
Oedodera marmorata	Diplodactylidae		3	-	GU460146	-	JQ945594	-	GU459947	JQ398455	-	GU459748	-	GU459546	JQ945488	-	JQ945701	-
Omanosaura cyanura	Lacertidae	UAE		CN761	-	-	CN761	CN761	-	CN761	-	-	-	-	-	CN761	-	-
Omanosaura jayakari	Lacertidae	UAE		AO32	AF080352	-	AO32	AO32	-	AO32	-	-	-	-	-	AO32	-	-
Pareas carinatus	Pareidae			AF544773	AF544802	FJ433985	AF544692	JQ598940	-	JF827653	FJ434086	-	EF144069	-	EF144096	-	-	-
Petrosaurus mearnsi	Phrynosomatidae			L40444	L41450	HQ876221	-	EF653316	JN648436	AF210354	JF804557	-	HQ876333	-	-	-	-	KU985980
Phelsuma inexpectata	Gekkonidae			AY221291	FJ829917	JQ073109	FJ830087	FJ830003	JN393939	-	-	JN394016	-	-	FJ830270	-	-	-
Phelsuma ornata	Gekkonidae		Code	AY221323	FJ829948	JQ073110	FJ830118	FJ830033	EU423282	-	-	-	-	-	FJ830301	-	-	-
Phrynocephalus arabicus	Agamidae	UAE	ZMMU R 12713	-	-	-	-	-	KF691651	KF691675	-	-	-	KJ363507	-	-	-	KF691708
Phrynocephalus maculatus	Agamidae	UAE	ZMMU R 12501	-	-	-	-	KF691623	KF691650	KF691674	-	-	-	KJ363506	-	-	-	KF691707
Phrynosoma cornutum	Phrynosomatidae			DQ385390	L41453	AY987975	AY987989	AY141087	DQ385344	AY141049	KR360082	-	KJ124005	FJ356738	-	-	-	KU986066
Phymaturus palluma	Liolaemidae			KT203839	-	JF806024	JX969520	KT203834	AF099216	-	JF804560	-	JF804598	JF806209	-	-	-	-
Platyceps rhodorachis rhodorachis	Colubridae	UAE	CAS 185035	AY039154	MG700224	-	AY486945	AY486921	AY487012	AY487051	-	-	-	-	-	-	-	MG700035
Platyceps ventromaculatus	Colubridae	UAE	MHNG2443.10	AY039136	-	-	-	-	-	-	-	-	-	-	-	-	-	AY039174
Plica plica	Tropiduridae			AB218961	AB218961	JF806028	EF615737	AB218961	AF528748	AB218961	JF804573	-	JF804607	FJ356742	-	-	-	KU245104
Podarcis lilfordi	Lacertidae		3	AF133447	KX658188	-	EF679323	AF052639	EU006763	-	-	-	-	-	-	JX126668	-	KF003303
Podarcis pityusensis	Lacertidae			EF694768	KX658227	-	EF679328	AF052640	EU006764	-	-	-	-	-	-	JX126689	-	KF003305
Pogona vitticeps	Agamidae			AB166795	AB166795	DQ340739	DQ340691	NC_006922	AB166795	AB166795	JF804563	-	JF804600	JF806200	-	-	-	AB166795
Polychrus marmoratus	Polychrotidae			NC_012839	NC_012839	AY987966	AY987983	NC_012839	NC_012839	NC_012839	JF804564	-	HQ876335	FJ356748	-	-	-	NC_012839
Porthidium arcosae	Viperidae			EU624241	EU624275	-	-	AF292575	-	AF292613	-	-	-	-	-	-	-	-
Porthidium nasutum	Viperidae			AF057204	EU624277	KX694748	KX694790	DQ061210	-	U41887	KX694988	-	-	-	-	-	-	-
Pristurus carteri	Sphaerodactylidae	UAE	S1785	KJ849846	-	-	KJ849979	**MK751374**	-	-	-	-	-	KJ850039	KJ850114	**MK751380**	KJ849923	-
Pristurus celerrimus	Sphaerodactylidae	UAE	AO72	KJ849850	-	-	KJ849982	**MK751375**	-	-	-	-	-	KJ850043	KJ850118	**MK751381**	KJ849927	-
Pristurus minimus	Sphaerodactylidae	UAE	S7990	KJ849885	-	-	KJ850005	**MK751376**	-	-	-	-	-	KJ850075	KJ850148	**MK751382**	KJ849949	-
Pristurus rupestris-sp.3	Sphaerodactylidae	UAE	CN133	KY023386	-	-	KY023920	KY023829	-	-	-	-	-	KY024038	KY024111	KY023964	-	-
Psammodromus algirus	Lacertidae			AY151914	DQ298675	EU108364	AY151998	AY151835	DQ150391	DQ150380	EU108030	KX080854	-	EF632241	-	KX080785	KX080994	-
Psammophis schokari	Lamprophiidae	UAE		FJ404121	AY611852	-	AY611943	AY612034	-	FJ404324	-	-	-	-	FJ404396	-	-	-
Pseudoceramodactylus khobarensis	Gekkonidae	UAE		KC190702	KC190896	HQ443614	KC191005	-	-	-	-	-	-	-	KC191123	-	-	-
Pseudocerastes persicus	Viperidae	UAE	CN10306	-	AJ275770	-	-	J275717/KX34321	-	-	-	-	-	-	-	-	-	-
Pseudotrapelus jensvindumi	Agamidae	UAE	Pseujens01	**MK751371**	KU097482	-	KU097540	**MK751377**	-	KU169211	-	-	-	-	**MK751384**	KU097617	**MK782053**	-
Ptyodactylus orlovi	Phyllodactylidae	UAE	CN2960	MF084420	-	-	MF084480	MF084662	-	-	-	-	-	-	MF084601	MF084715	MF084541	-
Ptyodactylus ruusaljibalicus	Phyllodactylidae	UAE	CN8173	MF084457	-	-	MF084518	MF084692	-	-	-	-	-	-	MF084639	MF084752	MF084579	-
Rhacodactylus leachianus	Diplodactylidae		3	AF090176	GU460148	-	JQ945612	-	GU459949	-	-	GU459750	-	GU459548	JQ945505	-	JQ945718	-
Rhagerhis moilensis	Lamprophiidae	UAE	E1110.16/HLMD	AY643313	AY643355	-	DQ486157	AY643397	-	DQ486309	-	-	-	-	-	-	-	-

SPECIES	FAMILY	COUNTRY	CODE	12S	16S	BDNF	CMOS	Cytb	ND2	ND4	NT3	PDC	R35	RAG1	RAG2	MC1R	ACM4	COI
Rhineura floridana	Rhineuridae			KU744569	EU203657	FJ441848	AY444022	-	-	KU744593	GU456034	EU293714	DQ119613	AY444048	DQ119631	-	EF534899	-
Rhinophis drummondhayi	Uropeltidae			AY700994	AY701029	FJ433966	AF544719	AF544673	-	-	FJ434071	-	FJ433920	-	FJ433896	-	-	-
Saara hardwickii	Agamidae			HM040904	FJ639591	GU457849	-	AB474757	AB113803	-	GU456005	-	-	GU457972	-	-	-	-
Scincus mitranus	Scincidae	UAE		EU278015	EU278080	-	-	EU278247	-	-	-	-	-	-	-	-	-	-
Scincus scincus conirostris	Scincidae	UAE		AY218025	AY308304	HM160669	AY217873	AY217822	AY607278	-	GU456028	-	HM161142	HM161238	-	-	-	-
Shinisaurus crocodilurus	Shinisauridae			NC_005959	AB080274	GU457857	AY099976	NC_005959	NC_005959	NC_005959	GU456013	-	HQ876341	AY662610	-	-	-	-
Spalerosophis diadema cliffordii	Colubridae	UAE		AY039144	HQ658450	-	AF471155	AF471049	AY487020	AY487059	KX695051	-	-	KX695115	-	-	-	AY039186
Sphaerodactylus elegans	Sphaerodactylidae			KF017637	X86048	-	EF534912	KF017628	JN393942	-	-	EF534828	-	EF534787	EF534954	-	EF534869	-
Sphaerodactylus macrolepis	Sphaerodactylidae			KC840470	X86047	-	HQ426580	-	KP640637	-	-	HQ426238	-	HQ426326	HQ426499	-	HQ426409	-
Sphenodon punctatus	Sphenodontidae		3	AF534390	AF534390	GU457846	AF039483	AF534390	AF534390	AF534390	-	HQ426257	HQ876320	AY662576	HQ426516	-	AY168473	AF534390
Stenodactylus arabicus	Gekkonidae	UAE	E150536	KC190693	KC190887	-	KC190996	-	-	-	-	-	-	-	KC191115	KF898478	-	-
Stenodactylus doriae	Gekkonidae	UAE	E150538	KC190653	KC190847	-	KC190939	-	-	-	-	-	-	-	KC191050	-	-	-
Stenodactylus leptocosymbotus	Gekkonidae	UAE		KC190665	KC190860	HQ443633	KC190943	-	HQ443536	-	-	-	-	-	KC191052	-	-	-
Stenodactylus slevini	Gekkonidae	UAE	E1505334	KC190687	KC190881	-	KC190993	-	-	-	-	-	-	-	KC191060	-	-	-
Telescopus dhara dhara	Colubridae	UAE		HQ658443	HQ267786	-	-	-	-	-	-	-	-	-	-	-	-	-
Teratoscincus keyserlingii	Sphaerodactylidae	UAE		AY753545	AY753545	-	HQ426589	AY753545	AY753545	AY753545	-	-	-	-	HQ426508	-	HQ426418	AY753545
Teratoscincus roborowskii	Sphaerodactylidae			KP115216	KP115216	-	EF534925	KP115216	KP115216	KP115216	-	EF534841	-	EF534799	EF534967	-	EF534882	MF573804
Trachydactylus hajarensis	Gekkonidae	UAE	CN2575	KT302073	-	-	KT302105	-	-	-	-	KT302133	-	KT302135	KT302141	-	KT302096	-
Trachylepis tessellata	Scincidae	UAE		JQ598776	JQ598788	-	-	-	-	-	-	-	-	-	-	-	-	-
Trapelus flavimaculatus	Agamidae	UAE	Trapflav01	-	-	-	-	**MK751378**	AB201529	-	-	-	-	-	-	-	-	-
Tropidophis haetianus	Tropidophiidae			NC_012573	NC_012573	AY988039	AY099962	NC_012573	NC_012573	NC_012573	EU390938	-	JN703089	AY988073	-	-	-	NC_012573
Uromastyx aegyptia leptieni	Agamidae	UAE	ZFMK52398	FJ639660	FJ639623	-	-	MF960595	-	-	-	-	-	-	-	-	-	-
Uromastyx aegyptia microlepis	Agamidae	UAE	SPM001689	FJ639657	MF980196	-	-	MF960570	-	MF993191	MF960471	-	-	-	-	MF960369	MF960260	-
Urostrophus vautieri	Leiosauridae			-	KM517592	HQ876219	KT342957	KT342907	AF528734	KM517830	JF804576	-	HQ876330	HQ876435	-	-	-	-
Uta stansburiana	Phrynosomatidae			DQ385408	L41489	DQ385334	AF315389	AY141102	AF049863	AY141066	JF804577	-	JF804610	-	-	JX481733	-	KU986271
Varanus griseus griseus	Varanidae	UAE		KU746369	KU746383	-	-	KU867856	U71334	-	-	-	-	AY662608	-	-	-	-
Woodworthia maculatus	Diplodactylidae		5	-	HM542437	-	JQ945628	-	GU459852	JQ398454	-	GU459651	-	GU459449	JQ945522	-	JQ945735	-
Xantusia vigilis	Xantusiidae			AY218042	DQ249035	EU108491	EU116835	EU116656	EU130279	AY584459	EU390904	HQ426258	HM161146	AY662642	DQ119626	-	HQ426431	KU986173
Xenodermus javanicus	Xenodermidae			AF544781	AF544810	EU402667	AF544711	AY425810	-	U49320	-	-	JN703065	EU402869	-	-	-	-
Xenopeltis unicolor	Xenopeltidae			AF512735	AF512735	FJ433968	AF544689	LC105622	-	-	FJ434073	-	FJ433922	EU402870	FJ433898	-	-	-
Xenosaurus grandis	Xenosauridae			-	-	GU457858	AY662567	-	U71333	-	GU456014	-	JN703069	AY662607	-	-	-	-
Xenotyphlops grandidieri	Xenotyphlopidae			KT316450	-	GU902457	-	KF770851	-	-	GU902627	-	-	JQ073250	-	-	-	JQ909623
Zonosaurus ornatus	Gerrhosauridae			AJ416929	AJ416930	JN654807	DQ100120	DQ004409	-	-	JN568317	-	JN568497	JN614040	KC515332	-	-	JQ909633
