# Supplementary material for: An integrative assessment of the diversity, phylogeny, distribution, and conservation of the terrestrial reptiles (Sauropsida, Squamata) of the United Arab Emirates
Source: PLoS One. 2019 May 2;14(5):e0216273. doi: 10.1371/journal.pone.0216273 (PMC6497385; doi:10.1371/journal.pone.0216273)
Supplement: S2 Table — Gene composition, fragment lengths, partitions, models and run specifications for the different phylogenetic analyses are shown. (PDF) [file pone.0216273.s013.pdf]

S2 Table. Information on the dataset used for the phylogenetic analyses. Composition, length, partitions, models and run- specifications for the dataset used for phylogenetic analyses.

Dataset	Genes	Nº seq	bp	Subst. model (unlinked) & partitions	Clock model (unlinked)	Tree model (linked)	Alignments	Other prior parameters	Run specifications
146 taxa<br>14,454 bp	12S	125	329	GTR + G	Uncorrelated relaxed<br>Lognormal	Yule process<br>User-specified tree: RaxML tree	**Geneious**<br>Muscle alignment<br><br>**Gblocks**<br>Low stringent selection	**Partition Finder**<br>Branch length: linked<br>Models of evolution: BEAST<br>Model selection: BIC<br>Greedy search algorithm<br><br>**RaxML**<br>Analysis: ML + thorough bootstrap<br><br>**BEAST**<br>Alpha Uniform (0,10)<br>Base substitution Uniform (0,100)<br><br>**Calibration points:** Appendix S2	**RaxML**<br>Generations: 100<br>Reps: 1,000<br><br>**BEAST**<br>Runs: 3<br>Generations: $1x10^8$<br>Sampling frequency: 10,000<br>Burnin: 10%
	16S	108	442						
	COI	52	1.605	GTR + G			**TranslatorX**<br>Muscle protein alignment		
	CYTB	118	1.167	GTR + G					
	ND2	75	1.056	GTR + G					
	ND4	69	1.383	GTR + G					
	CMOS	120	945	GTR + G	Strict clock				
	RAG2	52	720						
	RAG1	91	2.934						
	ACM4	41	444						
	NT3	66	669						
	PDC	23	441						
	BDNF	74	714	GTR + G					
	MC1R	34	861	GTR + G					
	R35	57	744	GTR + G					
