# Supplementary material for: An integrative assessment of the diversity, phylogeny, distribution, and conservation of the terrestrial reptiles (Sauropsida, Squamata) of the United Arab Emirates
Source: PLoS One. 2019 May 2;14(5):e0216273. doi: 10.1371/journal.pone.0216273 (PMC6497385; doi:10.1371/journal.pone.0216273)
Supplement: S3 Table — Table containing all the species recorded in this study grouped by families and higher taxa. The table contains information regarding the regional IUCN conservation category (yet unpublished) for all the species and indicates if they are endemic to the UAE and if it is a medically important venomous species. Asterisks highlight the three introduced species. (PDF) [file pone.0216273.s014.pdf]

S3 Table. Checklist of the 60 species of UAE terrestrial reptiles. Table containing all the species recorded in this study and separated by families and by higher taxa. The table contains information regarding the global and regional IUCN conservation category for all the species and indicates if they are endemic and if it is a medically important venomous species. Asterisks highlight the three introduced species.

Taxa		Family/Species	Global IUCN	Regional IUCN	Endemic	Venomous
Lizards (46)	Agamids (6)					
		Agamidae (6)				
		Phrynocephalus arabicus	LC	LC	NO	NO
		Phrynocephalus maculatus	LC	LC	NO	NO
		Pseudotrapelus jensvindumi	NE	LC	NO	NO
		Trapelus flavimaculatus	LC	LC	NO	NO
		Uromastyx aegyptia leptieni	VU	VU	NO	NO
		Uromastyx aegyptia microlepis	VU	VU	NO	NO
	Amphisbaenids(1)					
		Trogonophidae (1)				
		Diplometopon zarudnyi	LC	LC	NO	NO
	Geckos (22)					
		Gekkonidae (11)				
		Bunopus tuberculatus	LC	LC	NO	NO
		Cyrtopodion scabrum	LC	LC	NO	NO
		*Hemidactylus flaviviridis**	LC	NA	NO	NO
		Hemidactylus persicus	LC	VU	NO	NO
		Hemidactylus robustus	LC	LC	NO	NO
		Pseudoceramodactylus khobarensis	LC	LC	NO	NO
		Stenodactylus arabicus	LC	LC	NO	NO
		Stenodactylus doriae	LC	LC	NO	NO
		Stenodactylus leptocosymbotes	LC	LC	NO	NO
		Stenodactylus slevini	LC	LC	NO	NO
		Trachydactylus hajarensis	NE	LC	NO	NO
		Phyllodactylidae (6)				
		Asaccus caudivolvulus	CR	CR	YES	NO
		Asaccus gallagheri	LC	LC	NO	NO
		Asaccus gardneri	NE	LC	NO	NO
		Asaccus margaritae	NE	VU	NO	NO
		Ptyodactylus orlovi	NE	LC	NO	NO
		Ptyodactylus ruusaljibalicus	NE	LC	NO	NO
		Sphaerodactylidae (5)				
		Pristurus carteri	LC	CR	NO	NO
		Pristurus celerrimus	LC	LC	NO	NO
		Pristurus minimus	LC	LC	NO	NO
		Pristurus rupestris-sp.3	NE	LC	NO	NO
		Teratoscincus keyserlingii	LC	CR	NO	NO
	Lacertids (10)					
		Lacertidae (10)				
		Acanthodactylus blanfordii	LC	VU	NO	NO
		Acanthodactylus boskianus asper	LC	NT	NO	NO
		Acanthodactylus gongrorhynchatus	DD	LC	NO	NO
		Acanthodactylus haasi	LC	DD	NO	NO
		Acanthodactylus opheodurus	LC	DD	NO	NO
		Acanthodactylus schmidti	LC	LC	NO	NO
		Mesalina adramitana	LC	LC	NO	NO
		Mesalina brevirostris	LC	LC	NO	NO
		Omanosaura cyanura	LC	LC	NO	NO
		Omanosaura jayakari	LC	LC	NO	NO
	Skinks (6)					
		Scincidae (6)				
		Ablepharus pannonicus	LC	LC	NO	NO
		*Chalcides ocellatus ocellatus**	LC	NA	NO	NO
		Heremites septemtaeniatus	LC	DD	NO	NO
		Scincus mitranus	LC	LC	NO	NO
		Scincus scincus conirostris	LC	DD	NO	NO
		Trachylepis tessellata	LC	LC	NO	NO
	Varanids (1)					
		Varanidae (1)				
		Varanus griseus griseus	LC	LC	NO	NO
Snakes (14)		Boidae (1)				
		Eryx jayakari	LC	LC	NO	NO
		Colubridae (5)				
		Lytorhynchus diadema diadema	LC	LC	NO	NO
		Platyceps rhodorachis rhodorachis	LC	LC	NO	NO
		Platyceps ventromaculatus	LC	VU	NO	NO
		Spalerosophis diadema cliffordii	LC	LC	NO	NO
		Telescopus dhara dhara	LC	LC	NO	NO
		Lamprophiidae (2)				
		Psammophis schokari	LC	LC	NO	NO
		Rhagerhis moilensis	LC	LC	NO	NO
		Leptotyphlopidae (2)				
		Myriopholis macrorhyncha	LC	LC	NO	NO
		Typhlopidae (1)				
		*Indotyphlops braminus**	LC	NA	NO	NO
		Viperidae (4)				
		Cerastes gasperettii gasperettii	LC	LC	NO	YES
		Echis carinatus sochureki	LC	LC	NO	YES
		Echis omanensis	LC	LC	NO	YES
		Pseudocerastes persicus	LC	LC	NO	YES
