# Supplementary material for: An integrative assessment of the diversity, phylogeny, distribution, and conservation of the terrestrial reptiles (Sauropsida, Squamata) of the United Arab Emirates
Source: PLoS One. 2019 May 2;14(5):e0216273. doi: 10.1371/journal.pone.0216273 (PMC6497385; doi:10.1371/journal.pone.0216273)
Supplement: S4 Table — Number and percentage of the total number of species, threatened species and medically important venomous species based on presence data. (PDF) [file pone.0216273.s015.pdf]

S4 Table. Information on the 60 species of UAE terrestrial reptiles for the whole country and independently for each emirate. Number and percentage of the total number of species, threatened species and medically important venomous species based on presence data. The conservation status follows the yet unpublished regional IUCN red list

	Species		Threatened species		Venomous species	
**Emirates/ UAE**	60	100,0%	8	100,0%	4	100%
Abu Dhabi	43	71,6%	6	75,0%	3	75%
Ajman	18	30,0%	0	0,0%	2	50%
Dubai	36	60,0%	3	37,5%	3	75%
Fujairah	25	41,6%	2	25,0%	3	75%
Ras al-Khaymah	37	61,6%	1	12,5%	4	100%
Sharjah	44	73,3%	5	62,5%	4	100%
Umm al-Quwain	24	40,0%	1	12,5%	2	50%
