# Supplementary material for: An integrative assessment of the diversity, phylogeny, distribution, and conservation of the terrestrial reptiles (Sauropsida, Squamata) of the United Arab Emirates
Source: PLoS One. 2019 May 2;14(5):e0216273. doi: 10.1371/journal.pone.0216273 (PMC6497385; doi:10.1371/journal.pone.0216273)
Supplement: S5 Table — Number of occupied 4 km2 cells for each approach, area occupied by each species for each approach. Asterisks highlight the three introduced species. (PDF) [file pone.0216273.s016.pdf]

S5 Table. Area of Occupancy (AOO) and Species Potential Distribution (SPD) at 4 km2 of all 60 terrestrial reptiles of the UAE. Number of occupied cells of 4 km2 for each approach, area occupied by each species for each approach. Asterisks highlight the three introduced species.

SPECIES	AOO		SPD	
	Nº of grids (2x2 km)	Area km^2	Nº of grids (2x2 km)	Area km^2
Ablepharus pannonicus	3	12	16	64
Acanthodactylus blanfordii	2	8	2	8
Acanthodactylus boskianus asper	6	24	538	2,152
Acanthodactylus gongrorhynchatus	53	212	18,179	72,716
Acanthodactylus haasi	5	20	11,565	46,260
Acanthodactylus opheodurus	8	32	7,399	29,596
Acanthodactylus schmidti	356	1,424	18,475	73,900
Asaccus caudivolvulus	2	8	2	8
Asaccus gallagheri	42	168	1,303	5,212
Asaccus gardneri	7	28	289	1,156
Asaccus margaritae	1	4	1	4
Bunopus tuberculatus	263	1,052	19,444	77,776
Cerastes gasperettii gasperettii	128	512	16,321	65,284
*Chalcides ocellatus ocellatus**	20	80	11,504	46,016
Cyrtopodion scabrum	48	192	15,287	61,148
Diplometopon zarudnyi	71	284	11,638	46,552
Echis carinatus sochureki	77	308	12,606	50,424
Echis omanensis	67	268	1,407	5,628
Eryx jayakari	156	624	18,604	74,416
*Hemidactylus flaviviridis**	38	152	19,412	77,648
Hemidactylus persicus	4	16	258	1,032
Hemidactylus robustus	80	320	12,040	48,160
Heremites septemtaeniata	3	12	786	3,144
*Indotyphlops braminus**	4	16	337	1,348
Lytorhynchus diadema diadema	98	392	16,473	65,892
Mesalina adramitana	68	272	5,772	23,088
Mesalina brevirostris	68	272	5,929	23,716
Myriopholis macrorhyncha	17	68	7,057	28,228
Omanosaura cyanura	25	100	1,459	5,836
Omanosaura jayakari	31	124	1,253	5,012
Phrynocephalus arabicus	260	1,040	17,753	71,012
Phrynocephalus maculatus	40	160	14,466	57,864
Platyceps rhodorachis rhodorachis	43	172	1,323	5,292
Platyceps ventromaculatus	8	32	29	116
Pristurus carteri	2	8	8	32
Pristurus celerrimus	54	216	1,293	5,172
Pristurus minimus	25	100	3,316	13,264
Pristurus rupestris-sp. 3	128	512	1,433	5,732
Psammophis schokari	129	516	19,591	78,364
Pseudoceramodactylus khobarensis	33	132	12,844	51,376
Pseudocerastes persicus	21	84	815	3,260
Pseudotrapelus jensvindumi	59	236	1,627	6,508
Ptyodactylus orlovi	45	180	2,237	8,948
Ptyodactylus ruusaljibalicus	5	20	1,222	4,888
Rhagerhis moilensis	65	260	7,044	28,176
Scincus mitranus	246	984	18,010	72,040
Scincus scincus conirostris	10	40	5,178	20,712
Spalerosophis diadema cliffordii	13	52	1,512	6,048
Stenodactylus arabicus	211	844	16,196	64,784
Stenodactylus doriae	185	740	18,621	74,484
Stenodactylus leptocosymbotes	53	212	4,827	19,308
Stenodactylus slevini	45	180	11,234	44,936
Telescopus dhara dhara	7	28	1,507	6,028
Teratoscincus keyserlingii	54	216	2,363	9,452
Trachydactylus hajarensis	52	208	1,425	5,700
Trachylepis tessellata	14	56	1,359	5,436
Trapelus flavimaculatus	90	360	13,381	53,524
Uromastyx aegyptia leptieni	140	560	14,910	59,640
Uromastyx aegyptia microlepis	81	324	13,840	55,360
Varanus griseus griseus	118	472	18,285	73,140
