# Supplementary material for: An integrative assessment of the diversity, phylogeny, distribution, and conservation of the terrestrial reptiles (Sauropsida, Squamata) of the United Arab Emirates
Source: PLoS One. 2019 May 2;14(5):e0216273. doi: 10.1371/journal.pone.0216273 (PMC6497385; doi:10.1371/journal.pone.0216273)
Supplement: S6 Table — BIO1 = Annual Mean Temperature, BIO4 = Temperature Seasonality, BIO5 = Max Temperature of Warmest Month, BIO6 = Min Temperature of Coldest Month, BIO7 = Temperature Annual Range, BIO10 = Mean Temperature of Warmest Quarter, BIO11 = Mean Temperature of Coldest Quarter, BIO12 = Annual Precipitation, BIO13 = Precipitation of Wettest Month, BIO16 = Precipitation of Wettest Quarter, BIO18 = Precipitation of Warmest Quarter, BIO19 = Precipitation of Coldest Quarter. (PDF) [file pone.0216273.s017.pdf]

S6 Table. Loadings, eigenvalues, and variance explained by the two first components retained from the Principal Component Analysis (PCA) performed on the 19 bioclimatic variables used in this study. BIO1 = Annual Mean Temperature, BIO4 = Temperature Seasonality, BIO5 = Max Temperature of Warmest Month, BIO6 = Min Temperature of Coldest Month, BIO7 = Temperature Annual Range, BIO10 = Mean Temperature of Warmest Quarter, BIO11 = Mean Temperature of Coldest Quarter, BIO12 = Annual Precipitation, BIO13 = Precipitation of Wettest Month, BIO16 = Precipitation of Wettest Quarter, BIO18 = Precipitation of Warmest Quarter, BIO19 = Precipitation of Coldest Quarter

**Climatic Variables**	**PC1**	**PC2**
BIO1	0,27302	-0,07493
BIO2	0,24910	-0,26812
BIO3	0,15036	-0,22257
BIO4	0,25088	-0,22293
BIO5	0,27909	-0,19821
BIO6	-0,19897	0,29878
BIO7	0,26455	-0,25462
BIO8	0,21436	-0,24354
BIO9	0,21691	-0,04110
BIO10	0,28572	-0,13842
BIO11	0,14398	0,14678
BIO12	-0,24658	-0,25498
BIO13	-0,22789	-0,23086
BIO14	-0,21354	-0,27939
BIO15	0,14579	0,31607
BIO16	-0,23035	-0,23839
BIO17	-0,23325	-0,30298
BIO18	-0,22223	-0,24408
BIO19	-0,24206	-0,15392
STD	3,173	2,0892
Variance explained (%)	53	22,9
