# Supplementary material for: An integrative assessment of the diversity, phylogeny, distribution, and conservation of the terrestrial reptiles (Sauropsida, Squamata) of the United Arab Emirates
Source: PLoS One. 2019 May 2;14(5):e0216273. doi: 10.1371/journal.pone.0216273 (PMC6497385; doi:10.1371/journal.pone.0216273)
Supplement: S7 Table — The table contains: the name of each emirate, the area of each emirate in km2; the number of protected areas that are inside each emirate; the area in km2 that is protected inside each emirate and the percentage of protected areas by emirate. (PDF) [file pone.0216273.s018.pdf]

S7 Table. Information on the protected areas of each emirate. The table contains: the name of each emirate, the area of each emirate in km2; the number of protected areas that are inside each emirate; the area in km2 that is protected inside each emirate and the percentage of protected areas by emirate.

Name	Area (km^2)	Nº of protected areas	Area protected (km^2)	Area protected (%)
Abu Dhabi	65.027	17	12.103	19
Ajman	268	2	31	12
Dubai	4.422	8	1.390	31
Fujairah	1.930	4	277	14
Ras al-Khaymah	2.758	2	270	10
Sharjah	2.664	17	420	16
Umm al-Quwain	820	0	0	0
