# Supplementary material for: An integrative assessment of the diversity, phylogeny, distribution, and conservation of the terrestrial reptiles (Sauropsida, Squamata) of the United Arab Emirates
Source: PLoS One. 2019 May 2;14(5):e0216273. doi: 10.1371/journal.pone.0216273 (PMC6497385; doi:10.1371/journal.pone.0216273)
Supplement: S8 Table — List of all 60 UAE species, showing their regional IUCN conservation category, their area of occupancy, area of occupancy inside protected areas, and the percentage of area of occupancy inside protected areas. Asterisks highlight the three introduced species. The introduced species have not been included in Fig 9 and in the analysis and discussion of the results of the gap analysis. (PDF) [file pone.0216273.s019.pdf]

S8 Table. Information for the gap analysis with distribution records with the AOO approach. List of all 60 species of the UAE, showing their regional IUCN conservation category, their area of occupancy, area of occupancy inside protected areas, and the percentage of area of occupancy inside a protected area. The asterisk highlights the three introduced species. Asterisks highlight the three introduced species. The introduced species have not been included in Fig. 9 and in the analysis and discussion of the results of the gap analysis

**Species**	**Regional IUCN**	**Total area (AOO) (km^2)**	**Area protected (km^2)**	**Area protected (%)**
Asaccus caudivolvulus	CR	8	0	0.0
Asaccus gardneri	LC	28	0	0.0
Asaccus margaritae	VU	4	0	0.0
*Indotyphlops braminus**	NA	16	0	0.0
Platyceps ventromaculatus	VU	32	0	0.0
Pristurus carteri	CR	8	0	0.0
Ptyodactylus ruusaljibalicus	LC	20	0	0.0
Pristurus celerrimus	LC	216	20	9.3
*Chalcides ocellatus ocellatus**	NA	80	8	10.0
Platyceps rhodorachis rhodorachis	LC	172	20	11.6
Eryx jayakari	LC	624	88	14.1
Teratoscincus keyserlingii	CR	216	32	14.8
Uromastyx aegyptia leptieni	VU	564	84	14.9
Pristurus minimus	LC	100	16	16.0
Acanthodactylus boskianus asper	NT	24	4	16.7
Asaccus gallagheri	LC	168	28	16.7
Trachydactylus hajarensis	LC	208	36	17.3
Hemidactylus robustus	LC	320	56	17.5
Stenodactylus arabicus	LC	844	152	18.0
Diplometopon zarudnyi	LC	284	52	18.3
*Hemidactylus flaviviridis**	NA	152	28	18.4
Cerastes gasperettii gasperettii	LC	512	96	18.8
Acanthodactylus haasi	DD	20	4	20.0
Stenodactylus slevini	LC	180	36	20.0
Phrynocephalus arabicus	LC	1040	212	20.4
Lytorhynchus diadema diadema	LC	392	80	20.4
Pseudoceramodactylus khobarensis	LC	132	28	21.2
Cyrtopodion scabrum	LC	192	44	22.9
Rhagerhis moilensis	LC	260	60	23.1
Pristurus rupestris-sp. 3	LC	512	120	23.4
Pseudocerastes persicus	LC	84	20	23.8
Acanthodactylus schmidti	LC	1424	356	25.0
Scincus mitranus	LC	984	248	25.2
Psammophis schokari	LC	516	132	25.6
Omanosaura jayakari	LC	124	32	25.8
Stenodactylus doriae	LC	740	196	26.5
Varanus griseus griseus	LC	472	128	27.1
Acanthodactylus gongrorhynchatus	LC	212	60	28.3
Mesalina adramitana	LC	272	80	29.4
Echis omanensis	LC	268	80	29.9
Phrynocephalus maculatus	LC	160	48	30.0
Scincus scincus conirostris	DD	40	12	30.0
Trapelus flavimaculatus	LC	360	108	30.0
Spalerosophis diadema cliffordii	LC	52	16	30.8
Pseudotrapelus jensvindumi	LC	236	76	32.2
Echis carinatus sochureki	LC	308	100	32.5
Bunopus tuberculatus	LC	1052	344	32.7
Ablepharus pannonicus	LC	12	4	33.3
Heremites septemtaeniatus	DD	12	4	33.3
Mesalina brevirostris	LC	272	96	35.3
Myriopholis macrorhyncha	LC	68	24	35.3
Uromastyx aegyptia microlepis	VU	324	116	35.8
Omanosaura cyanura	LC	100	36	36.0
Ptyodactylus orlovi	LC	180	72	40.0
Trachylepis tessellata	LC	56	24	42.9
Stenodactylus leptocosymbotes	LC	212	100	47.2
Hemidactylus persicus	VU	16	8	50.0
Telescopus dhara dhara	LC	28	16	57.1
Acanthodactylus opheodurus	DD	32	20	62.5
Acanthodactylus blanfordii	VU	8	8	100.0
