# Supplementary material for: An integrative assessment of the diversity, phylogeny, distribution, and conservation of the terrestrial reptiles (Sauropsida, Squamata) of the United Arab Emirates
Source: PLoS One. 2019 May 2;14(5):e0216273. doi: 10.1371/journal.pone.0216273 (PMC6497385; doi:10.1371/journal.pone.0216273)
Supplement: S9 Table — List of all 60 species of the UAE, showing their regional IUCN conservation category, their SPD area, the SPD area inside protected areas, and the percentage of the SPD area inside protected areas. The eight species that did not have the minimum of five records to infer their SDMs are underlined. In these cases, the SPD was calculated by the sum of all 4 km2 squares inside the minimum convex polygon of each species. Asterisks highlight the three introduced species. The introduced species have not been included in Fig 9 and in the analysis and discussion of the results of the gap analysis. (PDF) [file pone.0216273.s020.pdf]

S9 Table. Information for the gap analysis with distribution records with the species potential distribution (SPD) approach obtained with species distribution modeling (SDM). List of all 60 species of the UAE, showing their regional IUCN conservation category, their SPD area, the SPD area inside protected areas, and the percentage of the SPD area inside protected areas. The eight species that did not have the minimum of five records to infer their SDMs are underlined. In these cases, the SPD was calculated by the sum of all 4 km2 squares inside the minimum convex polygon of each species. Asterisks highlight the three introduced species. The introduced species have not been included in Fig. 9 and in the analysis and discussion of the results of the gap analysis.

Species	Regional IUCN	Total area (km^2)	Area protected (km^2)	Area protected (%)
Asaccus caudivolvulus	CR	8	0	0.0
Asaccus margaritae	VU	4	0	0.0
Platyceps ventromaculatus	VU	116	0	0.0
Pristurus carteri	CR	32	0	0.0
Asaccus gardneri	LC	1,156	52	4.5
Acanthodactylus boskianus asper	NT	2,152	164	7.6
Spalerosophis diadema cliffordii	LC	6,048	480	7.9
Telescopus dhara dhara	LC	6,028	500	8.3
Trachydactylus hajarensis	LC	5,700	508	8.9
Omanosaura cyanura	LC	5,836	612	10.5
Platyceps rhodorachis rhodorachis	LC	5292	580	11.0
Pristurus rupestris-sp. 3	LC	5,732	640	11.2
Pseudotrapelus jensvindumi	LC	6,508	736	11.3
Pristurus celerrimus	LC	5,172	592	11.4
Ptyodactylus ruusaljibalicus	LC	4,888	568	11.6
Echis omanensis	LC	5,628	656	11.7
Ptyodactylus orlovi	LC	8,948	1,068	11.9
Mesalina adramitana	LC	23,088	2,772	12.0
Asaccus gallagheri	LC	5,212	632	12.1
Trachylepis tessellata	LC	5,436	660	12.1
Omanosaura jayakari	LC	5,012	612	12.2
Pseudocerastes persicus	LC	3,260	416	12.8
Teratoscincus keyserlingii	CR	9,452	1,208	12.8
Acanthodactylus haasi	DD	46,260	5,932	12.8
Diplometopon zarudnyi	LC	46,552	6,188	13.3
Pristurus minimus	LC	13,264	1,776	13.4
Uromastyx aegyptia leptieni	VU	59,640	8,080	13.5
Echis carinatus sochureki	LC	50,424	7,068	14.0
Hemidactylus robustus	LC	48,160	6,940	14.4
Myriopholis macrorhyncha	LC	28,228	4,236	15.0
Pseudoceramodactylus khobarensis	LC	51,376	7,932	15.4
Stenodactylus slevini	LC	44,936	7,044	15.7
Trapelus flavimaculatus	LC	53,524	8,456	15.8
Mesalina brevirostris	LC	23,716	3,992	16.8
Scincus scincus conirostris	DD	20,712	3,552	17.1
Hemidactylus persicus	VU	1,032	180	17.4
Rhagerhis moilensis	LC	28,176	5,052	17.9
Lytorhynchus diadema diadema	LC	65,892	12,032	18.3
Heremites septemtaeniatus	DD	3,144	580	18.4
Cerastes gasperettii gasperettii	LC	65,284	12,544	19.2
Psammophis schokari	LC	78,364	16,032	20.5
Eryx jayakari	LC	74,416	15,272	20.5
Phrynocephalus arabicus	LC	71,012	14,684	20.7
Bunopus tuberculatus	LC	77,776	16,160	20.8
Stenodactylus doriae	LC	74,484	15,828	21.3
Varanus griseus griseus	LC	73,140	15,604	21.3
Acanthodactylus gongrorhynchatus	LC	72,716	15,592	21.4
Acanthodactylus schmidti	LC	73,900	15,856	21.5
Scincus mitranus	LC	72,040	15,672	21.8
Uromastyx aegyptia microlepis	VU	55,360	12,044	21.8
Cyrtopodion scabrum	LC	61,148	13,516	22.1
Stenodactylus arabicus	LC	64,784	15,088	23.3
Phrynocephalus maculatus	LC	57,864	13,728	23.7
Stenodactylus leptocosymbotes	LC	19,308	4,676	24.2
Acanthodactylus opheodurus	DD	29,596	8,516	28.8
Ablepharus pannonicus	LC	64	20	31.3
Acanthodactylus blanfordii	VU	8	8	100.0
