# Supplementary material for: An integrative assessment of the diversity, phylogeny, distribution, and conservation of the terrestrial reptiles (Sauropsida, Squamata) of the United Arab Emirates
Source: PLoS One. 2019 May 2;14(5):e0216273. doi: 10.1371/journal.pone.0216273 (PMC6497385; doi:10.1371/journal.pone.0216273)
Supplement: S10 Table — The numbers show the evolutionary uniqueness of each species with respect to other UAE reptiles in millions of years. Asterisks highlight the three introduced species. (PDF) [file pone.0216273.s021.pdf]

S10 Table. Evolutionary distinctiveness (ED) of each of the 60 species of UAE terrestrial reptiles. The numbers show the evolutionary uniqueness of each species with respect to other UAE reptiles in millions of years. Asterisks highlight the three introduced species.

**Species**	**Evolutionary distinctiveness (ED)**
Ablepharus pannonicus	68.5
Acanthodactylus blanfordii	20.1
Acanthodactylus boskianus asper	15.9
Acanthodactylus gongrorhynchatus	15.8
Acanthodactylus haasi	15.8
Acanthodactylus opheodurus	15.9
Acanthodactylus schmidti	20.1
Asaccus caudivolvulus	26.3
Asaccus gallagheri	30.5
Asaccus gardneri	26.3
Asaccus margaritae	30.5
Bunopus tuberculatus	46.1
Cerastes gasperettii gasperettii	47.4
*Chalcides ocellatus ocellatus**	68.5
Cyrtopodion scabrum	35.8
Diplometopon zarudnyi	141.0
Echis carinatus sochureki	22.6
Echis omanensis	22.6
Eryx jayakari	100.4
*Hemidactylus flaviviridis**	54.7
Hemidactylus persicus	34.1
Hemidactylus robustus	34.1
Heremites septemtaeniatus	55.9
*Indotyphlops braminus**	120.4
Lytorhynchus diadema diadema	35.4
Mesalina adramitana	32.6
Mesalina brevirostris	32.6
Myriopholis macrorhyncha	120.4
Omanosaura cyanura	50.4
Omanosaura jayakari	50.4
Phrynocephalus arabicus	33.1
Phrynocephalus maculatus	33.1
Platyceps rhodorachis rhodorachis	13.6
Platyceps ventromaculatus	13.6
Pristurus carteri	32.2
Pristurus celerrimus	66.4
Pristurus minimus	32.2
Pristurus rupestris-sp. 3	45.4
Psammophis schokari	33.4
Pseudoceramodactylus khobarensis	57.5
Pseudocerastes persicus	49.2
Pseudotrapelus jensvindumi	34.1
Ptyodactylus orlovi	42.7
Ptyodactylus ruusaljibalicus	42.7
Rhagerhis moilensis	33.4
Scincus mitranus	65.9
Scincus scincus conirostris	65.9
Spalerosophis diadema cliffordii	24.7
Stenodactylus arabicus	35.5
Stenodactylus doriae	14.1
Stenodactylus leptocosymbotes	14.1
Stenodactylus slevini	22.7
Telescopus dhara dhara	18.4
Teratoscincus keyserlingii	100.0
Trachydactylus hajarensis	35.8
Trachylepis tessellata	55.9
Trapelus flavimaculatus	84.2
Uromastyx aegyptia leptieni	62.8
Uromastyx aegyptia microlepis	62.8
Varanus griseus griseus	159.2
